# Convergent genome streamlining accompanies independent miniaturization in the world’s smallest fishes

**DOI:** 10.1073/pnas.2614605123

**Published:** 2026-10-14

**Authors:** Hiranya Sudasinghe, Michael Matschiner, Ralf Britz, Kevin W. Conway, Heok Hui Tan, Walter Salzburger, Catherine L. Peichel, Lukas Rüber

**Affiliations:** ^a^Natural History Museum Bern, Bern 3005, Switzerland; ^b^ Division of Evolutionary Ecology, Institute of Ecology and Evolution, University of Bern, Bern 3012, Switzerland; ^c^ Faculty of Biology, Ludwig Maximilian University of Munich, Planegg 82152, Germany; ^d^ Senckenberg Natural History Collections, Dresden 01109, Germany; ^e^ Department of Ecology and Conservation Biology and Biodiversity Research and Teaching Collections, Texas A&M University, College Station, TX 77843; ^f^ Lee Kong Chian Natural History Museum, National University of Singapore, Singapore 117377, Singapore; ^g^ Department of Environmental Sciences, Zoological Institute, University of Basel, Basel 4051, Switzerland; ^h^ Division of Aquatic Ecology and Evolution, Institute of Ecology and Evolution, University of Bern, Bern 3012, Switzerland

**Keywords:** body size, comparative genomics, miniaturization, macroevolution, Cypriniformes

## Abstract

Miniaturization, the reduction of adult body size to an extreme degree, has evolved repeatedly across vertebrates. Yet its genomic underpinnings remain poorly understood. Cypriniformes, the most species-rich order of freshwater fishes, contains multiple miniaturized lineages that have evolved via contrasting developmental processes. Proportioned dwarfs are tiny-bodied but otherwise morphologically similar to larger relatives, while progenetic miniatures exhibit developmental truncation and retain larval-like anatomical features into adulthood. Using a new time-calibrated phylogeny of 309 cypriniform species and comparative genomic analyses of 33 high-quality genome assemblies, we investigated the evolutionary history and genomic correlates of miniaturization across this order. Ancestral state reconstruction revealed multiple independent origins of both miniature types, with reversals to the nonminiature state rarely recovered and miniature origins nonrandomly distributed across the phylogeny. The origins of the two types of miniatures differed in their timing. Progenetic miniatures arose predominantly in the Eocene whereas proportioned dwarfs arose mainly within the Miocene period. Genome size variation across Cypriniformes has been overwhelmingly driven by polyploidy. However, progenetic miniatures showed a directionally consistent, though nonsignificant, trend toward genome size reduction. Comparative genomic analyses revealed that all three independently evolved progenetic miniature lineages share convergent signatures of repeat loss alongside genome-wide intron shortening, patterns absent in proportioned dwarfs. Our study provides the broadest evidence to date that progenetic miniaturization, despite independent origins, is underpinned by predictable structural genomic changes, revealing a fundamental link between developmental truncation and genome architecture in vertebrates.

## Significance

Miniaturization is widespread among vertebrates, yet its genomic basis remains poorly understood. In Cypriniformes, the most species-rich order of freshwater fishes, miniaturization has evolved repeatedly, producing two distinct forms: proportioned dwarfs, which closely resemble smaller versions of larger relatives, and progenetic miniatures, which retain larval traits due to developmental truncation into adulthood. By reconstructing the evolutionary history of miniaturization and performing comparative genomic analyses, we show that progenetic miniatures, but not proportioned dwarfs, exhibit consistent genome size reductions. Independently evolved progenetic lineages share convergent genomic signatures, including repeat loss and genome-wide intron shortening. These findings demonstrate that progenetic miniaturization is driven by predictable structural genomic changes, revealing a fundamental link between genome architecture and developmental truncation in vertebrates.

Miniaturization, the evolution of extremely small adult body size, offers a natural experiment for understanding how developmental, morphological, and genomic systems reorganize under strong selection (1). Across animals, miniaturization has repeatedly evolved alongside heterochronic shifts in developmental timing, often resulting in paedomorphosis: the retention of juvenile traits in reproductively mature adults (1, 2). Paedomorphosis occurs via two mechanisms: neoteny, where somatic development slows relative to reproductive maturation, and progenesis, where reproductive maturation accelerates relative to somatic growth (2). While neoteny is extensively documented in vertebrates (2–4), progenesis, despite its strong association with miniaturization (5), remains less well studied. Progenesis, however, has been implicated in the repeated evolution of miniaturization in invertebrates, including interstitial meiofauna and parasitic lineages (2, 6–9).

Among fishes, Cypriniformes is the most species-rich order of freshwater fishes with over 5,000 described species. This taxon provides an ideal system for studying miniaturization because it contains multiple independently derived miniaturized lineages with contrasting anatomical outcomes (10). Miniaturized fishes are conventionally defined as species reaching sexual maturity at or below a standard length of 26 mm, while often exhibiting paedomorphic features compared to larger relatives (11). Within Cypriniformes, two distinct morphological outcomes have been recognized: proportioned dwarfs, which are miniaturized but morphologically similar to larger relatives, and progenetic miniatures, which exhibit developmental truncation and retain larval-like anatomical features into adulthood (5, 10, 12–14). Four genera—*Paedocypris*, *Danionella*, *Sundadanio*, and *Fangfangia*—exhibit extreme progenesis, with *Paedocypris progenetica* representing the world’s smallest fish. It reaches sexual maturity at just under 8 mm standard length (15). These progenetic miniatures display striking, sexually dimorphic, morphological novelties not developed in proportioned dwarfs and nonminiature cypriniform fishes (5, 14–17). For example, the pectoral and pelvic girdles of male *Paedocypris* are highly modified and hypothesized to be a clasping organ (14, 15). The recurrent evolution of miniaturization and the presence or absence of novelties across progenetic miniature and proportioned dwarf cypriniform taxa provides a powerful comparative framework for investigating the evolutionary consequences of extreme body-size reduction.

Despite extensive morphological characterization, the genomic architecture underlying miniaturization in cypriniforms remains poorly understood. Recent work on *Paedocypris* revealed genomes (0.41 to 0.43 Gb) approximately one-third the size of zebrafish genomes (~1.5 Gb), characterized by intron shortening and reduced repetitive content (18). Whether this pattern is generally present across miniaturized lineages, however, remains unknown. Here, we make use of a new phylogenomic framework spanning all cypriniform families, including all known progenetic miniatures and most proportioned dwarfs (19), to reconstruct a fossil-calibrated temporal framework for Cypriniformes and investigate the evolutionary history of miniaturization across this order. This is complemented by newly generated reference genome assemblies for representatives of both types of miniatures (20). Together, these resources enable phylogenetic comparative analyses testing for correlations between body size and genome architecture across evolutionary time. Specifically, we test: 1) whether the frequency and phylogenetic distribution of independent miniaturization origins differ between the two types of miniatures; 2) whether the timing of these events reveals temporal clustering; 3) whether independent miniaturization events consistently associate with genome size reduction; and 4) whether shared genomic mechanisms, specifically repeat loss and intron shortening, underlie convergent genome streamlining. Our findings demonstrate that convergent genome streamlining through repeat loss and intron shortening is a predictable genomic consequence of progenetic miniaturization but not of proportioned dwarfism, revealing a fundamental genomic distinction between the two types of miniatures.

## Results

### A Time-Calibrated Phylogeny of Cypriniformes.

**Fig. 1.**
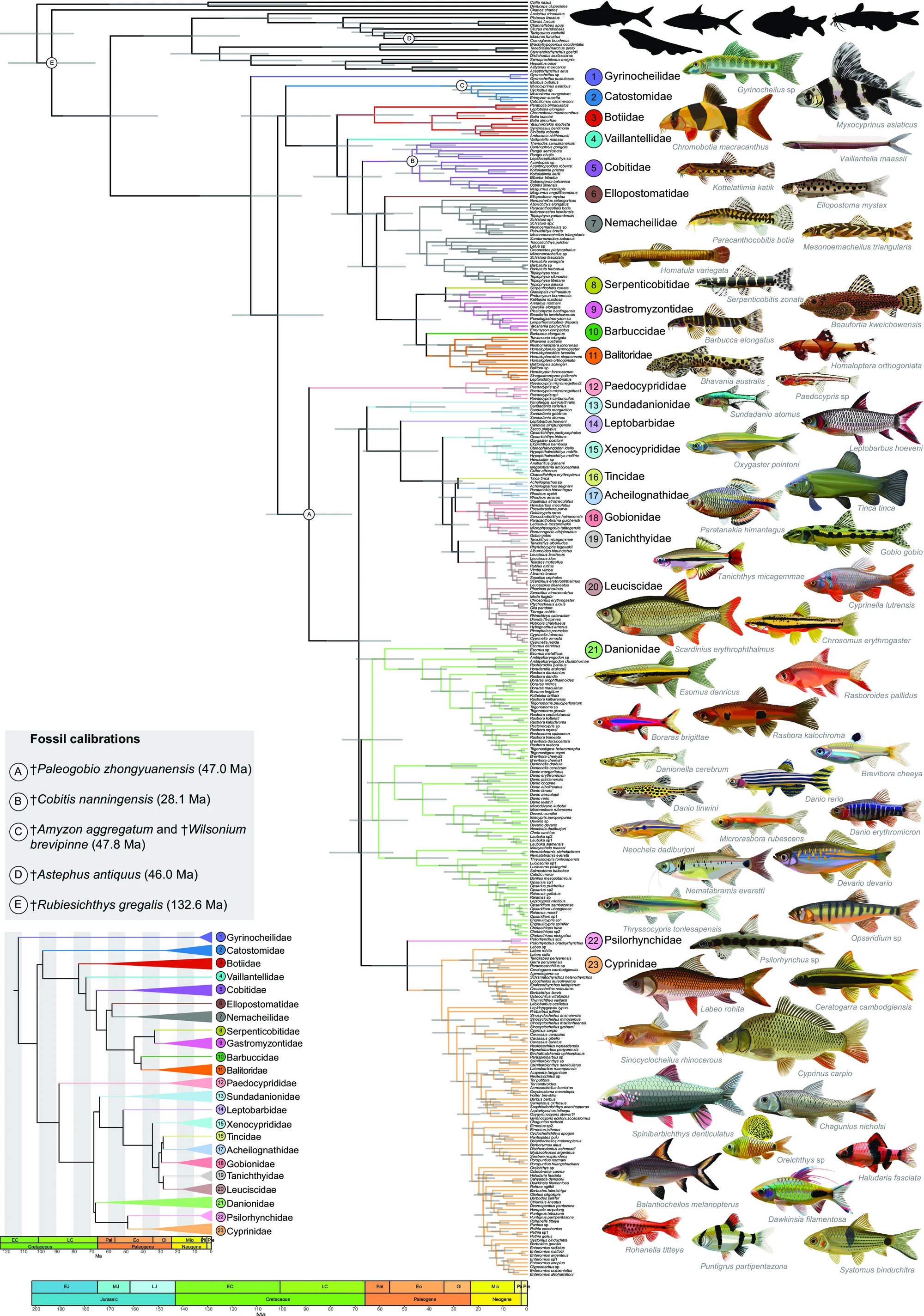
Time-calibrated phylogeny of Cypriniformes inferred from a dataset of combined first and second codon positions of BUSCO loci and ultraconserved elements (UCEs; 120 loci, 55,332 bp) under a relaxed clock model in BEAST 2. Branches are colored by family and node bars represent 95% HPD intervals in million years (Ma). Labeled nodes indicate fossil calibration points. *Inset* shows the same phylogeny collapsed to the family level. Fish illustrations by Nadeela Hirimuthugoda.

To provide a temporal framework for investigating the macroevolutionary history of miniaturization across Cypriniformes, we inferred a time-calibrated phylogeny using Bayesian divergence time estimation in BEAST 2. Our estimates support an early Cretaceous origin of crown Cypriniformes at approximately 112.0 Ma (95% HPD: 91.6 to 133.0 Ma; Fig. 1), which is in broad agreement with previously published molecular dating studies (e.g., refs. 21–25). The divergence estimates among the three major suborders fall within the late Early to early Late Cretaceous, with Cyprinoidei diverging from a clade combining Cobitoidei and Catostomoidei at 101.1 Ma (95% HPD: 83.2 to 120.4 Ma), Cobitoidei and Catostomoidei separating from each other at 97.8 Ma (95% HPD: 81.0 to 117.5 Ma), and the crown ages of Cyprinoidei and Cobitoidei estimated at 88.3 Ma (95% HPD: 71.6 to 106.6 Ma) and 77.0 Ma (95% HPD: 62.9 to 92.1 Ma), respectively.

At the family level, crown ages broadly fall into two temporal classes. Families with Paleogene origin include Botiidae (61.9 Ma), Danionidae (62.7 Ma), Cobitidae (46.5 Ma), Nemacheilidae (43.5 Ma), Sundadanionidae (47.2 Ma), Cyprinidae (34.7 Ma), Balitoridae (31.2 Ma), Gastromyzontidae (26.9 Ma), and Catostomidae (26.3 Ma). Families with Neogene crown age include Xenocyprididae (19.3 Ma), Gobionidae (19.4 Ma), Leuciscidae (16.9 Ma), and Acheilognathidae (15.2 Ma), with Paedocyprididae (24.4 Ma) straddling the Oligocene–Miocene boundary. For families represented by only one or two species, stem ages are reported in place of crown ages and ranged from 72.6 Ma (Vaillantellidae; Late Cretaceous) to 27.8 Ma (Tanichthyidae; late Oligocene to early Miocene). Complete stem and crown age estimates with 95% highest posterior density (HPD) intervals, together with comparisons of key divergence time estimates against previously published molecular dating analyses, are provided in *SI Appendix*, Table S1.

### Genome Size Diversity across Cypriniformes Reveals Exceptionally Compact Genomes in Loaches.

To characterize genome size variation across Cypriniformes and establish a baseline for comparative analyses of miniaturization, we estimated haploid genome sizes for 261 species using k-mer profiling. Genome size showed a strong positive correlation with repeat content (r = 0.687, *P* < 0.0001; *SI Appendix*, Fig. S1), indicating that repeat element accumulation is a primary driver of genome size variation across the order (26–28), and a weak negative correlation with GC content (r = −0.360, *P* < 0.0001; *SI Appendix*, Fig. S1), consistent with patterns reported in other teleosts (29).

Across the 261 species, the median haploid genome size was 752.7 Mb, with a sevenfold span ranging from 347.9 Mb in the balitorid loach *Homalopterula gymnogaster* to 2,509.8 Mb in the polyploid *Cycleptus* sp. (Catostomidae). Genome size within most families was remarkably conserved (intrafamilial fold variation: 1.0 to 1.5), with only Cobitidae, Cyprinidae, and Danionidae exceeding threefold variation (*SI Appendix*, Fig. S2).

A particularly striking finding were the consistently small genome sizes observed across multiple loach (Cobitoidei) families (*SI Appendix*, Fig. S2): Balitoridae (347.9 to 537.5 Mb), Barbuccidae (379.5 Mb), Gastromyzontidae (411.6 to 474.3 Mb), Vaillantellidae (427.5 Mb), Serpenticobitidae (440.3 Mb), and Ellopostomatidae (452.7 Mb), with several falling below or approaching the genome size of pufferfishes, including the genera *Fugu*, *Takifugu,* and *Tetraodon,* which have the smallest known vertebrate genomes ranging from 346.5 to 385.0 Mb (30, 31).

### Progenetic Miniaturization and Proportioned Dwarfism Differ in Their Frequencies and Times of Origin.

**Fig. 2.**
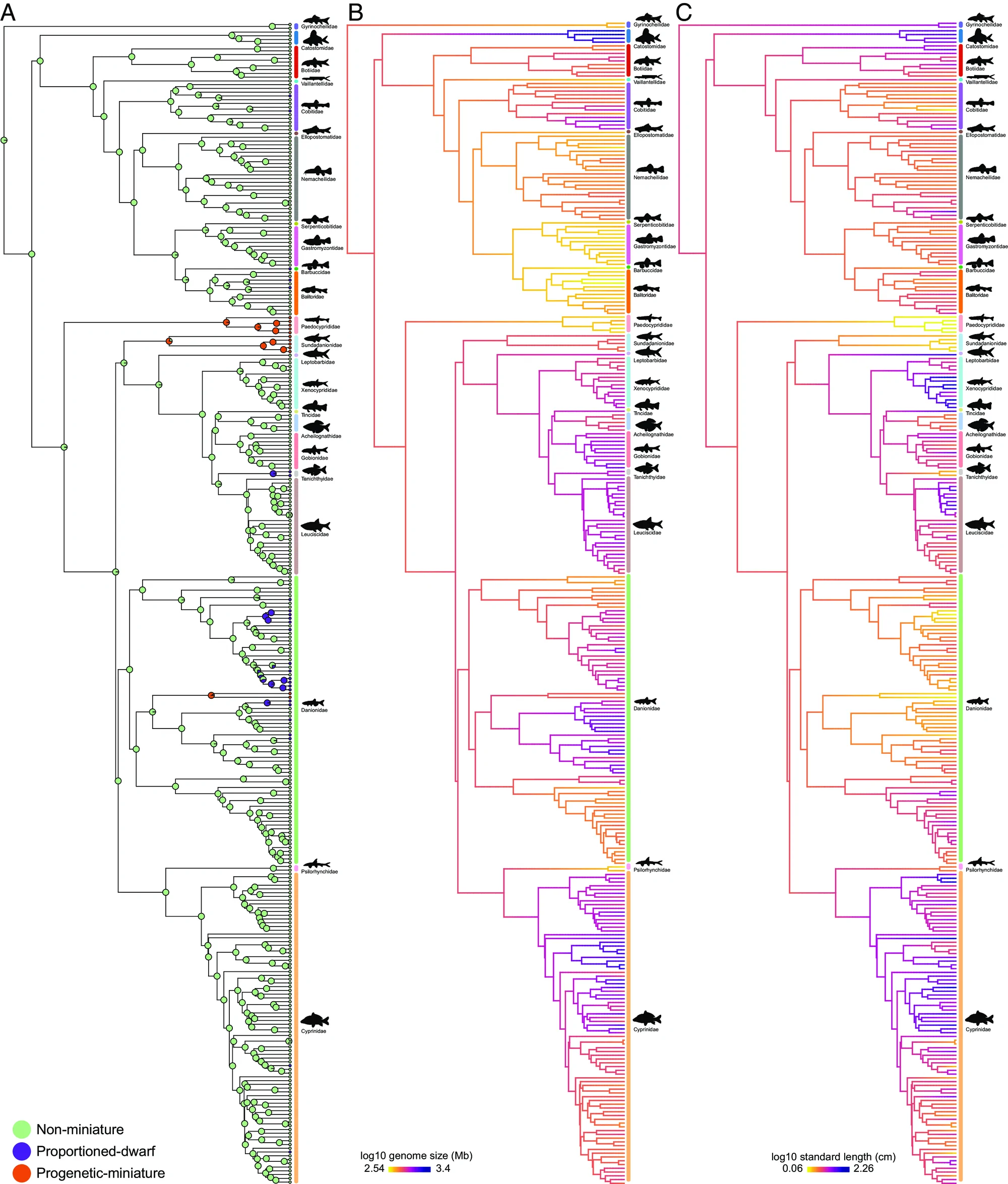
Ancestral state reconstructions of miniaturization, genome size, and body size across Cypriniformes plotted on the pruned time-calibrated phylogeny. (*A*) Stochastic character mapping of miniaturization status using an ER model across 1,000 reconstructions. Pie charts at nodes represent the posterior probability of each miniaturization state. Maximum likelihood ancestral state reconstruction of (*B*) log-transformed haploid genome size and (*C*) log-transformed standard length as a proxy for body size. In panels (*B* and *C*), warmer colors indicate smaller values and cooler colors indicate larger values of the respective log-transformed trait.

Stochastic character mapping on the time-calibrated phylogeny revealed that miniaturization is a derived condition within Cypriniformes, with the ancestral state at the root inferred as nonminiature (Fig. 2*A*). Across reconstructions, transitions from nonminiature to miniature states were recovered far more frequently than reversals. Progenetic miniaturization evolved rarely, with a median of four independent origins per reconstruction (95% range: 2 to 6), representing three independent origins across the four recognized genera (*Paedocypris*, *Danionella*, and *Sundadanio* + *Fangfangia*), all within Cyprinoidei. Proportioned dwarfism evolved far more frequently, with a median of 18 independent origins per reconstruction (95% range: 15 to 19), concentrated predominantly within Danionidae, but with additional origins in Cyprinidae, Tanichthyidae, Cobitidae, Balitoridae, and Barbuccidae. The two miniaturization types differed markedly in their inferred times of origin. Progenetic miniature origins were concentrated in the Eocene (median 46.0 Ma; 95% range: 38.2 to 52.1 Ma), whereas proportioned dwarf origins occurred predominantly in the Miocene (median 8.8 Ma; 95% range: 7.4 to 10.6 Ma).

### Progenetic Miniaturization but Not Proportioned Dwarfism Shows a Consistent Trend Toward Genome Size Reduction.

**Fig. 3.**
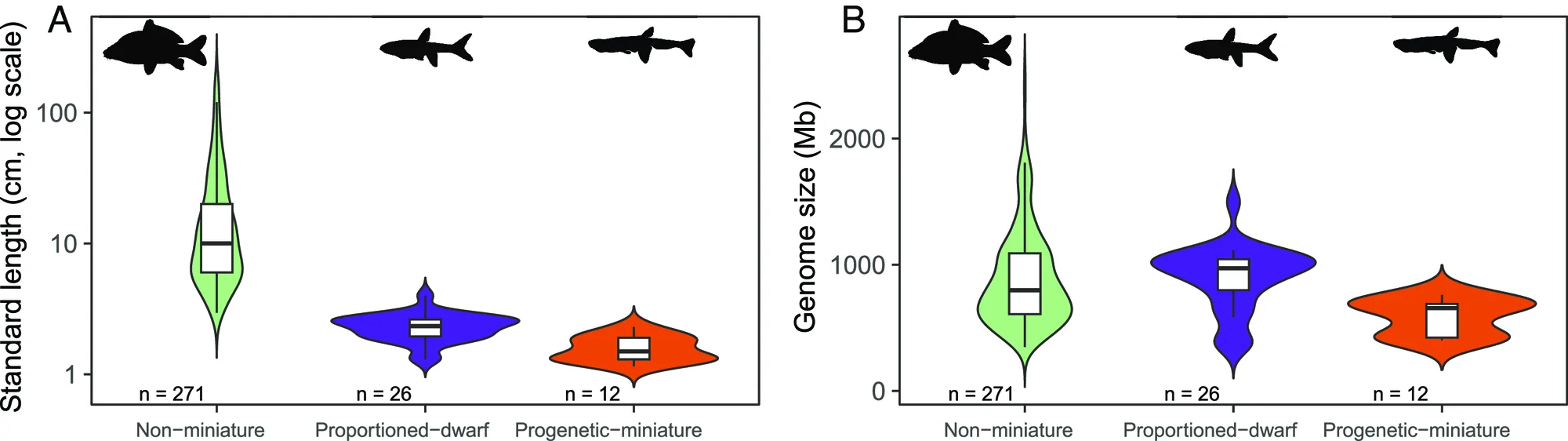
Distribution of (*A*) log-transformed standard length as a proxy for body size and (*B*) haploid genome size across nonminiature, proportioned dwarf, and progenetic miniature cypriniform species included in the phylogenetic comparative analyses.

Ancestral state reconstruction of genome size revealed recurrent expansions and reductions across the phylogeny (Fig. 2*B*). Loaches showed consistently smaller genome sizes than Cyprinoidei overall, with the clade comprising Serpenticobitidae, Gastromyzontidae, Barbuccidae, and Balitoridae exhibiting particularly marked genome size reduction. The non-Cobitoidei families Gyrinocheilidae and Psilorhynchidae also exhibited genome size reduction. Ancestral state reconstruction of body size revealed a broadly similar pattern, with body size and genome size decoupled in most clades, notably in Danionidae, where most species are small-bodied despite spanning a wide genome size range (Fig. 2*C*). Both traits showed strong phylogenetic signal (genome size: Pagel’s λ = 1.000, K = 1.110, *P* = 0.001; body size: Pagel’s λ = 0.946, K = 0.322, *P* = 0.001), though the contrasting K values indicate that genome size is more conserved within lineages while body size is more evolutionarily variable, consistent with the repeated independent evolution of miniaturization. Not surprisingly, phylogenetic generalized least squares (PGLS) regression of body size against miniaturization status revealed highly significant reductions in both progenetic miniatures (85% smaller than nonminiatures; *P* = 2.72 × 10^−7^: Fig. 3*A*) and proportioned dwarfs (72% smaller; *P* = 3.56 × 10^−11^: Fig. 3*A*).

PGLS regression indicated that miniaturization status did not significantly predict genome size in either miniaturization category across the full 309-species dataset (*SI Appendix*, Tables S2 and S3). Ploidy emerged as the overwhelmingly dominant predictor of genome size variation (β = 0.258, SE = 0.029, *P* < 2.2 × 10^−16^; R^2^ = 0.209), while body size was not a significant predictor after accounting for ploidy and miniaturization status. Proportioned dwarfs showed no meaningful difference in genome size relative to nonminiatures in any model. However, progenetic miniatures showed a consistent negative trend that was directionally consistent across all model structures and independent of ploidy and body size as covariates (β = −0.126, SE = 0.082, t = −1.533, *P* = 0.126). Progenetic miniatures had an approximate 25% genome size reduction relative to nonminiatures, which is equivalent to a reduction of roughly 190 Mb for a typical nonminiature cypriniform genome (Fig. 3*B*).

### Convergent Transposable Element (TE) Loss Underlies Genome Compaction in Progenetic Miniatures.

To test whether repeat loss represents a shared genomic mechanism of genome compaction across independently evolved miniature lineages, we annotated and compared TE content across a dataset of 33 high-quality cypriniform genome assemblies spanning miniature and nonminiature species. Progenetic miniatures occupied a consistently higher range of nonrepetitive DNA content (58.5 to 64.7%) compared to nonminiatures (34.3 to 74.4%; (*SI Appendix*, Fig. S3), and total repeat content showed a strong positive correlation with assembly size across all 33 species (r = 0.951, *P* < 2.2 × 10^−16^; *SI Appendix*, Fig. S4). PGLS regression indicated that while total TE content did not differ significantly between nonminiatures and all miniatures combined (β = 9.440, SE = 6.133, *P* = 0.134), the comparison restricted to progenetic miniatures vs. nonminiatures approached significance (β = 13.877, SE = 7.984, *P* = 0.092), corresponding to approximately 14% more TE content in nonminiature genomes.

**Fig. 4.**
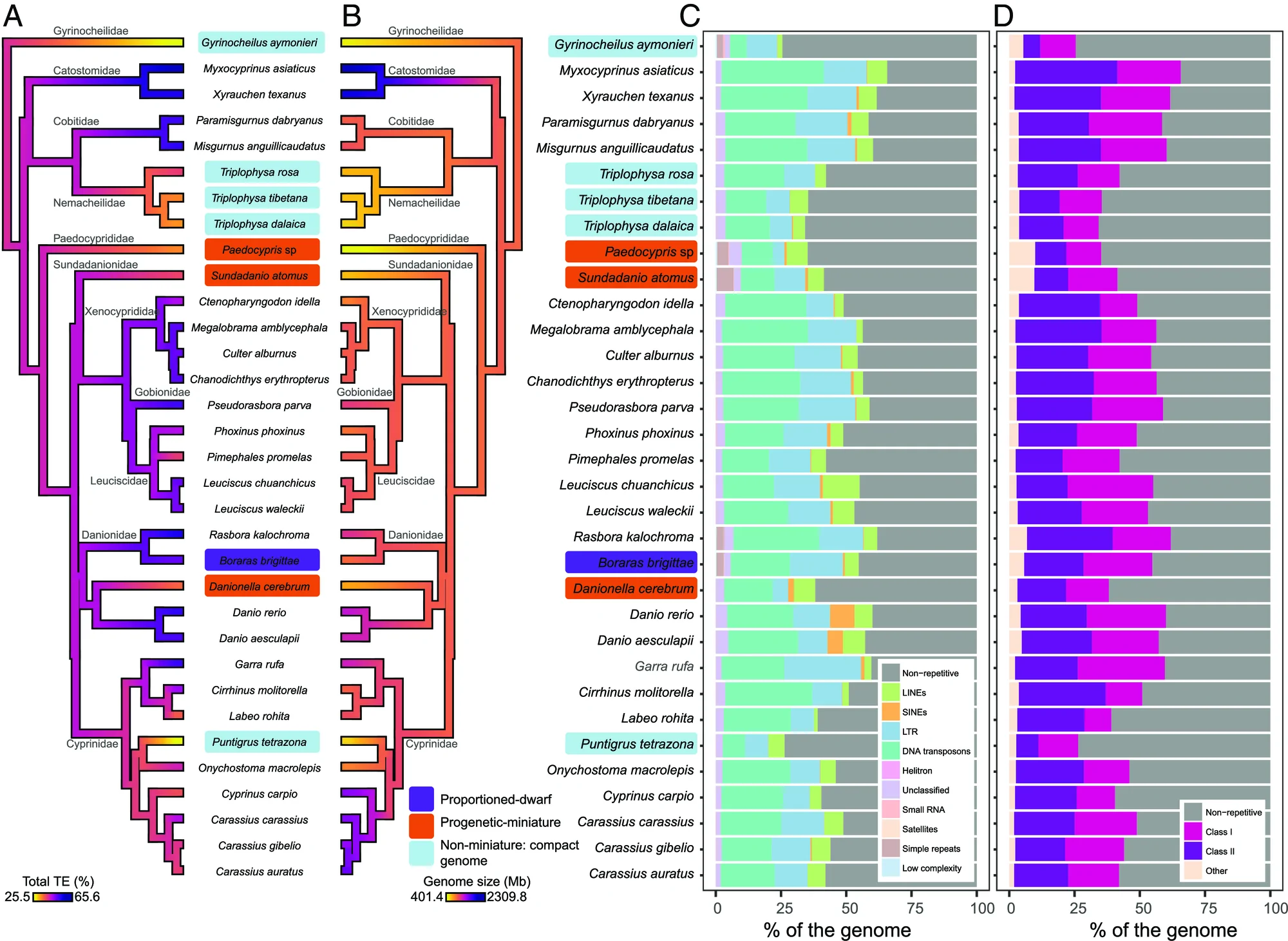
TE content and genome size variation across miniature and nonminiature cypriniform species in the 33-taxon comparative genomic dataset. Maximum likelihood ancestral state reconstruction of (*A*) total TE content as a percentage of the genome and (*B*) haploid genome size (Mb) plotted on the 33-taxon time-calibrated phylogeny. In both panels, warmer colors indicate lower values and cooler colors indicate higher values. Distribution of (*C*) major TE subclasses and (*D*) major repeat classes (Class I, Class II, and other repeats) as a percentage of the genome across miniature and nonminiature cypriniform species.

Maximum likelihood ancestral state reconstruction illustrated the convergent TE loss across all three independently evolved progenetic miniature lineages (*Paedocypris* sp., *Sundadanio atomus*, and *Danionella cerebrum*), each showing marked reductions in TE content relative to their closest nonminiature relatives (Fig. 4*A*). In contrast, the proportioned dwarf *Boraras brigittae* showed no comparable reduction, reinforcing the pattern that genome compaction is specifically associated with progenetic miniatures rather than proportioned dwarfs. However, TE loss was not exclusive to progenetic miniatures. Several nonminiature species including *Gyrinocheilus aymonieri*, nemacheilid loaches, and *Puntigrus tetrazona* also exhibited TE loss, consistent with the broader pattern of genome compaction observed across certain cypriniform lineages independent of miniaturization (Figs. 2*B* and 4 *A* and *B*).

At the repeat class level, Class II elements were significantly reduced in progenetic miniatures relative to nonminiatures (β = 10.507, SE = 4.876, *P* = 0.039), while Class I content did not differ significantly (β = 5.300, SE = 3.751, *P* = 0.168). Within each class, DNA transposons and LTR elements were both significantly reduced in progenetic miniatures (β = 10.554, SE = 4.875, *P* = 0.038; β = 7.579, SE = 2.984, *P* = 0.017, respectively), identifying these as the primary drivers of genome compaction in these lineages. Interestingly, other repeat content, driven primarily by simple repeats was significantly enriched in progenetic miniatures relative to nonminiatures (β = −3.207, SE = 1.035, *P* = 0.004; simple repeats: β = −2.819, SE = 0.701, *P* = 0.0004; see Fig. 4 *C* and *D*), suggesting that genome compaction in progenetic miniatures reflects a complex remodeling of repeat composition rather than a uniform depletion of all repetitive elements. Results for all repeat categories are provided in *SI Appendix*, Figs. S5 and S6 and Tables S4–S7.

To determine whether the depletion of TEs in progenetic miniature genomes reflects a reduction in recent transposition activity, a loss of older elements, or both, we examined the relative age distribution of TE insertions across the dataset using CpG-adjusted Kimura two-parameter (K2P) divergence landscapes (*SI Appendix*, Fig. S7). The three progenetic miniature species, together with the nonminiature species that exhibited TE loss in the ancestral state reconstruction (*G. aymonieri*, nemacheilid loaches, and *P. tetrazona*), showed broadly similar TE age distributions relative to the remaining nonminiature species, characterized by low proportions of both recent and intermediate divergence insertions, suggesting a sustained reduction in TE activity across both recent and intermediate timescales rather than a depletion confined to a specific period (*SI Appendix*, Fig. S7 *B* and *C*). In contrast, the proportioned dwarf *B. brigittae* showed a TE age distribution comparable to nonminiature species, consistent with the absence of overall TE depletion in this lineage.

PGLS comparisons across TE age classes supported this interpretation. Among intermediate divergence insertions (K2P 5 to 20%), LTR elements were significantly reduced in progenetic miniatures (β = 4.786, SE = 2.033, *P* = 0.025), while other repeat content was significantly elevated (β = −1.463, SE = 0.557, *P* = 0.014), with Class II elements and DNA transposons additionally approaching significance. Among recent insertions (K2P < 5%), Class II elements and DNA transposons showed higher levels in nonminiatures approaching significance. In contrast, LINEs were elevated in progenetic miniatures among recent insertions, potentially reflecting ongoing LINE activity in otherwise streamlined genomes. Among ancient insertions (K2P > 20%), LTR elements showed a trend toward reduction in progenetic miniatures (β = 1.937, SE = 1.083, *P* = 0.084), though no other repeat types differed significantly. Taken together, these results indicate that TE depletion in progenetic miniature genomes is not confined to a single temporal class but reflects a sustained and prolonged reduction in transposition activity spanning both recent and intermediate timescales, consistent with a genome-wide and temporally extended process of repeat loss rather than a recent or episodic purging of insertions. Full results for all TE age class comparisons are provided in *SI Appendix*, Figs. S8–S13 and Tables S4–S7.

### Intron Shortening Is a Consistent Genomic Signature across Progenetic Miniature Lineages in Cypriniformes.

**Fig. 5.**
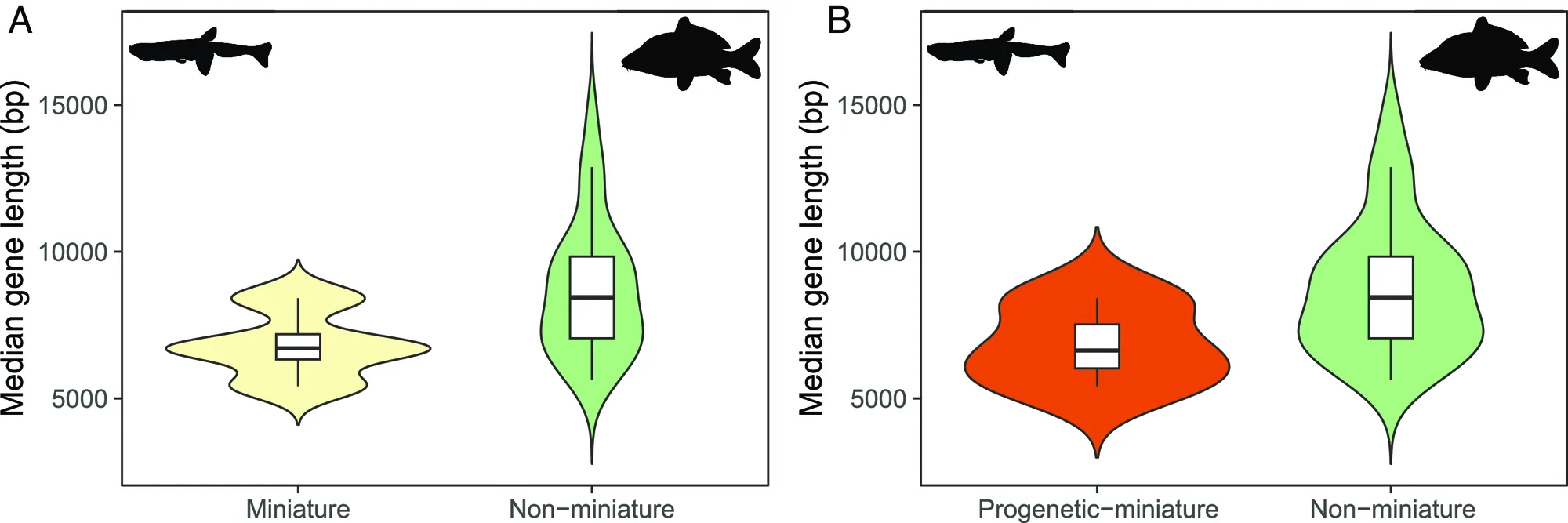
Distribution of median gene length (bp) across (*A*) miniature and nonminiature and (*B*) progenetic miniature and nonminiature cypriniform species included in the comparative genomic analyses.

To test whether intron shortening represents a shared genomic mechanism of genome compaction alongside TE loss, we examined gene length and intron length distributions across the 33-species comparative genomic dataset. Median gene lengths were marginally but nonsignificantly longer in nonminiature species relative to all miniatures combined (β = 2,433.5, SE = 1,291.6, *P* = 0.069) and to progenetic miniatures (β = 2,302.6, SE = 1,520.5, *P* = 0.140; Fig. 5), likely reflecting limited statistical power given the small number of progenetic miniature species.

**Fig. 6.**
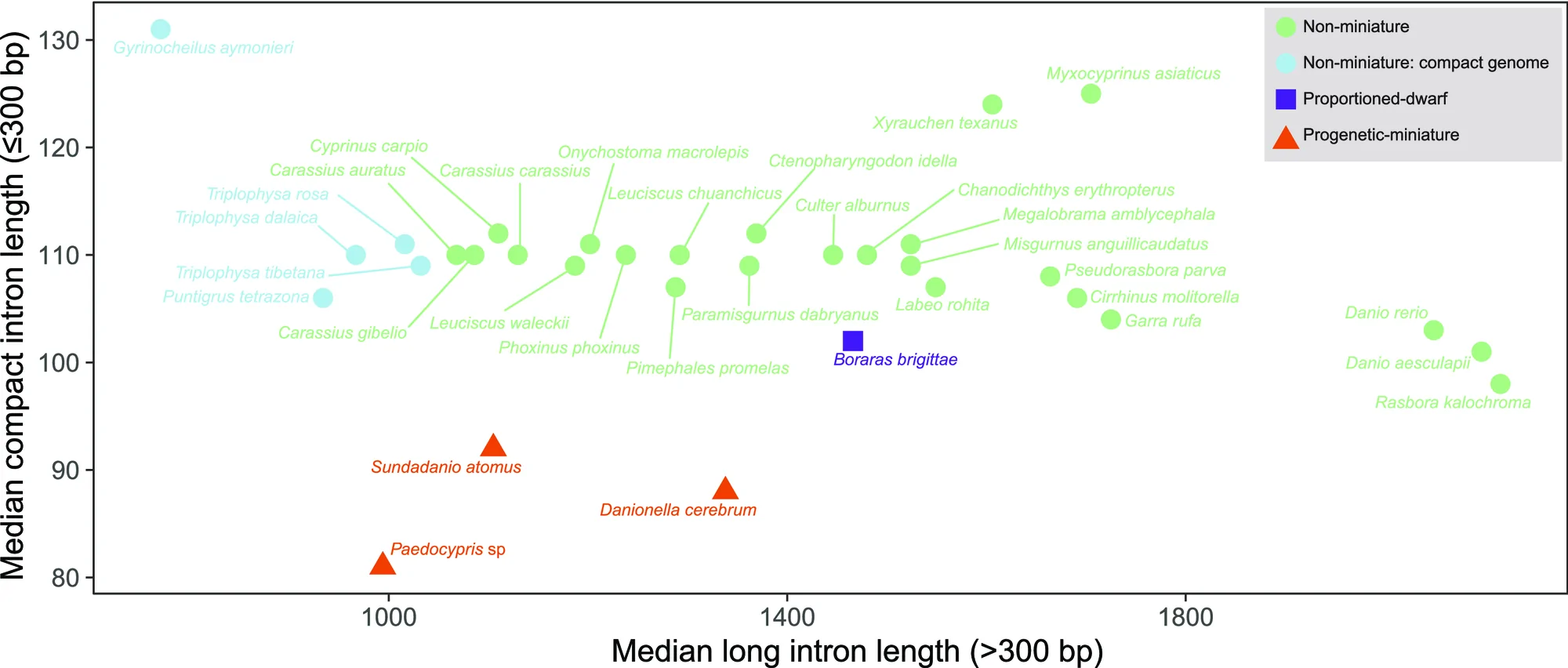
Distribution of median long intron length (>300 bp) vs. median compact intron length (≤300 bp) across miniature and nonminiature cypriniform species included in the comparative genomic analyses. Each point represents a species, colored by miniaturization status.

The most striking finding emerged from the comparison of intron length distributions by size class. Plotting median long intron length (>300 bp) against median compact intron length (≤300 bp) clearly separated all three progenetic miniature species from the remainder of the dataset (Fig. 6), suggesting convergent intron shortening of compact introns across independently evolved progenetic miniature lineages. The proportioned dwarf *B. brigittae* clustered with nonminiature species. Furthermore, the nonminiature species exhibiting TE loss and reduced genome sizes (*G. aymonieri*, nemacheilid loaches, and *P. tetrazona*) also clustered with the rest of the nonminiature species (Fig. 6). Notably, nonminiature species with compact genomes showed comparatively shorter long introns relative to other nonminiature species, suggesting that while these lineages undergo some degree of intron shortening, it is concentrated in long introns rather than in compact introns (Fig. 6). This suggests that intron shortening of compact introns is specifically associated with progenetic miniaturization and does not accompany TE-mediated genome compaction in nonminiature lineages in Cypriniformes.

PGLS analyses confirmed that intron length differences between progenetic miniatures and nonminiatures were most pronounced in compact intron size classes. Ultra-short introns (≤100 bp) and compact introns (≤300 bp) were significantly shorter in progenetic miniatures (β = 7.968, SE = 1.064, *P* = 2.405e-08; β = 20.513, SE = 3.435, *P* = 1.51e-06 respectively), while long introns (>300 bp) also differed significantly but less markedly (β = 429.04, SE = 201.78, *P* = 0.042). Intron shortening was not confined to specific positions in the genes. Compact introns showed pronounced differences across first (5′), last (3′), and middle intron positions, indicating a genome-wide rather than position-specific phenomenon, with the exception of single introns which did not differ significantly between groups (*SI Appendix*, Figs. S14–S17 and Tables S5 and S6).

In contrast to the pronounced intron length differences, the proportion of compact vs. long introns and the median number of introns per gene did not differ significantly between progenetic miniatures and nonminiatures (*SI Appendix*, Figs. S18 and S19), providing evidence for genome-wide intron shortening but not intron loss in progenetic miniature lineages.

## Discussion

Miniaturization, the reduction of adult body size to an extreme degree, is a recurrent theme across animal diversity, having evolved independently across numerous invertebrate and vertebrate lineages (1). The morphological consequences of miniaturization have been extensively documented (e.g., refs. 1, 2, and 32–34), yet its genomic underpinnings remain poorly investigated, with only a handful of studies addressing this question in invertebrates (e.g., refs. 35–37) and vertebrates (e.g., refs. 18, 38, and 39). Miniaturization is comparatively frequent in teleost fishes (10, 11, 40). Within Cypriniformes, the morphological and developmental correlates of miniaturization have been especially well characterized. Progenetic miniatures undergo profound developmental truncation, giving rise to striking morphological novelties not shown in their larger relatives, while proportioned dwarfs retain a normal developmental trajectory despite their reduced body size (e.g., refs. 5, 12, 14, 41, and 42). Here, we build on this foundation to provide one of the most comprehensive investigations to date of the evolutionary history and genomic correlates of miniaturization in a vertebrate order.

### Evolutionary Origins and Timing of Miniaturization.

Our time-calibrated phylogeny provides a temporal framework for tracing the evolutionary history of miniaturization across the order. Miniaturization evolved independently multiple times within Cypriniformes, with transitions predominantly unidirectional from nonminiature to miniature states. This unidirectionality is most readily explained for progenetic miniaturization, which involves profound and largely irreversible developmental truncation (1, 5, 14). The frequency of independent origins differed strikingly between the two types: progenetic miniaturization arose rarely, with only three independent origins all within Cyprinoidei, while proportioned dwarfism evolved far more frequently, concentrated predominantly within Danionidae. This nonrandom phylogenetic distribution suggests that the propensity for miniaturization is phylogenetically structured within Cypriniformes, indicating that certain lineages may possess developmental or ecological preconditions that facilitate body size reduction. The difference in frequency likely reflects the greater developmental constraints on progenetic miniaturization, which requires extreme heterochronic shifts resulting in the simultaneous truncation of multiple organ systems, compared to the simpler body size reduction characterizing proportioned dwarfism, which may face fewer developmental and genetic constraints (5, 14, 42). Furthermore, we document an apparent temporal dissociation between the two types: progenetic miniature origins were concentrated in the Eocene while proportioned dwarfs originated predominantly in the Miocene, suggesting that the two types not only differ morphologically and developmentally but may have originated in response to different macroevolutionary processes operating at different times in cypriniform history.

### Genome Size Evolution across Cypriniformes.

Our cypriniform genome size dataset represents one of the largest comparative genome size resources assembled for any vertebrate group, capturing an exceptional range of genomic diversity within a single order. Genome size within most families was remarkably conserved, consistent with patterns reported across teleosts more broadly (43, 44), with order-wide variation driven predominantly by polyploidy (45, 46). Against this background, miniaturization status did not significantly predict genome size across the full cypriniform dataset. Proportioned dwarfs showed no meaningful difference in genome size relative to nonminiatures, demonstrating that body size reduction alone does not entail genome compaction. This decoupling of genome size from body size reduction is consistent with the broader C-value enigma, reinforcing that genome size evolution is governed by mechanisms largely independent of organismal size or complexity (47, 48). Progenetic miniatures, however, showed a directionally consistent ~25% genome size reduction relative to nonminiatures, robust across all model structures and independent of ploidy and body size. Although this trend did not reach statistical significance, possibly reflecting the small number of independent progenetic miniature lineages against the broad genomic diversity of nonminiatures in our PGLS analyses, its consistency suggests a genuine biological tendency toward genome compaction in progenetic lineages. This extends the genome compaction previously reported in *Paedocypris* (18) to progenetic miniatures more broadly, while confirming that it is not a feature of proportioned dwarfism. This suggests that the mechanisms linking body size reduction and genome evolution differ fundamentally between the two types of miniaturization.

A particularly striking finding were the consistently small genome sizes across multiple loach families, with Serpenticobitidae, Gastromyzontidae, Barbuccidae, and Balitoridae showing particularly marked reductions. Given the monophyly of the clade comprising these four families, this pattern likely reflects a shared ancestral genome compaction event rather than independent reductions within the suborder. The ecological correlates of genome compaction across these lineages are intriguing. Loaches along with Psilorhynchidae (torrent minnows), which also had reduced genome sizes, are predominantly benthic fishes adapted to fast-flowing, well-oxygenated streams (49), while progenetic miniatures outside of *Danionella* are confined to the chronically resource-limited, acidic peat swamp forests of Southeast Asia (15, 50). Both groups therefore inhabit physiologically demanding environments, which has been linked to selection for smaller genomes through the relationship between cell size, nuclear DNA content, and metabolic rate (e.g., refs. 45, 47, and 51–53). Habitat demand alone, however, does not predict genome reduction: many nonminiature cypriniforms occupy similarly harsh environments without comparable genome-size reduction. A more general mechanism may instead underlie these patterns. Genome size is tightly coupled to cell size and, in turn, to cell-division and developmental rate across fishes and other vertebrates (e.g., refs. 47, 48, 52, and 54). Selection for smaller cells or faster development—whether driven by high aerobic demand, as plausibly in the benthic loaches of fast-flowing streams, or by accelerated maturation, as in progenetic miniatures—is expected to favor genome reduction. This relationship is general and independent of body size, and could account for the reduced genomes of both the nonminiature loaches and the progenetic miniatures through distinct selective pressures acting on a shared mechanism.

In the progenetic miniatures specifically, developmental rate offers a plausible candidate selective pressure. Extreme body-size reduction is thought to facilitate exploitation of structurally complex, resource-limited or unstable environments (1) and is often accompanied by fast life histories, consistent with selection for rapid development. Across amphibians, genome size is linked to developmental rate rather than to life-cycle mode per se: species with shorter developmental periods have smaller genomes, consistent with selection reducing genome size where rapid development is favored (48). Progenesis itself has been considered largely irrelevant to genome size evolution (54), presumably because it was viewed as a shift in the timing of maturation rather than in developmental rate. In the progenetic miniatures examined here, however, early maturation is accompanied by rapid, time-compressed development, which by the same rate-based logic would favor genome reduction. Our findings thus suggest that progenesis may, contrary to this view, represent a route to genome compaction. This hypothesis that progenesis drives genome compaction through its effect on developmental rate could be tested using comparative data on cell and nuclear size and on developmental and metabolic rates, which are currently lacking for these lineages.

### Genomic Signatures of Genome Streamlining in Progenetic Miniatures.

Genome compaction has been documented across diverse vertebrate and invertebrate lineages irrespective of body size, with compact genomes consistently showing predictable signatures of repeat depletion and structural simplification (e.g., refs. 31, 55, and 56). Our study asked whether miniaturization in Cypriniformes is associated with such genomic streamlining and whether the same mechanisms operate repeatedly across independently evolved lineages. The answer depends critically on the type of miniaturization: proportioned dwarfism shows no evidence of genome streamlining, while progenetic miniaturization is consistently associated with TE loss and intron shortening across all three independently evolved lineages.

The primary causes of TE depletion were reduction of LTR elements and DNA transposons which are highly active element classes capable of rapid genome expansion (57–62). Depletion of these elements suggests either reduced transposition activity, increased elimination of newly inserted elements, or both, consistent with mechanisms proposed for genome compaction in other vertebrates with streamlined genomes (31, 43, 53, 56). LTR elements are removed through ectopic recombination between their long terminal repeats, leaving a solo LTR (63), and DNA transposons are removed through imprecise excision and deletion (64). In vertebrates with large genomes, solo-LTRs occur at low levels, indicating that ectopic-recombination–mediated removal proceeds slowly (65); more rapid removal by this route could thus contribute to the streamlining seen here. The gradual action of these processes is consistent with the sustained depletion we recover across divergence classes.

Genome compaction in progenetic miniatures involves compositional remodeling rather than uniform repeat depletion, with simple repeats significantly enriched relative to nonminiatures, indicating mechanistically specific streamlining rather than a general suppression of all repetitive elements. TE depletion spanning both recent and intermediate divergence classes further suggests a sustained process operating over an extended evolutionary period in each progenetic lineage rather than a recent episodic event. Notably, TE loss also occurred in several nonminiature species with compact genomes. These lineages showed reductions in the same fast-expanding classes (LTR elements and DNA transposons) as progenetic miniatures, indicating that depletion of these classes is a shared route to genome compaction across cypriniform lineages rather than a feature unique to progenetic miniaturization. They differed, however, in the relative enrichment of simple repeats, which characterized progenetic miniatures alone. This is consistent with the expectation that non-self-replicating simple repeats, unlike TEs, are not targeted by TE suppression and so rise in proportional representation only where the self-replicating fraction is preferentially depleted (66). More broadly, the demographic conditions favoring such streamlining remain unknown for these lineages. Although efficient selection under large effective population sizes is predicted to purge TEs (66), recent animal-wide analyses find no association between effective population size and either genome size or TE content (67). The current effective population sizes of these miniatures, which may not reflect conditions at the time of miniaturization, have not been estimated, precluding such an analysis here.

The most compelling evidence for a genomic mechanism associated with progenetic miniaturization came from gene architecture analyses. Intron length distributions clearly separated the three progenetic miniature species from all other species in the dataset, including the proportioned dwarf, and nonminiature species with reduced TE content with small genomes. Intron shortening was consistently associated with progenetic miniaturization and did not accompany TE-mediated genome compaction in nonminiature lineages. This intron shortening was genome-wide, affecting first, last, and middle intron positions, and was most pronounced in compact intron size classes. This is consistent with the observation that teleost introns fall into two functional classes: compact introns containing primarily sequences necessary for efficient splicing, and long introns disproportionately harboring regulatory elements and conserved sequences maintained across vertebrates (68). The preferential shortening of compact introns suggests that introns most tolerant of length reduction are the primary targets of genome streamlining, while long introns with greater regulatory content show comparatively less reduction. Interestingly, nonminiature species with compact genomes showed a contrasting pattern, with shortening concentrated in long introns rather than compact introns, suggesting that different mechanisms may drive intron shortening in these lineages compared to progenetic miniatures. Whether this reflects active selection for shorter introns in progenetic miniatures imposed by developmental constraints on transcription speed and splicing efficiency (69, 70), or is driven primarily by deletion bias, remains to be determined.

These genomic signatures invite comparison with pufferfishes, which have the smallest known vertebrate genomes, yet are not miniaturized. Genome compaction in pufferfishes likewise combines TE/repeat reduction via suppression of transposable-element activity (31) along with genome-wide intron shortening rather than intron loss (30, 31, 71), This convergence between distantly related, ecologically dissimilar lineages suggests that vertebrate genome reduction draws on a shared repertoire of genome reduction mechanisms, centered on depletion of self-replicating TEs. One feature may nonetheless be distinctive: the progenetic-miniature signature localizes to compact introns, whereas other compact-genomed cypriniforms instead shorten long introns, and pufferfish intron reduction has so far been assessed only in aggregate.

In sum, progenetic miniaturization across Cypriniformes, despite arising independently, is underpinned by convergent genomic signatures of TE loss and intron shortening. Whether proportioned dwarfs exhibit functional genomic differences from nonminiatures despite lacking structural genome compaction remains to be investigated. Gene expression differences have been reported in miniature gobies (39) and whether similar patterns occur in cypriniform miniatures is worth exploring. The goby genus *Schindleria*, and the clupeoid genus *Sundasalanx*, offer additional striking cases of progenetic miniaturization among teleosts (72, 73). They provide independent examples to test whether the genomic signatures identified herein extend beyond Cypriniformes and whether there is a shared genomic basis of progenetic miniaturization in vertebrates. Taken together, our results reveal that it is the mode of developmental reduction, rather than the degree of body size reduction, that determines whether miniaturization leaves a structural genomic signature.

## Materials and Methods

### Molecular Dating of the Cypriniformes Phylogeny.

In our previous work, we presented a phylogenomic hypothesis for Cypriniformes with complete family-level representation and broad genus-level coverage, comprising 335 species (316 cypriniform species) that included approximately 30% of all described cypriniform genera (19). That study analyzed multiple genome-wide marker datasets using both concatenation-based and coalescent-aware phylogenetic approaches. In the present study, we extend this phylogenomic framework by inferring a molecular dated phylogeny of Cypriniformes, providing a temporal context for lineage diversification and a necessary foundation for downstream phylogenetic comparative analyses of miniaturization.

As a basis for molecular dating, we selected the topology of the ASTRAL-IV species tree inferred from the combined first and second codon positions of the Benchmarking Universal Single-Copy Orthologs (BUSCO.NT.C12) and ultraconserved element (UCE) nucleotide dataset in Sudasinghe et al. (19). This dataset comprises 1,167 loci with a total aligned length of 541,983 bp. We chose this topology because it showed high overall congruence across alternative phylogenomic inference methods in the original study.

Molecular dating was performed in BEAST v2.7.7 (74) on the fixed ASTRAL-IV topology (75), using a completeness-selected subset of 120 loci (55,332 bp) to reduce computational demand. We applied a Birth–Death tree prior (76) and an Optimized Relaxed Clock (77), with divergence times calibrated using six undisputed Ostariophysi fossils as minimum-age constraints and a secondary constraint on the crown age of Otocephala (23). We ran eight independent MCMC chains and assessed convergence from effective sample sizes in Tracer (78), combining post–burn-in posteriors to generate a maximum clade credibility tree (MCC) (79). Full details of the fossil calibrations, priors, run settings, and convergence diagnostics are provided in *SI Appendix*, Table S8 and *SI Appendix*, Supplementary Information).

### K-mer Based Genome Size Estimation.

We estimated genome size by k-mer profiling of 275 short- and long-read datasets from Sudasinghe et al. (19), using GenomeScope 2.0 (80) at k = 21 and k = 31 (Dataset S1). After excluding 14 samples with poor model fit or uncertain identity, the final dataset comprised 261 genomes with complete family-level and extensive genus-level coverage of Cypriniformes (*SI Appendix*, Fig. S2, Tables S9 and S10). Estimates from k = 21 and k = 31 were strongly concordant, and we used k = 31 for all downstream analyses. K-mer–based estimates correlated strongly with independent cytometric measurements from the Animal Genome Size Database (r = 0.791, *P* < 0.0001; *SI Appendix*, Fig. S1 and Table S11), confirming their suitability for comparative analysis (81). Detailed profiling, ploidy handling, filtering, and validation procedures are detailed in *SI Appendix*, Supplementary Information 1.

### Phylogenetic Comparative Analyses of Miniaturization.

Within Cypriniformes, two distinct morphological outcomes of miniaturization have been recognized: proportioned dwarfs, which are miniaturized but retain an overall anatomical structure similar to that of larger relatives, and progenetic miniatures, which have undergone profound developmental truncation and retain larval-like anatomical features into adulthood (10, 13, 14). Progenetic miniaturization within Cypriniformes is confined to all included species of the four genera *Paedocypris*, *Danionella*, *Sundadanio*, and *Fangfangia*.

To test whether miniaturization evolved multiple times independently within Cypriniformes and to evaluate whether these events clustered temporally and/or reveal phylogenetic patterning, we performed ancestral character state reconstruction of type of miniaturization on the time-calibrated phylogeny inferred in the present study (see *Molecular Dating* section). The MCC tree was first pruned to remove outgroup taxa and seven additional cypriniform species that exhibited poor model fit during k-mer genome profiling. The resulting pruned tree comprised 309 cypriniform species, of which 12 are progenetic miniatures, 26 are proportioned dwarfs, and 271 are nonminiature species (*SI Appendix*, Table S12). This sampling encompassed all four recognized genera of progenetic miniatures and nearly all known genera of cypriniform proportioned dwarfs (*SI Appendix*, Table S13).

Miniaturization status was coded as a discrete trait with three states: nonminiature, proportioned dwarf, and progenetic miniature. Ancestral state reconstruction was performed using stochastic character mapping implemented in the make.simmap function of the R package phytools (82). We selected an equal-rates (ER) model of state transitions after comparing it against symmetric (SYM) and all-rates-different (ARD) models by corrected Akaike Information Criterion (AIC). Although ARD received the highest AIC weight, its fitted transition matrix was degenerate, with rates into and out of the progenetic-miniature state estimated at approximately zero, indicating nonidentifiability given the small number of progenetic miniature origins. This instability also affected SYM and was robust to the choice of root prior (*SI Appendix*, Table S14). Under the ER model we generated 1,000 stochastic maps. Results were then summarized and visualized by averaging posterior probabilities of ancestral states across all 1,000 simulations. To ensure that the inferred numbers and ages of miniaturization origins reflected divergence-time uncertainty rather than resting on a single chronogram, we repeated this analysis across 100 trees randomly sampled from the post–burn-in BEAST posterior, performing 1,000 stochastic maps on each under the same ER model (100,000 maps in total). For each tree, we identified all transitions from the nonminiature state to a miniature state (progenetic miniature or proportioned dwarf) and recorded the number and median age of origins of each type. We report the median and 95% range of these per-tree values across the 100 sampled trees.

To evaluate whether genome size reduction is a lineage-specific feature of *Paedocypris* or a genomic correlate of progenetic miniaturization, or a generality of miniaturization across Cypriniformes, we mapped genome size and body size as continuous traits onto the pruned 309-species time-calibrated phylogeny. Genome sizes for species in our k-mer profiling dataset were obtained from the GenomeScope 2.0 analyses described above (k = 31); for species represented by publicly available genome assemblies not included in the k-mer profiling dataset, genome sizes were obtained from the corresponding NCBI genome pages. Maximum reported standard length was used as a proxy for body size and was compiled from published literature for each species. Both traits were log10 transformed prior to all analyses.

Ancestral states for log10 transformed genome size and log10 transformed standard length were inferred using maximum likelihood ancestral character state reconstruction via the function fastAnc in the phytools package (82), and results were visualized using the package’s contmap function to produce continuous-trait phylogenetic heat maps.

Before conducting formal comparative tests, we quantified phylogenetic signal for both genome size and body size to assess the degree to which trait variation reflects shared evolutionary history. Two complementary metrics were calculated using the phylosig function in phytools: Pagel’s λ (83) and Blomberg’s K (84).

To characterize the mode of evolution underlying genome size and body size variation across Cypriniformes, we compared the fit of four evolutionary models using the fitContinuous function implemented in the R package geiger (85). For each trait, we fitted a Brownian motion (BM) model, an Ornstein–Uhlenbeck (OU) model, an early burst (EB) model, and a white noise (WN) model (*SI Appendix*, Table S15). Model support was compared using Akaike Information Criterion (AIC) scores, with ΔAIC > 2 considered indicative of a meaningful difference in fit (86). To test whether genome size differed significantly between miniaturized and nonminiaturized lineages while accounting for phylogenetic nonindependence among species, we fitted PGLS regression models using the phylolm package (87). Miniaturization status was included as a categorical predictor variable. To evaluate the relative contributions of miniaturization type, ploidy, and body size to genome size variation, we fitted a series of models incorporating these predictors individually and in combination, including models with interaction terms between body size and miniaturization status. Models were fitted separately under two evolutionary process assumptions: a BM model, in which trait variance accumulates proportionally to branch lengths via random drift (*SI Appendix*, Table S2), and an OU model, which incorporates stabilizing selection toward a trait optimum and may better capture the constrained evolution of genome size if functional or developmental limits exists between miniature and nonminiature lineages (*SI Appendix*, Table S3). Model support was compared using AIC scores, with ΔAIC > 2 considered indicative of a meaningful difference in fit (*SI Appendix*, Table S16).

All comparative analyses were conducted under two complementary grouping schemes: i) all miniature species combined (progenetic miniatures and proportioned dwarfs) vs. nonminiatures, and ii) progenetic miniatures vs. proportioned dwarfs vs. nonminiatures. This allowed us to distinguish genomic signatures that are broadly shared across miniaturization types. All analyses were conducted in R version 4.3.3 (88).

### Comparative Genomics of Miniaturization.

Malmstrøm et al. (18) reported two primary mechanisms underlying genome compaction in *Paedocypris*: reduced repetitive element content and intron shortening. In the present study, we tested whether these genomic signatures are consistently associated with miniaturization in a broader comparative framework. Specifically, we evaluated whether independent miniature lineages exhibit parallel patterns of genome compaction. We compiled a dataset of 33 high-quality reference genomes with available gene annotations, of which 31 are pseudo-chromosome-level assemblies, representing 11 cypriniform families spanning all four recognized suborders (*SI Appendix*, Table S17). This sampling includes four genomes generated in Sudasinghe et al. (20), with the remaining assemblies obtained from NCBI.

The dataset includes four miniature species: *Paedocypris* sp., *S. atomus*, *D. cerebrum*, and *B. brigittae*. The first three are progenetic miniatures, whereas *B. brigittae* represents a proportioned dwarf. The remaining 29 genomes correspond to nonminiature cypriniform species. Using this dataset, we investigated patterns of TE content and gene architecture across lineages.

Comparative analyses were conducted under two grouping schemes: i) all miniature species (combining progenetic miniatures and proportioned dwarfs) vs. nonminiatures, and ii) progenetic miniatures only vs. nonminiatures.

### TE Annotation and Analysis.

All genomes were annotated for TEs using the same pipeline to ensure comparability across species. For each genome, TEs were annotated de novo using Extensive de novo TE Annotator (EDTA) (89) to construct species-specific repeat libraries. EDTA was run with the --sensitive 1 option to enable RepeatModeler-based discovery of additional repeat families (90) and with --anno 1 to perform whole-genome TE annotation following library construction. The resulting custom TE libraries were subsequently used to mask each genome assembly using RepeatMasker with the slow search option to maximize sensitivity (91).

For each species, the CpG-adjusted Kimura two-parameter (K2P) divergence from the consensus sequence of each repeat family was estimated using the RepeatMasker utility script calcDivergenceFromAlign.pl, applied to the .align files generated during masking (92). These divergence estimates were used to characterize the genomic landscape of TE insertions and to approximate the relative age distribution of TE activity within each genome. In TE divergence landscapes, peaks correspond to periods of elevated TE accumulation. Low-divergence copies represent more recent insertions and highly diverged copies reflect older, inactive elements (53, 57, 61). We then tested whether miniaturized lineages exhibited distinct TE age distributions compared to nonminiatures by comparing the proportion of insertions in three divergence classes, used here as proxies for relative insertion age: recent (K2P < 5%), intermediate (K2P within 5 to 20%), and ancient (K2P > 20%). These thresholds were chosen to broadly capture distinct phases of TE activity, ongoing or recent transposition, historically active periods, and older inactive elements respectively.

The resulting .divsum files were processed using custom R scripts to quantify the genomic proportion occupied by nonrepetitive DNA and by major TE and repeat categories, including total repeats, class I (LTR, LINEs, SINEs) repeats, class II (DNA transposons, helitrons) repeats, other repeats (i.e., all repeats except class I and class II), retroelements, total interspersed repeats, LINEs, SINEs, LTR elements, DNA transposons, helitrons, small RNAs, satellites, simple repeats, low-complexity repeats, and unclassified repeats, and to generate TE divergence landscapes. Visualization was performed using the ggplot2 package (93).

### Comparative Analysis of Gene Architecture.

To ensure consistency across annotations, we first extracted the longest isoform for each gene from all genome annotations using the script agat_sp_keep_longest_isoform.pl from AGAT (Dainat, 2022). Proteome completeness and annotation consistency were then assessed using OMArk (94), which evaluates proteome quality by comparing each proteome to an inferred ancestral gene repertoire reconstructed for the focal lineage and reports both completeness and consistency based on lineage-appropriate gene family assignments. OMArk single-copy completeness and consistency metrics did not reveal any systematic bias between annotation sources or between miniature and nonminiature species (*SI Appendix*, Fig. S20).

We focused our gene architecture analyses on overall gene length and intron length distributions, as these metrics are most resilient to differences in annotation methodologies across genome sources. While exon lengths are highly conserved due to functional constraints on protein-coding sequences (95–97), estimates of exon lengths, particularly at gene boundaries, are sensitive to UTR annotation practices, which could vary between NCBI and de novo annotations. In contrast, intron lengths exhibit substantial biological variation and are less affected by annotation pipeline differences, as intron boundaries are defined by conserved splice sites (69, 95, 97, 98).

From the filtered GFF annotation files, we separately extracted gene and exon coordinates for each genome and processed these datasets in R for downstream analyses. For gene-level analyses, when applicable, features were filtered to retain only protein-coding genes (gbkey = Gene; gene_biotype = protein_coding), and all RNA genes were excluded. To remove biologically implausible models (95, 97), genes shorter than 150 bp were discarded. The longest gene in the dataset (2,462,016 bp), however, was retained as it falls in the known range for the longest genes among vertebrates (97). For each species, summary statistics of gene length distributions were then calculated.

Intron coordinates were inferred from the filtered exon coordinates for each gene. Introns shorter than 30 bp or longer than 1,000,000 bp were excluded as biologically implausible (95, 97). For each gene, total intron length was calculated, and introns were further classified by position as first (5′), last (3′), middle, or single introns. Because intron length distributions across cypriniform genomes were bimodal, with a first peak around ~80 bp, an antimode near ~300 bp, and a second peak around ~1,000 bp (*SI Appendix*, Fig. S21), introns were grouped into two primary size classes: compact introns (≤300 bp) and long introns (>300 bp). Compact introns were further subdivided into ultra-short introns (≤100 bp) and short introns (101 to 300 bp). We quantified the proportion of compact and long introns, calculated the median number of introns per gene among multi-intron genes, and summarized intron length distributions overall, by size class, by positional category, and by the combination of size class and position.

To evaluate potential biases introduced by differences in annotation sources, we compared length metrics between genomes obtained from NCBI and those generated in Sudasinghe et al. (20). Median gene lengths and intron lengths did not show systematic differences between annotation sources (*SI Appendix*, Figs. S22–S24), confirming that these metrics are comparable across our dataset.

### Phylogenetically Informed Comparative Analyses.

To test for associations between miniaturization and genomic traits while accounting for evolutionary relationships among species, we analyzed TE content and gene architecture metrics in a phylogenetic comparative framework. The time-calibrated phylogeny inferred in this study (see *Molecular Dating* section) was pruned to retain only the 33 species represented in the comparative genomics dataset.

Three species with available genome assemblies (*Xyrauchen texanus*, *Paramisgurnus dabryanus*, *Cirrhinus molitorella*) were not sampled in the original 335-species molecular dating analysis. For *X. texanus*, we used *Catostomus commersoni* as a phylogenetic proxy, justified by phylogenetic analyses placing *Xyrauchen* as nested within *Catostomus* rather than as a distinct lineage (99, 100). Similarly, *P. dabryanus* and *Misgurnus* do not recover as distinct clades in available phylogenies (101, 102), supporting the use of *Misgurnus mizolepis* as a proxy. For *C. molitorella*, a sister group relationship with *Labeo* (103, 104) justified its replacement by the most phylogenetically proximate *Labeo* species in our dataset, minimizing the divergence introduced by this substitution. Additionally, we replaced several species in the time tree with congeneric species within the same genus to match available genome assemblies: *Garra periyarensis* with *G. rufa*, *Leuciscus leuciscus* with *L. chuanchicus*, *L. idus* with *L. waleckii*, and *Gyrinocheilus* sp. with *G. aymonieri*. The resulting pruned phylogeny with 33 tips retained the divergence times and branch length estimates from the full molecular dating analysis. To infer the evolutionary trajectory of total TE% and genome size, we performed maximum likelihood ancestral character state reconstruction using the function fastAnc implemented in the phytools package.

Before conducting comparative tests, we quantified phylogen (105) etic signal for each genomic trait to assess the extent to which trait variation reflects shared evolutionary history vs. independent evolution. The phylogenetic signal was quantified using Pagel’s λ and Blomberg’s K (*SI Appendix*, Table S4).

To test whether genomic traits differed significantly between miniaturized and nonminiaturized lineages while controlling for phylogenetic nonindependence, we fitted PGLS regression models. For each genomic trait, we fitted separate models (BM model and OU model) with miniaturization status as a categorical predictor variable (*SI Appendix*, Tables S5 and S6). Model support was compared using AIC scores, with ΔAIC > 2 considered indicative of a meaningful difference in fit (*SI Appendix*, Table S7). Given the limited number of miniaturized species in our dataset (n = 4), we report uncorrected *P*-values but interpret results conservatively, emphasizing effect sizes and consistency across biologically related metrics.

## Supplementary Material

Appendix 01 (PDF)

Dataset S01 (XLSX)

## Data Availability

Supporting data of the study are available on supporting information and Zenodo at https://doi.org/10.5281/zenodo.23008328 (105). Publicly available datasets used in this study are cited in the supporting information and are accessible from their original repositories.
