# Supplementary material for: Convergent genome streamlining accompanies independent miniaturization in the world’s smallest fishes
Source: Proc Natl Acad Sci U S A. 2026 Oct 14;123(42):e2614605123. doi: 10.1073/pnas.2614605123 (PMC13684242; doi:10.1073/pnas.2614605123)
Supplement: Supplementary file 1 [file pnas.2614605123.sapp.pdf]

PNAS

Supporting Information for

Convergent genome streamlining accompanies independent miniaturization in the world's smallest fishes

Hiranya Sudasinghe[1,2], Michael Matschiner[3], Ralf Britz[4], Kevin W. Conway[5], Heok Hui Tan[6], Walter Salzburger[7], Catherine L. Peichel[2], Lukas Rüber[1,8]

[1]Natural History Museum Bern, 3005 Bern, Switzerland
[2]Division of Evolutionary Ecology, Institute of Ecology and Evolution, University of Bern, 3012 Bern, Switzerland
[3]Faculty of Biology, Ludwig Maximilian University of Munich, 82152 Planegg, Germany
[4]Senckenberg Natural History Collections, 01109 Dresden, Germany
[5]Department of Ecology and Conservation Biology and Biodiversity Research and Teaching Collections, Texas A&M University, TX 77843, USA
[6]Lee Kong Chian Natural History Museum, National University of Singapore, 117377 Singapore
[7]Zoological Institute, Department of Environmental Sciences, University of Basel, 4051 Basel, Switzerland
[8]Division of Aquatic Ecology and Evolution, Institute of Ecology and Evolution, University of Bern, 3012 Bern, Switzerland

***Corresponding author email:** lukas.ruber@nmbe.ch

This PDF file includes:

Supporting text
Figures S1 to S24
Tables S1 to S17
Legends for Datasets S1

Other supporting materials for this manuscript include the following:

Datasets S1

Supplementary Information
Molecular dating of the Cypriniformes phylogeny

Molecular dating was performed under a Bayesian framework using BEAST v2.7.7 (1). Given the substantial computational demands of molecular dating with large phylogenomic datasets, we reduced the alignment to a subset of 120 loci (55,332 bp) selected based on completeness across taxa from the original 1,167-locus dataset. The ASTRAL-IV species tree inferred from the full BUSCO.NT.C12 and UCE dataset was provided as a starting tree, and its topology was fixed by removing all topological operators from the XML configuration file (2).

We implemented a Birth-Death model (3) as the tree prior, and a Gamma model of among site rate variation with four rate categories, coupled with the Optimized Relaxed Clock (ORC) model (4) as the clock model. Fossil calibrations were selected following a review of the published Ostariophysi fossil record, and only fossils considered to be undisputed and convincing representatives of their respective clades were included. All fossil calibrations were used exclusively to specify minimum ages for the respective clades, by applying uniform prior distributions ranging from the fossil age to an arbitrarily selected, unrealistically old upper end of 250.0 Ma. Additionally, the crown age of Otocephala was constrained according to the timeline of Hughes et al. (5), applying a log-normal distribution prior with a mean (in real space) of 193.5 Ma and a standard deviation of 0.04 (2.5% quantile: 179 Ma; 97.5% quantile: 209 Ma).

We conducted eight independent Markov-chain Monte Carlo (MCMC) runs, each for at least 50 million generations, including a burn-in of 30%, and sampled parameters and trees every 10,000 generations. Convergence and mixing were assessed by examining effective sample size (ESS) values in Tracer v.1.7.2 (6). In the combined run, ESS values exceeded 200 for all parameters except two GTR substitution-rate parameters (rateAG, ESS = 183; rateCG, ESS = 194); all time and clock parameters were well sampled, including tree height (ESS = 6,985), all calibrated node ages (ESS = 746–8,621), and the ORC relaxed-clock parameters (ESS = 431–1,066) (SI Appendix, Table S8). The posterior tree distribution (after removing the burn-in proportions) from each run were combined using LogCombiner, and summarized using TreeAnnotator to generate a maximum clade credibility (MCC) tree with median node heights (7).

To contextualize our divergence time estimates and assess their congruence with the existing literature, we compared key inferred node ages across Cypriniformes with those reported in previously published molecular dating studies of Cypriniformes (SI Appendix, Table S1).

Fossil calibrations used in Bayesian divergence time estimation, including taxonomic assignment, minimum age, and justification for inclusion. Excluded calibrations and the reasons for their exclusion are also documented.

Our fossil calibration comprised the following ingroup fossils:

†*Paleogobio zhongyuanensis* Zhou 1990 as a stem representative of Cyprinoidei (Early Eocene, Fanxian, China, minimum age 47.0 Ma) (8, 9).

†*Palaeogobio zhongyuanensis* is an imperfectly known fossil described by Zhou (9) based on an articulated almost complete skeleton from the Middle Miocene of the Shanwang Basin in China. We have no way of independently verifying the accuracy of its taxonomic assignment but note that (8) list this species as the earliest member of the Cyprinidae. We used a conservative approach treating this fossil as *incertae sedis* in Cyprinoidei and therefore used this fossil to calibrate the node of the last common ancestor of this taxon.

†*Cobitis nanningensis* Chen, Liao & Lei 2015 as a stem representative of Cobitidae (Early-Middle Oligocene, lover part of Yongning Formation, minimum age 28.1 Ma) (10) .

†*Cobitis nanningensis* was described by (10) from Early-Middle Oligocene deposits of Nanning Basin, Guangxi Zhuang Autonomous Region, southern China. This oldest fossil cobitid species is based on a series of 1.8–3 mm long bifid spines, interpreted by the authors to represent cobitid suborbital spines. We agree that this type of bifid suborbital spine is unique among bony fishes and serves as a sufficiently convincing synapomorphy to assign this fossil fragment to the family Cobitidae and potentially to the genus *Cobitis* (10). We therefore calibrated the node of the last common ancestor of all Cobitidae with this fossil.

†*Amyzon aggregatum* Wilson 1977 and **†*Wilsonium brevipinne*** (Cope, 1893) as stem representatives of Catostomoidei (early Eocene, Allenby formation and Horsefly beds in British Columbia, Canada, minimum age 47.8 Ma) (11, 12).

†*Wilsonium brevipinne* was originally described as a member of †*Amyzon* by (12) and later transferred to its own genus by (13) based on the results of a phylogenetic analysis that placed this species as the sister group to all remaining members of Catostomidae, inclusive of the Asian taxa *Myxocyprinus* and *Plesiomyxocyprinus*. There is little doubt that †*Amyzon* and †*Wilsonium* belong to Catostomidae because they exhibit the pectinate pharyngeal teeth on ceratobranchial 5 characteristic of this family (11, 13). We therefore calibrated the node of the last common ancestor of all Catostomidae with one of these two fossils from the same time period.

Our fossil calibration comprised the following outgroup fossils:

†*Astephus antiquus* (Leidy 1873) constraining the split between *Ictalurus furcatus* and *Cranoglanis bouderius* (Early Eocene, Green River Formation, minimum age 46.0 Ma) (14, 15).

†*Astephus antiquus* was originally described as a member of *Pimelodus* by (14) based on Eocene material from the Green River Bridger formations. In the same year, (16) transferred Leidy's species to the newly established †*Astephus*, a subgenus of †*Rhineastes* (Ictaluridae). Since description, †*A. antiquus* has been considered as either member of Ictaluridae (15–18), Ariidae (19) or the monogeneric †Astephidae (20). The study of (20) presented alternative hypotheses on the placement †*Astephus* (including †*A. antiquus*) within sisoroid catfishes resulting from the analyses of different datasets, including: (1) inside of Ictaluridae based on a morphological dataset of 209 characters (20: figure9a); and (2) as sister taxon to a larger group of catfishes including members of Ictaluridae, Schilbeidae, Bagridae, Siluridae and Plotosidae based on a combined dataset of morphological and molecular characters (20: figure 7). In this second hypothesis, *Cranoglanis* is placed as the sister taxon to the monophyletic group that includes †*Astephus* plus the aforementioned families of catfishes, and not as the sister taxon of Ictaluridae, as is commonly reported (ref herein). This second hypothesis stems from the analysis of a combined dataset with large amounts of missing data for †*Astephus* and other extinct taxa and we consider the first hypothesis that places †*Astephus* as part of a monophyletic group that also includes all members of Ictaluridae and *Cranoglanis* as more plausible.

†*Rubiesichthys gregalis* Wenz 1984 as a stem representative of Gonorynchiformes (Early Cretaceous, El Montsech and Las Hoyas (Spain), minimum age 132.6 Ma) (21, 22).

†*Rubiesichthys gregalis* was originally described by (22) from the Lower Cretaceous of Montsech in Spain and subsequently assigned to the family Chanidae by (23, 23) after the study of additional material from the type locality and Las Hoyas, Spain. This systematic placement has not been challenged and we have used †*Rubiesichthys* to calibrate the node uniting *Chanos* and Otophysi (i.e. the last common ancestor of Ostariophysi).

We refrained from using the below listed fossils that have sometimes been used as calibration points in molecular studies. In our opinion, morphological re-examination of these fossils is urgently needed before they can be used as reliable calibration points in molecular studies.

†*Tischlingerichthys viohli* Arratia 1997
This is a fossil fish from the Late Jurassic of Bavaria, Germany. It was described by (24) based on a single specimen of about 126 mm standard length. It has since been used in numerous molecular analyses to calibrate time trees starting with (25). In the original paper (24) suggested that (p. 100) "at present I interpret †*Tischlingerichthys* as a probably ostariophysan because certain shared synapomorphies…"

The analysis of (24), however, has a number of issues. The first issue concerns the approach. (24) performed two different sets of analysis, one with a more inclusive taxon set with 32 terminal taxa, most of them represented by fossils and four fossil outgroups. The various analyses of this set differed in the inclusion of one additional fossil to test the influence of these on the resulting trees but were otherwise the same. The only ostariophysan taxa in all these analyses are †*Gordichthys*, a fossil chanid, and *Chanos*. No Otophysi were included in the analysis, and many of the traditionally used synapomorphies of Ostariophsi were not considered thus greatly limiting the significance of the result. These analyses recovered †*Tischlingerichthys* as the sister group to †*Gordichthys* plus *Chanos*.

The second set of analyses to "test the position of †*Tischlingerichthys* among ostariophysans" considered only five terminal taxa plus †*Tischlingerichthys* and a "combined" all-zero outgroup. Again, according to her own admission (p. 99), "Numerous ostariophysan characters which are unknown in fossils were not considered." In this restrictive analysis, *Tischlingerichthys* was recovered as the sister group to Gonorynchiformes or as the sister group to Otophysi.
The lack of sufficient overlap in terms of characters and taxa makes these two analyses unsuitable to evaluate the phylogenetic position of †*Tischlingerichthys*.

The second issue about the analyses of (24) concerns the characters and their description. It is beyond the scope of this paper to discuss all characters and their problems in detail, but we note here that (26) already commented on this issue.

We are thus not convinced by the evidence presented in (24) that †*Tischlingerichthys* is a stem group ostariophysan (24: fig. 68b) or stem group chanid (24: fig. 68c) and conclude that a re-study of this taxon is needed before it can be used as a reliable calibration point.

†*Santanichthys diasii* (Silva Santos 1958)
Originally described in the genus †*Leptolepis*, as †*L. diasii*, it was subsequently transferred to the genus †*Santanichthys* and considered to be a clupeomorph. This tiny fish, rarely exceeding 30 mm standard length was restudied by (27) who claimed it was a member of the otophysan order Characiformes. Their conclusion was met with considerable skepticism and (28) summarized their criticism and concluded that †*Santanichthys* is a stem Ostariophysan or Otophysan. Recently, (29) confirmed this stating that the "alignment of *Santanichthys* with the Otophysi clade is controversial at least...and there is no reason to retain this taxon as a crown Otophysi." As in the case of †*Tischlingerichthys* a restudy and reinterpretation of the skeletal characters of this tiny fish will be necessary to place it with confidence into the teleost tree. We therefore decided not to use †*Santanichthys* as a calibration point in our study.

†*Jianghanichthys hubeiensis* (Lei, 1977)
An Eocene fossil from China that was originally assigned to the family Cyprinidae and subsequently considered a catostomid fossil. (30) classified it in its own family Jianghanichthyidae. The analyses of Liu et al. (30) remained inconclusive as to where this fossil belongs among Cypriniformes and we therefore did not use it as a calibration point.

†*Sooinichthys varii* Alvarado-Ortega, Otero & Mayrinck 2025.
A small fish of up to 97.3 mm standard length was recently described from Lower Cretaceous marine limestone deposits in Mexico (29). The species was interpreted as a member of the Otophysi, a conclusion based on the reported presence of a Weberian apparatus in these heavily

crushed and fractured specimens. Their condition of preservation leaves ample room for interpretation and speculation, and we disagree with the identification of many of the key bone elements. A critical reinterpretation of the crucial characters of †*Sooinichthys* is beyond the scope of this paper, but we suspect that this fish could be a clupeomorph. In favour of this hypothesis is for example the presence of structures visible at the ventral midline in (29: fig. 4a), that we think represent ventral scutes, one of the unique synapomorphies of clupeomorphs. Because of the drastically different interpretation of where this fish belongs phylogenetically, we refrain from using it as a calibration point.

K-mer based genome size estimation

The phylogenomic dataset of Sudasinghe et al. (31) comprises 370 genome assemblies representing Cypriniformes, including 95 publicly available genome assemblies and 275 newly assembled genomic resources. Of the newly assembled genomes, 253 were Illumina short-read assemblies (31), four were high-quality reference genomes generated using long-read sequencing (32), and the remaining 18 consisted of publicly available raw Illumina short-read sequencing datasets downloaded from the NCBI Sequence Read Archive (31). We used these 275 datasets for k-mer–based genome profiling and genome size estimation in the present study.

K-mer profiling and genome characterization were conducted using KMC v3.0 (33) for Illumina short-read datasets and Meryl v.1.4.1 (34) for PacBio long-read datasets. For all datasets, k-mer spectra were generated using both k = 21 and k = 31 to evaluate the robustness of genome size estimates between the two k-mer lengths. GenomeScope 2.0 (35) was then used to infer genome size, heterozygosity, and repeat content from the k-mer frequency distributions (Dataset S1).

During GenomeScope 2.0 analyses, diploid species were modelled using ploidy = 2, whereas known polyploid cypriniform taxa were modelled using their reported ploidy levels (p = 4 or p = 6). Ploidy levels were assigned based on published literature. For a subset of samples, GenomeScope 2.0 failed to converge on the correct haploid (1n) k-mer coverage peak when using default parameters. In these cases, genome profiles were visually inspected, and a biologically informed coverage prior (-l) was manually specified to guide model convergence (36). Resulting estimates were re-evaluated to ensure consistency with expected k-mer distributions and biologically plausible genome characteristics.

Eleven samples exhibited poor model fit and elevated read error rates during genome profiling, and the taxonomic identities of an additional three samples were considered uncertain based on (31). These 14 samples (SI Appendix, Table S9) were excluded from all downstream analyses. The final dataset therefore comprised 261 genomes, providing complete family-level coverage and extensive genus-level representation across Cypriniformes (SI Appendix, Table S10; Fig. S2).

GenomeScope 2.0 reports genome size estimates as ranges. Therefore, for each genome, mean genome size estimates for k = 21 and k = 31 were calculated by averaging the minimum and maximum haploid genome length estimates returned by GenomeScope 2.0. Similarly, GenomeScope 2.0 reports repeat content estimates as ranges. The proportion of repeat content in the genome was estimated by dividing the minimum repeat length by the minimum haploid genome length and the maximum repeat length by the maximum haploid genome length. Then mean repeat content for each k-mer size was calculated as the average of these two values. Genome size estimates derived from k = 21 and k = 31 were strongly concordant for all species (SI Appendix, Fig. S2). Consequently, we used the genome size estimates derived from k = 31, which provided the most complete dataset, for all downstream comparative analyses.

Genome size variation within each cypriniform family was quantified as fold variation, calculated as the ratio of the maximum to minimum genome size estimates observed within each family. To explore potential correlates of genome size evolution, we evaluated the relationships between genome size, repeat content, and genomic GC content. GC content estimates were obtained from the corresponding genome assemblies (31, 32).

To assess the concordance between k-mer–based genome size estimates and traditional cytometric measurements, we compiled all available genome size data for Cypriniformes from the Animal Genome Size Database (37), accessed on 11 November 2024. This dataset was manually curated to match the taxonomic framework used in our study and filtered to retain only genera represented in our genomic dataset. Several records with questionable or ambiguous genome size estimates were excluded, resulting in a curated dataset of 216 cytometric genome size estimates spanning the cypriniform families Gyrinocheilidae, Catostomidae, Balitoridae, Botiidae, Cobitidae, Gastromyzontidae, Nemacheilidae, Acheilognathidae, Cyprinidae, Danionidae, Gobionidae, Leuciscidae, Tincidae, and Xenocyprididae (SI Appendix, Table S11). For both the k-mer–based and cytometric datasets, the mean genome size was then calculated at the genus level. Genome size estimates were log-transformed prior to comparisons, and correlations between datasets were assessed using Pearson correlation tests implemented in R. Statistical significance was evaluated at $p = 0.05$.

K-mer-based genome size estimates showed a strong and significant positive correlation with cytometric genome size measurements compiled from the Animal Genome Size Database ($r = 0.791$, $p < 0.0001$; SI Appendix, Fig. S1), confirming that our k-mer-based estimates are consistent with traditional flow cytometric and Feulgen densitometry measurements and are suitable for broad comparative analyses (38).

References

1. R. Bouckaert, *et al.*, BEAST 2.5: An advanced software platform for Bayesian evolutionary analysis. *PLoS Comput Biol* **15**, e1006650 (2019).
2. A. J. Drummond, M. A. Suchard, D. Xie, A. Rambaut, Bayesian Phylogenetics with BEAUti and the BEAST 1.7. *Molecular Biology and Evolution* **29**, 1969–1973 (2012).
3. T. Gernhard, The conditioned reconstructed process. *Journal of Theoretical Biology* **253**, 769–778 (2008).
4. J. Douglas, C. L. Jiménez-Silva, R. Bouckaert, StarBeast3: Adaptive parallelized Bayesian inference under the multispecies coalescent. *Systematic Biology* **71**, 901–916 (2022).
5. L. C. Hughes, *et al.*, Comprehensive phylogeny of ray-finned fishes (Actinopterygii) based on transcriptomic and genomic data. *Proc Natl Acad Sci USA* **115**, 6249–6254 (2018).
6. A. Rambaut, A. J. Drummond, D. Xie, G. Baele, M. A. Suchard, Posterior summarization in Bayesian phylogenetics using Tracer 1.7. *Systematic Biology* **67**, 901–904 (2018).
7. J. Heled, R. R. Bouckaert, Looking for trees in the forest: summary tree from posterior samples. *BMC Evol Biol* **13**, 221 (2013).
8. M. Chang, G. Chen, Fossil Cypriniformes from China and its adjacent areas and their palaeobiogeographical implications. *SP* **295**, 337–350 (2008).
9. J. Zhou, The Cyprinidae fossils from the middle Miocene of Shanwang Basin. *Vertebrata Palasiatica* **28**, 95–127 (1990).
10. G.-J. Chen, L. Wei, L. Xue-Qiang, First fossil cobitid (Teleostei: Cypriniformes) from early-middle Oligocene deposits of South China. *Vertebrata Palasiatica* **53**, 299–309 (2015).
11. M. V. H. Wilson, *Middle Eocene freshwater fishes from British Columbia* (Life Science Contribution Royal Ontario Museum, 1977).
12. E. D. Cope, Fossil fishes from British Columbia. *Proceedings of the Academy of Natural Sciences of Philadelphia* **45**, 401–402 (1893).
13. J. Liu, M. V. H. Wilson, A. M. Murray, A new catostomid fish (Ostariophysi, Cypriniformes) from the Eocene Kishenehn Formation and remarks on the North American species of †*Amyzon* Cope, 1872. *J. Paleontol.* **90**, 288–304 (2016).
14. J. Leidy, Notice of remains of fishes in the Bridger Tertiary Formation of Wyoming. *Proceedings of the Academy of Natural Sciences of Philadelphia* **25**, 97–99 (1873).
15. L. Grande, J. G. Lundberg, Revision and redescription of the genus *Astephus* (Siluriformes: Ictaluridae) with a discussion of its phylogenetic relationships. *Journal of Vertebrate Paleontology* **8**, 139–171 (1988).

16. E. D. Cope, "On the extinct Vertebrata of the Eocene of Wyoming observed by the expedition of 1872, with notes on the geology" (U.S. Geological and Geographical Survey of the Territories, 1873).
17. J. G. Lundberg, "The evolutionary history of North American catfishes, family Ictaluridae," University of Michigan. (1970).
18. J. G. Lundberg, "The phylogeny of ictalurid catfishes: a synthesis of recent work" in *Systematics, Historical Ecology, and North American Freshwater Fishes*, R. L. Mayden, Ed. (Stanford University Press, 1992), pp. 392–420.
19. D. S. Jordan, A classification of fishes including families and generas as far as known. *Standford University Publications Biological Sciences* **3**, 79–243 (1923).
20. M. Arce-H., J. G. Lundberg, M. A. O'Leary, Phylogeny of the North American catfish family Ictaluridae (Teleostei: Siluriformes) combining morphology, genes and fossils. *Cladistics* **33**, 406–428 (2017).
21. F. J. Poyato-Ariza, "A revision of *Rubiesichthys gregalis* Wenz 1984" in *Systematics and Paleoecology*, (Verlag Dr. Friedrich Pfeil, 1996), pp. 361–368.
22. S. Wenz, *Rubiesichthys gregalis* ng, n. sp., Pisces Gonorhynchiformes, du Crétacé inférieur du Montsech (Province de Lérida, Espagne). *Bulletin du Muséum national d'histoire naturelle. Section C, Sciences de la terre, paléontologie, géologie, minéralogie* **6**, 275–285 (1984).
23. F. J. Poyato-Ariza, "The phylogenetic relationships of *Rubiesichthys gregalis* and *Gordichthys conquensis* (Ostariophysi, Chanidae), from the Early Cretaceous of Spain" in *Systematics and Paleoecology*, (Verlag Dr. Friedrich Pfeil, 1996), pp. 329–348.
24. G. Arratia, Basal teleosts and teleostean phylogeny. *Palaeo Ichthyologica* **7**, 5–168 (1997).
25. M. Benton, *et al.*, Constraints on the timescale of animal evolutionary history. *Palaeontologia Electronica* (2015). https://doi.org/10.26879/424.
26. C. Patterson, Comments on basal teleosts and teleostean phylogeny, by Gloria Arratia. *Copeia* **1998**, 1107 (1998).
27. A. Filleul, J. G. Maisey, Redescription of *Santanichthys diasii* (Otophysi, Characiformes) from the Albian of the Santana formation and comments on its implication for otophysan relationships. *American Museum Novitates* **3455**, 1–21 (2004).
28. M. C. Malabarba, L. R. Malabarba, "Biogeography of Characiformes: an evaluation of the available information of fossil and extant taxa" in *Origin and Phylogenetic Interrelationships of Teleosts*, (Verlag Dr. Friedrich Pfeil, 2010), pp. 317–336.
29. J. Alvarado-Ortega, O. Otero, D. Mayrinck, A new otophysan from the Tlayúa Quarry, Mexico: a North American clue to the early diversification of this group of freshwater fishes. *Papers in Palaeontology* **11**, e70056 (2025).
30. J. Liu, M.-M. Chang, M. V. H. Wilson, A. M. Murray, A new family of Cypriniformes (Teleostei, Ostariophysi) based on a redescription of † *Jianghanichthys hubeiensis* (Lei, 1977) from the Eocene Yangxi Formation of China. *Journal of Vertebrate Paleontology* **35**, e1004073 (2015).
31. H. Sudasinghe, *et al.*, Phylogenomics of Cypriniformes, the most diverse order of freshwater fishes: consensus, challenges and limitations. [Preprint] (2026). Available at: http://biorxiv.org/lookup/doi/10.64898/2026.01.14.699467 [Accessed 17 January 2026].
32. H. Sudasinghe, *et al.*, Reference genomes of four miniature and non-miniature cypriniform fishes inhabiting acidic peat-swamp forest blackwaters of Southeast Asia. [Preprint] (2026).
33. M. Kokot, M. Długosz, S. Deorowicz, KMC 3: counting and manipulating k-mer statistics. *Bioinformatics* **33**, 2759–2761 (2017).
34. A. Rhie, B. P. Walenz, S. Koren, A. M. Phillippy, Merqury: reference-free quality, completeness, and phasing assessment for genome assemblies. *Genome Biol* **21**, 245 (2020).
35. T. R. Ranallo-Benavidez, K. S. Jaron, M. C. Schatz, GenomeScope 2.0 and Smudgeplot for reference-free profiling of polyploid genomes. *Nat Commun* **11**, 1432 (2020).
36. K. M. Jenike, *et al.*, *k*-mer approaches for biodiversity genomics. *Genome Res.* **35**, 219–230 (2025).
37. T. R. Gregory, Animal genome size database. (2025). Available at: http://www.genomesize.com.

38. T. A. Elliott, T. R. Gregory, What's in a genome? The C-value enigma and the evolution of eukaryotic genome content. *Phil. Trans. R. Soc. B* **370**, 20140331 (2015).

Figures

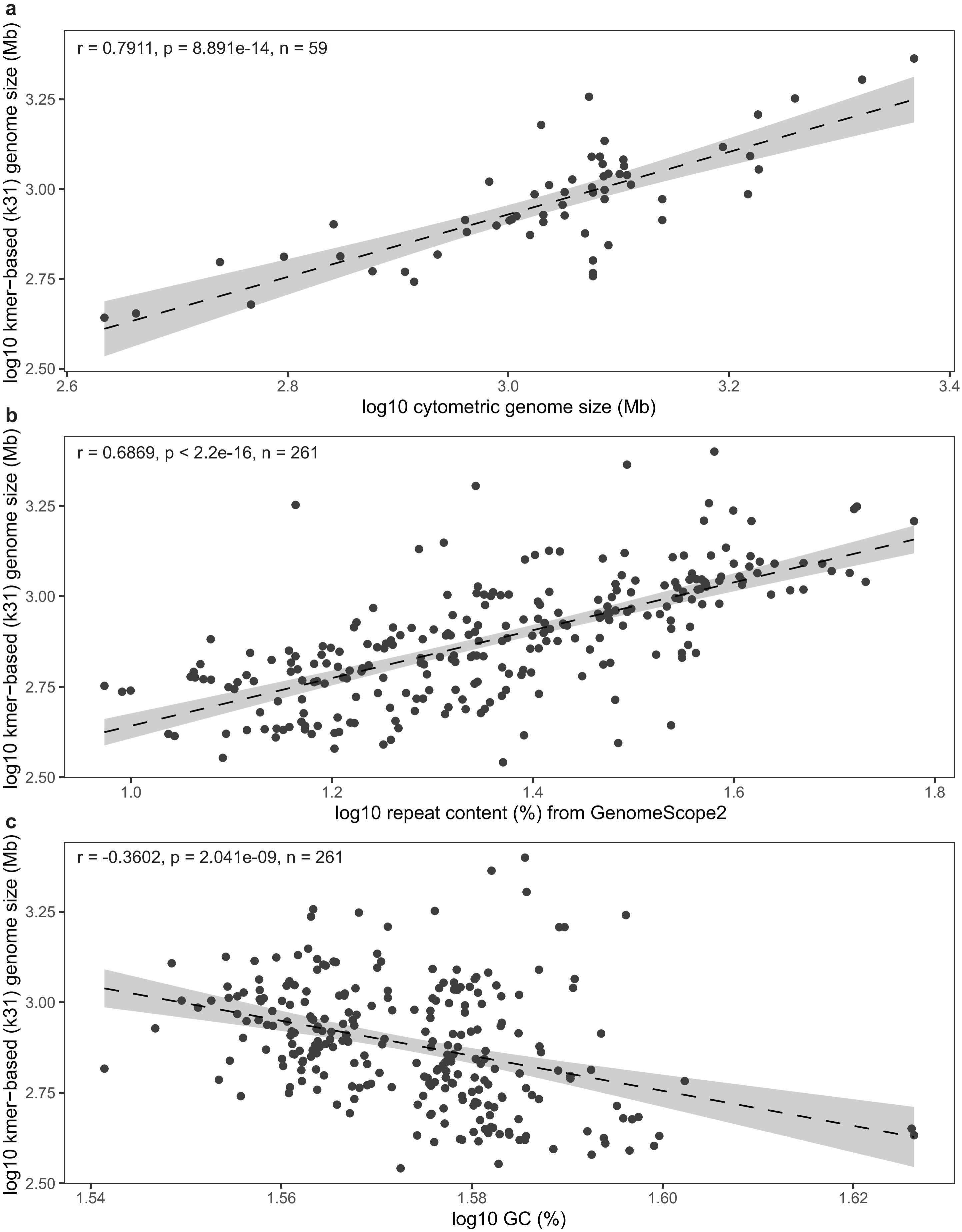

Fig. S1. Correlates of genome size variation across Cypriniformes based on k = 31 estimates. **a)** Correlation between cytometric genome size estimates compiled from the Animal Genome Size Database and k-mer-based genome size estimates from the present study, with each data point representing a genus-level mean. **b)** Correlation between repeat content estimated from GenomeScope 2.0 k-mer profiling and haploid genome size. **c)** Correlation between genomic GC content of the genome assembly and haploid genome size. Pearson correlation coefficients and associated p values are indicated in each panel.

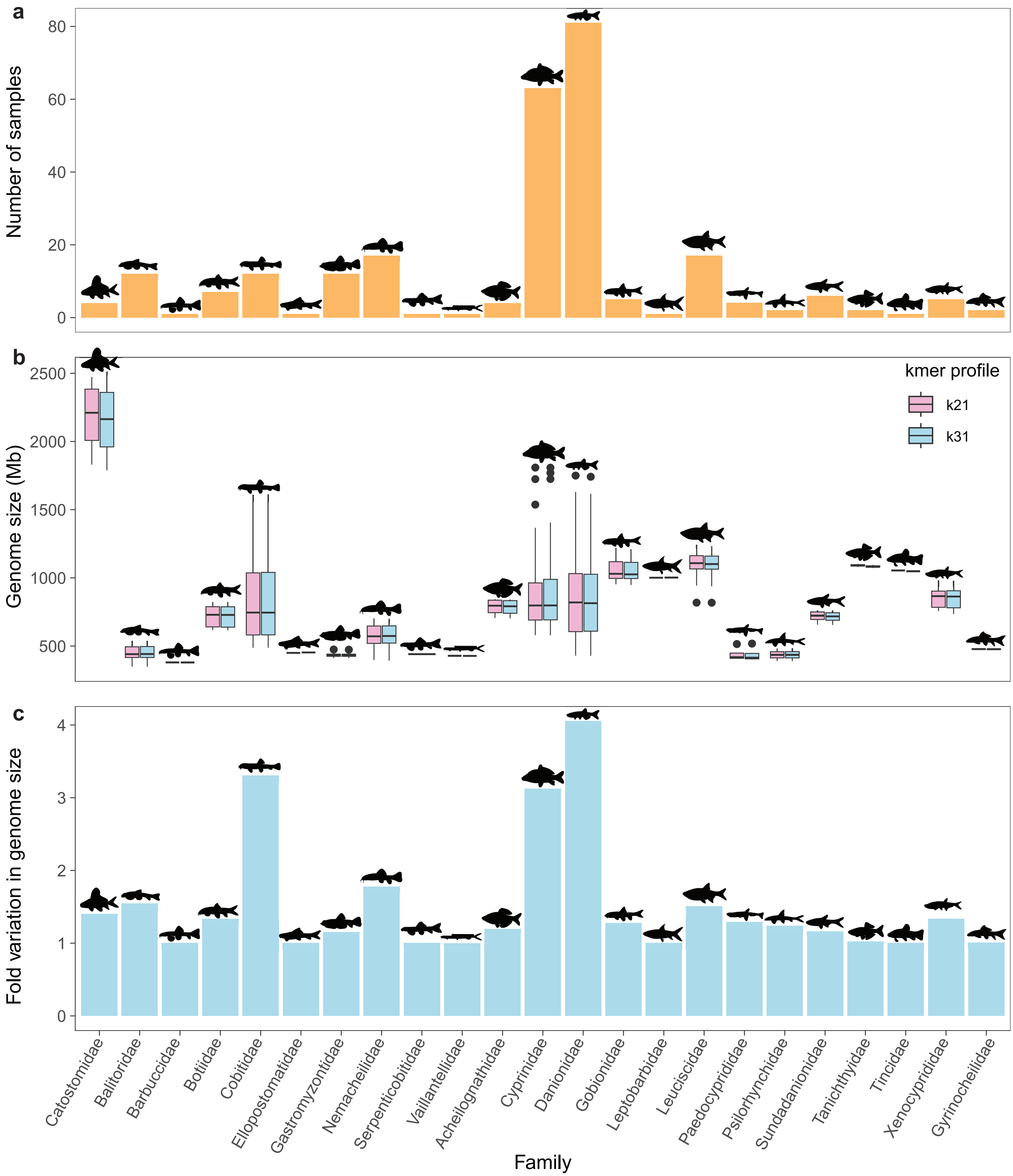

Fig. S2. K-mer-based genome size estimation across Cypriniformes for 261 samples. **a)** Number of species sampled per family included in the k-mer profiling dataset. **b)** Comparison of haploid genome size estimates derived from k = 21 and k = 31 across cypriniform families. **c)** Intrafamilial fold variation in genome size (based on k = 31 estimates) across cypriniform families.

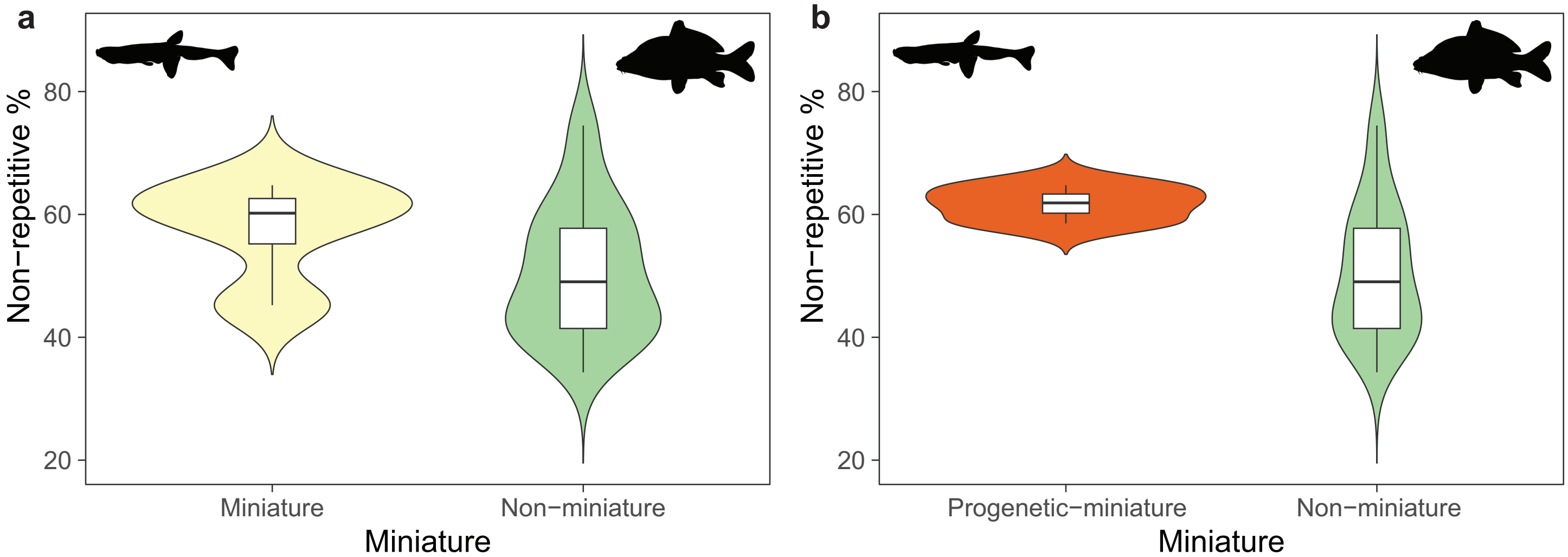

Fig. S3. Distribution of the percentage of non-repetitive DNA across **a)** miniature and non-miniature and **b)** progenetic miniature and non-miniature cypriniform species included in the comparative genomic analyses of transposable element content.

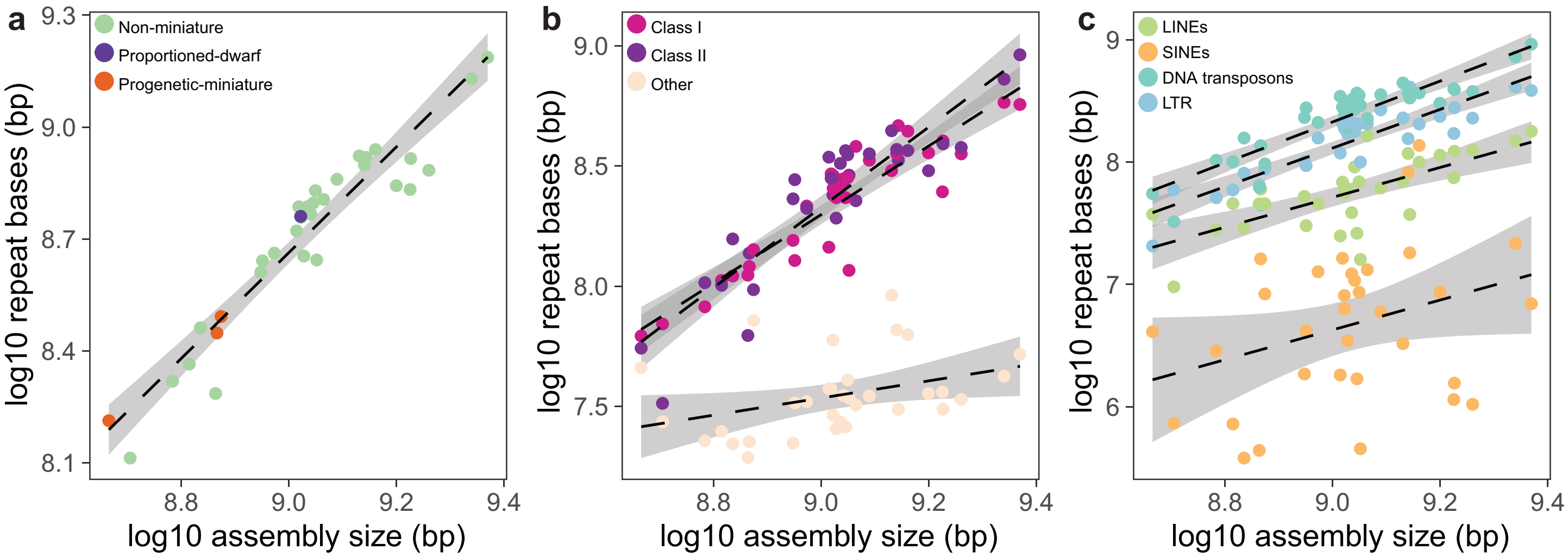

Fig. S4. Relationship between log-transformed genome assembly size and log-transformed repeat content across the 33-species comparative genomic dataset. **a)** Data points colored by miniaturization category (non-miniature, proportioned dwarf, and progenetic miniature). **b)** Data points colored by major repeat class (Class I, Class II, and other repeats). **c)** Data points colored by repeat subclass (LINEs, SINEs, DNA transposons, and LTR elements).

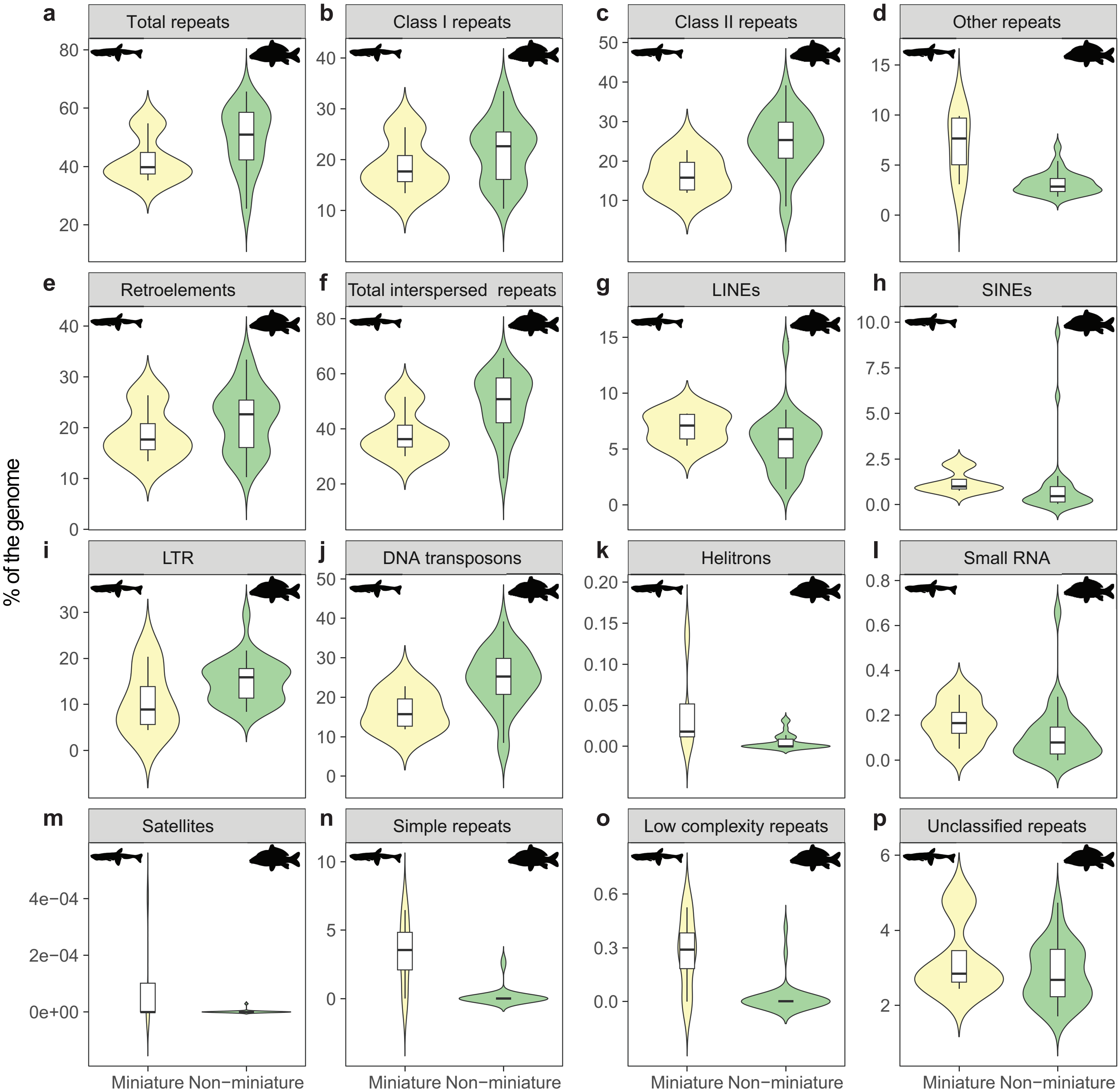

Fig. S5. Comparison of repeat content as a percentage of the genome between miniature (n = 4) and non-miniature (n = 29) cypriniform species included in the comparative genomic analyses of transposable element content. Panels show **a)** total repeats; **b)** Class I repeats; **c)** Class II repeats; **d)** other repeats (small RNAs, satellites, simple repeats, low-complexity regions, and unclassified elements); **e)** retroelements; **f)** total interspersed repeats; **g)** LINEs; **h)** SINEs; **i)** LTR elements; **j)** DNA transposons; **k)** helitrons; **l)** small RNAs; **m)** satellites; **n)** simple repeats; **o)** low-complexity repeats; and **p)** unclassified repeats.

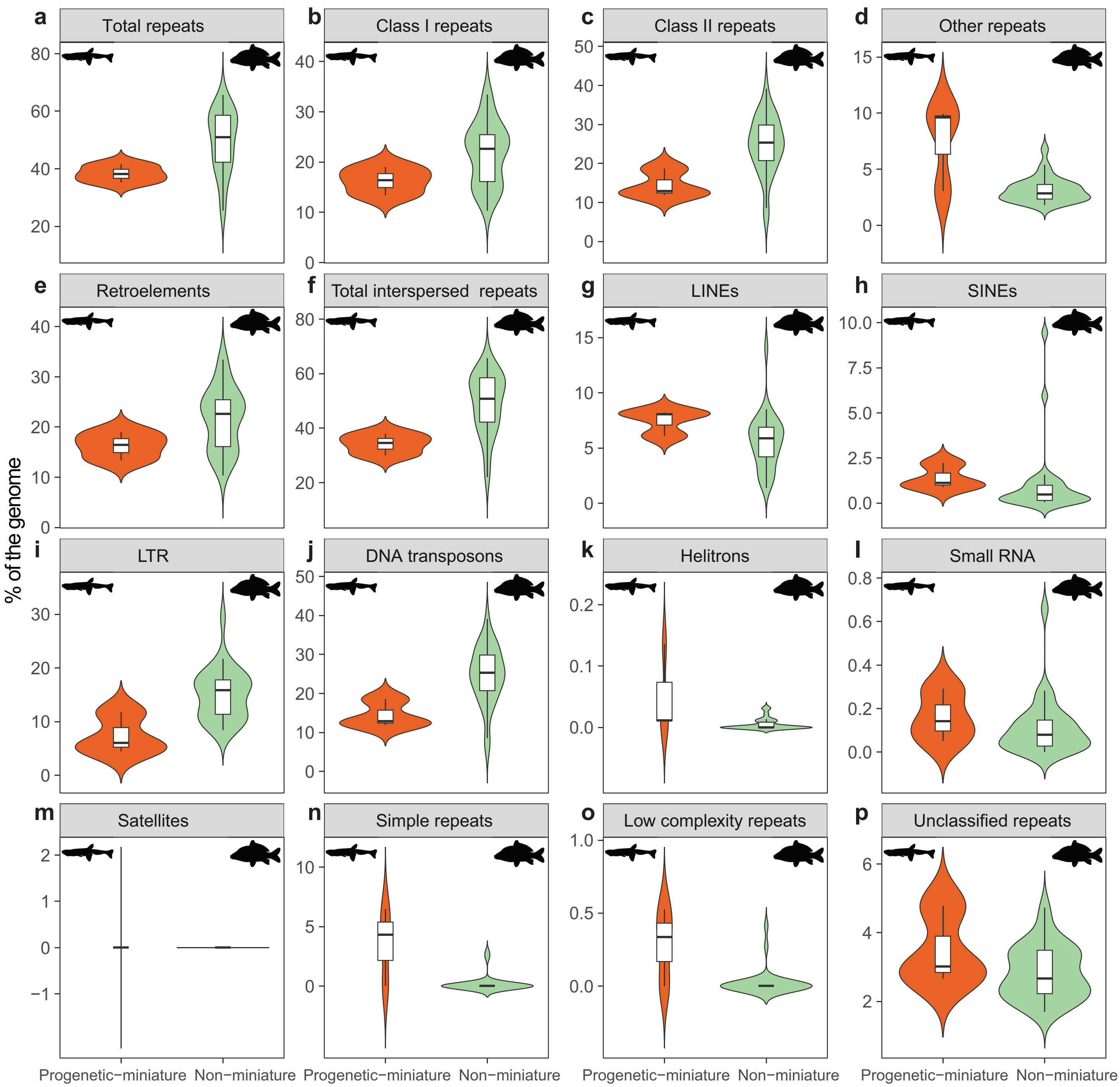

Fig. S6. Comparison of repeat content as a percentage of the genome between progenetic-miniature (n = 3) and non-miniature (n = 29) cypriniform species included in the comparative genomic analyses of transposable element content. Panels show **a)** total repeats; **b)** Class I repeats; **c)** Class II repeats; **d)** other repeats (small RNAs, satellites, simple repeats, low-complexity regions, and unclassified elements); **e)** retroelements; **f)** total interspersed repeats; **g)** LINEs; **h)** SINEs; **i)** LTR elements; **j)** DNA transposons; **k)** helitrons; **l)** small RNAs; **m)** satellites; **n)** simple repeats; **o)** low-complexity repeats; and **p)** unclassified repeats.

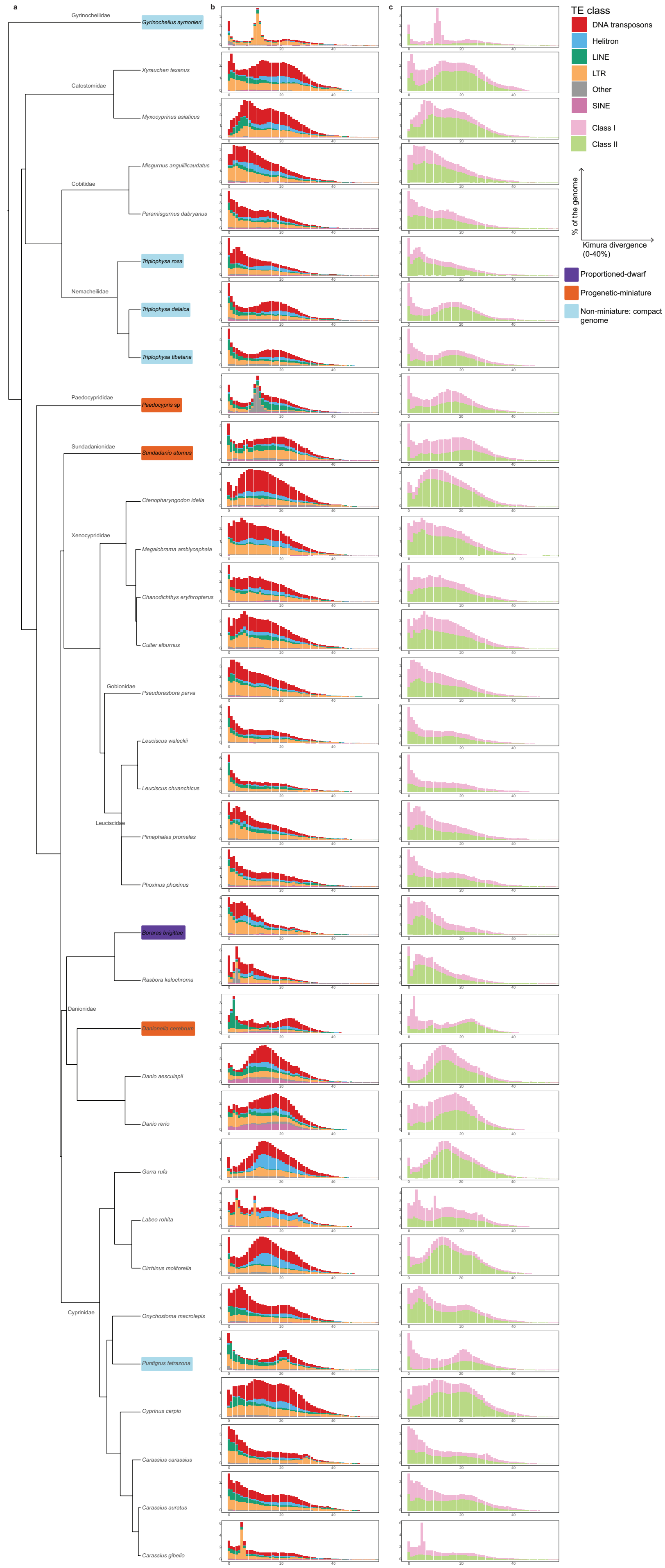

Fig. S7. Transposable element (TE) divergence landscapes for the 33-species comparative genomic dataset. **a)** Phylogenetic relationships among the 33 taxa included in the comparative genomic analyses. **b)** Repeat landscapes showing the genomic proportion occupied by major TE subclasses and other repeat categories (small RNAs, satellites, simple repeats, low-complexity regions, and unclassified elements) for each species. **c)** Repeat landscapes showing the genomic proportion occupied by Class I (LTR elements, LINEs, SINEs) and Class II (DNA transposons, helitrons) transposable elements for each species. In panels b and c, the x-axis represents CpG-adjusted Kimura two-parameter (K2P) divergence from TE consensus sequences, where low divergence values (left) correspond to recently active insertions and high divergence values (right) reflect older, inactive transposition events.

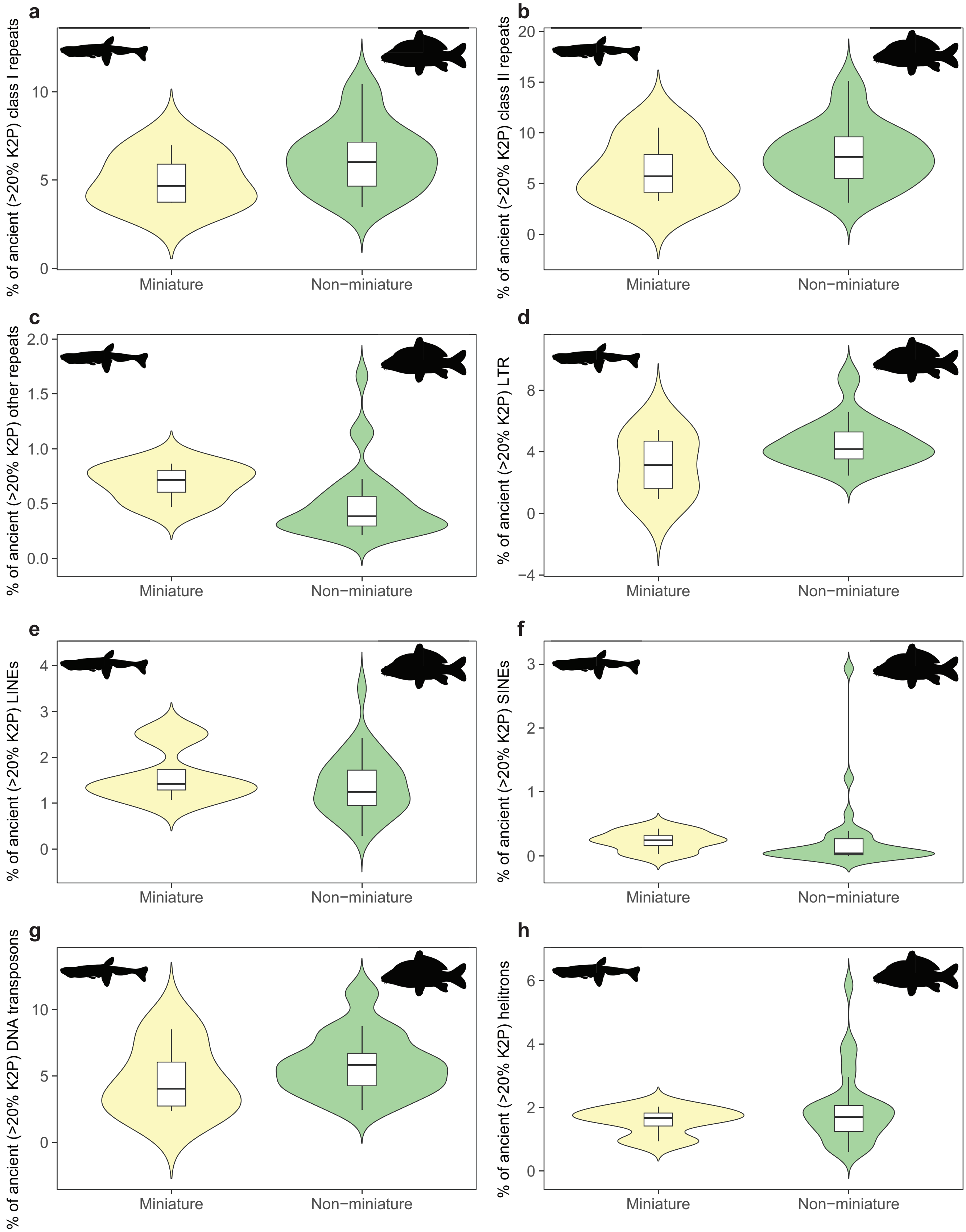

Fig. S8. Comparison of ancient transposable element (TE) insertions (CpG-adjusted Kimura two-parameter divergence > 20% from TE consensus sequences) between miniature (n = 4) and non-miniature (n = 29) cypriniform species included in the comparative genomic analyses of TE content. Panels show **a)** Class I repeats; **b)** Class II repeats; **c)** other repeats (small RNAs, satellites, simple repeats, low-complexity regions, and unclassified elements); **d)** LTR elements; **e)** LINEs; **f)** SINEs; **g)** DNA transposons; and **h)** helitrons.

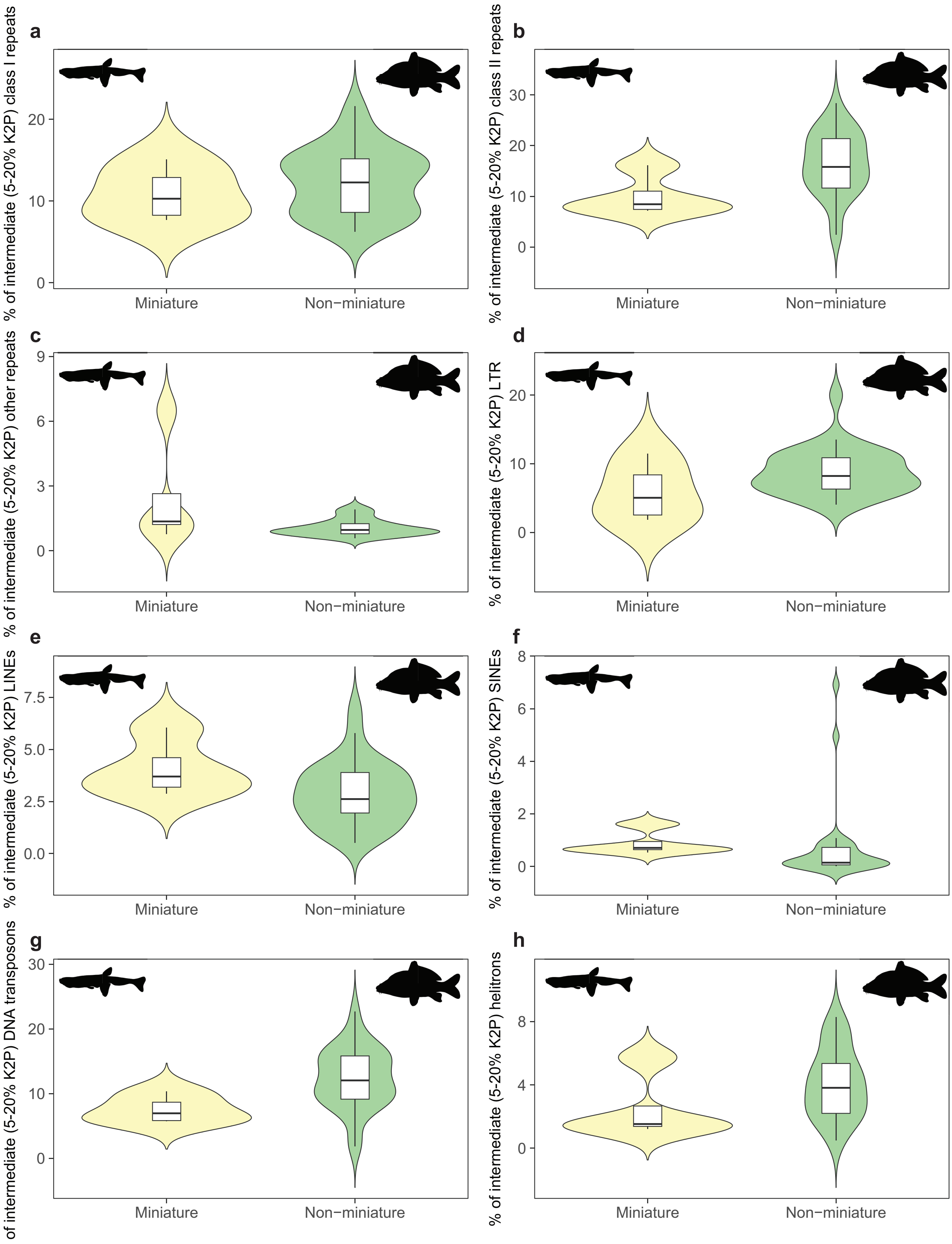

Fig. S9. Comparison of intermediate transposable element (TE) insertions (CpG-adjusted Kimura two-parameter divergence 5-20% from TE consensus sequences) between miniature (n = 4) and non-miniature (n = 29) cypriniform species included in the comparative genomic analyses of TE content. Panels show **a)** Class I repeats; **b)** Class II repeats; **c)** other repeats (small RNAs, satellites, simple repeats, low-complexity regions, and unclassified elements); **d)** LTR elements; **e)** LINEs; **f)** SINEs; **g)** DNA transposons; and **h)** helitrons.

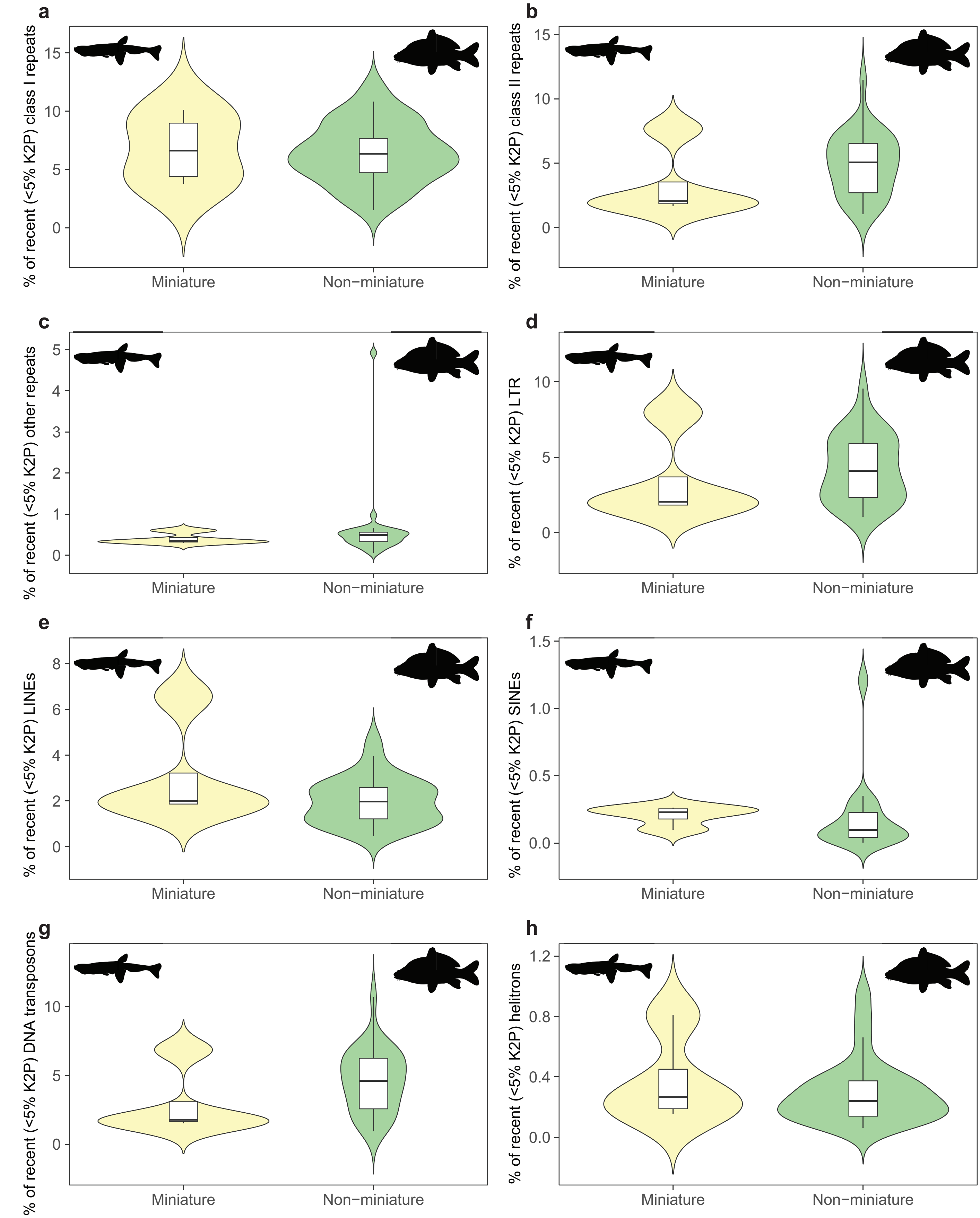

Fig. S10. Comparison of recent transposable element (TE) insertions (CpG-adjusted Kimura two-parameter divergence < 5% from TE consensus sequences) between miniature (n = 4) and non-miniature (n = 29) cypriniform species included in the comparative genomic analyses of TE content. Panels show **a)** Class I repeats; **b)** Class II repeats; **c)** other repeats (small RNAs, satellites, simple repeats, low-complexity regions, and unclassified elements); **d)** LTR elements; **e)** LINEs; **f)** SINEs; **g)** DNA transposons; and **h)** helitrons.

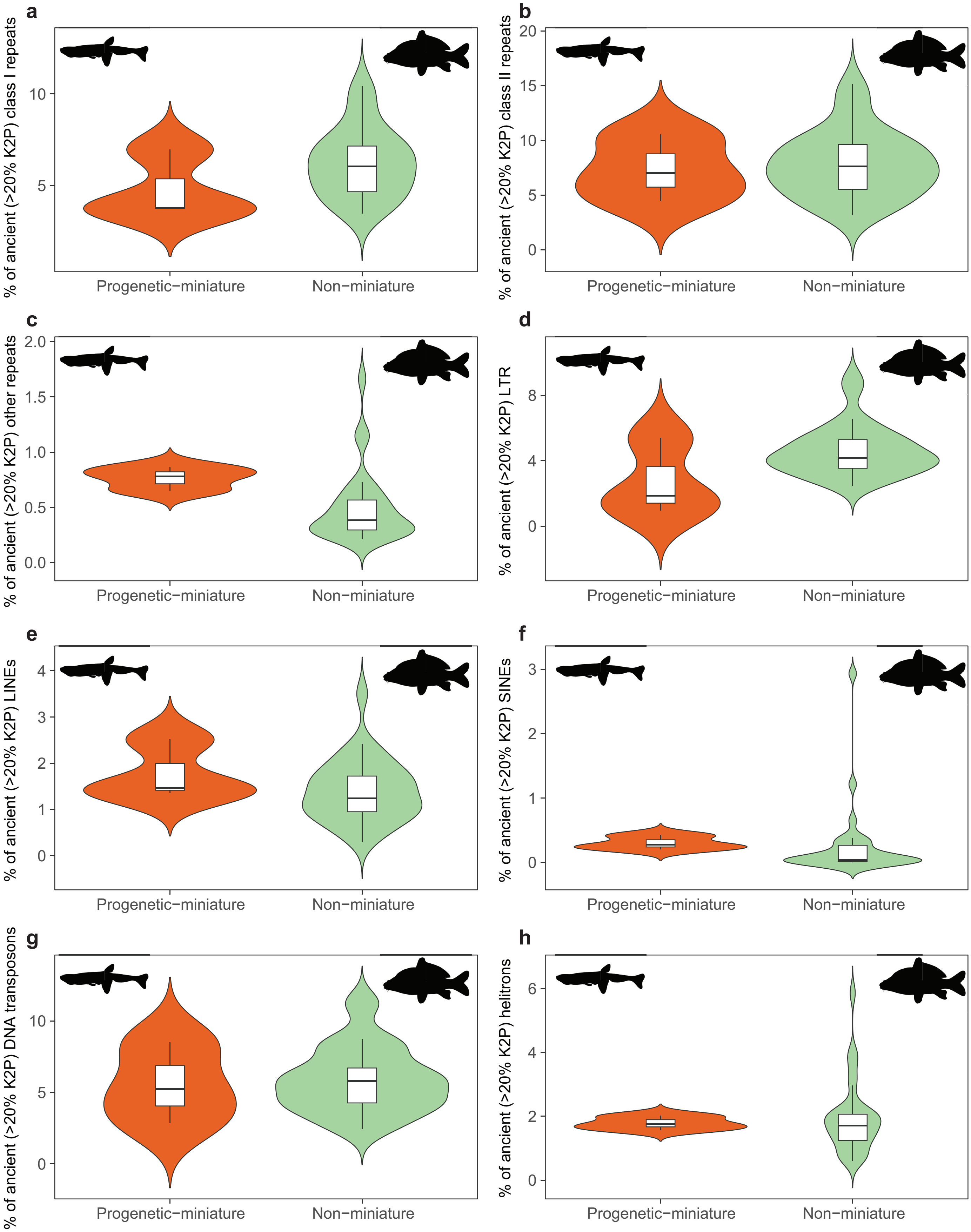

Fig. S11. Comparison of ancient ransposable element (TE) insertions (CpG-adjusted Kimura two-parameter divergence > 20% from TE consensus sequences) between progenetic-miniature (n = 3) and non-miniature (n = 29) cypriniform species included in the comparative genomic analyses of TE content. Panels show **a)** Class I repeats; **b)** Class II repeats; **c)** other repeats (small RNAs, satellites, simple repeats, low-complexity regions, and unclassified elements); **d)** LTR elements; **e)** LINEs; **f)** SINEs; **g)** DNA transposons; and **h)** helitrons.

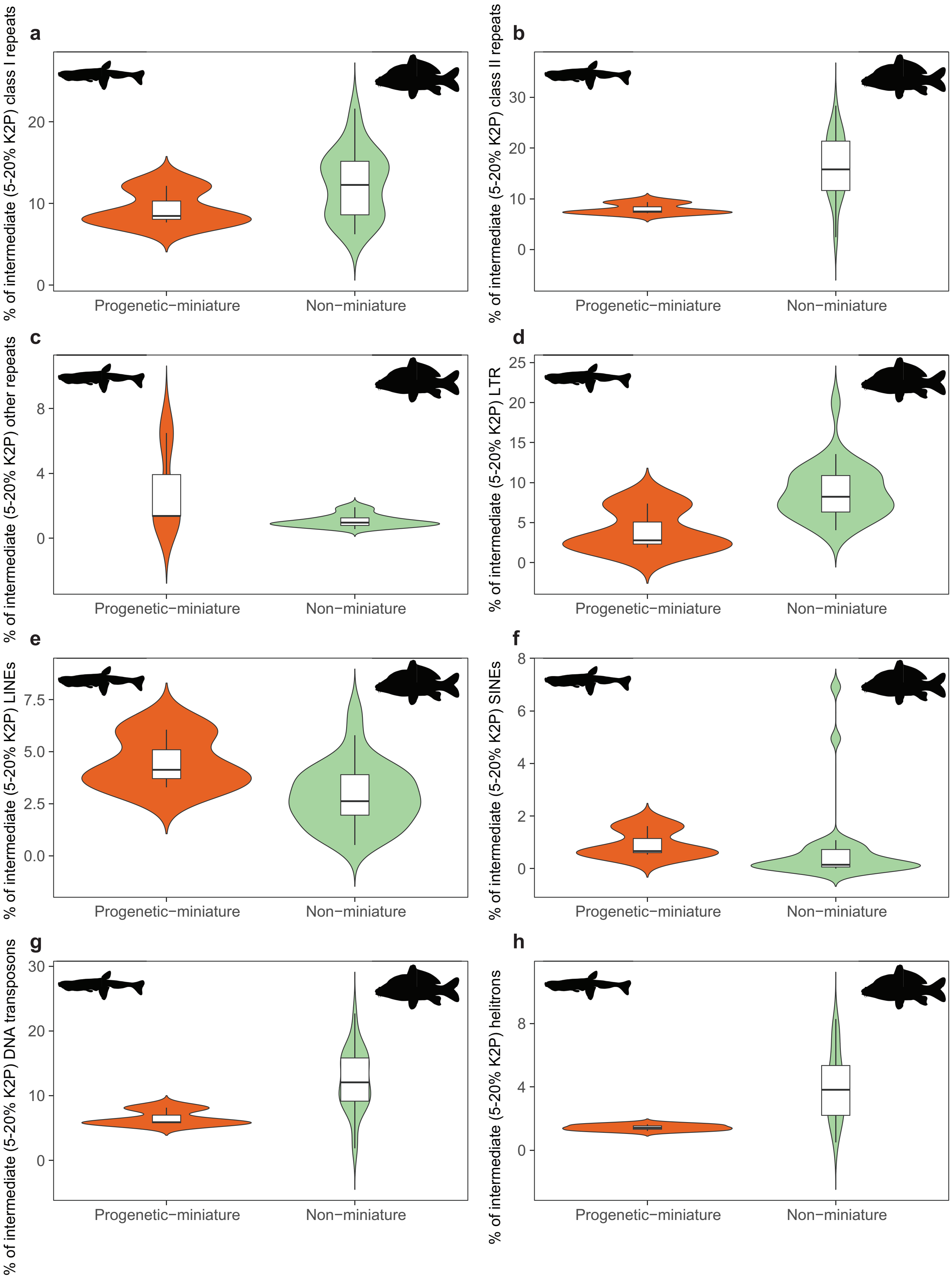

Fig. S12. Comparison of intermediate ransposable element (TE) insertions (CpG-adjusted Kimura two-parameter divergence 5-20% from TE consensus sequences) between progenetic-miniature (n = 3) and non-miniature (n = 29) cypriniform species included in the comparative genomic analyses of TE content. Panels show **a)** Class I repeats; **b)** Class II repeats; **c)** other repeats (small RNAs, satellites, simple repeats, low-complexity regions, and unclassified elements); **d)** LTR elements; **e)** LINEs; **f)** SINEs; **g)** DNA transposons; and **h)** helitrons.

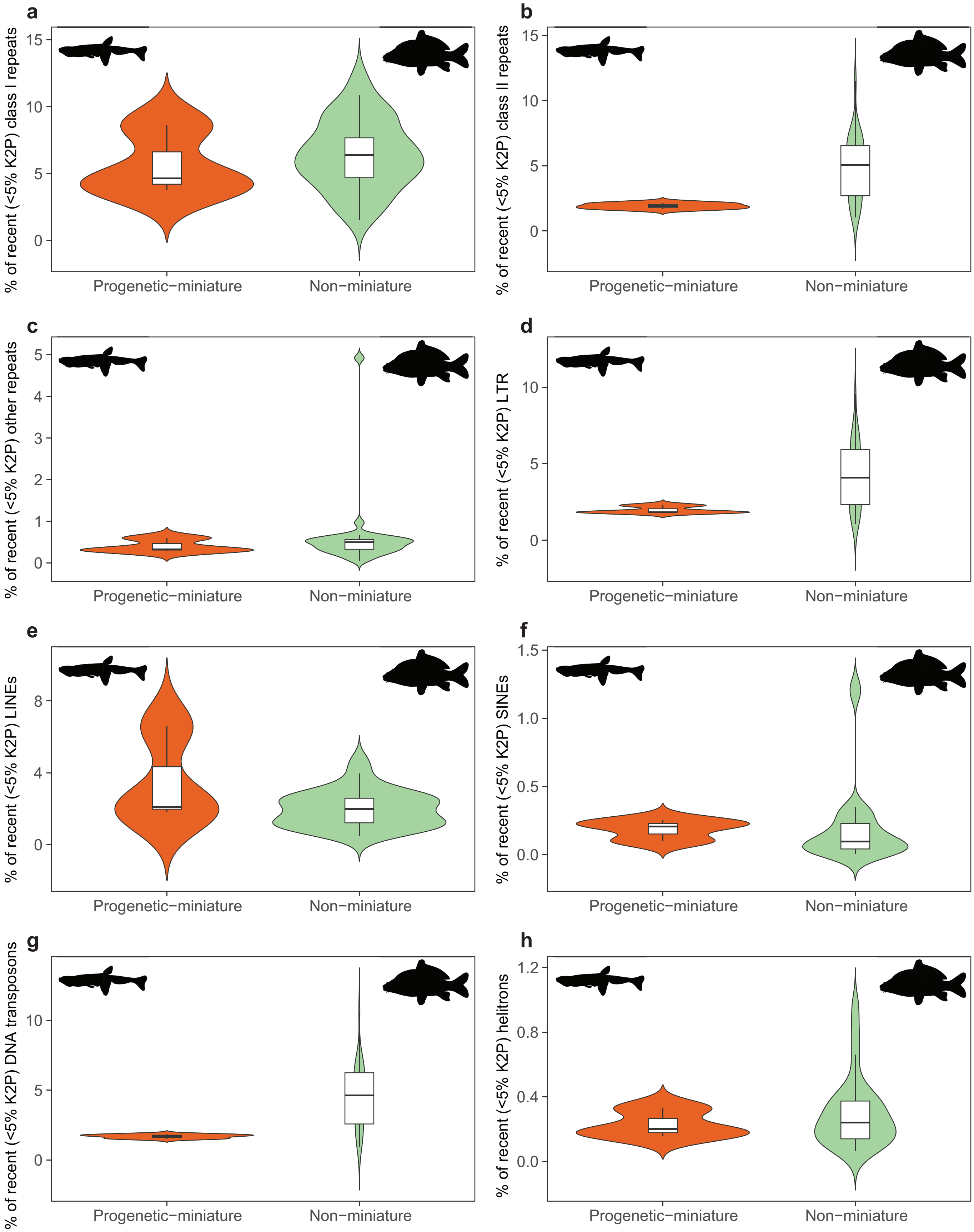

Fig. S13. Comparison of recent ransposable element (TE) insertions (CpG-adjusted Kimura two-parameter divergence < 5% from TE consensus sequences) between progenetic-miniature (n = 3) and non-miniature (n = 29) cypriniform species included in the comparative genomic analyses of TE content. Panels show **a)** Class I repeats; **b)** Class II repeats; **c)** other repeats (small RNAs, satellites, simple repeats, low-complexity regions, and unclassified elements); **d)** LTR elements; **e)** LINEs; **f)** SINEs; **g)** DNA transposons; and **h)** helitrons.

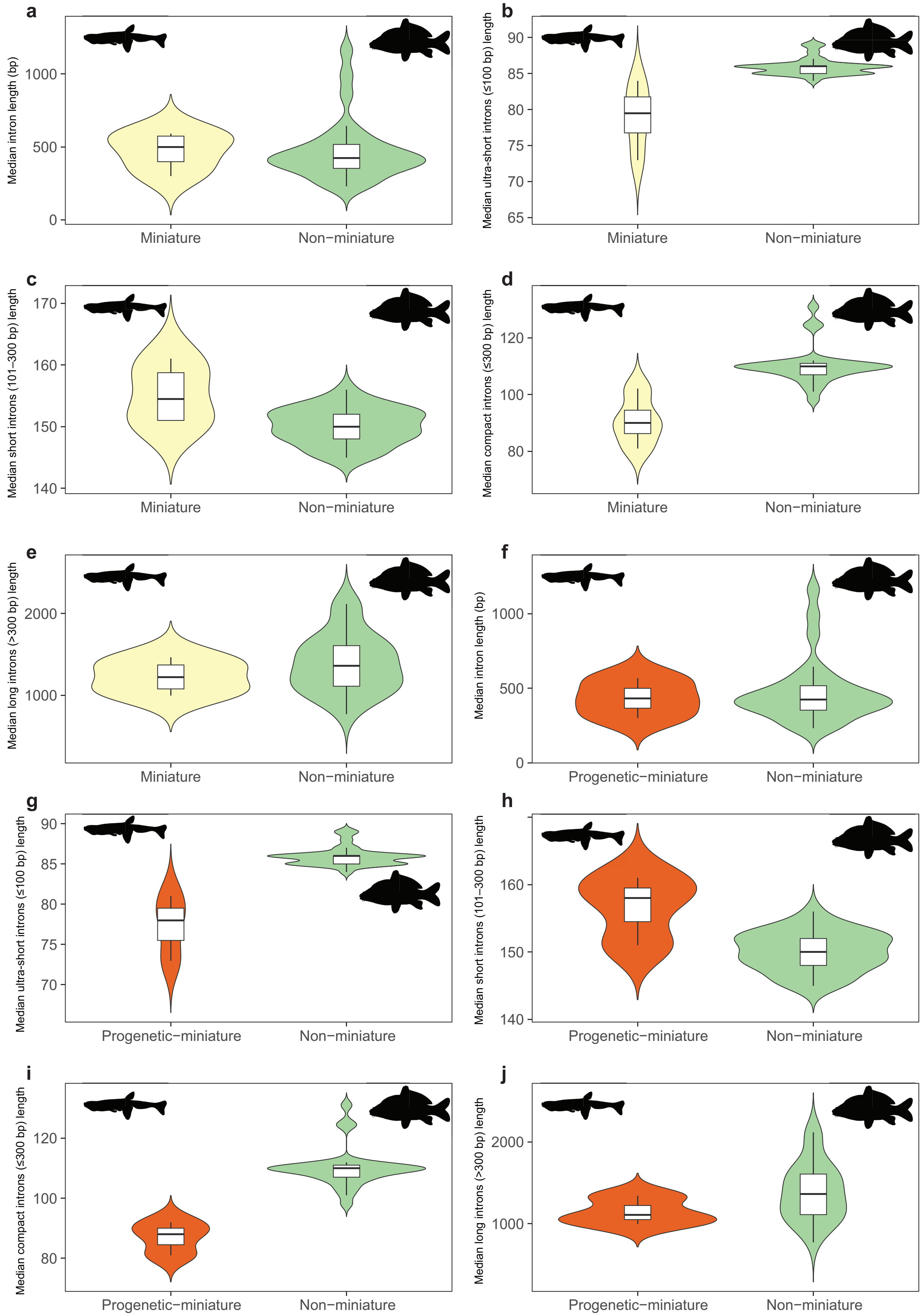

Fig. S14. Comparison of median intron lengths by size category between **a–e)** miniature (n = 4) and non-miniature (n = 29) and **f–j)** progenetic miniature (n = 3) and non-miniature (n = 29) cypriniform species in the 33-species comparative genomic dataset. Panels show **a, f)** overall median intron length; **b, g)** median ultra-short intron length (≤100 bp); **c, h)** median short intron length (101–300 bp); **d, i)** median compact intron length (≤300 bp); and **e, j)** median long intron length (>300 bp).

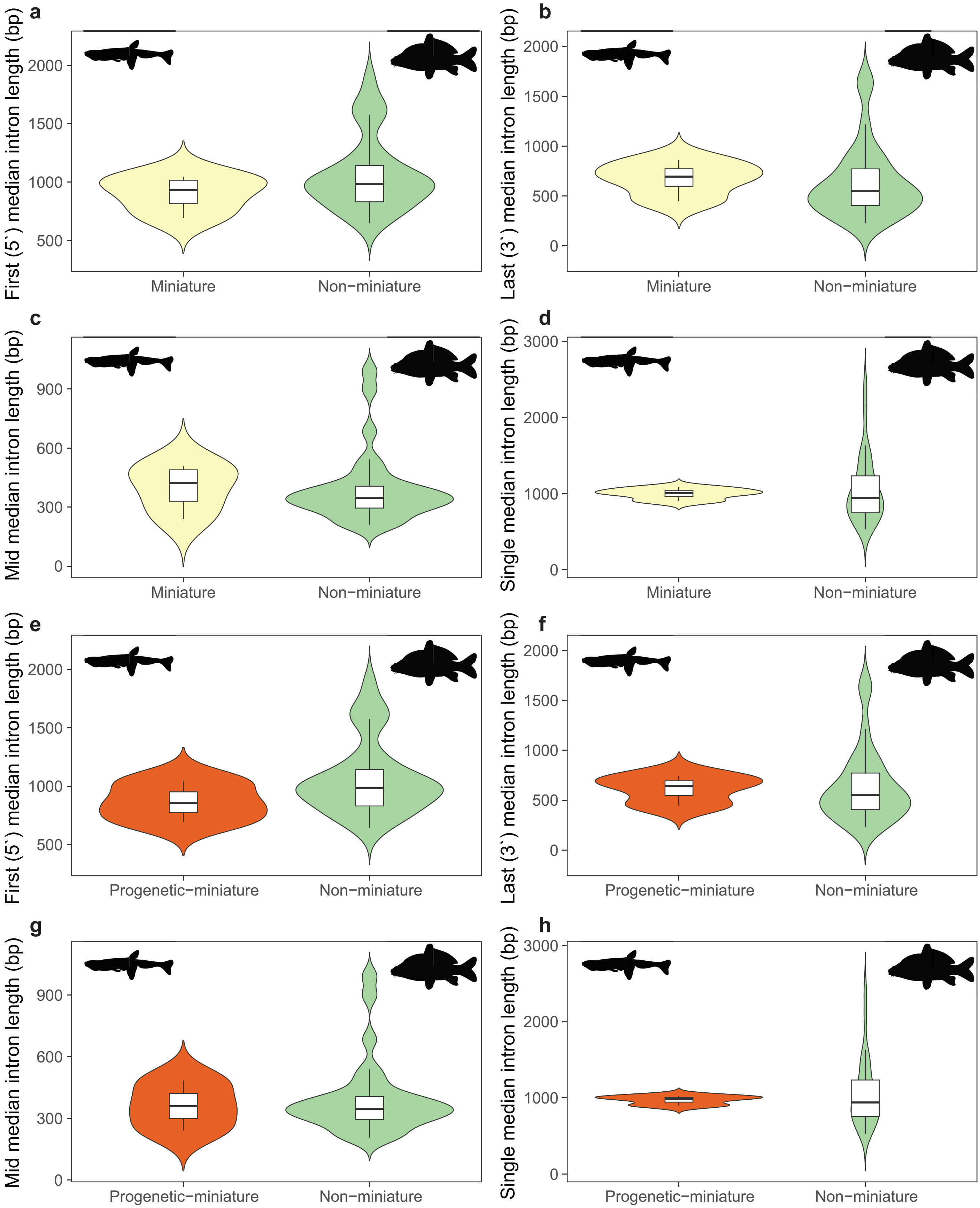

Fig. S15. Comparison of median intron lengths by positional category between **a–d)** miniature (n = 4) and non-miniature (n = 29) and **e–h)** progenetic miniature (n = 3) and non-miniature (n = 29) cypriniform species in the 33-species comparative genomic dataset. Panels show **a, e)** first (5′) intron length; **b, f)** last (3′) intron length; **c, g)** middle intron length; and **d, h)** single intron length.

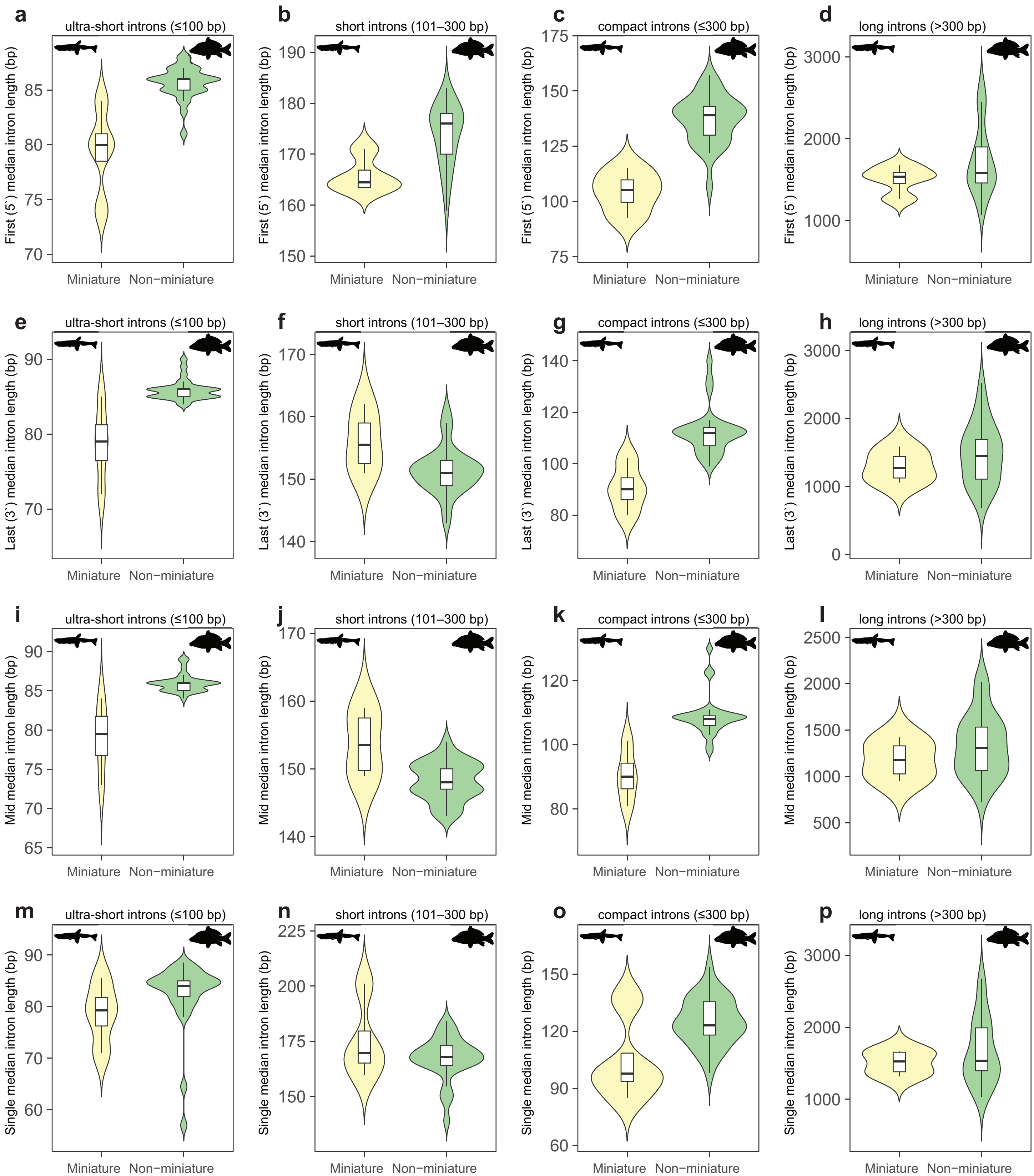

Fig. S16. Comparison of median intron lengths by combined size class and positional category between miniature (n = 4) and non-miniature (n = 29) cypriniform species in the 33-species comparative genomic dataset. First (5′) intron position for **a)** ultra-short (≤100 bp), **b)** short (101–300 bp), **c)** compact (≤300 bp), and **d)** long (>300 bp) introns. Last (3′) intron position for **e)** ultra-short, **f)** short, **g)** compact, and **h)** long introns. Middle intron position for **i)** ultra-short, **j)** short, **k)** compact, and **l)** long introns. Single introns for **m)** ultra-short, **n)** short, **o)** compact, and **p)** long introns.

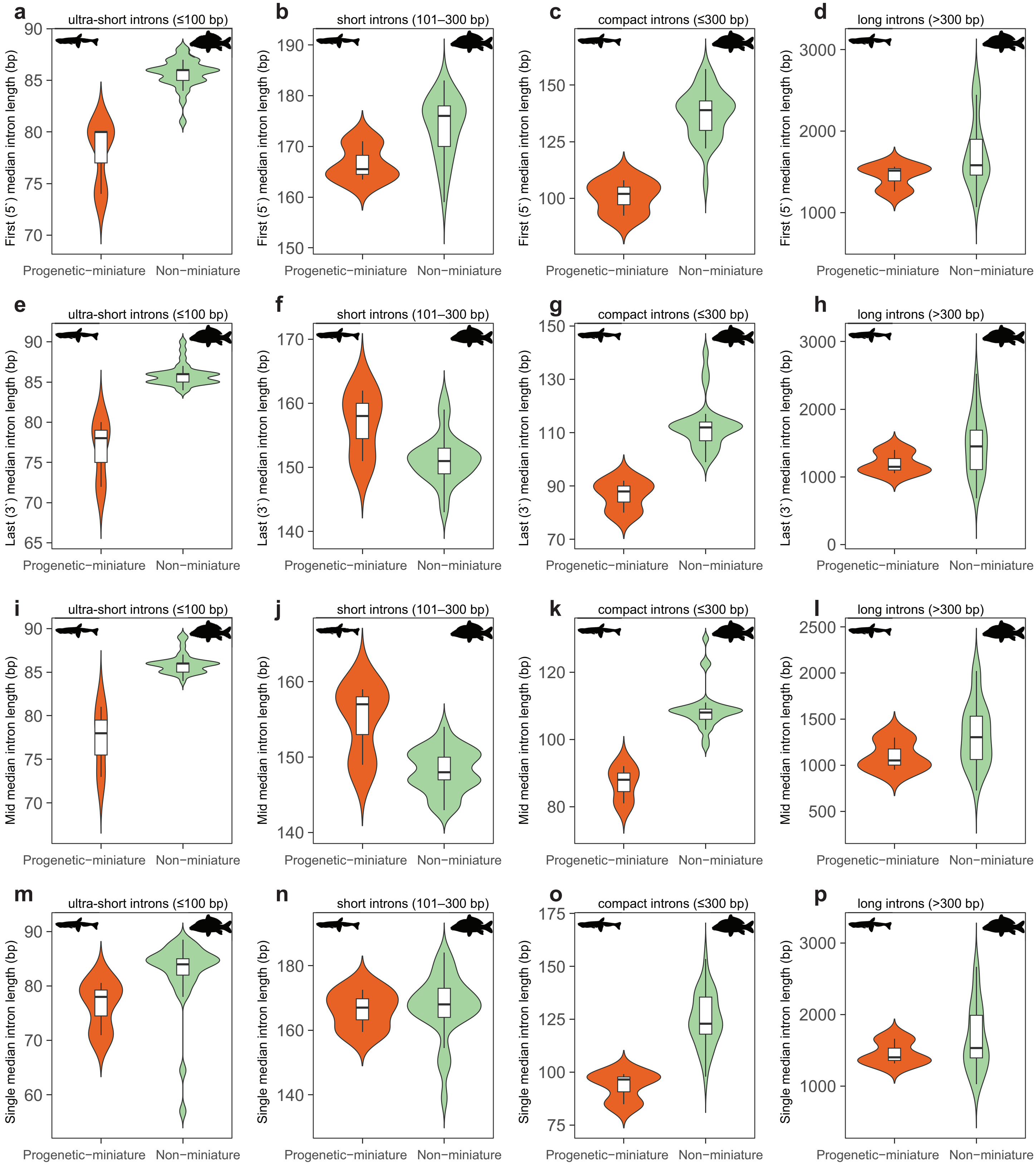

Fig. S17. Comparison of median intron lengths by combined size class and positional category between progenetic miniature (n = 3) and non-miniature (n = 29) cypriniform species in the 33-species comparative genomic dataset. First (5′) intron position for **a)** ultra-short (≤100 bp), **b)** short (101–300 bp), **c)** compact (≤300 bp), and **d)** long (>300 bp) introns. Last (3′) intron position for **e)** ultra-short, **f)** short, **g)** compact, and **h)** long introns. Middle intron position for **i)** ultra-short, **j)** short, **k)** compact, and **l)** long introns. Single introns for **m)** ultra-short, **n)** short, **o)** compact, and **p)** long introns.

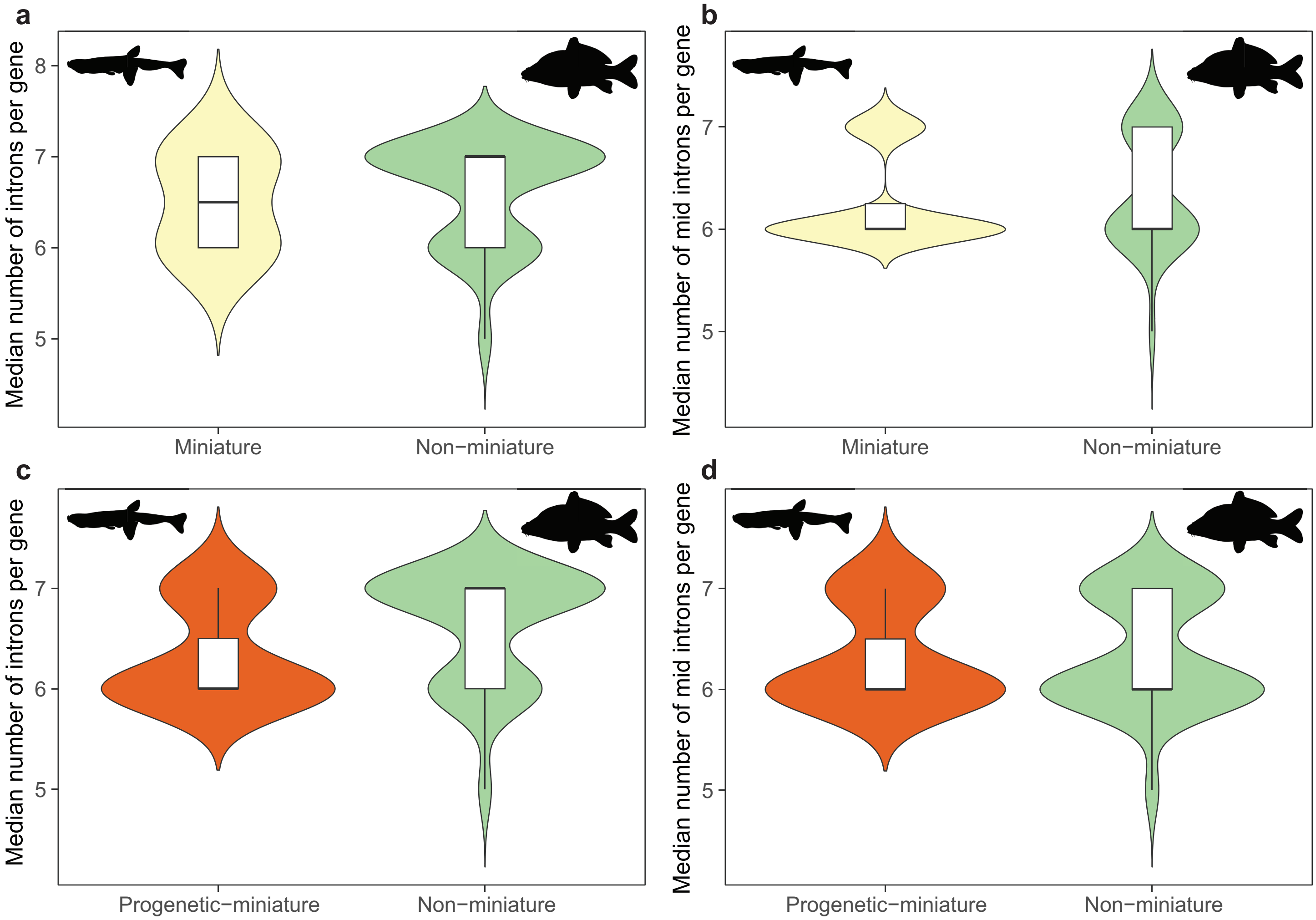

Fig. S18. Median number of introns per gene in the cypriniform species in the 33-species comparative genomic dataset in **a)** miniature (n = 4) and non-miniature (n = 29) and **c)** progenetic miniature (n = 3) and non-miniature (n = 29) species. Median number of middle introns per gene in **b)** miniature (n = 4) and non-miniature (n = 29) and **d)** progenetic miniature (n = 3) and non-miniature (n = 29) species.

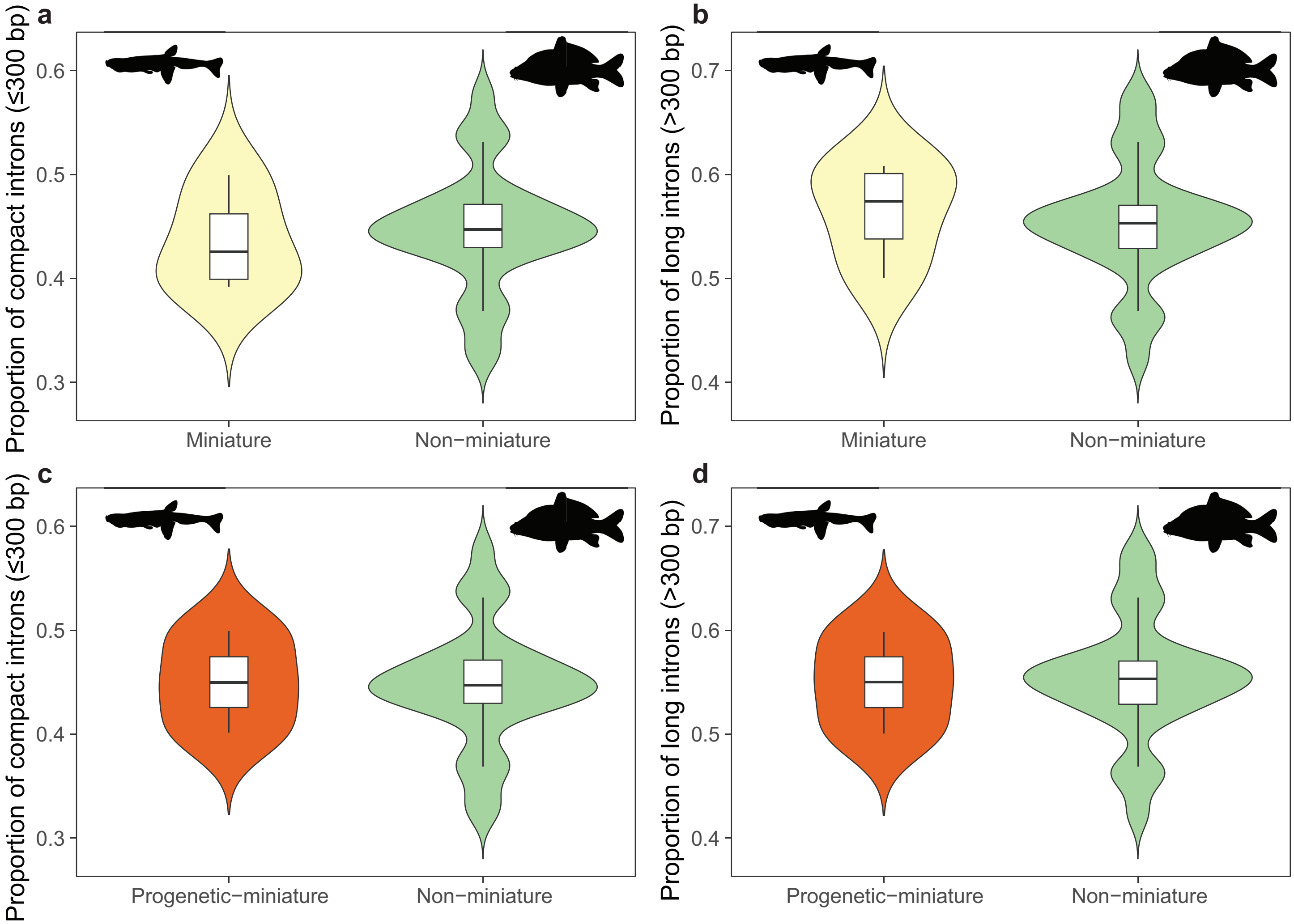

Fig. S19. Proportion of compact (≤300 bp) and long (>300 bp) introns in the 33-species comparative genomic dataset. Proportion of compact introns in **a)** miniature (n = 4) and non-miniature (n = 29) and **c)** progenetic miniature (n = 3) and non-miniature (n = 29) species. Proportion of long introns in **b)** miniature (n = 4) and non-miniature (n = 29) and **d)** progenetic miniature (n = 3) and non-miniature (n = 29) species.

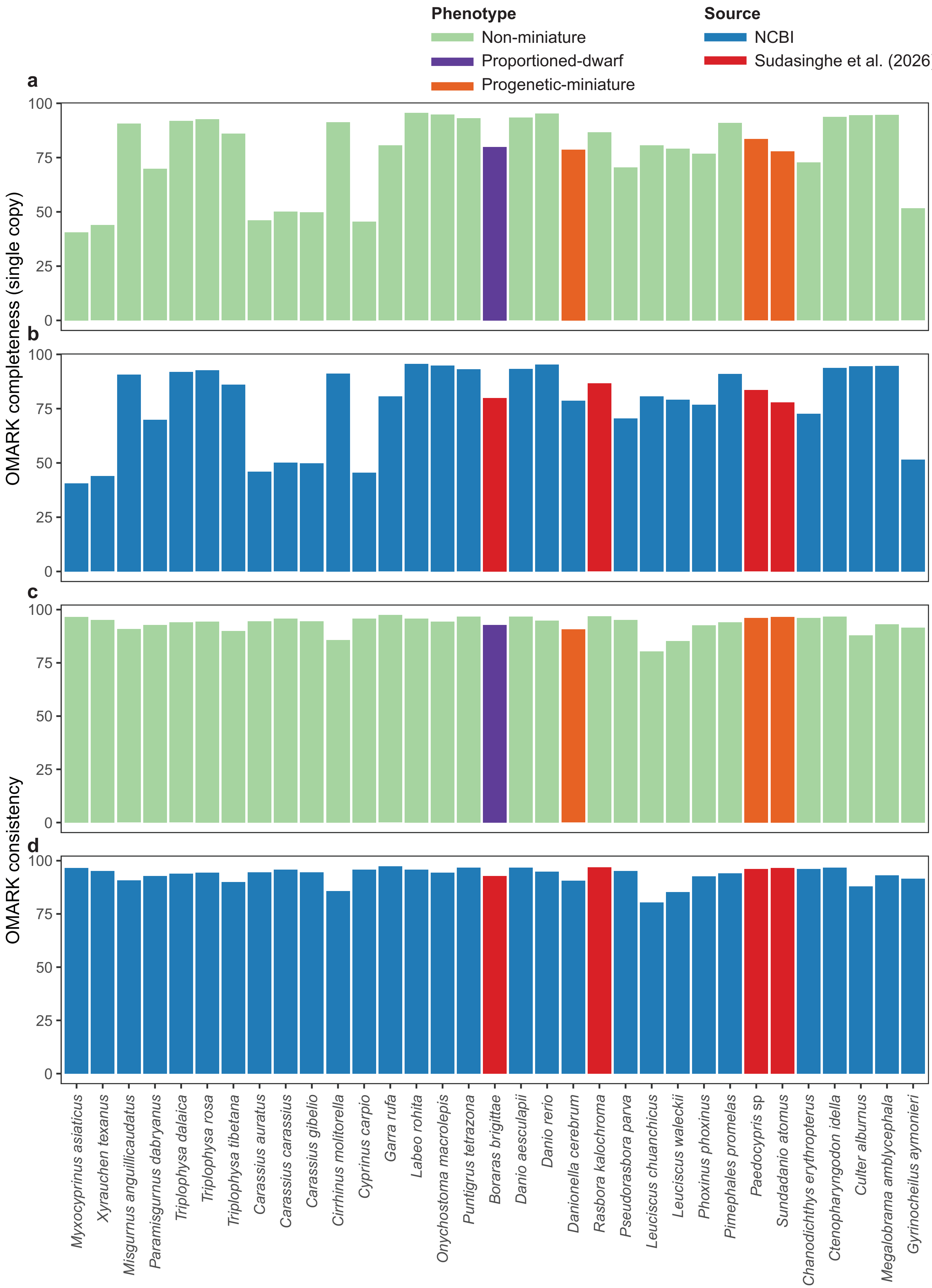

Fig. S20. Assessment of proteome quality and annotation consistency using OMArk for the 33-species dataset used in comparative genomic analyses of gene architecture. Single-copy completeness scores grouped by **a)** miniaturization category and **b)** annotation source. OMArk consistency scores grouped by **c)** miniaturization category and **d)** annotation source.

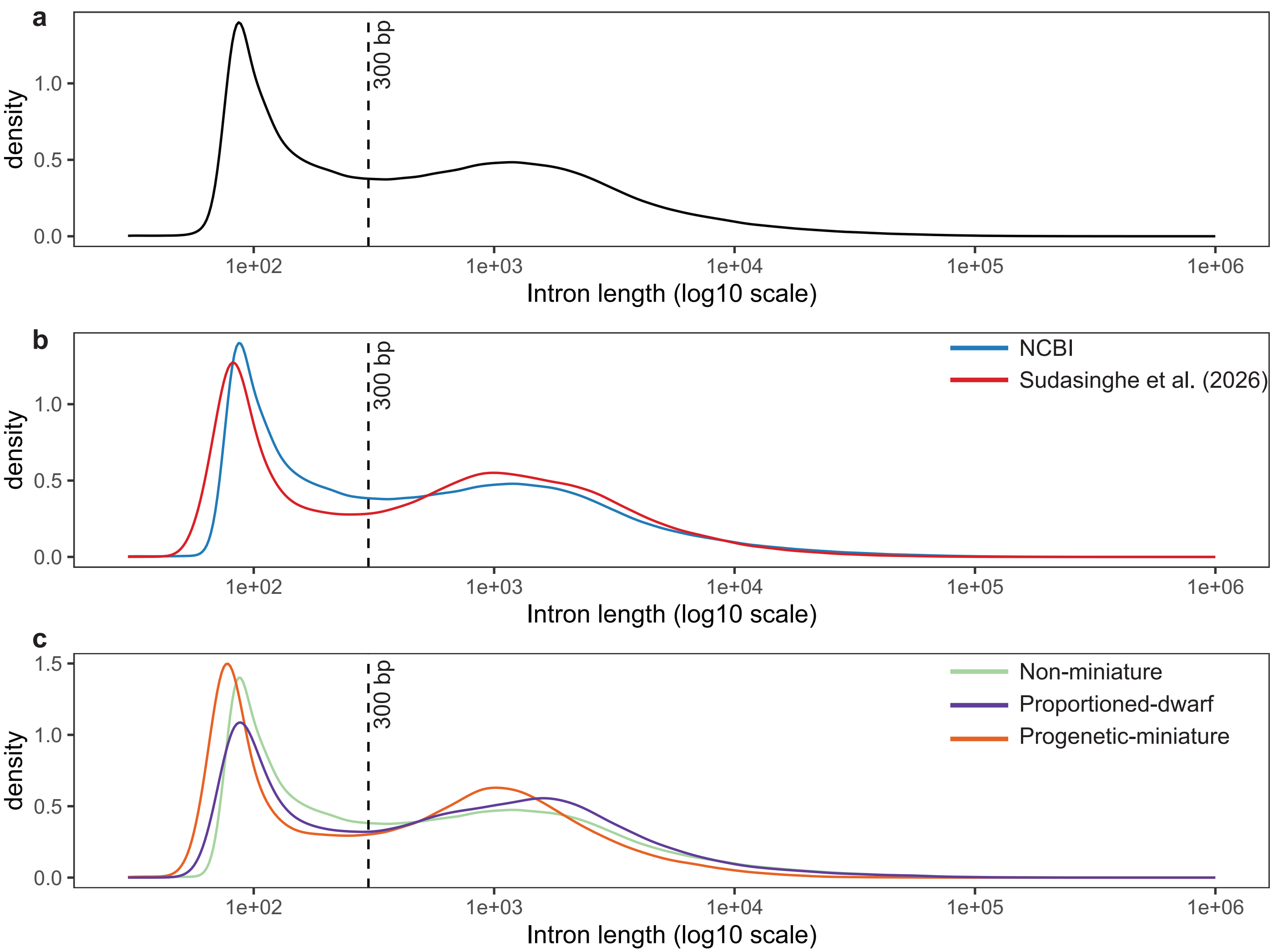

Fig. S21. Intron length distributions across the 33-species dataset used in comparative genomic analyses of gene architecture, illustrating the bimodal distribution. **a)** Overall distribution across all species. **b)** Distributions grouped by annotation source. **c)** Distributions grouped by miniaturization category.

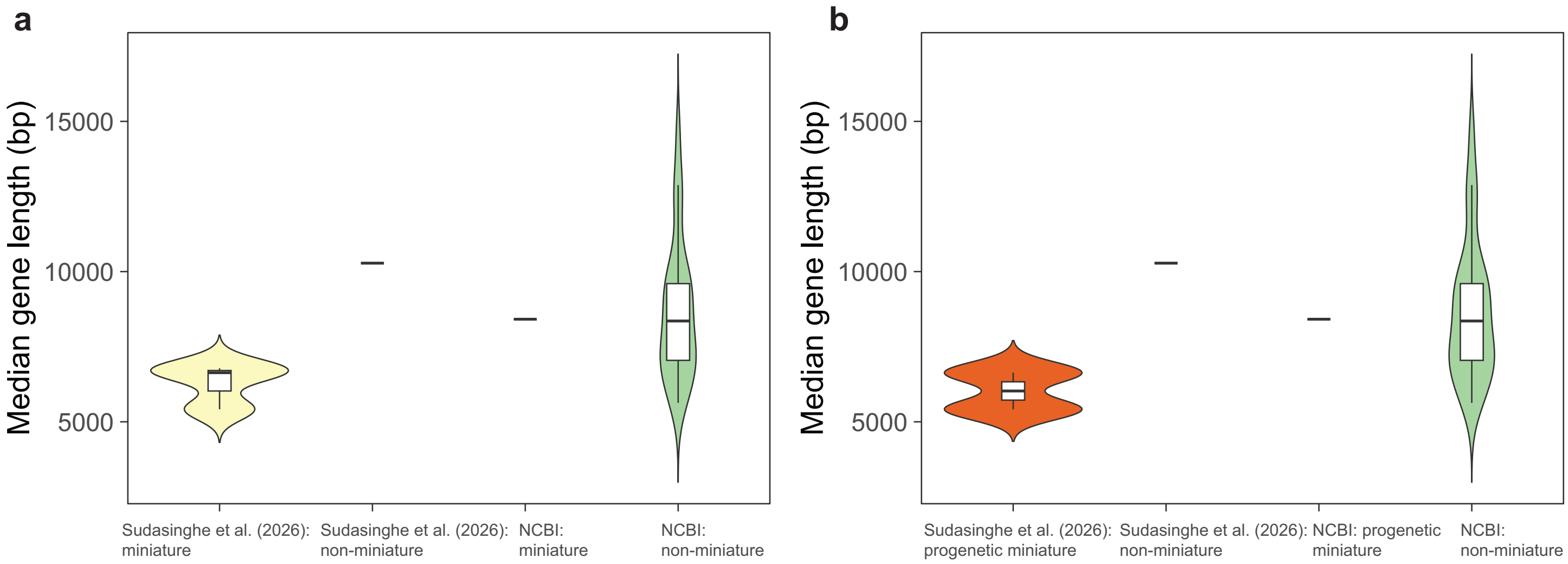

Fig. S22. Comparison of median gene lengths between annotation sources for **a)** miniature and non-miniature and **b)** progenetic miniature and non-miniature species, confirming the absence of systematic bias in gene length estimates between annotation sources in the 33-species comparative genomic dataset.

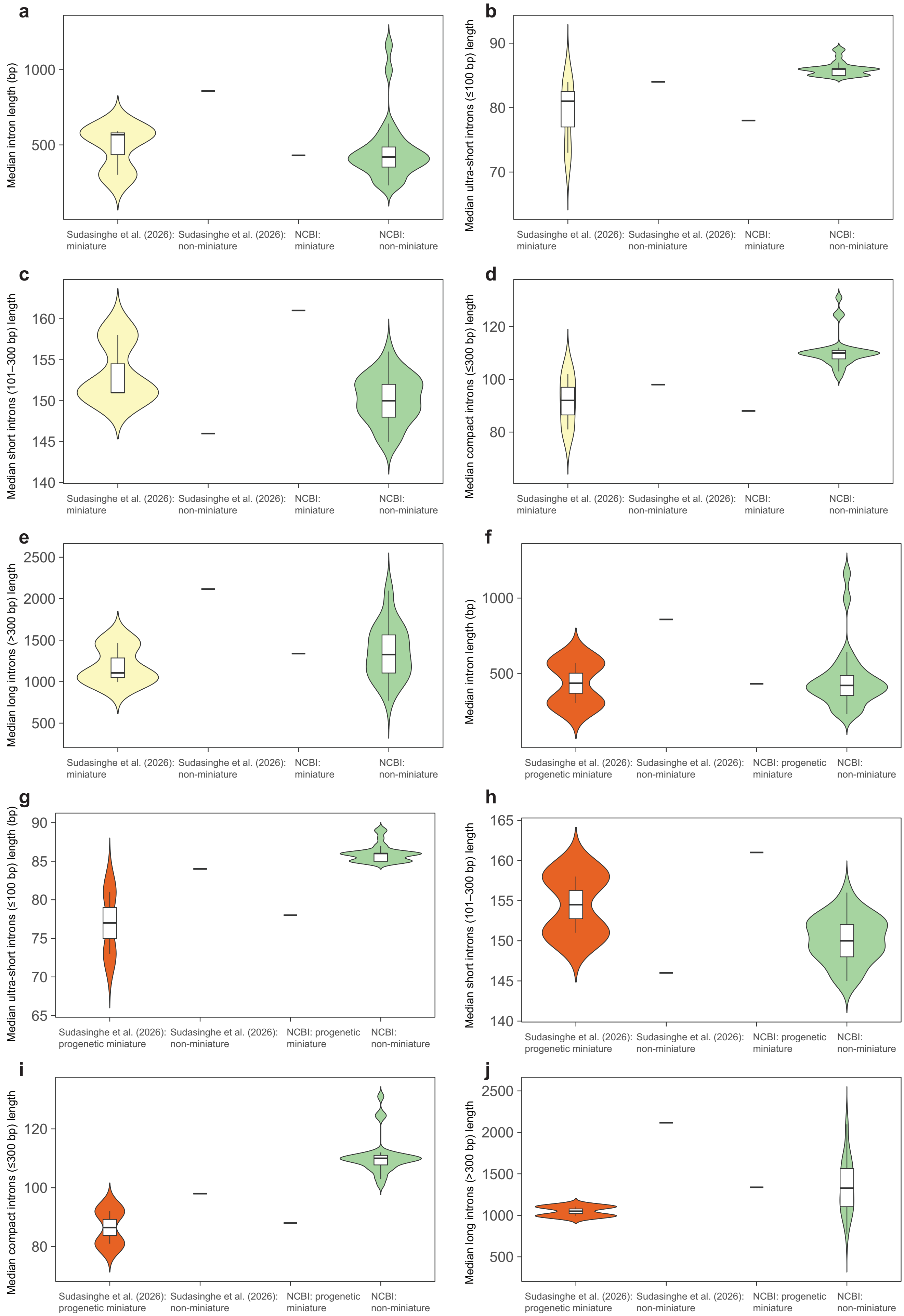

Fig. S23. Comparison of median intron lengths by size category between annotation sources for **a–e)** miniature and non-miniature and **f–j)** progenetic miniature and non-miniature species, confirming the absence of systematic bias in intron length estimates between annotation sources in the 33-species comparative genomic dataset. Panels show **a, f)** overall median intron length; **b, g)** median ultra-short intron length (≤100 bp); **c, h)** median short intron length (101–300 bp); **d, i)** median compact intron length (≤300 bp); and **e, j)** median long intron length (>300 bp).

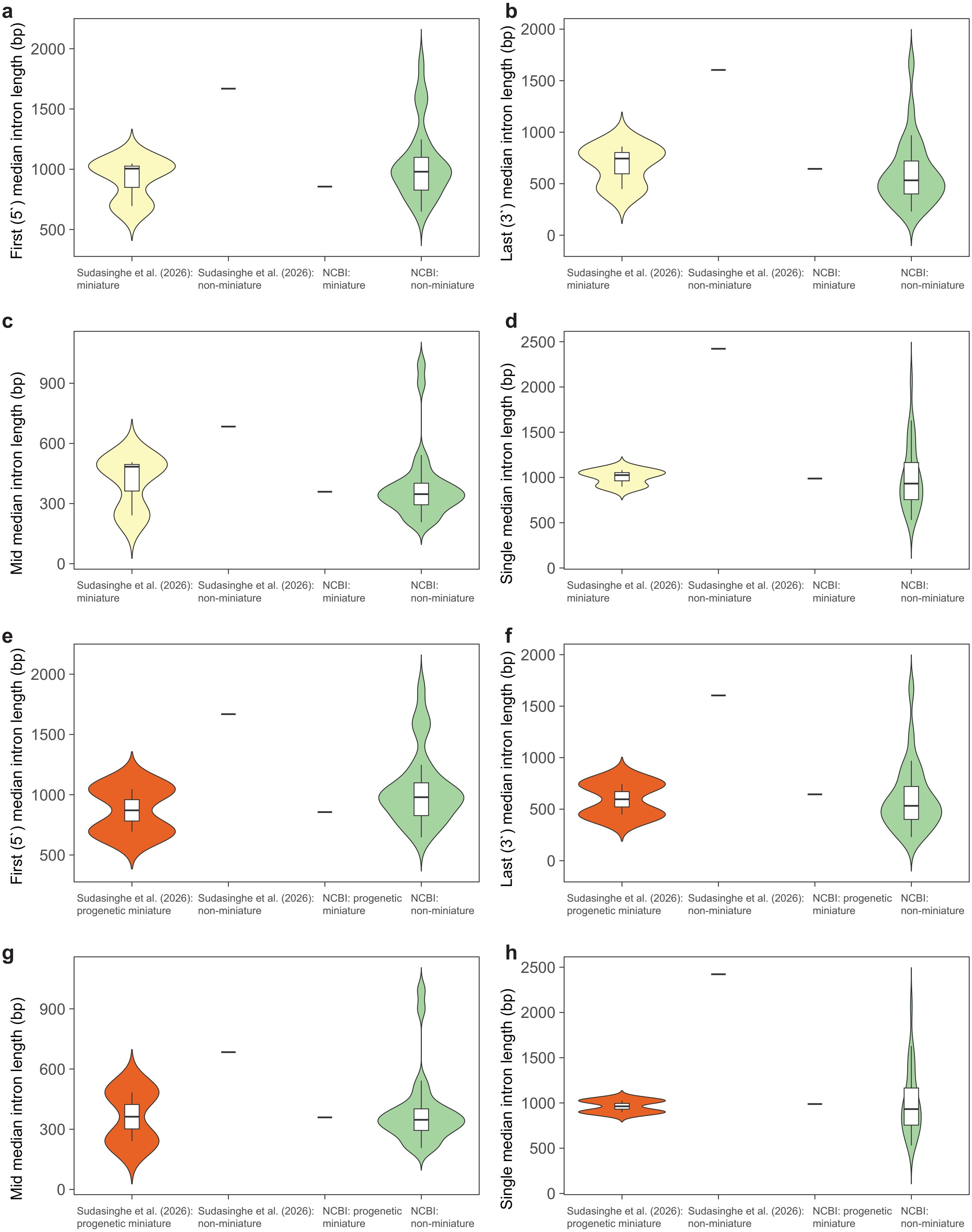

Fig. S24. Comparison of median intron lengths by positional category between annotation sources for **a–d)** miniature and non-miniature and **e–h)** progenetic miniature and non-miniature species, confirming the absence of systematic bias in intron length estimates between annotation sources in the 33-species comparative genomic dataset. Panels show **a, e)** first (5′) intron length; **b, f)** last (3′) intron length; **c, g)** middle intron length; and **d, h)** single intron length.

Tables

Table S1. Comparison of key divergence time estimates for major clades inferred in the present study with those reported in previously published molecular dating analyses. Node ages in the present study are reported as median estimates with 95% highest posterior density (HPD) intervals in million years (Ma).

Node	This study	Near et al. (2012)	Rabosky et al. (2013)	Chen et al. (2013)	Betancur-R et al. (2017)	Hughes et al. (2018)	Tao et al. (2019)	Šlechtová et al. (2021)	Šlechtová et al. (2025)	Lenglin et al. (2025)
Ostariophysi crown age (Gonorynchiformes & Otophysi)	193.4 (175.4-210.8)	215.2 (198-233.0)	146	163.8 (146.0-180.1)	200.3	160.8(154.0-170.3)	n/a	202	n/a	128
Otophysi crown age (Cypriniformes, Siluriformes, Gymnotiformes, Characiformes)	169.5 (149.7-188.3)	175.6 (156-187.8)	131.9	153.1 (136.4-169.8)	173.3	147.2 (138.2-156.7)	n/a	172.9	n/a	106
Siluriformes, Gymnotiformes, Characiformes crown age	136.2 (116.8-155.9)	135.7 (121.5-150.7)	111.9	136.1 (120.2-151.8)	162.2	127.9 (117.9-138.0)	n/a	115.1	n/a	94
Cypriniformes crown age (Paedocypris as sister group to the remaining Cypriniformes)	n/a	n/a	n/a	117.7 (94.5-138.6)	n/a	n/a	n/a	n/a	n/a	n/a
Cypriniformes crown age (Paedocypris, if included, not as sister group to the remaining Cypriniformes)	112.0 (91.6-133.0)	98.9 (82.4-118.6)	97.8	96.1 (77.2-115.1	100	97.4 (85.1-116.2)	154.5 (129.7-181.7)	92.3	77.0 (59.8-100.3)	49.3
Cobitoidei crown age	77.0 (62.9-92.1)	n/a	73.5	57.4 (39.7-75.4)	69	62.9 (50.0-78.6)	85.7 (52.4-115.6)	78.6	57.7 (45.7-75.2)	23.2
Cyprinoidei crown age (including Paedocypris)	88.3 (71.6-106.6)	n/a	n/a	n/a	83	n/a	n/a	n/a	n/a	n/a
Cyprinoidei crown age (excluding Paedocypris)	68.1 (55.9-82.1)	72.4 (53.9-91.4) Danio&Opsariichtys	85.2	78.3 (60.6-95.6)	71.4	63.2 (52.3-77.7)	59.2 (42.1-73.9)	82.9	n/a	41.4
Cobitoidei Botiidae crown age	61.9 (46.2–77.9)	n/a	39.3	n/a	23.4	n/a	49.0 (18.2-68.5)	66.4	47.2 (33.9-63.1)	16.6
Cobitoidei Cobitidae crown age	46.5 (36.8-57.4)	n/a	56.4	n/a	39.8	n/a	42.7 (32.6-57.0)	52.3	36.0 (28.7-44.4)	15
Cobitoidei Nemacheilidae crown age	43.5 (34.8-52.7)	n/a	51	n/a	36.5	n/a	41.6 (25.6-55.0)	50.1	39.1 (30.2-50.7)	15.8
Cobitoidei Balitoridae crown age	31.2 (24.3-39.1)	n/a	29.1	n/a	n/a	n/a	n/a	n/a	n/a	n/a
Cobitoidei Gastromyzontidae crown age	26.9 (20.4-34.1)	n/a	29.1	n/a	n/a	n/a	n/a	n/a	n/a	n/a
Cobitoidei Vaillantellidae stem age	72.6 (59.4-87.3)	n/a	65.8	57.0 (39.3-75.4)	65.3	n/a	n/a	n/a	54.6 (42.7-70.4)	22.6
Cobitoidei Ellopostomatidae stem age	57.6 (46.5-69.5)	n/a	63.6	51.5 (34.2-68.0)	n/a	n/a	n/a	n/a	42.0 (31.6-54.9)	n/a
Cobitoidei Serpenticobitidae stem age	32.9 (25.0-41.9)	n/a	66.0	n/a	n/a	n/a	n/a	n/a	n/a	n/a
Cobitoidei Barbuccidae stem age	40.8 (31.6-50.4)	n/a	44.7	n/a	n/a	n/a	n/a	n/a	n/a	n/a
Cyprinoidei Cyprinidae crown age	34.7 (27.5-42.2)	n/a	44.7	n/a	57.7	25.1 (17.6-34.5)	n/a	n/a	n/a	23
Cyprinoidei Danionidae crown age	62.7 (50.9-75.4)	n/a	76.8	61.4 (44.1-79.0)	66.3	n/a	n/a	n/a	n/a	34.9
Cyprinoidei Sundadanionidae crown age	47.2 (33.1-61.4)	n/a	n/a	n/a	n/a	n/a	n/a	n/a	n/a	n/a
Cyprinoidei Sundadanionidae stem age	64.9 (53.0-78.9)	n/a	66.3	50.4 (29.8-70.6)	68.4	n/a	n/a	n/a	n/a	n/a
Cyprinoidei Paedocyprididae crown age	24.4 (16.1-35.2)	n/a	n/a	n/a	n/a	n/a	n/a	n/a	n/a	n/a
Cyprinoidei Paedocyprididae stem age	88.4 (71.6-106.6)	n/a	n/a	117.7 (94.5-138.6)	83.3	n/a	n/a	n/a	n/a	n/a
Cyprinoidei Xenocyprididae crown age	19.3 (14.1-25.7)	n/a	35.6	n/a	47.4	n/a	n/a	n/a	n/a	10.7
Cyprinoidei Gobionidae crown age	19.4 (14.5-24.9)	n/a	37.9	n/a	41	n/a	n/a	n/a	n/a	n/a
Cyprinoidei Leuciscidae crown age	16.9 (13.3-21.0)	n/a	37.4	n/a	32.2	17.0 (10.5-26.1)	n/a	n/a	n/a	n/a
Cyprinoidei Acheilognathidae crown age	15.2 (10.4-20.4)	n/a	27.1	n/a	n/a	n/a	n/a	n/a	n/a	22.7
Cyprinoidei Leptobarbidae stem age	51.2 (36.5-67.3)	n/a	60.9	50.4 (29.8-70.6)	n/a	n/a	n/a	n/a	n/a	n/a
Cyprinoidei Psilorhynchidae stem age	48.1 (34.9-64.0)	n/a	67.4	62.5 (36.4-86.0)	n/a	n/a	n/a	n/a	n/a	n/a
Cyprinoidei Tincidae stem age	27.9 (21.4-35.0)	n/a	30.0	24.5 (15.4-39.0)	n/a	33.9 (24.5-46.0)	n/a	n/a	n/a	13.5
Cyprinoidei Tanichthyidae stem age	27.8 (21.3-35.0)	n/a	30.0	28.6 (16.5-41.5)	51.1	n/a	n/a	n/a	n/a	n/a
Catostomoidei Catostomidae crown age	26.3 (18.4-35.8)	52.8 (50.7-55.0)Hypentelium&Ictiobus calibration	42.1	n/a	33.1	n/a	62.9 (60.2-70.2)	45.8	73.1 (58.3-95.0)	13.8
Catostomoidei Catostomidae stem age	97.8 (81.0-117.5)	98.9 (82.4-118.6) Hypentelium&Ictiobus	92.5	82.6 (62.9-100.0)	92.1	n/a	154.5 (129.7-181.7)	82.9	17.7 (11.2-24.9)	26.6
Gyrinocheiloidei Gyrinocheilidae stem age	112.1 (91.6-133.0)	n/a	87.3	73.2 (54.0-91.5)	100	97.0 (85.0-116.1)	86.1 (52.2-116.1)	n/a	71.7 (62.4-93.2)	26.6

References

Betancur-R, R., Wiley, E. O., Arratia, G., Acero, A., Bailly, N., Miya, M., Lecointre, G., & Ortí, G. (2017). Phylogenetic classification of bony fishes. BMC Evolutionary Biology, 17(1), 162. https://doi.org/10.1186/s12862-017-0958-3

Chen, W.-J., Lavoué, S., & Mayden, R. L. (2013). Evolutionary origin and early biogeography of otophysan fishes (Ostariophysi: Teleostei). Evolution, 67(8), 2218–2239. https://doi.org/10.1111/evo.12104

Hughes, L. C., Ortí, G., Huang, Y., Sun, Y., Baldwin, C. C., Thompson, A. W., Arcila, D., Betancur-R., R., Li, C., Becker, L., Bellora, N., Zhao, X., Li, X., Wang, M., Fang, C., Xie, B., Zhou, Z., Huang, H., Chen, S., … Shi, Q. (2018). Comprehensive phylogeny of ray-finned fishes (Actinopterygii) based on transcriptomic and genomic data. Proceedings of the National Academy of Sciences, 115(24), 6249–6254. https://doi.org/10.1073/pnas.1719358115

Lenglin, A., Hidalgo, M., Miranda, G., De La Sota, A., Caminade, P., Belkhir, K., Otero, O., Antoine, P., Garcia-Davila, C., & Hubert, N. (2025). Early biogeography of Otophysi points to the Neotropics as the cradle of Characiphysan fishes. Ecology and Evolution, 15(11), e72431. https://doi.org/10.1002/ece3.72431

Near, T. J., Eytan, R. I., Dornburg, A., Kuhn, K. L., Moore, J. A., Davis, M. P., Wainwright, P. C., Friedman, M., & Smith, W. L. (2012). Resolution of ray-finned fish phylogeny and timing of diversification. Proceedings of the National Academy of Sciences, 109(34), 13698–13703. https://doi.org/10.1073/pnas.1206625109

Rabosky, D. L., Chang, J., Title, P. O., Cowman, P. F., Sallan, L., Friedman, M., Kaschner, K., Garilao, C., Near, T. J., Coll, M., & Alfaro, M. E. (2018). An inverse latitudinal gradient in speciation rate for marine fishes. Nature, 559(7714), 392–395. https://doi.org/10.1038/s41586-018-0273-1

Šlechtová, V. B., Dvořák, T., Freyhof, J., Kottelat, M., Levin, B., Golubtsov, A., Šlechta, V., & Bohlen, J. (2025). Reconstructing the phylogeny and evolutionary history of freshwater fishes (Nemacheilidae) across Eurasia since early Eocene. eLife, 13, RP101080. https://doi.org/10.7554/eLife.101080

Šlechtová, V. B., Musilova, Z., Tan, H. H., Kottelat, M., & Bohlen, J. (2021). One northward, one southward: Contrasting biogeographical history in two benthic freshwater fish genera across Southeast Asia (Teleostei: Cobitoidea: Nemacheilus, Pangio). Molecular Phylogenetics and Evolution, 161, 107139. https://doi.org/10.1016/j.ympev.2021.107139

Tao, W., Yang, L., Mayden, R. L., & He, S. (2019). Phylogenetic relationships of Cypriniformes and plasticity of pharyngeal teeth in the adaptive radiation of cyprinids. Science China Life Sciences, 62(4), 553–565. https://doi.org/10.1007/s11427-019-9480-3

Table S2. Results of phylogenetic generalized least squares (PGLS) regression analyses for log-transformed genome size and body size across Cypriniformes under a Brownian motion (BM) model of trait evolution.

Model	Predictor	β (estimate)	SE (Standard error)	t value	p-value	R²	Adjusted R²	N
log10GenomeSize ~ miniature vs non-miniature	Intercept	2.8456642	0.0822538	34.5961	<2e-16	0.0005666	-0.002689	309
	miniature	-0.0078134	0.0187284	-0.4172	0.6768			
log10GenomeSize ~ non-miniature vs proportioned-dwarf vs progenetic-miniature	Intercept	2.863487	0.082979	34.5085	<2e-16	0.007635	0.001149	309
	progenetic-miniature	-0.125775	0.08206	-1.5327	0.1264			
	proportioned-dwarf	-0.002135	0.019084	-0.1119	0.911			
log10GenomeSize ~ Ploidy	Intercept	2.790532	0.073569	37.9309	2.20E-16	0.2043	0.2017	309
	polyploid	0.259152	0.029188	8.8787	2.20E-16			
log10GenomeSize ~ miniature vs non-miniature + Ploidy	Intercept	2.7915258	0.0737551	37.8486	2.00E-16	0.2045	0.1993	309
	miniature	-0.0049448	0.0167387	-0.2954	0.7679			
	polyploid	0.2589849	0.0292369	8.8582	2.00E-16			
log10GenomeSize ~ non-miniature vs proportioned-dwarf vs progenetic-miniature + Ploidy	Intercept	2.80609131	0.07448703	37.6722	2.00E-16	0.2091	0.2013	309
	progenetic-miniature	-0.09937014	0.07344123	-1.3531	0.177			
	proportioned-dwarf	-0.00041616	0.01706662	-0.0244	0.9806			
	polyploid	0.25754284	0.02922184	8.8134	2.00E-16			
log10GenomeSize ~ log10BodySize	Intercept	2.823171	0.082791	34.1001	2.00E-16	0.008961	0.005733	309
	log10BodySize	0.020279	0.012171	1.6662	0.0967			
log10GenomeSize ~ log10BodySize + miniature vs non-miniature	Intercept	2.821967	0.083338	33.8617	2.00E-16	0.00903	0.002553	309
	log10BodySize	0.0209039	0.0129311	1.6166	0.107			
	miniature	0.0028718	0.0198144	0.1449	0.8849			
log10GenomeSize ~ log10BodySize + non-miniature vs proportioned-dwarf vs progenetic-miniature	Intercept	2.8398846	0.0841886	33.7324	2.00E-16	0.01534	0.005659	309
	log10BodySize	0.0199781	0.0129279	1.5453	0.1233			
	progenetic-miniature	-0.1092464	0.0825704	-1.3231	0.1868			
	proportioned-dwarf	0.0077728	0.0200915	0.3869	0.6991			
log10GenomeSize ~ log10BodySize + miniature vs non-miniature + Ploidy	Intercept	2.78684	0.0748799	37.2174	2.20E-16	0.2049	0.1971	309
	log10BodySize	0.0044689	0.0117556	0.3802	0.7041			
	miniature	-0.0026806	0.0177889	-0.1507	0.8803			
	polyploid	0.2571661	0.0296662	8.6687	2.63E-16			
log10GenomeSize ~ log10BodySize + non-miniature vs proportioned-dwarf vs progenetic-miniature + Ploidy	Intercept	2.8019784	0.0756924	37.0179	2.20E-16	0.2093	0.1989	309
	log10BodySize	0.0037676	0.0117545	0.3205	0.7488			
	progenetic-miniature	-0.0964086	0.0741276	-1.3006	0.1944			
	proportioned-dwarf	0.0014422	0.0180484	0.0799	0.9364			
	polyploid	0.2560255	0.0296453	8.6363	3.34E-16			
log10GenomeSize ~ log10BodySize * miniature vs non-miniature	Intercept	2.8232972	0.0836524	33.7503	2.00E-16	0.009215	-0.0005307	309
	log10BodySize	0.0206078	0.0130103	1.584	0.1142			
	miniature	-0.0096016	0.0558882	-0.1718	0.8637			
	log10BodySize:miniature	0.0348413	0.1459368	0.2387	0.8115			
log10GenomeSize ~ log10BodySize * non-miniature vs proportioned-dwarf vs progenetic-miniature	Intercept	2.842059	0.08445	33.6536	2.00E-16	0.01775	0.00154	309
	log10BodySize	0.020101	0.013003	1.5459	0.1232			
	progenetic-miniature	-0.173465	0.119207	-1.4552	0.1467			

	proportioned-dwarf	0.031958	0.0623	0.513	0.6083			
	log10BodySize:progenetic-miniature	0.293969	0.389548	0.7546	0.451			
	log10BodySize:proportioned-dwarf	-0.067048	0.162355	-0.413	0.6799			
log10BodySize ~ miniature vs non-miniature	Intercept	1.133629	0.362087	3.1308	0.001911	0.1113	0.1084	309
	miniature	-0.511156	0.082444	-6.2	1.82E-09			
log10BodySize ~ non-miniature vs proportioned-dwarf vs progenetic-miniature	Intercept	1.181398	0.366097	3.227	0.001387	0.1136	0.1078	309
	progenetic-miniature	-0.827324	0.362043	-2.2852	0.022988			
	proportioned-dwarf	-0.495936	0.084198	-5.8901	1.02E-08			

Table S3. Results of phylogenetic generalized least squares (PGLS) regression analyses for log-transformed genome size and body size across Cypriniformes under an Ornstein–Uhlenbeck (OU) model of trait evolution.

Model	Predictor	β (estimate)	SE (Standard error)	t value	p-value	R²	Adjusted R²	N
log10GenomeSize ~ miniature vs non-miniature	Intercept	2.8464091	0.4107606	6.9296	2.49E-11	0.0006119	-0.002643	309
	miniature	-0.0081419	0.0187795	-0.4336	0.6649			
log10GenomeSize ~ non-miniature vs proportioned-dwarf vs progenetic-miniature	Intercept	2.8641829	0.3877374	7.3869	1.44E-12	0.008068	0.001585	309
	progenetic-miniature	-0.1259239	0.079893	-1.5762	0.116			
	proportioned-dwarf	-0.0021376	0.0191639	-0.1115	0.9113			
log10GenomeSize ~ Ploidy	Intercept	2.792146	0.251733	11.0917	2.20E-16	0.2073	0.2047	309
	polyploid	0.259632	0.028978	8.9595	2.20E-16			
log10GenomeSize ~ miniature vs non-miniature + Ploidy	Intercept	2.7932193	0.2510412	11.1265	2.00E-16	0.2075	0.2024	309
	miniature	-0.0052409	0.0167967	-0.312	0.7552			
	polyploid	0.2594415	0.0290262	8.9382	2.00E-16			
log10GenomeSize ~ non-miniature vs proportioned-dwarf vs progenetic-miniature + Ploidy	Intercept	2.80767608	0.23605797	11.894	2.00E-16	0.2128	0.205	309
	progenetic-miniature	-0.09860622	0.06972523	-1.4142	0.1583			
	proportioned-dwarf	-0.00023076	0.01717182	-0.0134	0.9893			
	polyploid	0.25788167	0.02898577	8.8968	2.00E-16			
log10GenomeSize ~ log10BodySize	Intercept	2.823184	0.374153	7.5455	5.15E-13	0.009636	0.00641	309
	log10BodySize	0.021167	0.012247	1.7283	0.08495			
log10GenomeSize ~ log10BodySize + miniature vs non-miniature	Intercept	2.8219254	0.3745409	7.5344	5.58E-13	0.00971	0.003237	309
	log10BodySize	0.0218258	0.0130231	1.6759	0.09477			
	miniature	0.0029916	0.0198879	0.1504	0.88053			
log10GenomeSize ~ log10BodySize + non-miniature vs proportioned-dwarf vs progenetic-miniature	Intercept	2.839728	0.354962	8.0001	2.61E-14	0.01645	0.006777	309
	log10BodySize	0.020916	0.013028	1.6055	0.1094			
	progenetic-miniature	-0.10861	0.080079	-1.3563	0.176			
	proportioned-dwarf	0.008247	0.020192	0.4084	0.6832			
log10GenomeSize ~ log10BodySize + miniature vs non-miniature + Ploidy	Intercept	2.7877549	0.2455278	11.3541	2.00E-16	0.2082	0.2004	309
	log10BodySize	0.0053284	0.0118881	0.4482	0.6543			
	miniature	-0.002542	0.0178756	-0.1422	0.887			
	polyploid	0.2572796	0.0294603	8.7331	2.00E-16			
log10GenomeSize ~ log10BodySize + non-miniature vs proportioned-dwarf vs progenetic-miniature + Ploidy	Intercept	2.8027354	0.2319475	12.0835	2.00E-16	0.2133	0.2029	309
	log10BodySize	0.0046224	0.0118986	0.3885	0.6979			
	progenetic-miniature	-0.0949436	0.0703324	-1.3499	0.178			
	proportioned-dwarf	0.0020589	0.0181759	0.1133	0.9099			
	polyploid	0.2560225	0.0294153	8.7037	2.00E-16			
log10GenomeSize ~ log10BodySize * miniature vs non-miniature	Intercept	2.823346	0.373466	7.5598	4.77E-13	0.009929	0.0001903	309
	log10BodySize	0.02151	0.013105	1.6414	0.1017			
	miniature	-0.010417	0.055955	-0.1862	0.8524			
	log10BodySize:miniature	0.037505	0.146245	0.2565	0.7978			
log10GenomeSize ~ log10BodySize * non-miniature vs proportioned-dwarf vs progenetic-miniature	Intercept	2.841875	0.346977	8.1904	7.36E-15	0.01907	0.00288	309
	log10BodySize	0.021084	0.013107	1.6087	0.1087			
	progenetic-miniature	-0.174827	0.117309	-1.4903	0.1372			

	proportioned-dwarf	0.033439	0.062601	0.5342	0.5936			
	log10BodySize:progenetic-miniature	0.304054	0.389928	0.7798	0.4361			
	log10BodySize:proportioned-dwarf	-0.069796	0.163169	-0.4278	0.6691			
log10BodySize ~ miniature vs non-miniature	Intercept	1.002546	0.049468	20.267	2.20E-16	0.1827	0.1801	309
	miniature	-0.59795	0.072173	-8.285	3.71E-15			
log10BodySize ~ non-miniature vs proportioned-dwarf vs progenetic-miniature	Intercept	1.016456	0.050152	20.2677	2.20E-16	0.1892	0.1839	309
	progenetic-miniature	-0.810526	0.154112	-5.2593	2.72E-07			
	proportioned-dwarf	-0.545696	0.079413	-6.8716	3.56E-11			

Table S4. Phylogenetic signal estimates for transposable element content and gene architecture traits across Cypriniformes, reported as Pagel's λ and Blomberg's K with associated p values.

Trait	Pagel's λ	Pagel's λ-P-value	Blomberg's K	Blomberg's K-P-value
median gene length	0.802532	0.00245881	0.373982	0.013
overall median intron length	1.00969	3.02E-09	1.13564	0.001
first (5`) median intron length	0.992601	2.47E-06	0.762518	0.001
last (3`) median intron length	0.952229	2.47E-05	0.647167	0.001
mid median intron length	1.01547	1.49E-10	1.28209	0.001
single median intron length	0.509037	0.152696	0.277763	0.128
ultra-short introns (≤100 bp) median length	1.00366	1.88E-08	0.98057	0.003
short introns (101–300 bp) median length	1.00458	1.68E-06	1.13143	0.001
compact introns (≤300 bp) median length	1.01666	3.97E-12	1.38285	0.001
long introns (>300 bp) median length	1.01495	1.95E-07	0.916031	0.001
first (5`) ultra-short introns (≤100 bp) median intron length	0.908369	0.0019015	0.376057	0.046
last (3`) ultra-short introns (≤100 bp) median intron length	1.01666	5.24E-09	0.91926	0.003
mid ultra-short introns (≤100 bp) median intron length	1.00367	1.72E-08	0.987621	0.001
single ultra-short introns (≤100 bp) median intron length	7.45E-05	1	0.0779879	0.885
first (5`) short introns (101–300 bp) median intron length	0.482065	0.165687	0.298931	0.028
last (3`) short introns (101–300 bp) median intron length	0.908692	0.00215551	0.799045	0.001
mid short introns (101–300 bp) median intron length	1.0078	7.40E-07	1.17038	0.001
single short introns (101–300 bp) median intron length	7.45E-05	1	0.190763	0.353
first (5`) compact introns (≤300 bp) median intron length	0.922384	0.000444486	0.516034	0.002
last (3`) compact introns (≤300 bp) median intron length	0.995408	5.28E-09	1.25741	0.001
mid compact introns (≤300 bp) median intron length	1.0133	1.28E-10	1.35911	0.001
single compact introns (≤300 bp) median intron length	0.376595	0.574689	0.217382	0.166
first (5`) long introns (>300 bp) median intron length	0.995701	3.75E-06	0.764099	0.001
last (3`) long introns (>300 bp) median intron length	1.01666	5.82E-08	0.907078	0.001
mid long introns (>300 bp) median intron length	1.01618	9.21E-08	0.945564	0.001
single long introns (>300 bp) median intron length	0.726836	0.0284924	0.351664	0.012
median number of introns per gene	7.45E-05	1	0.107023	0.855
median number of mid introns per gene	7.45E-05	1	0.252534	0.071
proportion of compact introns (≤300 bp) per gene	1.00073	1.30E-10	1.61284	0.001
proportion of long introns (>300 bp) per gene	1.00073	1.30E-10	1.61284	0.001
% of non-repititive DNA	1.01036	6.81E-05	0.674322	0.001
% of total repeat DNA	1.01036	6.81E-05	0.674322	0.001
% of class I repeats	0.435864	0.624297	0.318678	0.013
% of class II repeats	0.957014	0.0017664	0.563171	0.001
% of other repeats	1.00736	1.18E-08	1.28411	0.001
% of retroelements	0.435864	0.624297	0.318678	0.022
% of total interspersed repeats	1.01163	2.61E-05	0.745715	0.001
% of LINEs	7.45E-05	1	0.178872	0.343
% of SINEs	1.00095	1.03E-06	0.774523	0.022

% of LTR	0.996143	0.207257	0.416149	0.004
% of DNA transposons	0.957351	0.00176658	0.563588	0.001
% of helitrons	1.01013	2.35E-05	0.650286	0.05
% of small RNA	0.796993	0.010617	0.490275	0.019
% of satellites	1.01666	0.000207951	0.445246	0.201
% of simple repeats	1.01666	2.32E-11	1.39963	0.001
% of low complexity repeats	1.01666	7.03E-13	2.28397	0.001
% of unclassified repeats	0.980167	4.60E-05	0.606783	0.001
% of recent (<5% K2P) class I repeats	7.45E-05	1	0.309686	0.017
% of recent (<5% K2P) class II repeats	0.963424	0.0231938	0.474302	0.001
% of recent (<5% K2P) other repeats	1.00932	0.189588	0.387764	0.222
% of recent (<5% K2P) LINEs	0.910883	0.30254	0.343248	0.033
% of recent (<5% K2P) SINEs	0.986788	6.95E-07	0.77696	0.012
% of recent (<5% K2P) LTR	7.45E-05	1	0.369941	0.011
% of recent (<5% K2P) DNA transposons	0.944907	0.0575844	0.457263	0.004
% of recent (<5% K2P) helitrons	1.00512	3.12E-05	0.632993	0.002
% of intermediate (5-20% K2P) class I repeats	7.45E-05	1	0.220081	0.147
% of intermediate (5-20% K2P) class II repeats	0.979662	0.000848262	0.590309	0.001
% of intermediate (5-20% K2P) other repeats	1.01062	8.44E-09	1.14364	0.028
% of intermediate (5-20% K2P) LINEs	0.498798	0.490254	0.183523	0.32
% of intermediate (5-20% K2P) SINEs	1.0158	3.73E-08	0.910588	0.013
% of intermediate (5-20% K2P) LTR	7.45E-05	1	0.227275	0.154
% of intermediate (5-20% K2P) DNA transposons	1.00911	0.000589941	0.606215	0.001
% of intermediate (5-20% K2P) helitrons	0.947989	0.00377356	0.475019	0.001
% of ancient (>20% K2P) class I repeats	7.45E-05	1	0.302804	0.032
% of ancient (>20% K2P) class II repeats	0.985574	2.46E-06	0.75023	0.001
% of ancient (>20% K2P) other repeats	1.01646	0.00130776	0.449547	0.009
% of ancient (>20% K2P) LINEs	7.45E-05	1	0.216739	0.207
% of ancient (>20% K2P) SINEs	0.867002	0.00524729	0.406832	0.107
% of ancient (>20% K2P) LTR	1.01661	0.0846263	0.389663	0.015
% of ancient (>20% K2P) DNA transposons	1.01661	4.46E-07	0.770712	0.001
% of ancient (>20% K2P) helitrons	0.929811	0.00513947	0.404155	0.026

Table S5. Results of phylogenetic generalized least squares (PGLS) regression analyses for transposable element content and gene architecture traits across Cypriniformes under a Brownian motion (BM) model of trait evolution, with miniaturization status as the categorical predictor.

Trait	Predictor	Intercept	β (estimate)	SE (Standard error)	t value	p-value	R^2	Adjusted R^2	N
median gene length	miniature vs non-miniature	5760.5	2955	1631.3	1.8114	0.07977	0.09572	0.06655	33
overall median intron length	miniature vs non-miniature	273.51	186.21	84.63	2.2003	0.03537	0.1351	0.1072	33
first (5`) median intron length	miniature vs non-miniature	625.44	449.37	144.58	3.1082	0.004012	0.2376	0.213	33
last (3`) median intron length	miniature vs non-miniature	248.14	378.84	193.35	1.9594	0.05911	0.1102	0.08149	33
mid median intron length	miniature vs non-miniature	242.305	143.579	68.917	2.0834	0.04556	0.1228	0.09452	33
single median intron length	miniature vs non-miniature	458.31	624.58	357.88	1.7452	0.09085	0.08946	0.06009	33
ultra-short introns (≤100 bp) median length	miniature vs non-miniature	81.0902	4.6592	1.0816	4.3075	0.0001543	0.3744	0.3543	33
short introns (101–300 bp) median length	miniature vs non-miniature	158.1352	-6.1909	1.5537	-3.9847	0.0003812	0.3387	0.3174	33
compact introns (≤300 bp) median length	miniature vs non-miniature	102.2557	10.95	3.3347	3.2836	0.002544	0.2581	0.2341	33
long introns (>300 bp) median length	miniature vs non-miniature	889.98	453.96	153.17	2.9637	0.0057966	0.2208	0.1957	33
first (5`) ultra-short introns (≤100 bp) median intron length	miniature vs non-miniature	80.8915	4.6347	1.9151	2.4201	0.02157	0.1589	0.1318	33
last (3`) ultra-short introns (≤100 bp) median intron length	miniature vs non-miniature	81.2226	4.5666	1.2663	3.6062	0.001077	0.2955	0.2728	33
mid ultra-short introns (≤100 bp) median intron length	miniature vs non-miniature	81.0869	4.6701	1.0781	4.332	0.0001441	0.3771	0.357	33
single ultra-short introns (≤100 bp) median intron length	miniature vs non-miniature	79.8125	2.8433	10.8576	0.2619	0.7952	0.002207	-0.02998	33
first (5`) short introns (101–300 bp) median intron length	miniature vs non-miniature	172.3188	3.3047	5.5495	0.5955	0.5558	0.01131	-0.02058	33
last (3`) short introns (101–300 bp) median intron length	miniature vs non-miniature	159.8659	-5.6716	2.6002	-2.1812	0.03688	0.1331	0.1051	33
mid short introns (101–300 bp) median intron length	miniature vs non-miniature	156.7372	-6.7037	1.4555	-4.6057	6.63E-05	0.4063	0.3871	33
single short introns (101–300 bp) median intron length	miniature vs non-miniature	182.904	-14.041	11.911	-1.1788	0.2474	0.0429	0.01203	33
first (5`) compact introns (≤300 bp) median intron length	miniature vs non-miniature	120.8675	17.9587	9.2845	1.9343	0.06225	0.1077	0.07891	33
last (3`) compact introns (≤300 bp) median intron length	miniature vs non-miniature	104.7157	12.5398	4.6039	2.7237	0.01051	0.1931	0.1671	33
mid compact introns (≤300 bp) median intron length	miniature vs non-miniature	101.3276	10.7677	3.2184	3.3457	0.002161	0.2653	0.2416	33
single compact introns (≤300 bp) median intron length	miniature vs non-miniature	117.0336	5.8117	14.9545	0.3886	0.7002	0.004848	-0.02725	33
first (5`) long introns (>300 bp) median intron length	miniature vs non-miniature	1126.62	597.24	194.2	3.0753	0.004365	0.2338	0.2091	33
last (3`) long introns (>300 bp) median intron length	miniature vs non-miniature	814.52	590.36	199.02	2.9664	0.005758	0.2211	0.196	33
mid long introns (>300 bp) median intron length	miniature vs non-miniature	873.5	411.77	146.78	2.8054	0.0086024	0.2025	0.1768	33
single long introns (>300 bp) median intron length	miniature vs non-miniature	1078.04	586.19	331.1	1.7704	0.08649	0.09182	0.06253	33
median number of introns per gene	miniature vs non-miniature	6.86998	-0.23253	0.79041	-0.2942	0.7706	0.002784	-0.02938	33
median number of mid introns per gene	miniature vs non-miniature	6.539729	0.098227	0.561621	0.1749	0.8623	0.0009858	-0.03124	33
proportion of compact introns (≤300 bp) per gene	miniature vs non-miniature	0.489083	-0.021446	0.022359	-0.9591	0.3449	0.02882	-0.002508	33
proportion of long introns (>300 bp) per gene	miniature vs non-miniature	0.510917	0.021446	0.022359	0.9591	0.3449	0.02882	-0.002508	33
% of non-repititive DNA	miniature vs non-miniature	62.6829	-9.4398	6.133	-1.5392	0.1339	0.071	0.04103	33
% of total repeat DNA	miniature vs non-miniature	37.3171	9.4398	6.133	1.5392	0.1339079	0.071	0.04103	33
% of class I repeats	miniature vs non-miniature	18.1668	1.7246	5.1411	0.3354	0.73955	0.003617	-0.02852	33
% of class II repeats	miniature vs non-miniature	13.0937	9.4497	4.7328	1.9966	0.05471	0.1139	0.08536	33
% of other repeats	miniature vs non-miniature	6.05659	-1.73452	0.84801	-2.0454	0.04938	0.1189	0.09049	33

% of retroelements	miniature vs non-miniature	18.1668	1.7246	5.1411	0.3354	0.73955	0.003617	-0.02852	33
% of total interspersed repeats	miniature vs non-miniature	33.8746	11.5361	6.1733	1.8687	0.0711398	0.1012	0.07225	33
% of LINEs	miniature vs non-miniature	6.21825	-0.97423	2.69598	-0.3614	0.7203	0.004195	-0.02793	33
% of SINEs	miniature vs non-miniature	0.61457	0.34539	0.96903	0.3564	0.7239	0.004081	-0.02804	33
% of LTR	miniature vs non-miniature	11.334	2.3534	3.7717	0.624	0.53722	0.0124	-0.01945	33
% of DNA transposons	miniature vs non-miniature	13.0476	9.4899	4.7324	2.0053	0.05373	0.1148	0.08627	33
% of helitrons	miniature vs non-miniature	0.046154	-0.0402	0.012203	-3.2944	0.002473	0.2593	0.2354	33
% of small RNA	miniature vs non-miniature	0.255961	-0.036109	0.110487	-0.3268	0.746	0.003434	-0.02871	33
% of satellites	miniature vs non-miniature	1.46E-04	-1.71E-04	3.82E-05	-4.4808	9.45E-05	0.3931	0.3735	33
% of simple repeats	miniature vs non-miniature	2.87053	-1.89276	0.56842	-3.3299	0.0022527	0.2634	0.2397	33
% of low complexity repeats	miniature vs non-miniature	0.269772	-0.127133	0.049815	-2.5521	0.0158553	0.1736	0.147	33
% of unclassified repeats	miniature vs non-miniature	2.66018	0.32166	0.47096	0.683	0.4996902	0.01482	-0.01696	33
% of recent (<5% K2P) class I repeats	miniature vs non-miniature	6.571	-1.5601	2.3635	-0.6601	0.51407	0.01386	-0.01795	33
% of recent (<5% K2P) class II repeats	miniature vs non-miniature	1.9705	2.4639	1.6839	1.4632	0.1535	0.0646	0.03443	33
% of recent (<5% K2P) other repeats	miniature vs non-miniature	-0.81488	1.81493	0.49916	3.636	0.0009932	0.299	0.2763	33
% of recent (<5% K2P) LINEs	miniature vs non-miniature	2.92467	-1.28544	0.96754	-1.3286	0.19369	0.05387	0.02335	33
% of recent (<5% K2P) SINEs	miniature vs non-miniature	0.121824	0.077911	0.146235	0.5328	0.598	0.009074	-0.02289	33
% of recent (<5% K2P) LTR	miniature vs non-miniature	3.5245	-0.3526	1.851	-0.1905	0.8502	0.001169	-0.03105	33
% of recent (<5% K2P) DNA transposons	miniature vs non-miniature	1.6266	2.4957	1.6155	1.5449	0.1325	0.07149	0.04153	33
% of recent (<5% K2P) helitrons	miniature vs non-miniature	0.343859	-0.031814	0.128197	-0.2482	0.80564	0.001983	-0.03021	33
% of intermediate (5-20% K2P) class I repeats	miniature vs non-miniature	10.6977	2.021	3.9074	0.5172	0.60867	0.008556	-0.02343	33
% of intermediate (5-20% K2P) class II repeats	miniature vs non-miniature	7.0056	7.9482	3.7754	2.1053	0.04347	0.1251	0.09687	33
% of intermediate (5-20% K2P) other repeats	miniature vs non-miniature	2.2075	-0.70616	0.45382	-1.556	0.129855	0.07244	0.04252	33
% of intermediate (5-20% K2P) LINEs	miniature vs non-miniature	3.64957	-0.62507	1.65299	-0.3781	0.7079	0.004592	-0.02752	33
% of intermediate (5-20% K2P) SINEs	miniature vs non-miniature	0.43762	0.20708	0.68801	0.301	0.7654	0.002914	-0.02925	33
% of intermediate (5-20% K2P) LTR	miniature vs non-miniature	6.6105	2.439	3.4158	0.714	0.4805	0.01618	-0.01556	33
% of intermediate (5-20% K2P) DNA transposons	miniature vs non-miniature	5.2581	6.0612	2.7516	2.2028	0.03517	0.1353	0.1075	33
% of intermediate (5-20% K2P) helitrons	miniature vs non-miniature	1.7475	1.887	1.4889	1.2674	0.2144	0.04927	0.0186	33
% of ancient (>20% K2P) class I repeats	miniature vs non-miniature	4.5681	1.4224	1.5913	0.8939	0.37827	0.02513	-0.00632	33
% of ancient (>20% K2P) class II repeats	miniature vs non-miniature	6.36973	0.83362	1.63719	0.5092	0.614236	0.008294	-0.0237	33
% of ancient (>20% K2P) other repeats	miniature vs non-miniature	0.63224	-0.12663	0.21796	-0.581	0.56546	0.01077	-0.02114	33
% of ancient (>20% K2P) LINEs	miniature vs non-miniature	1.307504	-0.019199	0.674233	-0.0285	0.9775	2.62E-05	-0.03223	33
% of ancient (>20% K2P) SINEs	miniature vs non-miniature	0.047395	0.193171	0.391003	0.494	0.6248	0.007812	-0.02419	33
% of ancient (>20% K2P) LTR	miniature vs non-miniature	3.2132	1.2485	1.2155	1.0271	0.3123	0.03291	0.001717	33
% of ancient (>20% K2P) DNA transposons	miniature vs non-miniature	4.61582	0.80958	1.25794	0.6436	0.52459	0.01318	-0.01865	33
% of ancient (>20% K2P) helitrons	miniature vs non-miniature	1.753908	0.024039	0.790573	0.0304	0.9759	2.98E-05	-0.03223	33
median gene length	progenetic-miniature vs non-miniature	6021.8	2645.8	2148.5	1.2315	0.22772	0.04812	0.01639	32

overall median intron length	progenetic-miniature vs non-miniature	278.11	180.76	111.55	1.6205	0.11559	0.08049	0.04984	32
first (5`) median intron length	progenetic-miniature vs non-miniature	682.81	381.49	189.58	2.0123	0.053243	0.1189	0.08956	32
last (3`) median intron length	progenetic-miniature vs non-miniature	341.76	268.08	252.88	1.0601	0.2976	0.03611	0.003979	32
mid median intron length	progenetic-miniature vs non-miniature	232.416	155.28	90.783	1.7105	0.09751	0.08886	0.05848	32
single median intron length	progenetic-miniature vs non-miniature	702.31	335.89	464.42	0.7232	0.4751	0.01714	-0.01563	32
ultra-short introns (≤100 bp) median length	progenetic-miniature vs non-miniature	78.2938	7.9678	1.0642	7.487	2.41E-08	0.6514	0.6398	32
short introns (101–300 bp) median length	progenetic-miniature vs non-miniature	159.5703	-7.8889	1.9893	-3.9657	0.0004196	0.3439	0.3221	32
compact introns (≤300 bp) median length	progenetic-miniature vs non-miniature	94.173	20.5133	3.4353	5.9714	1.51E-06	0.5431	0.5279	32
long introns (>300 bp) median length	progenetic-miniature vs non-miniature	911.05	429.04	201.78	2.1263	0.041822	0.131	0.102	32
first (5`) ultra-short introns (≤100 bp) median intron length	progenetic-miniature vs non-miniature	78.6006	7.3452	2.4017	3.0583	0.004652	0.2377	0.2123	32
last (3`) ultra-short introns (≤100 bp) median intron length	progenetic-miniature vs non-miniature	77.8117	8.6023	1.2029	7.1514	5.89E-08	0.6303	0.618	32
mid ultra-short introns (≤100 bp) median intron length	progenetic-miniature vs non-miniature	78.2871	7.9827	1.0569	7.5532	2.02E-08	0.6554	0.6439	32
single ultra-short introns (≤100 bp) median intron length	progenetic-miniature vs non-miniature	77.1179	6.0315	14.283	0.4223	0.6758	0.005909	-0.02723	32
first (5`) short introns (101–300 bp) median intron length	progenetic-miniature vs non-miniature	169.911	6.1535	7.2694	0.8465	0.404	0.02333	-0.009228	32
last (3`) short introns (101–300 bp) median intron length	progenetic-miniature vs non-miniature	160.4558	-6.3695	3.4217	-1.8615	0.0725	0.1035	0.07366	32
mid short introns (101–300 bp) median intron length	progenetic-miniature vs non-miniature	157.8575	-8.0292	1.8806	-4.2695	0.0001812	0.378	0.3572	32
single short introns (101–300 bp) median intron length	progenetic-miniature vs non-miniature	169.094	2.2985	14.9852	0.1534	0.8791	0.0007836	-0.03252	32
first (5`) compact introns (≤300 bp) median intron length	progenetic-miniature vs non-miniature	109.093	31.89	11.568	2.7567	0.009838	0.2021	0.1755	32
last (3`) compact introns (≤300 bp) median intron length	progenetic-miniature vs non-miniature	96.3233	22.4694	5.3592	4.1927	0.0002243	0.3695	0.3484	32
mid compact introns (≤300 bp) median intron length	progenetic-miniature vs non-miniature	93.8584	19.6049	3.4022	5.7624	2.71E-06	0.5254	0.5095	32
single compact introns (≤300 bp) median intron length	progenetic-miniature vs non-miniature	97.517	28.903	18.567	1.5567	0.1300332	0.07474	0.0439	32
first (5`) long introns (>300 bp) median intron length	progenetic-miniature vs non-miniature	1197.41	513.49	254.87	2.0148	0.0529699	0.1192	0.08982	32
last (3`) long introns (>300 bp) median intron length	progenetic-miniature vs non-miniature	878.49	514.68	261.44	1.9686	0.0583	0.1144	0.08488	32
mid long introns (>300 bp) median intron length	progenetic-miniature vs non-miniature	877.45	407.1	193.47	2.1042	0.0438477	0.1286	0.09956	32
single long introns (>300 bp) median intron length	progenetic-miniature vs non-miniature	1209.56	430.58	434.17	0.9917	0.32926	0.03174	-0.0005315	32
median number of introns per gene	progenetic-miniature vs non-miniature	6.47319	0.23695	1.03316	0.2293	0.8202	0.00175	-0.03152	32
median number of mid introns per gene	progenetic-miniature vs non-miniature	6.541825	0.095746	0.740315	0.1293	0.898	0.0005572	-0.03276	32
proportion of compact introns (≤300 bp) per gene	progenetic-miniature vs non-miniature	0.495843	-0.029443	0.029384	-1.002	0.3244	0.03238	0.0001289	32
proportion of long introns (>300 bp) per gene	progenetic-miniature vs non-miniature	0.504157	0.029443	0.029384	1.002	0.3244	0.03238	0.0001289	32
% of non-repititive DNA	progenetic-miniature vs non-miniature	66.4331	-13.8769	7.9836	-1.7382	0.09244	0.09149	0.06121	32
% of total repeat DNA	progenetic-miniature vs non-miniature	33.5669	13.8769	7.9836	1.7382	0.092435	0.09149	0.06121	32
% of class I repeats	progenetic-miniature vs non-miniature	14.348	6.2429	6.6518	0.9385	0.35547	0.02852	-0.003859	32
% of class II repeats	progenetic-miniature vs non-miniature	11.9181	10.8407	6.2259	1.7412	0.09189	0.09179	0.06151	32
% of other repeats	progenetic-miniature vs non-miniature	7.3009	-3.2067	1.035	-3.0982	0.004204	0.2424	0.2171	32
% of retroelements	progenetic-miniature vs non-miniature	14.348	6.2429	6.6518	0.9385	0.35547	0.02852	-0.003859	32
% of total interspersed repeats	progenetic-miniature vs non-miniature	29.2872	16.9637	7.9873	2.1238	0.042042	0.1307	0.1017	32
% of LINEs	progenetic-miniature vs non-miniature	6.8904	-1.7695	3.5464	-0.499	0.6214	0.00823	-0.02483	32

% of SINEs	progenetic-miniature vs non-miniature	0.44367	0.5476	1.27604	0.4291	0.6709	0.006101	-0.02703	32
% of LTR	progenetic-miniature vs non-miniature	7.0139	7.4648	4.7508	1.5713	0.1266	0.07604	0.04524	32
% of DNA transposons	progenetic-miniature vs non-miniature	11.8587	10.8966	6.225	1.7504	0.09026	0.09267	0.06243	32
% of helitrons	progenetic-miniature vs non-miniature	0.059397	-0.055868	0.015445	-3.6173	0.00108	0.3037	0.2805	32
% of small RNA	progenetic-miniature vs non-miniature	0.243218	-0.021032	0.145577	-0.1445	0.8861	0.0006952	-0.03261	32
% of satellites	progenetic-miniature vs non-miniature	-2.07E-06	4.01E-06	3.40E-06	1.1816	0.2466	0.04447	0.01262	32
% of simple repeats	progenetic-miniature vs non-miniature	3.65344	-2.81907	0.7006	-4.0238	0.0003577	0.3505	0.3289	32
% of low complexity repeats	progenetic-miniature vs non-miniature	0.323591	-0.19081	0.063075	-3.0252	0.005059	0.2337	0.2082	32
% of unclassified repeats	progenetic-miniature vs non-miniature	3.08063	-0.1758	0.60419	-0.291	0.773075	0.002814	-0.03043	32
% of recent (<5% K2P) class I repeats	progenetic-miniature vs non-miniature	5.0783	0.206	3.0741	0.067	0.947	0.0001497	-0.03318	32
% of recent (<5% K2P) class II repeats	progenetic-miniature vs non-miniature	1.4116	3.1251	2.2115	1.4131	0.1679	0.06241	0.03116	32
% of recent (<5% K2P) other repeats	progenetic-miniature vs non-miniature	0.17584	0.64275	0.56563	1.1363	0.2648	0.04127	0.009308	32
% of recent (<5% K2P) LINEs	progenetic-miniature vs non-miniature	3.5599	-2.037	1.257	-1.6205	0.1156	0.08048	0.04983	32
% of recent (<5% K2P) SINEs	progenetic-miniature vs non-miniature	0.039282	0.175572	0.190718	0.9206	0.3646	0.02747	-0.004944	32
% of recent (<5% K2P) LTR	progenetic-miniature vs non-miniature	1.4791	2.0674	2.3392	0.8838	0.3838	0.02538	-0.00711	32
% of recent (<5% K2P) DNA transposons	progenetic-miniature vs non-miniature	1.1627	3.0445	2.1236	1.4336	0.162	0.06412	0.03292	32
% of recent (<5% K2P) helitrons	progenetic-miniature vs non-miniature	0.248851	0.080596	0.165883	0.4859	0.6306	0.007807	-0.02527	32
% of intermediate (5-20% K2P) class I repeats	progenetic-miniature vs non-miniature	8.6221	4.4767	5.1023	0.8774	0.3872	0.02502	-0.007481	32
% of intermediate (5-20% K2P) class II repeats	progenetic-miniature vs non-miniature	5.5782	9.6371	4.953	1.9457	0.06111	0.1121	0.08245	32
% of intermediate (5-20% K2P) other repeats	progenetic-miniature vs non-miniature	2.84707	-1.46287	0.55747	-2.6241	0.01353	0.1867	0.1596	32
% of intermediate (5-20% K2P) LINEs	progenetic-miniature vs non-miniature	4.0823	-1.1371	2.174	-0.5231	0.6048	0.009037	-0.02399	32
% of intermediate (5-20% K2P) SINEs	progenetic-miniature vs non-miniature	0.19531	0.49377	0.90318	0.5467	0.5886	0.009865	-0.02314	32
% of intermediate (5-20% K2P) LTR	progenetic-miniature vs non-miniature	4.3445	5.1201	4.4365	1.1541	0.2576	0.04251	0.01059	32
% of intermediate (5-20% K2P) DNA transposons	progenetic-miniature vs non-miniature	4.8014	6.6016	3.6237	1.8218	0.07848	0.09961	0.06959	32
% of intermediate (5-20% K2P) helitrons	progenetic-miniature vs non-miniature	0.7768	3.0355	1.9347	1.569	0.1271	0.07583	0.04503	32
% of ancient (>20% K2P) class I repeats	progenetic-miniature vs non-miniature	4.2552	1.7926	2.0949	0.8557	0.39895	0.02383	-0.008714	32
% of ancient (>20% K2P) class II repeats	progenetic-miniature vs non-miniature	7.04772	0.03144	2.14581	0.0147	0.988407	7.16E-06	-0.03333	32
% of ancient (>20% K2P) other repeats	progenetic-miniature vs non-miniature	0.64769	-0.14491	0.28727	-0.5044	0.61763	0.008411	-0.02464	32
% of ancient (>20% K2P) LINEs	progenetic-miniature vs non-miniature	1.55262	-0.30921	0.88486	-0.3495	0.7292	0.004054	-0.02914	32
% of ancient (>20% K2P) SINEs	progenetic-miniature vs non-miniature	0.031162	0.212378	0.515381	0.4121	0.6832	0.005628	-0.02752	32
% of ancient (>20% K2P) LTR	progenetic-miniature vs non-miniature	2.6715	1.8895	1.5916	1.1871	0.2445	0.04487	0.01303	32
% of ancient (>20% K2P) DNA transposons	progenetic-miniature vs non-miniature	5.256079	0.052044	1.643893	0.0317	0.97495	3.34E-05	-0.0333	32
% of ancient (>20% K2P) helitrons	progenetic-miniature vs non-miniature	1.79164	-0.020605	1.042035	-0.0198	0.9844	1.30E-05	-0.03332	32

Table S6. Results of phylogenetic generalized least squares (PGLS) regression analyses for transposable element content and gene architecture traits across Cypriniformes under an Ornstein–Uhlenbeck (OU) model of trait evolution, with miniaturization status as the categorical predictor.

Trait	Predictor	Intercept	β (estimate)	SE (Standard error)	t value	p-value	R²	Adjusted R²	N
median gene length	miniature vs non-miniature	6553.6	2433.5	1291.6	1.8841	0.06897	0.1027	0.0738	33
overall median intron length	miniature vs non-miniature	284.74	178.165	85.274	2.0893	0.04498	0.1234	0.09516	33
first (5`) median intron length	miniature vs non-miniature	657.53	424.69	144.34	2.9423	0.006118	0.2183	0.1931	33
last (3`) median intron length	miniature vs non-miniature	365.03	281.77	185.51	1.5189	0.1389	0.06926	0.03924	33
mid median intron length	miniature vs non-miniature	248.927	139.069	69.576	1.9988	0.05446	0.1142	0.08559	33
single median intron length	miniature vs non-miniature	956.21	146.86	244.79	0.6	0.552898	0.01148	-0.02041	33
ultra-short introns (≤100 bp) median length	miniature vs non-miniature	80.7725	4.9859	1.0848	4.5962	6.81E-05	0.4053	0.3861	33
short introns (101–300 bp) median length	miniature vs non-miniature	157.7984	-6.0813	1.5463	-3.9329	0.0004401	0.3329	0.3113	33
compact introns (≤300 bp) median length	miniature vs non-miniature	101.621	11.45	3.369	3.3985	0.001879	0.2714	0.2479	33
long introns (>300 bp) median length	miniature vs non-miniature	918.56	435.65	153.81	2.8324	0.008048	0.2056	0.18	33
first (5`) ultra-short introns (≤100 bp) median intron length	miniature vs non-miniature	79.5	6.175	1.00612	6.1374	8.30E-07	0.5486	0.534	33
last (3`) ultra-short introns (≤100 bp) median intron length	miniature vs non-miniature	80.788	5.0011	1.2675	3.9455	0.0004249	0.3343	0.3128	33
mid ultra-short introns (≤100 bp) median intron length	miniature vs non-miniature	80.7738	4.9929	1.0814	4.6169	6.42E-05	0.4074	0.3883	33
single ultra-short introns (≤100 bp) median intron length	miniature vs non-miniature	78.75	3.7901	3.5103	1.0797	0.2886	0.03624	0.005154	33
first (5`) short introns (101–300 bp) median intron length	miniature vs non-miniature	165.9076	8.1078	3.1646	2.562	0.01549	0.1747	0.1481	33
last (3`) short introns (101–300 bp) median intron length	miniature vs non-miniature	157.7819	-4.8387	2.303	-2.101	0.04387	0.1246	0.09641	33
mid short introns (101–300 bp) median intron length	miniature vs non-miniature	156.4888	-6.6116	1.4589	-4.5321	8.17E-05	0.3985	0.3791	33
single short introns (101–300 bp) median intron length	miniature vs non-miniature	175	-7.545	5.8181	-1.2968	0.2043	0.05146	0.02086	33
first (5`) compact introns (≤300 bp) median intron length	miniature vs non-miniature	104.633	32.9961	5.9607	5.5356	4.62E-06	0.4971	0.4809	33
last (3`) compact introns (≤300 bp) median intron length	miniature vs non-miniature	103.3324	13.5511	4.6259	2.9294	0.006319	0.2168	0.1915	33
mid compact introns (≤300 bp) median intron length	miniature vs non-miniature	100.7136	11.2389	3.2511	3.457	0.001608	0.2782	0.255	33
single compact introns (≤300 bp) median intron length	miniature vs non-miniature	104.3751	21.0359	7.3408	2.8656	0.007412	0.2094	0.1839	33
first (5`) long introns (>300 bp) median intron length	miniature vs non-miniature	1172.23	564.17	193.88	2.9099	0.006636	0.2145	0.1892	33
last (3`) long introns (>300 bp) median intron length	miniature vs non-miniature	860.55	558.48	199.5	2.7994	0.008732	0.2018	0.176	33
mid long introns (>300 bp) median intron length	miniature vs non-miniature	899.44	396.25	147.4	2.6882	0.01145	0.189	0.1629	33
single long introns (>300 bp) median intron length	miniature vs non-miniature	1407.36	300.62	255.56	1.1763	0.2484	0.04273	0.01185	33
median number of introns per gene	miniature vs non-miniature	6.5	0.13323	0.30012	0.4439	0.6602	0.006317	-0.02574	33
median number of mid introns per gene	miniature vs non-miniature	6.25024	0.14972	0.29185	0.513	0.6116	0.008418	-0.02357	33
proportion of compact introns (≤300 bp) per gene	miniature vs non-miniature	0.486839	-0.020464	0.02258	-0.9063	0.3718	0.02581	-0.005614	33
proportion of long introns (>300 bp) per gene	miniature vs non-miniature	0.513161	0.020464	0.02258	0.9063	0.3718	0.02581	-0.005614	33
% of non-repititive DNA	miniature vs non-miniature	60.7506	-9.0898	5.8063	-1.5655	0.1276	0.07327	0.04337	33
% of total repeat DNA	miniature vs non-miniature	39.2494	9.0898	5.8063	1.5655	0.1276	0.07327	0.04337	33
% of class I repeats	miniature vs non-miniature	18.7564	2.7343	3.3736	0.8105	0.4238	0.02075	-0.01084	33
% of class II repeats	miniature vs non-miniature	15.2929	8.9797	4.1526	2.1624	0.038416	0.1311	0.103	33
% of other repeats	miniature vs non-miniature	6.22176	-2.02216	0.84678	-2.3881	0.023216	0.1554	0.1281	33

% of retroelements	miniature vs non-miniature	18.7564	2.7343	3.3736	0.8105	0.4238	0.02075	-0.01084	33
% of total interspersed repeats	miniature vs non-miniature	35.4314	11.6487	5.9227	1.9668	0.0582131	0.1109	0.08226	33
% of LINEs	miniature vs non-miniature	6.9164	-1.4028	1.291	-1.0867	0.2856	0.03669	0.005619	33
% of SINEs	miniature vs non-miniature	0.78738	0.25555	0.9352	0.2733	0.7865	0.002403	-0.02978	33
% of LTR	miniature vs non-miniature	10.5912	4.2241	2.8443	1.4851	0.1476177	0.06642	0.0363	33
% of DNA transposons	miniature vs non-miniature	15.2467	9.0191	4.1525	2.172	0.037628	0.1321	0.1041	33
% of helitrons	miniature vs non-miniature	0.046005	-0.039916	0.012094	-3.3006	0.002433	0.26	0.2362	33
% of small RNA	miniature vs non-miniature	0.168196	-0.054382	0.069043	-0.7877	0.43687	0.01962	-0.012	33
% of satellites	miniature vs non-miniature	1.31E-04	-1.52E-04	3.78E-05	-4.0075	0.0003577	0.3413	0.32	33
% of simple repeats	miniature vs non-miniature	2.90529	-1.97159	0.57331	-3.4389	0.001687	0.2761	0.2528	33
% of low complexity repeats	miniature vs non-miniature	0.269832	-0.130838	0.050404	-2.5958	0.0143	0.1786	0.1521	33
% of unclassified repeats	miniature vs non-miniature	2.971195	0.017205	0.437854	0.0393	0.9689	4.98E-05	-0.03221	33
% of recent (<5% K2P) class I repeats	miniature vs non-miniature	6.77744	-0.52856	1.45396	-0.3635	0.7187	0.004245	-0.02788	33
% of recent (<5% K2P) class II repeats	miniature vs non-miniature	2.7953	2.0108	1.4059	1.4302	0.16266	0.0619	0.03164	33
% of recent (<5% K2P) other repeats	miniature vs non-miniature	-0.51353	1.4413	0.49031	2.9396	0.00616	0.218	0.1928	33
% of recent (<5% K2P) LINEs	miniature vs non-miniature	3.11065	-1.1898	0.65575	-1.8144	0.0793	0.096	0.06684	33
% of recent (<5% K2P) SINEs	miniature vs non-miniature	0.142848	0.070457	0.140251	0.5024	0.619	0.008075	-0.02392	33
% of recent (<5% K2P) LTR	miniature vs non-miniature	3.45711	0.56177	1.29416	0.4341	0.667236	0.006042	-0.02602	33
% of recent (<5% K2P) DNA transposons	miniature vs non-miniature	2.4981	2.0061	1.3164	1.524	0.13764	0.0697	0.03969	33
% of recent (<5% K2P) helitrons	miniature vs non-miniature	0.347891	-0.028669	0.119315	-0.2403	0.8117	0.001859	-0.03034	33
% of intermediate (5-20% K2P) class I repeats	miniature vs non-miniature	10.8397	1.5017	2.1577	0.696	0.4916	0.01538	-0.01638	33
% of intermediate (5-20% K2P) class II repeats	miniature vs non-miniature	8.7812	7.4085	3.3593	2.2054	0.03497	0.1356	0.1077	33
% of intermediate (5-20% K2P) other repeats	miniature vs non-miniature	2.23827	-0.75211	0.45843	-1.6406	0.111	0.07989	0.05021	33
% of intermediate (5-20% K2P) LINEs	miniature vs non-miniature	4.08796	-1.26257	0.80249	-1.5733	0.1258	0.07395	0.04407	33
% of intermediate (5-20% K2P) SINEs	miniature vs non-miniature	0.50799	0.18195	0.68005	0.2676	0.7908	0.002304	-0.02988	33
% of intermediate (5-20% K2P) LTR	miniature vs non-miniature	5.864	2.918	1.8657	1.564	0.127972	0.07313	0.04324	33
% of intermediate (5-20% K2P) DNA transposons	miniature vs non-miniature	6.4963	5.6251	2.5062	2.2445	0.03208	0.1398	0.112	33
% of intermediate (5-20% K2P) helitrons	miniature vs non-miniature	2.2852	1.7919	1.188	1.5083	0.1416	0.06837	0.03832	33
% of ancient (>20% K2P) class I repeats	miniature vs non-miniature	4.9934	1.27101	1.04385	1.2176	0.2326	0.04564	0.01486	33
% of ancient (>20% K2P) class II repeats	miniature vs non-miniature	6.44769	0.95619	1.59638	0.599	0.55354	0.01144	-0.02045	33
% of ancient (>20% K2P) other repeats	miniature vs non-miniature	0.68907	-0.16709	0.1753	-0.9531	0.3478883	0.02847	-0.002868	33
% of ancient (>20% K2P) LINEs	miniature vs non-miniature	1.60402	-0.24238	0.35738	-0.6782	0.5027	0.01462	-0.01716	33
% of ancient (>20% K2P) SINEs	miniature vs non-miniature	0.201797	0.080626	0.309689	0.2603	0.7963	0.002182	-0.03001	33
% of ancient (>20% K2P) LTR	miniature vs non-miniature	3.14378	1.48696	0.94241	1.5778	0.124755	0.07434	0.04448	33
% of ancient (>20% K2P) DNA transposons	miniature vs non-miniature	4.68757	0.85802	1.23946	0.6923	0.49393	0.01522	-0.01654	33
% of ancient (>20% K2P) helitrons	miniature vs non-miniature	1.67531	0.25061	0.62248	0.4026	0.690011	0.005201	-0.02689	33
median gene length	progenetic-miniature vs non-miniature	6673.5	2302.6	1520.5	1.5144	0.1404	0.07101	0.04005	32

overall median intron length	progenetic-miniature vs non-miniature	289.66	172.62	110.74	1.5588	0.1295	0.07493	0.04409	32
first (5`) median intron length	progenetic-miniature vs non-miniature	709.07	364.36	184.33	1.9767	0.05733	0.1152	0.08574	32
last (3`) median intron length	progenetic-miniature vs non-miniature	427.36	208.12	230.01	0.9048	0.3728	0.02657	-0.005882	32
mid median intron length	progenetic-miniature vs non-miniature	239.85	149.927	90.601	1.6548	0.1084	0.08364	0.0531	32
single median intron length	progenetic-miniature vs non-miniature	966.69	138.37	285.55	0.4846	0.6314911	0.007767	-0.02531	32
ultra-short introns (≤100 bp) median length	progenetic-miniature vs non-miniature	78.1422	8.0863	1.046	7.7309	1.26E-08	0.6658	0.6547	32
short introns (101–300 bp) median length	progenetic-miniature vs non-miniature	159.102	-7.6148	1.9216	-3.9628	0.0004229	0.3436	0.3217	32
compact introns (≤300 bp) median length	progenetic-miniature vs non-miniature	93.8708	20.6779	3.4514	5.9912	1.43E-06	0.5447	0.5296	32
long introns (>300 bp) median length	progenetic-miniature vs non-miniature	936.75	414.87	198.55	2.0895	0.04524	0.127	0.09795	32
first (5`) ultra-short introns (≤100 bp) median intron length	progenetic-miniature vs non-miniature	78	7.67245	1.00682	7.6205	1.69E-08	0.6594	0.648	32
last (3`) ultra-short introns (≤100 bp) median intron length	progenetic-miniature vs non-miniature	77.5983	8.7553	1.1743	7.4558	2.61E-08	0.6495	0.6378	32
mid ultra-short introns (≤100 bp) median intron length	progenetic-miniature vs non-miniature	78.1396	8.0986	1.0396	7.7897	1.08E-08	0.6692	0.6581	32
single ultra-short introns (≤100 bp) median intron length	progenetic-miniature vs non-miniature	76.5	6.0401	3.9629	1.5242	0.1379	0.07187	0.04093	32
first (5`) short introns (101–300 bp) median intron length	progenetic-miniature vs non-miniature	166.6667	7.3468	3.6352	2.021	0.05228	0.1198	0.0905	32
last (3`) short introns (101–300 bp) median intron length	progenetic-miniature vs non-miniature	158.2623	-5.4271	2.749	-1.9742	0.05762	0.115	0.08548	32
mid short introns (101–300 bp) median intron length	progenetic-miniature vs non-miniature	157.5322	-7.8438	1.8439	-4.2539	0.0001893	0.3762	0.3554	32
single short introns (101–300 bp) median intron length	progenetic-miniature vs non-miniature	166.3333	1.1217	5.8407	0.1921	0.849	0.001228	-0.03206	32
first (5`) compact introns (≤300 bp) median intron length	progenetic-miniature vs non-miniature	100.883	36.9138	6.9612	5.3028	9.91E-06	0.4838	0.4666	32
last (3`) compact introns (≤300 bp) median intron length	progenetic-miniature vs non-miniature	95.3678	22.974	5.2942	4.3394	0.0001491	0.3856	0.3652	32
mid compact introns (≤300 bp) median intron length	progenetic-miniature vs non-miniature	93.544	19.7718	3.4153	5.7891	2.52E-06	0.5277	0.5119	32
single compact introns (≤300 bp) median intron length	progenetic-miniature vs non-miniature	93.5	31.853	7.3569	4.3296	0.0001533	0.3846	0.364	32
first (5`) long introns (>300 bp) median intron length	progenetic-miniature vs non-miniature	1234.73	491.08	247.75	1.9822	0.05669	0.1158	0.08633	32
last (3`) long introns (>300 bp) median intron length	progenetic-miniature vs non-miniature	916.47	493.05	256.38	1.9231	0.064	0.1098	0.08008	32
mid long introns (>300 bp) median intron length	progenetic-miniature vs non-miniature	902.14	393.84	190.36	2.0689	0.04726	0.1249	0.0957	32
single long introns (>300 bp) median intron length	progenetic-miniature vs non-miniature	1425.02	280.91	300.48	0.9349	0.3573	0.02831	-0.004083	32
median number of introns per gene	progenetic-miniature vs non-miniature	6.33333	0.29989	0.34073	0.8801	0.3858	0.02517	-0.007323	32
median number of mid introns per gene	progenetic-miniature vs non-miniature	6.333333	0.067018	0.335273	0.1999	0.8429	0.00133	-0.03196	32
proportion of compact introns (≤300 bp) per gene	progenetic-miniature vs non-miniature	0.493354	-0.028225	0.02936	-0.9613	0.3441	0.02988	-0.002453	32
proportion of long introns (>300 bp) per gene	progenetic-miniature vs non-miniature	0.506646	0.028225	0.02936	0.9613	0.3441	0.02988	-0.002453	32
% of non-repititive DNA	progenetic-miniature vs non-miniature	63.9085	-12.8731	6.9622	-1.849	0.07433	0.1023	0.07238	32
% of total repeat DNA	progenetic-miniature vs non-miniature	36.0915	12.8731	6.9622	1.849	0.0743323	0.1023	0.07238	32
% of class I repeats	progenetic-miniature vs non-miniature	16.2367	5.3002	3.751	1.413	0.1679	0.0624	0.03115	32
% of class II repeats	progenetic-miniature vs non-miniature	13.9912	10.5068	4.8762	2.1547	0.039338	0.134	0.1052	32
% of other repeats	progenetic-miniature vs non-miniature	7.3656	-3.3591	1.0058	-3.3396	0.002254	0.271	0.2467	32
% of retroelements	progenetic-miniature vs non-miniature	16.2367	5.3002	3.751	1.413	0.1679	0.0624	0.03115	32
% of total interspersed repeats	progenetic-miniature vs non-miniature	31.5627	16.2805	7.1307	2.2832	0.029675	0.148	0.1196	32
% of LINEs	progenetic-miniature vs non-miniature	7.4508	-1.9375	1.475	-1.3136	0.199	0.05439	0.02287	32

% of SINEs	progenetic-miniature vs non-miniature	0.75112	0.3002	1.16281	0.2582	0.798	0.002217	-0.03104	32
% of LTR	progenetic-miniature vs non-miniature	7.3962	7.5791	2.9843	2.5397	0.01651	0.177	0.1495	32
% of DNA transposons	progenetic-miniature vs non-miniature	13.9374	10.5549	4.8756	2.1648	0.038485	0.1351	0.1063	32
% of helitrons	progenetic-miniature vs non-miniature	0.057517	-0.053438	0.014913	-3.5834	0.001183	0.2997	0.2764	32
% of small RNA	progenetic-miniature vs non-miniature	0.162026	-0.048213	0.07974	-0.6046	0.54998	0.01204	-0.02089	32
% of satellites	progenetic-miniature vs non-miniature	-1.94E-06	3.90E-06	3.39E-06	1.1506	0.259	0.04226	0.01034	32
% of simple repeats	progenetic-miniature vs non-miniature	3.65306	-2.84885	0.69891	-4.0761	0.0003096	0.3564	0.335	32
% of low complexity repeats	progenetic-miniature vs non-miniature	0.322526	-0.192931	0.063395	-3.0433	0.004832	0.2359	0.2104	32
% of unclassified repeats	progenetic-miniature vs non-miniature	3.34673	-0.40821	0.51089	-0.799	0.4306	0.02084	-0.0118	32
% of recent (<5% K2P) class I repeats	progenetic-miniature vs non-miniature	5.66959	0.59329	1.61455	0.3675	0.715852	0.004481	-0.0287	32
% of recent (<5% K2P) class II repeats	progenetic-miniature vs non-miniature	1.9081	3.0015	1.5353	1.955	0.05996	0.113	0.08344	32
% of recent (<5% K2P) other repeats	progenetic-miniature vs non-miniature	0.19617	0.61828	0.55992	1.1042	0.2783	0.03906	0.007025	32
% of recent (<5% K2P) LINEs	progenetic-miniature vs non-miniature	3.50698	-1.59005	0.73604	-2.1603	0.03887	0.1346	0.1058	32
% of recent (<5% K2P) SINEs	progenetic-miniature vs non-miniature	0.086663	0.134859	0.173462	0.7775	0.443	0.01975	-0.01292	32
% of recent (<5% K2P) LTR	progenetic-miniature vs non-miniature	1.9756	2.11	1.3497	1.5633	0.1285	0.07533	0.04451	32
% of recent (<5% K2P) DNA transposons	progenetic-miniature vs non-miniature	1.6809	2.9075	1.4318	2.0307	0.05123	0.1209	0.09155	32
% of recent (<5% K2P) helitrons	progenetic-miniature vs non-miniature	0.242729	0.089771	0.134502	0.6674	0.5096	0.01463	-0.01821	32
% of intermediate (5-20% K2P) class I repeats	progenetic-miniature vs non-miniature	9.4273	2.9141	2.4334	1.1976	0.2404622	0.04562	0.01381	32
% of intermediate (5-20% K2P) class II repeats	progenetic-miniature vs non-miniature	7.4549	8.9667	3.9378	2.2771	0.03008	0.1474	0.1189	32
% of intermediate (5-20% K2P) other repeats	progenetic-miniature vs non-miniature	2.88117	-1.51244	0.55555	-2.7224	0.01069	0.1981	0.1714	32
% of intermediate (5-20% K2P) LINEs	progenetic-miniature vs non-miniature	4.49021	-1.66486	0.91353	-1.8224	0.07837	0.09967	0.06966	32
% of intermediate (5-20% K2P) SINEs	progenetic-miniature vs non-miniature	0.34258	0.37487	0.86132	0.4352	0.6665	0.006274	-0.02685	32
% of intermediate (5-20% K2P) LTR	progenetic-miniature vs non-miniature	3.9955	4.7864	2.0328	2.3546	0.02528	0.156	0.1278	32
% of intermediate (5-20% K2P) DNA transposons	progenetic-miniature vs non-miniature	6.007	6.2083	3.0051	2.0659	0.04757	0.1245	0.09537	32
% of intermediate (5-20% K2P) helitrons	progenetic-miniature vs non-miniature	1.4023	2.7454	1.3089	2.0976	0.04447	0.1279	0.09883	32
% of ancient (>20% K2P) class I repeats	progenetic-miniature vs non-miniature	4.813	1.4553	1.2054	1.2073	0.2367607	0.04633	0.01454	32
% of ancient (>20% K2P) class II repeats	progenetic-miniature vs non-miniature	7.12106	0.19242	1.97792	0.0973	0.92315	0.0003154	-0.03301	32
% of ancient (>20% K2P) other repeats	progenetic-miniature vs non-miniature	0.74272	-0.22477	0.20375	-1.1032	0.2787151	0.03899	0.006952	32
% of ancient (>20% K2P) LINEs	progenetic-miniature vs non-miniature	1.78139	-0.42015	0.40631	-1.0341	0.3094	0.03442	0.002231	32
% of ancient (>20% K2P) SINEs	progenetic-miniature vs non-miniature	0.251787	0.026861	0.362754	0.074	0.9415	0.0001827	-0.03314	32
% of ancient (>20% K2P) LTR	progenetic-miniature vs non-miniature	2.7197	1.937	1.0832	1.7882	0.08385	0.09632	0.0662	32
% of ancient (>20% K2P) DNA transposons	progenetic-miniature vs non-miniature	5.30488	0.14979	1.54611	0.0969	0.92346	0.0003128	-0.03301	32
% of ancient (>20% K2P) helitrons	progenetic-miniature vs non-miniature	1.7965	0.12188	0.72592	0.1679	0.86779	0.0009388	-0.03236	32

Table S7. Comparison of Brownian motion (BM) and Ornstein–Uhlenbeck (OU) model fit within the phylogenetic generalized least squares (PGLS) regression framework for transposable element content and gene architecture traits across Cypriniformes.

		Brownian motion model			Ornstein Uhlenbeck model			
**Trait**	**Predictor**	**BM_log likelihood**	**BM_AIC**	**BM_AICw**	**OU_log likelihood**	**OU_AIC**	**OU_AICw**	**delta AIC**
median gene length	miniature vs non-miniature	-299.7	605.4	0.09766831	-296.5	601	0.90233169	4.44681
overall median intron length	miniature vs non-miniature	-202.1	410.1	0.94325102	-203.9	415.8	0.05674898	5.621389
first (5`) median intron length	miniature vs non-miniature	-219.7	445.5	0.91786383	-221.2	450.3	0.08213617	4.827341
last (3`) median intron length	miniature vs non-miniature	-229.3	464.7	0.8139695	-229.8	467.6	0.1860305	2.952024
mid median intron length	miniature vs non-miniature	-195.3	396.6	0.95003862	-197.2	402.5	0.04996138	5.890505
single median intron length	miniature vs non-miniature	-249.7	505.3	0.0155038	-244.5	497	0.9844962	8.302089
ultra-short introns (≤100 bp) median length	miniature vs non-miniature	-58.2	122.4	0.92875084	-59.76	127.53	0.07124916	5.135315
short introns (101–300 bp) median length	miniature vs non-miniature	-70.15	146.29	0.91029618	-71.46	150.93	0.08970382	4.634513
compact introns (≤300 bp) median length	miniature vs non-miniature	-95.35	196.7	0.04782785	-97.34	202.68	0.95217215	5.982275
long introns (>300 bp) median length	miniature vs non-miniature	-221.6	449.3	0.93274375	-223.3	454.6	0.06725625	5.259241
first (5`) ultra-short introns (≤100 bp) median intron length	miniature vs non-miniature	-77.05	160.1	7.13E-05	-66.5	141	1.00E+00	19.09733
last (3`) ultra-short introns (≤100 bp) median intron length	miniature vs non-miniature	-63.4	132.8	0.92410055	-64.9	137.8	0.07589945	4.998823
mid ultra-short introns (≤100 bp) median intron length	miniature vs non-miniature	-58.09	122.17	0.92929131	-59.66	127.33	0.07070869	5.151708
single ultra-short introns (≤100 bp) median intron length	miniature vs non-miniature	-134.3	274.6	9.18E-12	-107.9	223.8	1.00E+00	50.82785
first (5`) short introns (101–300 bp) median intron length	miniature vs non-miniature	-112.2	230.3	0.00037479	-103.3	214.5	0.99962521	15.77754
last (3`) short introns (101–300 bp) median intron length	miniature vs non-miniature	-87.14	180.28	0.4867711	-86.09	180.18	0.5132289	0.1058561
mid short introns (101–300 bp) median intron length	miniature vs non-miniature	-67.99	141.99	0.9277965	-69.55	147.09	0.0722035	5.106648
single short introns (101–300 bp) median intron length	miniature vs non-miniature	-137.4	280.7	7.54E-06	-124.6	257.1	1.00E+00	23.59124
first (5`) compact introns (≤300 bp) median intron length	miniature vs non-miniature	-129.1	264.3	0.008993164	-123.4	254.9	0.991006836	9.404513
last (3`) compact introns (≤300 bp) median intron length	miniature vs non-miniature	-106	218	0.93441453	-107.7	223.3	0.06558547	5.313132
mid compact introns (≤300 bp) median intron length	miniature vs non-miniature	-94.18	194.36	0.95174567	-96.16	200.32	0.04825433	5.963625
single compact introns (≤300 bp) median intron length	miniature vs non-miniature	-144.9	295.7	7.09E-06	-132	272	1.00E+00	23.71445
first (5`) long introns (>300 bp) median intron length	miniature vs non-miniature	-229.5	465	0.91781899	-230.9	469.8	0.08218101	4.826152
last (3`) long introns (>300 bp) median intron length	miniature vs non-miniature	-230.3	466.6	0.92805717	-231.8	471.7	0.07194283	5.114443
mid long introns (>300 bp) median intron length	miniature vs non-miniature	-220.2	446.5	0.93300027	-221.9	451.7	0.06699973	5.267434
single long introns (>300 bp) median intron length	miniature vs non-miniature	-247.1	500.2	0.07861309	-243.6	495.3	0.92138691	4.922684
median number of introns per gene	miniature vs non-miniature	-47.84	101.69	1.73E-09	-26.67	61.34	1.00E+00	40.35381
median number of mid introns per gene	miniature vs non-miniature	-36.57	79.14	2.56698E-05	-25	58	0.99997433	21.14034
proportion of compact introns (≤300 bp) per gene	miniature vs non-miniature	69.81	-133.62	0.95169441	67.83	-127.66	0.04830559	5.961394
proportion of long introns (>300 bp) per gene	miniature vs non-miniature	69.81	-133.62	0.95169441	67.83	-127.66	0.04830559	5.961394
% of non-repititive DNA	miniature vs non-miniature	-115.5	236.9	0.7846175	-115.8	239.5	0.2153825	2.585561
% of total repeat DNA	miniature vs non-miniature	-115.5	236.9	0.7846175	-115.8	239.5	0.2153825	2.585561
% of class I repeats	miniature vs non-miniature	-109.6	225.3	0.003988913	-103.1	214.2	0.996011087	11.04048
% of class II repeats	miniature vs non-miniature	-106.9	219.8	0.50314	-105.9	219.8	0.49686	0.0251203

% of other repeats	miniature vs non-miniature	-50.17	106.33	0.91887745	-51.59	111.19	0.08112255	4.854383
% of retroelements	miniature vs non-miniature	-109.6	225.3	0.003988913	-103.1	214.2	0.996011087	11.04048
% of total interspersed repeats	miniature vs non-miniature	-115.7	237.3	0.8223273	-116.2	240.4	0.1776727	3.06439
% of LINEs	miniature vs non-miniature	-88.33	182.67	1.68E-06	-74.04	156.07	1.00E+00	26.59604
% of SINEs	miniature vs non-miniature	-54.57	115.14	0.8358171	-55.2	118.4	0.1641829	3.254858
% of LTR	miniature vs non-miniature	-99.41	204.83	0.07521554	-95.91	199.81	0.92478446	5.018406
% of DNA transposons	miniature vs non-miniature	-106.9	219.8	0.5034622	-105.9	219.8	0.4965378	0.02769818
% of helitrons	miniature vs non-miniature	89.79	-173.59	0.90293485	88.56	-169.13	0.09706515	4.460536
% of small RNA	miniature vs non-miniature	17.09	-28.18	0.0249112	21.75	-35.51	0.9750888	7.334422
% of satellites	miniature vs non-miniature	280.1	-554.2	0.90748221	278.8	-549.7	0.09251779	4.566546
% of simple repeats	miniature vs non-miniature	-36.96	79.93	0.94514209	-38.81	85.62	0.05485791	5.693178
% of low complexity repeats	miniature vs non-miniature	43.37	-80.75	0.9592773	41.22	-74.43	0.0407227	6.318789
% of unclassified repeats	miniature vs non-miniature	-30.76	67.52	0.7439525	-30.82	69.65	0.2560475	2.133228
% of recent (<5% K2P) class I repeats	miniature vs non-miniature	-83.99	173.98	0.001168581	-76.24	160.48	0.998831419	13.50159
% of recent (<5% K2P) class II repeats	miniature vs non-miniature	-72.8	151.6	0.2876433	-70.9	149.8	0.7123567	1.813716
% of recent (<5% K2P) other repeats	miniature vs non-miniature	-32.68	71.35	0.8926718	-33.8	75.59	0.1073282	4.236655
% of recent (<5% K2P) LINEs	miniature vs non-miniature	-54.52	115.04	0.01271682	-49.17	106.33	0.98728318	8.704063
% of recent (<5% K2P) SINEs	miniature vs non-miniature	7.837	-9.675	0.818014	7.334	-6.669	0.181986	3.0059
% of recent (<5% K2P) LTR	miniature vs non-miniature	-75.93	157.85	0.0157162	-70.79	149.58	0.9842838	8.274444
% of recent (<5% K2P) DNA transposons	miniature vs non-miniature	-71.43	148.87	0.2091536	-69.1	146.2	0.7908464	2.66007
% of recent (<5% K2P) helitrons	miniature vs non-miniature	12.18	-18.36	0.7247393	12.21	-16.43	0.2752607	1.936187
% of intermediate (5-20% K2P) class I repeats	miniature vs non-miniature	-100.6	207.2	0.000431174	-91.83	191.67	0.999568827	15.49714
% of intermediate (5-20% K2P) class II repeats	miniature vs non-miniature	-99.45	204.89	0.5621819	-98.7	205.4	0.4378181	0.5000438
% of intermediate (5-20% K2P) other repeats	miniature vs non-miniature	-29.53	65.07	0.95146393	-31.51	71.02	0.04853607	5.951389
% of intermediate (5-20% K2P) LINEs	miniature vs non-miniature	-72.19	150.38	4.56E-06	-58.89	125.79	1.00E+00	24.59541
% of intermediate (5-20% K2P) SINEs	miniature vs non-miniature	-43.27	92.53	0.8948223	-44.41	96.81	0.1051777	4.281947
% of intermediate (5-20% K2P) LTR	miniature vs non-miniature	-96.14	198.29	0.000300827	-87.04	182.07	0.999699173	16.21735
% of intermediate (5-20% K2P) DNA transposons	miniature vs non-miniature	-89.01	184.02	0.6565485	-88.66	185.31	0.3434515	1.295902
% of intermediate (5-20% K2P) helitrons	miniature vs non-miniature	-68.74	143.48	0.1526958	-66.03	140.05	0.8473042	3.427225
% of ancient (>20% K2P) class I repeats	miniature vs non-miniature	-70.94	147.87	0.004949424	-64.63	137.27	0.995050576	10.60704
% of ancient (>20% K2P) class II repeats	miniature vs non-miniature	-71.87	149.75	0.861369	-72.7	153.4	0.138631	3.653414
% of ancient (>20% K2P) other repeats	miniature vs non-miniature	-5.334	16.667	0.1598745	-2.674	13.349	0.8401255	3.318324
% of ancient (>20% K2P) LINEs	miniature vs non-miniature	-42.6	91.2	2.79081E-05	-31.11	70.22	0.999972092	20.97313
% of ancient (>20% K2P) SINEs	miniature vs non-miniature	-24.62	55.24	0.107492	-21.5	51	0.892508	4.233239
% of ancient (>20% K2P) LTR	miniature vs non-miniature	-62.05	130.09	0.1137902	-58.99	125.99	0.8862098	4.105195
% of ancient (>20% K2P) DNA transposons	miniature vs non-miniature	-63.18	132.36	0.8871803	-64.24	136.48	0.1128197	4.124514
% of ancient (>20% K2P) helitrons	miniature vs non-miniature	-47.85	101.7	0.1067453	-44.73	97.45	0.8932547	4.248853

median gene length	progenetic-miniature vs non-miniature	-291	588.1	0.1024631	-287.9	583.7	0.8975369	4.340303
overall median intron length	progenetic-miniature vs non-miniature	-196.4	398.8	0.94184815	-198.2	404.3	0.05815185	5.569573
first (5`) median intron length	progenetic-miniature vs non-miniature	-213.4	432.7	0.91673511	-214.8	437.5	0.08326489	4.797583
last (3`) median intron length	progenetic-miniature vs non-miniature	-222.6	451.2	0.8193098	-223.1	454.2	0.1806902	3.023357
mid median intron length	progenetic-miniature vs non-miniature	-189.8	385.6	0.94895375	-191.7	391.4	0.05104625	5.845256
single median intron length	progenetic-miniature vs non-miniature	-242	490.1	0.02861799	-237.5	483	0.97138201	7.049368
ultra-short introns (≤100 bp) median length	progenetic-miniature vs non-miniature	-47.51	101.03	0.93017909	-49.1	106.2	0.06982091	5.178887
short introns (101–300 bp) median length	progenetic-miniature vs non-miniature	-67.53	141.06	0.91001468	-68.84	145.69	0.08998532	4.627628
compact introns (≤300 bp) median length	progenetic-miniature vs non-miniature	-85.01	176.03	0.9594366	-87.18	182.35	0.0405634	6.32696
long introns (>300 bp) median length	progenetic-miniature vs non-miniature	-215.4	436.7	0.93124272	-217	441.9	0.06875728	5.211875
first (5`) ultra-short introns (≤100 bp) median intron length	progenetic-miniature vs non-miniature	-73.56	153.12	5.44E-06	-60.44	128.88	1.00E+00	24.24284
last (3`) ultra-short introns (≤100 bp) median intron length	progenetic-miniature vs non-miniature	-51.43	108.87	0.92280647	-52.91	113.83	0.07719353	4.962208
mid ultra-short introns (≤100 bp) median intron length	progenetic-miniature vs non-miniature	-47.29	100.58	0.93115921	-48.9	105.8	0.06884079	5.209268
single ultra-short introns (≤100 bp) median intron length	progenetic-miniature vs non-miniature	-130.6	267.2	1.09E-11	-104.4	216.7	1.00E+00	50.48064
first (5`) short introns (101–300 bp) median intron length	progenetic-miniature vs non-miniature	-109	224	0.000553997	-100.5	209	0.999446004	14.9956
last (3`) short introns (101–300 bp) median intron length	progenetic-miniature vs non-miniature	-84.89	175.77	0.472501	-83.78	175.55	0.527499	0.2202141
mid short introns (101–300 bp) median intron length	progenetic-miniature vs non-miniature	-65.73	137.47	0.92710669	-67.28	142.55	0.07289331	5.086144
single short introns (101–300 bp) median intron length	progenetic-miniature vs non-miniature	-132.1	270.3	5.78E-07	-116.8	241.6	1.00E+00	28.72754
first (5`) compact introns (≤300 bp) median intron length	progenetic-miniature vs non-miniature	-123.9	253.7	0.02798835	-119.3	246.6	0.97201165	7.095159
last (3`) compact introns (≤300 bp) median intron length	progenetic-miniature vs non-miniature	-99.24	204.49	0.93538639	-100.9	209.8	0.06461361	5.345069
mid compact introns (≤300 bp) median intron length	progenetic-miniature vs non-miniature	-84.7	175.4	0.95799469	-86.83	181.66	0.04200531	6.254092
single compact introns (≤300 bp) median intron length	progenetic-miniature vs non-miniature	-139	284	8.47E-07	-124	256.1	1.00E+00	27.96224
first (5`) long introns (>300 bp) median intron length	progenetic-miniature vs non-miniature	-222.8	451.7	0.91679712	-224.2	456.5	0.08320288	4.799209
last (3`) long introns (>300 bp) median intron length	progenetic-miniature vs non-miniature	-223.6	453.3	0.92732428	-225.2	458.4	0.07267572	5.092592
mid long introns (>300 bp) median intron length	progenetic-miniature vs non-miniature	-214	434	0.93117813	-215.6	439.2	0.06882187	5.209858
single long introns (>300 bp) median intron length	progenetic-miniature vs non-miniature	-239.9	485.7	0.09495646	-236.6	481.2	0.90504354	4.509129
median number of introns per gene	progenetic-miniature vs non-miniature	-46.57	99.13	2.59E-09	-25.79	59.59	1.00E+00	39.54329
median number of mid introns per gene	progenetic-miniature vs non-miniature	-35.9	77.8	3.26E-05	-24.57	57.14	1.00E+00	20.6641
proportion of compact introns (≤300 bp) per gene	progenetic-miniature vs non-miniature	67.35	-128.7	0.95066866	65.39	-122.79	0.04933134	5.917212
proportion of long introns (>300 bp) per gene	progenetic-miniature vs non-miniature	67.35	-128.7	0.95066866	65.39	-122.79	0.04933134	5.917212
% of non-repititive DNA	progenetic-miniature vs non-miniature	-112	230	0.7581424	-112.1	232.3	0.2418576	2.285045
% of total repeat DNA	progenetic-miniature vs non-miniature	-112	230	0.7581424	-112.1	232.3	0.2418576	2.285045
% of class I repeats	progenetic-miniature vs non-miniature	-106.2	218.3	0.002958534	-99.34	206.68	0.997041466	11.6402
% of class II repeats	progenetic-miniature vs non-miniature	-104	214.1	0.4646398	-102.9	213.8	0.5353602	0.283355
% of other repeats	progenetic-miniature vs non-miniature	-46.62	99.25	0.91943902	-48.06	104.12	0.08056098	4.869499
% of retroelements	progenetic-miniature vs non-miniature	-106.2	218.3	0.002958534	-99.34	206.68	0.997041466	11.6402
% of total interspersed repeats	progenetic-miniature vs non-miniature	-112	230	0.8024675	-112.4	232.8	0.1975325	2.803576

% of LINEs	progenetic-miniature vs non-miniature	-86.03	178.06	2.09E-06	-71.96	151.91	1.00E+00	26.15255
% of SINEs	progenetic-miniature vs non-miniature	-53.32	112.64	0.8341534	-53.94	115.88	0.1658466	3.230708
% of LTR	progenetic-miniature vs non-miniature	-95.39	196.78	0.02765023	-90.83	189.66	0.97234977	7.120163
% of DNA transposons	progenetic-miniature vs non-miniature	-104	214.1	0.4647575	-102.9	213.8	0.5352425	0.2824083
% of helitrons	progenetic-miniature vs non-miniature	87.93	-169.87	0.91362301	86.57	-165.15	0.08637699	4.717393
% of small RNA	progenetic-miniature vs non-miniature	16.14	-26.29	0.03003646	20.62	-33.24	0.96996354	6.949693
% of satellites	progenetic-miniature vs non-miniature	357.4	-708.9	0.94785615	355.5	-703.1	0.05214385	5.800393
% of simple repeats	progenetic-miniature vs non-miniature	-34.14	74.27	0.94847113	-36.05	80.1	0.05152887	5.825418
% of low complexity repeats	progenetic-miniature vs non-miniature	42.91	-79.82	0.96006961	40.73	-73.46	0.03993039	6.359736
% of unclassified repeats	progenetic-miniature vs non-miniature	-29.4	64.8	0.6843999	-29.17	66.34	0.3156001	1.548133
% of recent (<5% K2P) class I repeats	progenetic-miniature vs non-miniature	-81.46	168.92	0.000747826	-73.26	154.52	0.999252174	14.39519
% of recent (<5% K2P) class II repeats	progenetic-miniature vs non-miniature	-70.92	147.84	0.1523485	-68.2	144.4	0.8476515	3.432598
% of recent (<5% K2P) other repeats	progenetic-miniature vs non-miniature	-27.29	60.58	0.93885055	-29.02	66.04	0.06114945	5.462671
% of recent (<5% K2P) LINEs	progenetic-miniature vs non-miniature	-52.84	111.69	0.0116678	-47.4	102.8	0.9883322	8.878373
% of recent (<5% K2P) SINEs	progenetic-miniature vs non-miniature	7.501	-9.001	0.8305054	6.911	-5.823	0.1694946	3.178426
% of recent (<5% K2P) LTR	progenetic-miniature vs non-miniature	-72.72	151.43	0.004685838	-66.36	140.71	0.995314162	10.71703
% of recent (<5% K2P) DNA transposons	progenetic-miniature vs non-miniature	-69.62	145.24	0.1055837	-66.49	140.97	0.8944163	4.273336
% of recent (<5% K2P) helitrons	progenetic-miniature vs non-miniature	11.97	-17.93	0.5583498	12.73	-17.46	0.4416502	0.468935
% of intermediate (5-20% K2P) class I repeats	progenetic-miniature vs non-miniature	-97.67	201.34	0.000368204	-88.77	185.53	0.999631796	15.81301
% of intermediate (5-20% K2P) class II repeats	progenetic-miniature vs non-miniature	-96.72	199.44	0.5151211	-95.78	199.56	0.4848789	0.1210056
% of intermediate (5-20% K2P) other repeats	progenetic-miniature vs non-miniature	-26.82	59.65	0.94707365	-28.71	65.41	0.05292635	5.768951
% of intermediate (5-20% K2P) LINEs	progenetic-miniature vs non-miniature	-70.37	146.74	4.88E-06	-57.14	122.28	1.00E+00	24.46034
% of intermediate (5-20% K2P) SINEs	progenetic-miniature vs non-miniature	-42.26	90.53	0.8967545	-43.43	94.85	0.1032455	4.323344
% of intermediate (5-20% K2P) LTR	progenetic-miniature vs non-miniature	-93.2	192.4	0.000102334	-83.01	174.02	0.999897667	18.37434
% of intermediate (5-20% K2P) DNA transposons	progenetic-miniature vs non-miniature	-86.72	179.44	0.6379921	-86.29	180.58	0.3620079	1.133319
% of intermediate (5-20% K2P) helitrons	progenetic-miniature vs non-miniature	-66.64	139.28	0.08574702	-63.27	134.55	0.91425298	4.733412
% of ancient (>20% K2P) class I repeats	progenetic-miniature vs non-miniature	-69.19	144.37	0.00580287	-63.04	134.09	0.99419713	10.28717
% of ancient (>20% K2P) class II repeats	progenetic-miniature vs non-miniature	-69.95	145.91	0.8495341	-70.69	149.37	0.1504659	3.461903
% of ancient (>20% K2P) other repeats	progenetic-miniature vs non-miniature	-5.607	17.214	0.1432287	-2.818	13.637	0.8567713	3.577457
% of ancient (>20% K2P) LINEs	progenetic-miniature vs non-miniature	-41.61	89.22	2.95608E-05	-30.18	68.36	0.999970439	20.85806
% of ancient (>20% K2P) SINEs	progenetic-miniature vs non-miniature	-24.31	54.62	0.1071604	-21.19	50.38	0.8928396	4.240161
% of ancient (>20% K2P) LTR	progenetic-miniature vs non-miniature	-60.39	126.79	0.1021285	-57.22	122.44	0.8978715	4.34759
% of ancient (>20% K2P) DNA transposons	progenetic-miniature vs non-miniature	-61.43	128.86	0.878801	-62.41	132.82	0.121199	3.962249
% of ancient (>20% K2P) helitrons	progenetic-miniature vs non-miniature	-46.84	99.68	0.1042534	-43.69	95.38	0.8957466	4.301667

Table S8. Effective sample sizes (ESS) for parameters in the BEAST divergence-time analysis, combined across all post–burn-in samples.

Summary Statistic	mean	stderr of mean	stdev	variance	median	value range	geometric mean	95% HPD interval	auto-correlation time (ACT)	effective sample size (ESS)	number of samples
posterior	-1521974.118	1.3635	38.5103	1483.0431	-1521973.634	[-1522133.4057, -1521823.6343]	n/a	[-1522051.0726, -1521900.0493]	360610	797.7	28765
likelihood	-1520211.275	0.2311	18.2546	333.2293	-1520210.837	[-1520294.9687, -1520141.8657]	n/a	[-1520247.3357, -1520176.2081]	46118.8304	6237.1	28765
prior	-1762.8429	1.3919	34.1725	1167.7568	-1762.1265	[-1903.4081, -1617.9783]	n/a	[-1829.7323, -1695.89]	477210	602.8	28765
treeLikelihood.reduced	-1520211.275	0.2311	18.2546	333.2293	-1520210.837	[-1520294.9687, -1520141.8657]	n/a	[-1520247.3357, -1520176.2081]	46118.8304	6237.1	28765
Tree.height	199.32	0.0921	7.6957	59.2233	199.1388	[170.9319, 234.4561]	199.1717	[184.4577, 214.4252]	41180.1431	6985.2	28765
Tree.treeLength	7851.3554	24.9226	563.8691	317950	7830.2705	[5935.1062, 10429.8055]	7831.1741	[6730.2609, 8943.791]	561970	511.9	28765
rateAC	0.3792	0.00019335	0.0041708	0.000017396	0.3792	[0.3633, 0.3972]	0.3792	[0.3708, 0.3871]	618180	465.3	28765
rateAG	1.1979	0.0011283	0.0153	0.00023331	1.1978	[1.1405, 1.2729]	1.1978	[1.1667, 1.2266]	1569600	183.3	28765
rateAT	0.3228	0.00015862	0.0036094	0.000013028	0.3228	[0.3085, 0.3373]	0.3228	[0.3158, 0.3299]	555540	517.8	28765
rateCG	0.4029	0.00032919	0.0045881	0.00002105	0.4029	[0.3826, 0.4209]	0.4029	[0.394, 0.4118]	1480800	194.2	28765
rateGT	0.3107	0.00023575	0.0036524	0.00001334	0.3107	[0.2957, 0.326]	0.3107	[0.3035, 0.3177]	1198400	240	28765
freqParameter.1	0.2714	0.000054385	0.0013456	1.8106E-06	0.2715	[0.2661, 0.2771]	0.2714	[0.2688, 0.2741]	469920	612.1	28765
freqParameter.2	0.2386	0.000031725	0.0012612	1.5907E-06	0.2386	[0.234, 0.244]	0.2386	[0.2361, 0.2411]	182010	1580.4	28765
freqParameter.3	0.2211	0.000081619	0.0012212	1.4913E-06	0.2211	[0.2162, 0.226]	0.2211	[0.2186, 0.2234]	1285000	223.9	28765
freqParameter.4	0.2688	0.000056669	0.0013958	1.9482E-06	0.2688	[0.2634, 0.2748]	0.2688	[0.2662, 0.2716]	474170	606.6	28765
gammaShape	0.4467	0.000019487	0.0025924	6.7208E-06	0.4467	[0.4363, 0.4574]	0.4467	[0.4415, 0.4516]	16253.7899	17697.4	28765
ORCsigma	0.4378	0.00060827	0.0176	0.00031094	0.4373	[0.3798, 0.521]	0.4375	[0.4045, 0.4738]	342290	840.4	28765
ORCRatesStat.mean	0.0009844	3.1513E-06	0.000070907	5.0278E-09	0.00098207	[7.3544E-4, 1.2893E-3]	0.00098186	[8.4688E-4, 1.1236E-3]	568180	506.3	28765
ORCRatesStat.variance	1.9977E-07	1.4434E-09	3.3818E-08	1.1436E-15	1.9616E-07	[1.029E-7, 4.2274E-7]	1.9703E-07	[1.3922E-7, 2.6772E-7]	524020	548.9	28765
ORCRatesStat.coefficientOfVariation	0.4758	0.00058931	0.0192	0.00037037	0.4744	[0.4197, 0.5995]	0.4754	[0.4408, 0.5151]	269730	1066.4	28765
ORCucldMean	0.00093566	3.7821E-06	0.000078481	6.1593E-09	0.00093184	[6.8741E-4, 1.303E-3]	0.0009324	[7.8975E-4, 1.0951E-3]	668050	430.6	28765
BirthDeath	-1387.8671	1.0997	24.3892	594.8317	-1387.8967	[-1483.5629, -1292.8791]	n/a	[-1437.4993, -1341.6703]	584810	491.9	28765
BDBirthRate	0.0378	0.000064313	0.0044197	0.000019534	0.0378	[0.015, 0.0568]	0.0375	[0.0291, 0.0465]	60909.8738	4722.6	28765
BDDeathRate	0.1933	0.0036203	0.1229	0.0151	0.1795	[1.1742E-5, 0.7077]	0.1406	[1.8335E-5, 0.4147]	249600	1152.4	28765
monophyletic(cyprinoidei)	1	0	0	0	0	[1, 1]	n/a	n/a	n/a	n/a	28765
logP(mrca(cyprinoidei))	-5.3132	0	0	0	0	[-5.3132, -5.3132]	n/a	n/a	n/a	n/a	28765
mrca.age(cyprinoidei.originate)	101.4448	0.352	9.6148	92.4442	101.0787	[69.2153, 144.0964]	100.9904	[83.1644, 120.4467]	385560	746.1	28765
monophyletic(catostomoidei)	1	0	0	0	0	[1, 1]	n/a	n/a	n/a	n/a	28765
logP(mrca(catostomoidei))	-5.3093	0	0	0	0	[-5.3093, -5.3093]	n/a	n/a	n/a	n/a	28765
mrca.age(catostomoidei.originate)	98.157	0.3413	9.4286	88.8985	97.7761	[65.8591, 139.907]	97.7058	[81.015, 117.6347]	376920	763.2	28765
monophyletic(cobitidae)	1	0	0	0	0	[1, 1]	n/a	n/a	n/a	n/a	28765
logP(mrca(cobitidae))	-5.4022	0	0	0	0	[-5.4022, -5.4022]	n/a	n/a	n/a	n/a	28765
mrca.age(cobitidae.originate)	67.0794	0.2257	6.5921	43.4562	66.759	[45.0783, 102.8913]	66.7575	[54.6485, 80.1682]	337340	852.7	28765
monophyletic(all)	0	0	0	0	0	[0, 0]	n/a	n/a	n/a	n/a	28765
logP(mrca(all))	-3.7349	0.0113	1.0051	1.0101	-3.3529	[-14.7724, -2.9637]	n/a	[-5.7932, -2.9637]	36568.1792	7866.1	28765
mrca.age(all)	199.32	0.0921	7.6957	59.2233	199.1388	[170.9319, 234.4561]	199.1717	[184.4577, 214.4252]	41180.1431	6985.2	28765
monophyletic(ictalurus)	1	0	0	0	0	[1, 1]	n/a	n/a	n/a	n/a	28765
logP(mrca(ictalurus))	-5.3181	0	0	0	0	[-5.3181, -5.3181]	n/a	n/a	n/a	n/a	28765
mrca.age(ictalurus.originate)	48.1805	0.0227	2.1032	4.4233	47.5626	[46, 67.2593]	48.1368	[46, 52.3861]	33366.0058	8621	28765
monophyletic(gonorhynchiformes)	1	0	0	0	0	[1, 1]	n/a	n/a	n/a	n/a	28765
logP(mrca(gonorhynchiformes))	-4.7656	0	0	0	0	[-4.7656, -4.7656]	n/a	n/a	n/a	n/a	28765
mrca.age(gonorhynchiformes.originate)	193.2839	0.1247	8.991	80.8377	193.4194	[152.9772, 229.3568]	193.0734	[175.3493, 210.833]	55319.7661	5199.8	28765

Table S9. Species excluded from downstream analyses.

Voucher_reads	Genus	Species	Remarks
LR15244	*Catostomus*	*commersoni*	poor model fit in kmer profiling; genome size estimates do not converge
LR15246	*Ictiobus*	*bubalus*	high read error and poor model fit in kmer profiling; genome size estimates has a % diff > 10% between k=21 and k=31 models
LR15340	*Acantopsis*	sp	high read error and poor model fit in kmer profiling; genome size estimates has a % diff > 10% between k=21 and k=31 models
KUFOS-AN-2019.3.1	*Eechathalakenda*	*ophicephalus*	high read error and poor model fit in kmer profiling; genome size estimates has a % diff > 10% between k=21 and k=31 models
LR15296	*Enteromius*	sp	high read error and poor model fit in kmer profiling; genome size estimates has a % diff > 10% between k=21 and k=31 models; unrealistic high level of heterozygozity
KUFOS-AN-2021.7.1	*Neolissochilus*	*wynaadensis*	high read error and poor model fit in kmer profiling; genome size estimates has a % diff > 10% between k=21 and k=31 models
LR15108	*Puntioplites*	*bulu*	high read error and poor model fit in kmer profiling; genome size estimates has a % diff > 10% between k=21 and k=31 models
LR15100	*Tor*	*tambroides*	poor model fit in kmer profiling; genome size estimates do not converge
LR14717	*Devario*	sp	high read error and poor model fit in kmer profiling; genome size estimates has a % diff > 10% between k=21 and k=31 models; unrealistic high level of heterozygozity
LR12807	*Pectenocypris*	sp	generic ID doubtful; probable sample contamination with Rasbora and Pectenocypris
LR10789	*Rasbora*	*einthovenii*	high read error and poor model fit in kmer profiling; genome size estimates has a % diff > 10% between k=21 and k=31 models; unrealistic high level of heterozygozity
129LR02128	*Salmostoma*	sp	generic ID doubtful: probable library contamination with Salmostoma and Mastacembelus
1736_2012	*Gobio*	*gobio*	high read error and poor model fit in kmer profiling; genome size estimates has a % diff > 10% between k=21 and k=31 models; unrealistic high level of heterozygozity
117LR15718	*Aphyocypris*	*chinensis*	probable hybrid sample of *Nipponocypris* X *Aphyocypris*

Table S10. Genome size estimates for species included in the k-mer profiling dataset (n = 261).

Voucher_reads	Voucher_original	BioProject	BioSample	Accession	Suborder	Family	Genus	Species	mean_k21_genomesize_MB	mean_k31_genomesize_MB	mean_GC_content	mean_repeat_content_k31	mean_unique_content_k31	Ploidy	Miniature
LR15247	TCWC 19725.02	PRJNA1483025	SAMN61141513	SRR39401240	Catostomoidei	Catostomidae	*Cycleptus*	sp	2473.239595	2509.817213	38.51	38.079649	61.920351	polyploid	non-miniature
LR15245	TCWC 20294.01	PRJNA1483025	SAMN61141514	SRR39401184	Catostomoidei	Catostomidae	*Erimyzon*	*sucetta*	1830.213582	1788.172173	37.68	14.58509748	85.41490252	polyploid	non-miniature
LR15248	TCWC 20297.11	PRJNA1483025	SAMN61141516	SRR39401167	Catostomoidei	Catostomidae	*Moxostoma*	*congestum*	2066.817793	2017.530905	38.525	22.03797974	77.96202026	polyploid	non-miniature
LR15059	LR15059	PRJNA1483025	SAMN61141517	SRR39401083	Catostomoidei	Catostomidae	*Myxocyprinus*	*asiaticus*	2354.006876	2309.856512	38.2	31.17281062	68.8271894	polyploid	non-miniature
LR15354	LR15354	PRJNA1483025	SAMN61141518	SRR39401208	Cobitoidei	Balitoridae	*Balitora*	sp	490.2965405	492.1509565	37.65	18.1114232	81.8885767	diploid	non-miniature
LR15094	LR15094	PRJNA1483025	SAMN61141519	SRR39401044	Cobitoidei	Balitoridae	*Balitoropsis*	*zollingeri*	415.8431725	416.506414	38.46	15.13223984	84.86776016	diploid	non-miniature
KUFOS-AN-2019.1.1	KUFOS-AN-2019.1.1	PRJNA1393636	SAMN54310100	SRR36627411	Cobitoidei	Balitoridae	*Bhavania*	*australis*	432.301652	432.345153	38.275	18.46152696	81.53847315	diploid	non-miniature
LR15176	LR15176	PRJNA1483025	SAMN61141520	SRR39401148	Cobitoidei	Balitoridae	*Hemimyzon*	*formosanum*	507.478481	509.5217015	37.76	22.76720366	77.23279624	diploid	non-miniature
LR15139	LR15139	PRJNA1483025	SAMN61141521	SRR39401075	Cobitoidei	Balitoridae	*Homaloptera*	*orthogoniata*	448.5771285	450.377603	38.215	14.78019756	85.21980244	diploid	non-miniature
LR15140	LR15140	PRJNA1483025	SAMN61141522	SRR39401239	Cobitoidei	Balitoridae	*Homalopteroides*	*stephensoni*	487.738089	488.628322	37.64	22.49866935	77.50133075	diploid	non-miniature
LR15093	LR15093	PRJNA1483025	SAMN61141523	SRR39401159	Cobitoidei	Balitoridae	*Homalopteroides*	*tweediei*	416.690849	413.243012	38.08	24.61690508	75.38309492	diploid	proportioned-dwarf
LR15091	LR15091	PRJNA1483025	SAMN61141524	SRR39401228	Cobitoidei	Balitoridae	*Homalopterula*	*gymnogaster*	349.2578485	347.959084	37.37	23.46332924	76.5366709	diploid	non-miniature
LR15172	LR15172	PRJNA1483025	SAMN61141525	SRR39401156	Cobitoidei	Balitoridae	*Lepturichthys*	*fimbriatus*	530.576996	530.93438	38.05	23.39528192	76.60471818	diploid	non-miniature
LR15487	LR15487	PRJNA1483025	SAMN61141526	SRR39401119	Cobitoidei	Balitoridae	*Neohomaloptera*	*johorensis*	359.387026	357.9961275	38.265	12.34288086	87.65711928	diploid	proportioned-dwarf
LR15088	LR15088	PRJNA1483025	SAMN61141527	SRR39401128	Cobitoidei	Balitoridae	*Sinogastromyzon*	*puliensis*	536.854406	537.5911635	37.935	25.47440458	74.52559542	diploid	non-miniature
LR15181	LR15181	PRJNA1483025	SAMN61141528	SRR39401117	Cobitoidei	Balitoridae	*Travancoria*	*elongata*	415.421302	419.2010845	37.91	15.94507755	84.05492245	diploid	non-miniature
LR15360	LR15360	PRJNA1483025	SAMN61141529	SRR39401194	Cobitoidei	Barbuccidae	*Barbucca*	*elongatus*	379.940554	379.578087	39.135	15.94304494	84.05695493	diploid	proportioned-dwarf
LR15330	LR15330	PRJNA1483025	SAMN61141530	SRR39401255	Cobitoidei	Botiidae	*Ambastaia*	*sidthimunki*	729.2012915	728.1004125	38.66	15.52128856	84.47871151	polyploid	non-miniature
LR15149	LR15149	PRJNA1483025	SAMN61141531	SRR39401107	Cobitoidei	Botiidae	*Botia*	*kubotai*	820.856938	820.033105	39.225	18.23438853	81.76561159	polyploid	non-miniature
LR15148	LR15148	PRJNA1483025	SAMN61141532	SRR39401183	Cobitoidei	Botiidae	*Chromobotia*	*macracanthus*	822.221016	821.307172	38.45	16.67373349	83.32626651	polyploid	non-miniature
LR15074	LR15074	PRJNA1483025	SAMN61141533	SRR39401067	Cobitoidei	Botiidae	*Leptobotia*	*elongata*	628.959658	626.037003	38.935	24.5738555	75.4261445	diploid	non-miniature
112LR15712	LR15712	PRJNA1483025	SAMN61141534	SRR39401246	Cobitoidei	Botiidae	*Parabotia*	*bimaculatus*	617.668014	615.909513	38.935	25.02578134	74.97421882	diploid	non-miniature
LR15151	LR15151	PRJNA1483025	SAMN61141535	SRR39401097	Cobitoidei	Botiidae	*Sinibotia*	*robusta*	756.346688	755.627618	38.645	18.15028908	81.84971092	polyploid	non-miniature
LR15446	LR15446	PRJNA1483025	SAMN61141536	SRR39401095	Cobitoidei	Botiidae	*Syncrossus*	*berdmorei*	651.924117	650.9152395	39.13	15.2188136	84.78118632	polyploid	non-miniature
115LR15715	LR15715	PRJNA1483025	SAMN61141537	SRR39401094	Cobitoidei	Cobitidae	*Acanthopsoides*	*robertsi*	594.7010075	595.835145	37.105	17.83197221	82.16802779	diploid	non-miniature
LR15193	LR15193	PRJNA1483025	SAMN61141539	SRR39401092	Cobitoidei	Cobitidae	*Bibarba*	*bibarba*	527.501072	527.2919795	38.07	16.75059624	83.24940376	diploid	non-miniature
LR15575	LR15575	PRJNA1483025	SAMN61141540	SRR39401091	Cobitoidei	Cobitidae	*Canthophrys*	*gongota*	540.872888	540.609599	38.64	17.61239861	82.38760148	diploid	non-miniature
LR15465	LR15465	PRJNA1483025	SAMN61141541	SRR39401052	Cobitoidei	Cobitidae	*Cobitis*	*sinensis*	1609.234857	1612.45102	38.83	60.18439853	39.81560147	diploid	non-miniature
LR15170	LR15170	PRJNA1483025	SAMN61141542	SRR39401177	Cobitoidei	Cobitidae	*Kottelatlimia*	*katik*	1035.049959	1043.902574	36.82	46.70781289	53.29218706	diploid	proportioned-dwarf
114LR15714	LR15714	PRJNA1483025	SAMN61141543	SRR39401176	Cobitoidei	Cobitidae	*Kottelatlimia*	*pristes*	890.119866	892.8929045	36.94	33.61137405	66.38862595	diploid	non-miniature
LR15467	LR15467	PRJNA1483025	SAMN61141544	SRR39401175	Cobitoidei	Cobitidae	*Lepidocephalichthys*	sp	655.6040215	656.0133925	34.79	14.4345278	85.5654722	diploid	non-miniature
LR15162	LR15162	PRJNA1483025	SAMN61141545	SRR39401174	Cobitoidei	Cobitidae	*Misgurnus*	*anguillicaudatus*	1248.361327	1236.165002	37.66	46.71336343	53.28663653	diploid	non-miniature
BNHS-FWF-981	BNHS-FWF-981	PRJNA1393636	SAMN54310101	SRR36627410	Cobitoidei	Cobitidae	*Pangio*	*bhujia*	674.482895	676.7185705	38.1	35.36530117	64.63469891	diploid	proportioned-dwarf
LR15141	LR15141	PRJNA1483025	SAMN61141546	SRR39401173	Cobitoidei	Cobitidae	*Pangio*	*semicincta*	816.3844045	813.269303	37.92	34.49735954	65.50264046	diploid	non-miniature
LR15609	LR15609	PRJNA1483025	SAMN61141547	SRR39401172	Cobitoidei	Cobitidae	*Sabanejewia*	*balcanica*	1040.697371	1039.00824	38.29	45.31050021	54.68949984	diploid	non-miniature
LR15146	LR15146	PRJNA1483025	SAMN61141548	SRR39401171	Cobitoidei	Cobitidae	*Theriodes*	*sandakanensis*	487.1853845	487.8762655	38.185	21.5355918	78.46440821	diploid	non-miniature
141LR15748	LR15748	PRJNA1483025	SAMN61141549	SRR39401170	Cobitoidei	Ellopostomatidae	*Ellopostoma*	*mystax*	449.932183	452.711028	38.195	18.33655419	81.66344581	diploid	non-miniature
LR15180	LR15180	PRJNA1483025	SAMN61141550	SRR39401169	Cobitoidei	Gastromyzontidae	*Annamia*	*normani*	417.142881	417.169605	37.93	12.43813281	87.56186719	diploid	non-miniature
LR15085	LR15085	PRJNA1483025	SAMN61141551	SRR39401168	Cobitoidei	Gastromyzontidae	*Beaufortia*	*kweichowensis*	463.373696	462.685559	37.77	16.05823406	83.94176594	diploid	non-miniature
LR15491	LR15491	PRJNA1483025	SAMN61141552	SRR39401061	Cobitoidei	Gastromyzontidae	*Erromyzon*	*compactus*	446.403103	446.541696	38.14	16.69201682	83.30798307	diploid	non-miniature
LR15090	LR15090	PRJNA1483025	SAMN61141553	SRR39401060	Cobitoidei	Gastromyzontidae	*Glaniopsis*	*muliradiatus*	474.3838815	472.8609795	38.095	20.54425523	79.45574477	diploid	non-miniature
LR15133	LR15133	PRJNA1483025	SAMN61141554	SRR39401059	Cobitoidei	Gastromyzontidae	*Katibasia*	*insidiosa*	427.185856	427.0149425	38.52	13.04301957	86.95698043	diploid	non-miniature
LR15175	LR15175	PRJNA1483025	SAMN61141555	SRR39401058	Cobitoidei	Gastromyzontidae	*Liniparhomaloptera*	*disparis*	430.0967585	431.0346715	38.365	13.96482243	86.03517757	diploid	non-miniature

LR15476	LR15476	PRJNA1483025	SAMN61141556	SRR39401057	Cobitoidei	Gastromyzontidae	*Plesiomyzon*	*baotingensis*	429.1058805	428.9316375	37.52	14.89622761	85.10377228	diploid	non-miniature
LR15425	LR15425	PRJNA1483025	SAMN61141557	SRR39401056	Cobitoidei	Gastromyzontidae	*Protomyzon*	*borneensis*	416.279624	416.6464905	38.51	10.89620903	89.10379097	diploid	non-miniature
LR15067	LR15067	PRJNA1483025	SAMN61141558	SRR39401055	Cobitoidei	Gastromyzontidae	*Pseudogastromyzon*	sp	438.298321	438.344733	38.035	14.91140696	85.08859316	diploid	non-miniature
LR15561	LR15561	PRJNA1483025	SAMN61141559	SRR39401054	Cobitoidei	Gastromyzontidae	*Sewellia*	*elongata*	411.629913	411.1789435	37.675	11.05285769	88.94714218	diploid	non-miniature
LR15188	LR15188	PRJNA1483025	SAMN61141560	SRR39401053	Cobitoidei	Gastromyzontidae	*Vanmanenia*	sp	438.759753	438.5723105	38.36	15.34271941	84.65728048	diploid	non-miniature
113LR15713	LR15713	PRJNA1483025	SAMN61141561	SRR39401084	Cobitoidei	Gastromyzontidae	*Yaoshania*	*pachychilus*	435.03648	435.496029	38.17	14.37973904	85.62026084	diploid	non-miniature
LR15343	LR15343	PRJNA1483025	SAMN61141562	SRR39401082	Cobitoidei	Nemacheilidae	*Aborichthys*	*elongatus*	517.287277	518.9789115	38.035	21.37942651	78.62057349	diploid	non-miniature
LR15190	LR15190	PRJNA1483025	SAMN61141563	SRR39401050	Cobitoidei	Nemacheilidae	*Barbatula*	sp	646.8225025	647.9074045	38.82	29.52406303	70.47593689	diploid	non-miniature
LR15073	LR15073	PRJNA1483025	SAMN61141564	SRR39401048	Cobitoidei	Nemacheilidae	*Homatula*	*variegata*	700.53089	697.3544255	38.135	36.50368811	63.49631189	diploid	non-miniature
KUFOS-AN-2019.2.1	KUFOS-AN-2019.2.1	PRJNA1393636	SAMN54310102	SRR36627404	Cobitoidei	Nemacheilidae	*Indoreonectes*	*keralensis*	398.207894	393.362597	38.775	30.55154008	69.44845979	diploid	non-miniature
LR15462	LR15462	PRJNA1483025	SAMN61141565	SRR39401215	Cobitoidei	Nemacheilidae	*Lefua*	sp	549.922583	551.812393	37.575	21.04294222	78.95705778	diploid	non-miniature
KUFOS-AN-2021.1.1	KUFOS-AN-2021.1.1	PRJNA1393636	SAMN54310103	SRR36627403	Cobitoidei	Nemacheilidae	*Mesonoemacheilus*	*triangularis*	499.488246	493.90045	36.915	20.70205808	79.29794192	diploid	non-miniature
LR15337	LR15337	PRJNA1483025	SAMN61141566	SRR39401214	Cobitoidei	Nemacheilidae	*Micronemacheilus*	sp	552.0035355	551.353682	37.93	23.62578727	76.37421273	diploid	non-miniature
LR15459	LR15459	PRJNA1483025	SAMN61141567	SRR39401213	Cobitoidei	Nemacheilidae	*Nemacheilus*	*selangoricus*	472.4957335	475.761951	38.45	22.29665178	77.70334822	diploid	non-miniature
LR15611	LR15611	PRJNA1483025	SAMN61141568	SRR39401212	Cobitoidei	Nemacheilidae	*Neonoemacheilus*	sp	612.61913	611.2010315	35.765	23.77711735	76.22288257	diploid	non-miniature
LR15490	LR15490	PRJNA1483025	SAMN61141569	SRR39401211	Cobitoidei	Nemacheilidae	*Oreonectes*	*platycephalus*	602.371967	606.3790165	40.025	24.47238913	75.52761087	diploid	non-miniature
LR15504	LR15504	PRJNA1483025	SAMN61141570	SRR39401210	Cobitoidei	Nemacheilidae	*Paracanthocobitis*	*botia*	550.041868	550.526874	35.955	19.81183983	80.18816017	diploid	non-miniature
LR15189	LR15189	PRJNA1483025	SAMN61141571	SRR39401209	Cobitoidei	Nemacheilidae	*Petruichthys*	*brevis*	571.338394	573.6448505	36.625	21.97872776	78.02127233	diploid	non-miniature
LR15152	LR15152	PRJNA1483025	SAMN61141572	SRR39401207	Cobitoidei	Nemacheilidae	*Schistura*	*fasciolata*	697.111332	697.99144	38.055	35.34255914	64.65744078	diploid	non-miniature
LR15454	LR15454	PRJNA1483025	SAMN61141573	SRR39401206	Cobitoidei	Nemacheilidae	*Schistura*	sp1	519.0821805	521.701272	36.8	19.53220271	80.46779729	diploid	non-miniature
LR15496	LR15496	PRJNA1483025	SAMN61141574	SRR39401205	Cobitoidei	Nemacheilidae	*Schistura*	sp2	690.5186275	689.9526065	35.86	33.32503056	66.67496944	diploid	non-miniature
LR15455	LR15455	PRJNA1483025	SAMN61141575	SRR39401204	Cobitoidei	Nemacheilidae	*Sundoreonectes*	*sabanus*	656.4244465	655.1661915	38.23	30.00109317	69.99890676	diploid	non-miniature
LR15072	LR15072	PRJNA1483025	SAMN61141576	SRR39401089	Cobitoidei	Nemacheilidae	*Traccatichthys*	*pulcher*	581.477804	580.90548	37	22.13097198	77.86902802	diploid	non-miniature
LR15357	LR15357	PRJNA1483025	SAMN61141577	SRR39401088	Cobitoidei	Serpenticobitidae	*Serpenticobitis*	*zonata*	NA	440.308335	39.095	34.48299497	65.51700503	diploid	non-miniature
LR15334	LR15334	PRJNA1483025	SAMN61141578	SRR39401087	Cobitoidei	Vaillantellidae	*Vaillantella*	*maassii*	428.3150045	427.556523	39.78	14.17621934	85.82378054	diploid	non-miniature
LR15562	LR15562	PRJNA1483025	SAMN61141579	SRR39401086	Cyprinoidei	Acheilognathidae	*Acheilognathus*	*deignani*	757.3498185	752.796849	37.89	22.19187431	77.80812583	diploid	non-miniature
LR15063	LR15063	PRJNA1483025	SAMN61141580	SRR39401085	Cyprinoidei	Acheilognathidae	*Acheilognathus*	sp	834.4712905	830.2436615	38.01	27.17588672	72.82411322	diploid	non-miniature
LR15636	LR15636	PRJNA1483025	SAMN61141581	SRR39401045	Cyprinoidei	Acheilognathidae	*Paratanakia*	*himantegus*	705.787949	703.096636	37.825	16.1280591	83.87194097	diploid	non-miniature
LR15661	LR15661	PRJNA1483025	SAMN61141582	SRR39401043	Cyprinoidei	Acheilognathidae	*Rhodeus*	*amarus*	841.4043695	840.9927755	38.07	26.92010717	73.07989283	diploid	non-miniature
LR15266	LR15266	PRJNA1483025	SAMN61141583	SRR39401081	Cyprinoidei	Cyprinidae	*Acapoeta*	*tanganicae*	1298.952291	1296.278517	36.77	37.78368453	62.21631551	polyploid	non-miniature
123LR15726	LR15726	PRJNA1483025	SAMN61141584	SRR39401080	Cyprinoidei	Cyprinidae	*Acrossocheilus*	sp	939.853593	937.795839	36.905	29.80463587	70.19536419	diploid	non-miniature
LR15615	LR15615	PRJNA1483025	SAMN61141585	SRR39401079	Cyprinoidei	Cyprinidae	*Ageneiogarra*	sp	1159.513166	1156.287975	36.115	36.18040899	63.81959109	diploid	non-miniature
LR15639	LR15639	PRJNA1483025	SAMN61141586	SRR39401078	Cyprinoidei	Cyprinidae	*Balantiocheilos*	*melanopterus*	739.994228	738.167276	36.585	17.19819723	82.80180277	diploid	non-miniature
LR15079	LR15079	PRJNA1483025	SAMN61141587	SRR39401154	Cyprinoidei	Cyprinidae	*Barbichthys*	*laevis*	892.2714405	887.40424	36.005	20.90348881	79.09651114	diploid	non-miniature
LR15102	LR15102	PRJNA1483025	SAMN61141588	SRR39401152	Cyprinoidei	Cyprinidae	*Barbodes*	*lateristriga*	739.006048	740.2849255	36.175	17.94188446	82.05811554	diploid	non-miniature
120LR15721	LR15721	PRJNA1483025	SAMN61141589	SRR39401151	Cyprinoidei	Cyprinidae	*Barbodes*	*sellifer*	779.856393	778.466779	36.9	25.09242478	74.90757522	diploid	non-miniature
LR15256	LR15256	PRJNA1483025	SAMN61141590	SRR39401150	Cyprinoidei	Cyprinidae	*Barboides*	*gracilis*	637.7446625	636.29766	38.46	23.418011	76.581989	diploid	proportioned-dwarf
EA0124	EA0124	PRJNA1483025	SAMN61141591	SRR39401149	Cyprinoidei	Cyprinidae	*Barbonymus*	*altus*	812.5814355	809.7455375	36.39	20.01326207	79.98673793	diploid	non-miniature
LR15111	LR15111	PRJNA1483025	SAMN61141592	SRR39401147	Cyprinoidei	Cyprinidae	*Ceratogarra*	*cambodgiensis*	848.402555	847.3830295	35.22	16.77129121	83.22870879	diploid	non-miniature
EA0357	EA0357	PRJNA1483025	SAMN61141593	SRR39401146	Cyprinoidei	Cyprinidae	*Chagunius*	*nicholsi*	735.1767355	734.8841255	36.47	18.22099877	81.77900123	diploid	non-miniature
EA0401	EA0401	PRJNA1483025	SAMN61141594	SRR39401145	Cyprinoidei	Cyprinidae	*Chagunius*	*nicholsi*	721.6636435	720.7493135	36.41	15.80898627	84.1910138	diploid	non-miniature
LR15270	LR15270	PRJNA1483025	SAMN61141595	SRR39401144	Cyprinoidei	Cyprinidae	*Clypeobarbus*	sp	831.387745	829.8340545	37.595	30.88071622	69.11928384	diploid	non-miniature
LR10955	LR10955	PRJNA1483025	SAMN61141596	SRR39401143	Cyprinoidei	Cyprinidae	*Crossocheilus*	*reticulatus*	1306.005909	1296.106392	37.195	35.50874892	64.49125112	diploid	non-miniature
LR10341	LR10341	PRJNA1483025	SAMN61141597	SRR39401142	Cyprinoidei	Cyprinidae	*Cyclocheilichthys*	*apogon*	833.8678355	833.4889075	36.67	27.00398049	72.99601963	diploid	non-miniature
KUFOS-AN-2021.4.1	KUFOS-AN-2021.4.1	PRJNA1393636	SAMN54310104	SRR36627402	Cyprinoidei	Cyprinidae	*Dawkinsia*	*filamentosa*	654.118315	638.710795	37.18	16.06996591	83.93003401	diploid	non-miniature

140LR15737	LR15737	PRJNA1483025	SAMN61141598	SRR39401049	Cyprinoidei	Cyprinidae	*Desmopuntius*	*pentazona*	761.649411	765.758192	37.205	27.6709824	72.32901754	diploid	non-miniature
122LR15725	LR15725	PRJNA1483025	SAMN61141599	SRR39401047	Cyprinoidei	Cyprinidae	*Discherodontus*	*ashmeadi*	724.5058075	723.054833	36.62	17.58345062	82.41654938	diploid	non-miniature
119LR15720	LR15720	PRJNA1483025	SAMN61141600	SRR39401046	Cyprinoidei	Cyprinidae	*Eirmotus*	*isthmus*	649.103723	648.622376	37.7	19.57914492	80.42085508	diploid	non-miniature
LR09582	LR09582	PRJNA1483025	SAMN61141601	SRR39401076	Cyprinoidei	Cyprinidae	*Eirmotus*	sp2	660.444307	658.8349075	37.76	21.03191223	78.96808777	diploid	non-miniature
LR15276	LR15276	PRJNA1483025	SAMN61141602	SRR39401074	Cyprinoidei	Cyprinidae	*Enteromius*	sp	880.2979075	878.386854	38.295	29.3805256	70.6194744	diploid	non-miniature
LR15606	LR15606	PRJNA1483025	SAMN61141603	SRR39401042	Cyprinoidei	Cyprinidae	*Enteromius*	sp1	862.4056615	859.7960585	38.285	29.92997071	70.07002929	diploid	non-miniature
LR01655	LR01655	PRJNA1483025	SAMN61141604	SRR39401041	Cyprinoidei	Cyprinidae	*Epalzeorhynchos*	*kalopterum*	1366.941311	1362.220601	37.16	39.10739642	60.89260359	diploid	non-miniature
LR15563	LR15563	PRJNA1483025	SAMN61141605	SRR39401040	Cyprinoidei	Cyprinidae	*Folifer*	*brevifilis*	902.407894	900.587643	36.69	26.63512295	73.36487705	diploid	non-miniature
KUFOS-RR-2022.P.4	KUFOS-RR-2022.P.4	PRJNA1393636	SAMN54310106	SRR36627400	Cyprinoidei	Cyprinidae	*Garra*	*periyarensis*	967.490774	967.001756	35.585	25.41006559	74.58993441	diploid	non-miniature
LR15635	LR15635	PRJNA1483025	SAMN61141606	SRR39401039	Cyprinoidei	Cyprinidae	*Haludaria*	*fasciata*	593.7811045	590.2574145	37.07	11.80774113	88.19225896	diploid	non-miniature
LR15078	LR15078	PRJNA1483025	SAMN61141607	SRR39401038	Cyprinoidei	Cyprinidae	*Hampala*	*ampalong*	668.149162	667.0398945	36.325	13.80415216	86.19584784	diploid	non-miniature
KUFOS-RR-2022.P.3	KUFOS-RR-2022.P.3	PRJNA1393636	SAMN54310107	SRR36627399	Cyprinoidei	Cyprinidae	*Hypselobarbus*	*periyarensis*	765.603222	752.678211	36.765	26.58458237	73.4154177	polyploid	non-miniature
LR15278	LR15278	PRJNA1483025	SAMN61141608	SRR39401037	Cyprinoidei	Cyprinidae	*Labeo*	*cylindricus*	1029.026109	1028.751666	35.85	26.08850821	73.91149174	diploid	non-miniature
LR15282	LR15282	PRJNA1483025	SAMN61141609	SRR39401036	Cyprinoidei	Cyprinidae	*Labeobarbus*	sp	1223.110867	1263.354553	36.7	24.66768204	75.33231801	polyploid	non-miniature
LR12884	LR12884	PRJNA1483025	SAMN61141610	SRR39401035	Cyprinoidei	Cyprinidae	*Labiobarbus*	*ocellatus*	1158.390144	1143.454791	36.38	30.79723043	69.20276957	diploid	non-miniature
KUFOS-RR-2022.P.2	KUFOS-RR-2022.P.2	PRJNA1393636	SAMN54310108	SRR36627398	Cyprinoidei	Cyprinidae	*Lepidopygopsis*	*typus*	NA	1406.545258	36.545	20.48279825	79.51720178	polyploid	non-miniature
LR15104	LR15104	PRJNA1483025	SAMN61141611	SRR39401077	Cyprinoidei	Cyprinidae	*Lobocheilos*	*aureolineatus*	1019.941463	1011.286788	35.445	23.77283387	76.22716613	diploid	non-miniature
LR15565	LR15565	PRJNA1483025	SAMN61141612	SRR39401238	Cyprinoidei	Cyprinidae	*Mystacoleucus*	*argenteus*	683.068658	683.1196445	36.495	14.58110776	85.41889217	diploid	non-miniature
LR15612	LR15612	PRJNA1483025	SAMN61141613	SRR39401237	Cyprinoidei	Cyprinidae	*Neolissochilus*	sp	859.148132	857.157747	36.63	34.45494456	65.54505544	polyploid	non-miniature
143LR15682	LR15682	PRJNA1483025	SAMN61141614	SRR39401236	Cyprinoidei	Cyprinidae	*Oliotius*	*oligolepis*	605.8518375	605.885024	36.49	13.03447269	86.96552731	diploid	non-miniature
EA1291	EA1291	PRJNA1483025	SAMN61141615	SRR39401235	Cyprinoidei	Cyprinidae	*Oreichthys*	sp	682.6832825	679.6391495	36.955	21.79302518	78.20697474	diploid	non-miniature
LR09367	LR09367	PRJNA1483025	SAMN61141616	SRR39401234	Cyprinoidei	Cyprinidae	*Osteobrama*	*cunma*	720.6217455	718.0802345	36.425	17.87122773	82.12877227	diploid	non-miniature
LR15096	LR15096	PRJNA1483025	SAMN61141617	SRR39401233	Cyprinoidei	Cyprinidae	*Osteochilus*	*vittatoides*	1116.880553	1105.531316	35.84	31.77736895	68.22263105	diploid	non-miniature
LR01656	LR01656	PRJNA1483025	SAMN61141618	SRR39401232	Cyprinoidei	Cyprinidae	*Paracrossochilus*	*vittatus*	1281.129962	1281.936214	35.36	34.61154055	65.38845941	diploid	non-miniature
LR15618	LR15618	PRJNA1483025	SAMN61141619	SRR39401231	Cyprinoidei	Cyprinidae	*Paraspinibarbus*	sp	835.5795385	823.683848	36.425	36.01849444	63.98150556	diploid	non-miniature
KUFOS-AN-2021.5.1	KUFOS-AN-2021.5.1	PRJNA1393636	SAMN54310110	SRR36627409	Cyprinoidei	Cyprinidae	*Pethia*	*conchonius*	617.8897375	601.786245	37.68	28.13006801	71.86993207	diploid	non-miniature
LR06584	LR06584	PRJNA1483025	SAMN61141620	SRR39401230	Cyprinoidei	Cyprinidae	*Pethia*	sp1	686.515958	686.5376455	37.79	21.92100921	78.07899079	diploid	non-miniature
LR15646	LR15646	PRJNA1483025	SAMN61141621	SRR39401158	Cyprinoidei	Cyprinidae	*Pethia*	*gelius*	685.863271	683.0203955	37.775	22.43388704	77.56611296	diploid	non-miniature
LR15083	LR15083	PRJNA1483025	SAMN61141622	SRR39401138	Cyprinoidei	Cyprinidae	*Poropuntius*	*normani*	846.9258515	844.2840135	36.62	25.8082846	74.1917154	diploid	non-miniature
LR15065	LR15065	PRJNA1483025	SAMN61141623	SRR39401160	Cyprinoidei	Cyprinidae	*Probarbus*	*jullieni*	1355.013711	1350.403991	36.455	19.35043726	80.64956274	polyploid	non-miniature
125LR15728	LR15728	PRJNA1483025	SAMN61141624	SRR39401161	Cyprinoidei	Cyprinidae	*Puntigrus*	*partipentazona*	599.7943175	597.0353715	37.845	16.26110464	83.73889536	diploid	non-miniature
146LR15695	LR15695	PRJNA1483025	SAMN61141625	SRR39401162	Cyprinoidei	Cyprinidae	*Puntigrus*	*tetrazona*	579.6666315	579.1733795	37.775	12.76734439	87.23265561	diploid	non-miniature
EA1300	EA1300	PRJNA1483025	SAMN61141627	SRR39401164	Cyprinoidei	Cyprinidae	*Puntius*	sp	800.6292725	797.7085135	35.975	22.00119092	77.99880908	diploid	non-miniature
LR15257	LR15257	PRJNA1483025	SAMN61141628	SRR39401165	Cyprinoidei	Cyprinidae	*Rohanella*	*titteya*	755.9280485	752.468901	36.645	23.41543222	76.58456771	diploid	non-miniature
124LR15727	LR15727	PRJNA1483025	SAMN61141629	SRR39401216	Cyprinoidei	Cyprinidae	*Rohtee*	*ogilbii*	708.2852905	707.9410645	36.57	14.36426422	85.63573571	diploid	non-miniature
145LR15691	LR15691	PRJNA1483025	SAMN61141630	SRR39401166	Cyprinoidei	Cyprinidae	*Sahyadria*	*denisonii*	702.825914	697.7408185	36.315	13.14257215	86.85742785	diploid	non-miniature
LR15061	LR15061	PRJNA1483025	SAMN61141631	SRR39401218	Cyprinoidei	Cyprinidae	*Sawbwa*	*resplendens*	793.4834405	792.2622485	37.225	25.74898102	74.25101891	diploid	proportioned-dwarf
LR15564	LR15564	PRJNA1483025	SAMN61141632	SRR39401229	Cyprinoidei	Cyprinidae	*Scaphiodonichthys*	*acanthopterus*	782.533517	780.9871525	36.63	18.54138554	81.45861446	diploid	non-miniature
LR15101	LR15101	PRJNA1483025	SAMN61141633	SRR39401221	Cyprinoidei	Cyprinidae	*Schismatorhynchos*	*heterorhynchos*	929.110408	892.758514	36.11	29.74378842	70.25621158	diploid	non-miniature
LR09392	LR09392	PRJNA1483025	SAMN61141634	SRR39401222	Cyprinoidei	Cyprinidae	*Semiplotus*	*cirrhosus*	793.995474	790.364518	36.74	20.68133163	79.31866837	diploid	non-miniature
111LR15711	LR15711	PRJNA1483025	SAMN61141635	SRR39401223	Cyprinoidei	Cyprinidae	*Spinibarbichthys*	*denticulatus*	1723.798922	1725.003298	36.57	39.76625046	60.23374954	polyploid	non-miniature
LR15617	LR15617	PRJNA1483025	SAMN61141636	SRR39401224	Cyprinoidei	Cyprinidae	*Spinibarbichthys*	sp	1537.113363	1769.692711	36.995	52.79047965	47.20952032	polyploid	non-miniature
126LR15731	LR15731	PRJNA1483025	SAMN61141637	SRR39401225	Cyprinoidei	Cyprinidae	*Striuntius*	*lineatus*	653.126696	652.6217305	36.615	15.65602856	84.34397144	diploid	non-miniature
LR06590	LR06590	PRJNA1483025	SAMN61141638	SRR39401226	Cyprinoidei	Cyprinidae	*Systomus*	*binduchitra*	652.39371	649.663832	36.485	11.72607105	88.27392895	diploid	non-miniature
KUFOS-RR-2022.P.1	KUFOS-RR-2022.P.1	PRJNA1393636	SAMN54310111	SRR36627408	Cyprinoidei	Cyprinidae	*Tariqilabeo*	*periyarensis*	1044.691178	1038.771302	35.925	30.44231188	69.55768812	diploid	non-miniature

LR12880	LR12880	PRJNA1483025	SAMN61141639	SRR39401227	Cyprinoidei	Cyprinidae	*Thynnichthys*	*vaillanti*	950.0666015	946.176263	36.23	22.51738437	77.48261563	diploid	non-miniature
LR09401	LR09401	PRJNA1483025	SAMN61141640	SRR39401153	Cyprinoidei	Cyprinidae	*Tor*	*putitora*	1808.307077	1807.676521	36.59	37.60799209	62.39200791	polyploid	non-miniature
LR01638	LR01638	PRJNA1483025	SAMN61141642	SRR39401157	Cyprinoidei	Danionidae	*Amblypharyngodon*	*chulabhornae*	562.962259	561.2414775	36.375	12.50628345	87.49371664	diploid	non-miniature
fAmbSpp1	fAmbSpp1	PRJEB19273	SAMEA104130232	ERS3583632	Cyprinoidei	Danionidae	*Amblypharyngodon*	sp	581.728592	582.919701	36.385	13.25118492	86.74881508	diploid	non-miniature
LR15576	LR15576	PRJNA1483025	SAMN61141643	SRR39401220	Cyprinoidei	Danionidae	*Barilius*	*mesopotamicus*	631.3848505	632.689895	37.89	16.99791001	83.00208991	diploid	non-miniature
LR15871	LR15871	PRJNA1393767	SAMN54311767	SRR36627391	Cyprinoidei	Danionidae	*Boraras*	*brigittae*	988.95725	989.810021	38.11	34.83462242	65.16537763	diploid	proportioned-dwarf
fBorBri1	fBorBri1	PRJEB19273	SAMEA104026417	ERS3583663	Cyprinoidei	Danionidae	*Boraras*	*brigittae*	954.4988155	950.8321535	37.295	37.03870486	62.96129509	diploid	proportioned-dwarf
fBorMac1	fBorMac1	PRJEB19273	SAMEA104026409	ERS3583680	Cyprinoidei	Danionidae	*Boraras*	*maculatus*	926.329696	923.9557445	36.975	31.4792687	68.52073125	diploid	proportioned-dwarf
LR12206	LR12206	PRJNA1483025	SAMN61141644	SRR39401219	Cyprinoidei	Danionidae	*Boraras*	*micros*	979.406567	953.8686275	37.495	38.5144762	61.4855238	diploid	proportioned-dwarf
LR12190	LR12190	PRJNA1483025	SAMN61141645	SRR39401217	Cyprinoidei	Danionidae	*Boraras*	*urophthalmoides*	1049.458764	1043.537416	36.865	36.64706884	63.35293112	diploid	proportioned-dwarf
LR09154	LR09154	PRJNA1483025	SAMN61141646	SRR39401141	Cyprinoidei	Danionidae	*Brevibora*	*cheeya1*	1060.505295	1057.686109	36.87	34.98536646	65.01463354	diploid	proportioned-dwarf
LR10130	LR10130	PRJNA1483025	SAMN61141647	SRR39401140	Cyprinoidei	Danionidae	*Brevibora*	*cheeya2*	1038.498306	1029.485158	37.255	35.45790172	64.54209824	diploid	proportioned-dwarf
LR11616	LR11616	PRJNA1483025	SAMN61141648	SRR39401139	Cyprinoidei	Danionidae	*Brevibora*	*dorsiocellata*	870.2978495	866.844275	36.185	25.42497976	74.57502018	diploid	proportioned-dwarf
LR15567	LR15567	PRJNA1483025	SAMN61141649	SRR39401122	Cyprinoidei	Danionidae	*Cabdio*	*morar*	552.56314	549.1380975	38.295	9.992699696	90.0073003	diploid	non-miniature
fSalSpp1	fSalSpp1	PRJEB19273	SAMEA104130237	ERS3583677	Cyprinoidei	Danionidae	*Cabdio*	sp	546.8581005	545.008121	38.3	9.799058389	90.20094161	diploid	non-miniature
fCheCac1	fCheCac1	PRJEB19273	SAMEA104130244	ERS3583665	Cyprinoidei	Danionidae	*Chela*	*cachius*	1016.696674	1008.955852	36.315	20.65499586	79.34500414	diploid	non-miniature
131LR08589	LR08589	PRJNA1483025	SAMN61141650	SRR39401121	Cyprinoidei	Danionidae	*Chelaethiops*	*elongatus*	581.0360465	579.350842	38.52	15.21571716	84.78428284	diploid	non-miniature
LR15596	LR15596	PRJNA1483025	SAMN61141651	SRR39401120	Cyprinoidei	Danionidae	*Chelaethiops*	*bibie*	605.3423	608.825888	38.135	19.70585374	80.29414643	diploid	non-miniature
LR15597	LR15597	PRJNA1483025	SAMN61141652	SRR39401118	Cyprinoidei	Danionidae	*Chelaethiops*	sp2	592.8523575	593.233103	38.17	16.40151991	83.59848009	diploid	non-miniature
LR01642	LR01642	PRJNA1483025	SAMN61141653	SRR39401137	Cyprinoidei	Danionidae	*Danio*	*erythromicron*	1011.558199	1006.792028	36.13	23.33284908	76.66715087	diploid	proportioned-dwarf
LR02056	LR02056	PRJNA1483025	SAMN61141654	SRR39401136	Cyprinoidei	Danionidae	*Danio*	*margaritatus*	929.9375845	928.546121	35.925	17.42602191	82.57397809	diploid	proportioned-dwarf
fDevDev2	fDevDev2	PRJEB19273	SAMEA104132453	ERS3583631	Cyprinoidei	Danionidae	*Devario*	*devario*	1630.174967	1617.316438	37.255	37.17728269	62.82271731	diploid	non-miniature
fDevDev3	fDevDev3	PRJEB19273	SAMEA104132451	ERS3583635	Cyprinoidei	Danionidae	*Devario*	*devario*	1749.706862	1741.259617	39.46	52.44615917	47.55384083	diploid	non-miniature
144LR15698	LR15698	PRJNA1483025	SAMN61141655	SRR39401135	Cyprinoidei	Danionidae	*Devario*	*sondhii*	1068.535952	1064.408718	35.98	22.14562574	77.85437426	diploid	non-miniature
fDanAeq2	fDanAeq2	PRJEB19273	SAMEA104132461	ERS3583672	Cyprinoidei	Danionidae	*Devario*	sp	1627.877875	1613.603733	38.88	41.4604256	58.53957443	diploid	non-miniature
LR15598	LR15598	PRJNA1483025	SAMN61141656	SRR39401134	Cyprinoidei	Danionidae	*Engraulicypris*	sp1	573.83676	574.0788735	38.085	15.58897333	84.41102667	diploid	non-miniature
LR15656	LR15656	PRJNA1483025	SAMN61141657	SRR39401133	Cyprinoidei	Danionidae	*Engraulicypris*	*spinifer*	557.1908095	554.3224095	38.575	12.67496682	87.32503327	diploid	non-miniature
116LR15716	LR15716	PRJNA1483025	SAMN61141658	SRR39401132	Cyprinoidei	Danionidae	*Esomus*	*danricus*	527.9941165	521.562969	37.525	19.2429367	80.7570633	diploid	non-miniature
LR01653	LR01653	PRJNA1483025	SAMN61141659	SRR39401131	Cyprinoidei	Danionidae	*Esomus*	*metallicus*	514.2231505	513.0350255	38.225	14.0032484	85.9967517	diploid	non-miniature
LR12503	LR12503	PRJNA1483025	SAMN61141660	SRR39401130	Cyprinoidei	Danionidae	*Esomus*	sp	520.142126	519.440578	38.17	14.79949801	85.20050218	diploid	non-miniature
fHorAtu1	fHorAtu1	PRJEB19273	SAMEA104130248	ERS3583662	Cyprinoidei	Danionidae	*Horadandia*	*atukorali*	587.07259	585.840953	36.935	14.809369	85.19063108	diploid	proportioned-dwarf
LR01646	LR01646	PRJNA1483025	SAMN61141661	SRR39401129	Cyprinoidei	Danionidae	*Inlecypris*	*auropurpurea*	1318.710992	1316.965625	36.625	31.00061246	68.99938754	diploid	non-miniature
LR01674	LR01674	PRJNA1483025	SAMN61141662	SRR39401127	Cyprinoidei	Danionidae	*Kottelatia*	*brittani*	688.5714295	689.6199065	36.5	15.48006576	84.51993424	diploid	non-miniature
fLauMye1	fLauMye1	PRJEB19273	SAMEA104130242	ERS3583667	Cyprinoidei	Danionidae	*Laubuka*	*siamensis*	1337.327615	1336.049704	35.825	26.07221746	73.92778255	diploid	non-miniature
132LR09413	LR09413	PRJNA1483025	SAMN61141663	SRR39401126	Cyprinoidei	Danionidae	*Laubuka*	sp1	1302.83026	1300.971656	36.075	25.24441075	74.75558925	diploid	non-miniature
LR02238	LR02238	PRJNA1483025	SAMN61141664	SRR39401125	Cyprinoidei	Danionidae	*Laubuka*	sp1	1327.397792	1330.272617	36.265	26.7237997	73.2762003	diploid	non-miniature
KUFOS-AN-2021.3.1	KUFOS-AN-2021.3.1	PRJNA1393636	SAMN54310112	SRR36627407	Cyprinoidei	Danionidae	*Laubuka*	sp2	1283.333135	1271.61059	36.68	29.4850516	70.51494844	diploid	non-miniature
LR15601	LR15601	PRJNA1483025	SAMN61141665	SRR39401124	Cyprinoidei	Danionidae	*Leptocypris*	*niloticus*	619.8556505	620.067572	38.045	16.96280611	83.03719389	diploid	non-miniature
LR01659	LR01659	PRJNA1483025	SAMN61141666	SRR39401123	Cyprinoidei	Danionidae	*Luciosoma*	*pellegrinii*	619.836723	618.508899	37.615	11.54754008	88.45245984	diploid	non-miniature
fLucSpp1	fLucSpp1	PRJEB19273	SAMEA104130230	ERS3583634	Cyprinoidei	Danionidae	*Luciosoma*	sp1	601.8549145	599.554698	37.835	11.46548884	88.53451116	diploid	non-miniature
LR15098	LR15098	PRJNA1483025	SAMN61141667	SRR39401264	Cyprinoidei	Danionidae	*Malayochela*	*maassi*	832.0147615	828.8339765	36.75	23.61088681	76.38911313	diploid	non-miniature
LR15378	LR15378	PRJNA1483025	SAMN61141668	SRR39401263	Cyprinoidei	Danionidae	*Malayochela*	*maassi*	729.5774585	712.94025	37.045	28.63289399	71.36710601	diploid	non-miniature
LR15109	LR15109	PRJNA1483025	SAMN61141669	SRR39401262	Cyprinoidei	Danionidae	*Microdevario*	*kubotai*	1152.092582	1081.146097	36.12	30.35980261	69.64019739	diploid	proportioned-dwarf
LR15568	LR15568	PRJNA1483025	SAMN61141670	SRR39401261	Cyprinoidei	Danionidae	*Microdevario*	*kubotai*	1010.318699	1010.972865	35.7	22.46742552	77.53257448	diploid	proportioned-dwarf
LR15650	LR15650	PRJNA1483025	SAMN61141671	SRR39401258	Cyprinoidei	Danionidae	*Microrasbora*	*rubescens*	1023.775788	1019.876315	36.12	22.08524821	77.91475179	diploid	proportioned-dwarf

LR15651	LR15651	PRJNA1483025	SAMN61141672	SRR39401115	Cyprinoidei	Danionidae	*Microrasbora*	*rubescens*	1031.156996	1026.213713	36.155	22.93278448	77.06721552	diploid	proportioned-dwarf
121LR15723	LR15723	PRJNA1483025	SAMN61141673	SRR39401203	Cyprinoidei	Danionidae	*Nematabramis*	*everetti*	860.7013535	861.4281705	36.235	21.58896915	78.41103085	diploid	non-miniature
LR15099	LR15099	PRJNA1483025	SAMN61141674	SRR39401202	Cyprinoidei	Danionidae	*Nematabramis*	*steindachneri*	837.175678	831.6380315	36.4	22.15794883	77.84205117	diploid	non-miniature
LR14062	LR14062	PRJNA1483025	SAMN61141675	SRR39401201	Cyprinoidei	Danionidae	*Neochela*	*dadiburjori*	766.42748	761.266707	36.605	12.00586386	87.99413614	diploid	non-miniature
LR15289	LR15289	PRJNA1483025	SAMN61141676	SRR39401200	Cyprinoidei	Danionidae	*Opsaridium*	sp1	670.7835045	672.00933	37.755	20.86341963	79.13658037	diploid	non-miniature
LR15290	LR15290	PRJNA1483025	SAMN61141677	SRR39401199	Cyprinoidei	Danionidae	*Opsaridium*	*zambezense*	643.3171475	644.8919075	37.785	17.24183273	82.75816734	diploid	non-miniature
LR15602	LR15602	PRJNA1483025	SAMN61141678	SRR39401198	Cyprinoidei	Danionidae	*Opsaridium*	*ubangiense*	684.0337825	686.125769	37.795	22.08967442	77.91032558	diploid	non-miniature
LR02136	LR02136	PRJNA1483025	SAMN61141681	SRR39401195	Cyprinoidei	Danionidae	*Opsarius*	sp2	567.2292515	565.8439115	38.02	9.41062675	90.58937325	diploid	non-miniature
142LR15666	LR15666	PRJNA1483025	SAMN61141679	SRR39401197	Cyprinoidei	Danionidae	*Opsarius*	sp1	597.339292	596.7035535	37.63	11.58581788	88.41418212	diploid	non-miniature
LR12240	LR12240	PRJNA1483025	SAMN61141680	SRR39401196	Cyprinoidei	Danionidae	*Opsarius*	*pulchellus*	589.024859	588.440035	37.645	12.0163329	87.98366702	diploid	non-miniature
LR15081	LR15081	PRJNA1483025	SAMN61141682	SRR39401193	Cyprinoidei	Danionidae	*Pectenocypris*	sp	789.7901885	783.181521	36.85	21.68746293	78.31253707	diploid	non-miniature
LR15349	LR15349	PRJNA1483025	SAMN61141683	SRR39401192	Cyprinoidei	Danionidae	*Pectenocypris*	sp	784.015713	779.766298	36.9	20.57990489	79.42009505	diploid	non-miniature
fRaiGut1	fRaiGut1	PRJEB19273	SAMEA104130243	ERS3583669	Cyprinoidei	Danionidae	*Raiamas*	*guttatus*	618.5552755	623.62552	37.585	16.50809519	83.49190465	diploid	non-miniature
LR15292	LR15292	PRJNA1483025	SAMN61141684	SRR39401191	Cyprinoidei	Danionidae	*Raiamas*	*moorii*	620.012495	619.3998015	37.78	14.42400705	85.57599295	diploid	non-miniature
105LR15605	LR15605	PRJNA1483025	SAMN61141685	SRR39401260	Cyprinoidei	Danionidae	*Raiamas*	sp	684.3057705	686.7324995	37.83	20.96136125	79.03863883	diploid	non-miniature
LR10786	LR10786	PRJNA1483025	SAMN61141686	SRR39401259	Cyprinoidei	Danionidae	*Rasbora*	*cephalotaenia*	765.2149865	766.9291225	36.405	23.68579542	76.31420464	diploid	non-miniature
KUFOS-AN-2021.2.1	KUFOS-AN-2021.2.1	PRJNA1393636	SAMN54310113	SRR36627406	Cyprinoidei	Danionidae	*Rasbora*	*dandia*	555.9486295	540.6536305	36.955	18.78565504	81.21434496	diploid	non-miniature
fRasDan1	fRasDan1	PRJEB19273	SAMEA104130236	ERS3583681	Cyprinoidei	Danionidae	*Rasbora*	*daniconius*	676.8139335	675.4717815	37.07	19.42283143	80.57716849	diploid	non-miniature
LR15080	LR15080	PRJNA1483025	SAMN61141687	SRR39401189	Cyprinoidei	Danionidae	*Rasbora*	*kalbarensis*	817.9699085	812.5812705	36.885	26.1303236	73.86967634	diploid	proportioned-dwarf
LR15347	LR15347	PRJNA1483025	SAMN61141688	SRR39401188	Cyprinoidei	Danionidae	*Rasbora*	*kalbarensis*	819.747148	814.203926	36.88	26.00969582	73.99030406	diploid	proportioned-dwarf
LR15877	LR15877	PRJNA1393767	SAMN54311768	SRR36627390	Cyprinoidei	Danionidae	*Rasbora*	*kalochroma*	1295.036907	1290.849861	36.79	41.36926434	58.63073566	diploid	non-miniature
fRasKal1	fRasKal1	PRJEB19273	SAMEA104130246	ERS3583664	Cyprinoidei	Danionidae	*Rasbora*	*kalochroma*	1237.901692	1230.76338	36.625	39.81171283	60.18828713	diploid	non-miniature
LR10804	LR10804	PRJNA1483025	SAMN61141689	SRR39401187	Cyprinoidei	Danionidae	*Rasbora*	*kottelati*	1259.248406	1246.322742	37.16	42.25710608	57.74289392	diploid	non-miniature
LR09739	LR09739	PRJNA1483025	SAMN61141690	SRR39401257	Cyprinoidei	Danionidae	*Rasbora*	*myersi*	778.628299	766.9803475	36.7	29.28257155	70.71742845	diploid	non-miniature
fRasRas1	fRasRas1	PRJEB19273	SAMEA104130231	ERS3583629	Cyprinoidei	Danionidae	*Rasbora*	*rasbora*	920.124033	914.083791	36.565	30.36400287	69.63599713	diploid	non-miniature
LR09847	LR09847	PRJNA1483025	SAMN61141691	SRR39401256	Cyprinoidei	Danionidae	*Rasbora*	*trilineata*	896.8334345	885.3081555	36.36	32.66581181	67.33418813	diploid	non-miniature
LR05463	LR05463	PRJNA1483025	SAMN61141692	SRR39401254	Cyprinoidei	Danionidae	*Rasboroides*	*pallidus*	628.5963905	628.5166625	36.685	14.65054079	85.34945921	diploid	non-miniature
LR12212	LR12212	PRJNA1483025	SAMN61141693	SRR39401253	Cyprinoidei	Danionidae	*Rasbosoma*	*spilocerca*	907.9765275	901.6240925	36.575	29.97125712	70.02874288	diploid	proportioned-dwarf
LR12213	LR12213	PRJNA1483025	SAMN61141694	SRR39401116	Cyprinoidei	Danionidae	*Rasbosoma*	*spilocerca*	901.71471	895.1785345	36.59	29.16692072	70.83307922	diploid	proportioned-dwarf
KUFOS-AN-2021.6.1	KUFOS-AN-2021.6.1	PRJNA1393636	SAMN54310114	SRR36627405	Cyprinoidei	Danionidae	*Salmostoma*	*balookee*	582.255672	583.0889565	37.29	15.32054021	84.67945988	diploid	non-miniature
EA0634	EA0634	PRJNA1483025	SAMN61141695	SRR39401114	Cyprinoidei	Danionidae	*Thryssocypris*	*tonlesapensis*	448.330809	447.905259	42.28	16.53313017	83.46686983	diploid	non-miniature
EA0635	EA0635	PRJNA1483025	SAMN61141696	SRR39401113	Cyprinoidei	Danionidae	*Thryssocypris*	*tonlesapensis*	430.5066165	429.476711	42.305	13.58671542	86.41328447	diploid	non-miniature
LR08172	LR08172	PRJNA1483025	SAMN61141697	SRR39401112	Cyprinoidei	Danionidae	*Trigonopoma*	*gracile*	870.360911	872.3102705	36.855	29.28424067	70.71575933	diploid	non-miniature
LR10181	LR10181	PRJNA1483025	SAMN61141698	SRR39401111	Cyprinoidei	Danionidae	*Trigonopoma*	*pauciperforatum*	992.3662515	984.3918645	36.385	35.12946678	64.87053322	diploid	non-miniature
LR11656	LR11656	PRJNA1483025	SAMN61141699	SRR39401110	Cyprinoidei	Danionidae	*Trigonopoma*	sp	1081.769351	1076.334203	37.255	40.57677174	59.42322826	diploid	non-miniature
LR08812	LR08812	PRJNA1483025	SAMN61141700	SRR39401109	Cyprinoidei	Danionidae	*Trigonostigma*	*espei*	1114.404431	1111.510812	36.355	35.96935067	64.03064933	diploid	proportioned-dwarf
LR11086	LR11086	PRJNA1483025	SAMN61141701	SRR39401108	Cyprinoidei	Danionidae	*Trigonostigma*	*heteromorpha*	1120.628596	1115.305677	36.425	36.53286278	63.46713722	diploid	proportioned-dwarf
LR15569	LR15569	PRJNA1483025	SAMN61141702	SRR39401106	Cyprinoidei	Gobionidae	*Hemibarbus*	*maculatus*	1029.489612	1025.660767	37.66	31.45704334	68.54295661	diploid	non-miniature
LR15570	LR15570	PRJNA1483025	SAMN61141703	SRR39401105	Cyprinoidei	Gobionidae	*Ladislavia*	*taczanowskii*	1118.263871	1113.559064	38.245	36.99116859	63.00883141	diploid	non-miniature
LR15062	LR15062	PRJNA1483025	SAMN61141704	SRR39401104	Cyprinoidei	Gobionidae	*Microphysogobio*	*tafangensis*	952.537671	947.9214225	37.64	25.53273096	74.46726904	diploid	non-miniature
LR15571	LR15571	PRJNA1483025	SAMN61141705	SRR39401102	Cyprinoidei	Gobionidae	*Sarcocheilichthys*	*hainanensis*	1219.380388	1208.260729	38.17	41.26247212	58.73752784	diploid	non-miniature
LR15579	LR15579	PRJNA1483025	SAMN61141706	SRR39401190	Cyprinoidei	Gobionidae	*Squalidus*	*atromaculatus*	995.523156	994.7189	37.81	30.10430248	69.89569752	diploid	non-miniature
LR15106	LR15106	PRJNA1483025	SAMN61141707	SRR39401062	Cyprinoidei	Leptobarbidae	*Leptobarbus*	*hoeveni*	1001.135404	1001.959424	36.515	22.81550206	77.18449794	diploid	non-miniature
1360_2014	NMBE 1070731	PRJNA1483025	SAMN61141708	SRR39401252	Cyprinoidei	Leuciscidae	*Abramis*	*brama*	1085.946882	1081.577457	37.885	37.34699374	62.65300626	diploid	non-miniature
2180_2012	NMBE 1065116	PRJNA1483025	SAMN61141709	SRR39401250	Cyprinoidei	Leuciscidae	*Alburnoides*	*bipunctatus*	1125.0653	1096.164941	38.96	53.86762899	46.13237092	diploid	non-miniature

108LR15742	TCWC 20551.01	PRJNA1483025	SAMN61141710	SRR39401186	Cyprinoidei	Leuciscidae	*Chrosomus*	*erythrogaster*	1136.177841	1134.19284	37.855	40.34042264	59.65957745	diploid	non-miniature
LR15250	TCWC 20295.02	PRJNA1483025	SAMN61141711	SRR39401185	Cyprinoidei	Leuciscidae	*Cyprinella*	*lutrensis*	990.908487	1010.965464	37.69	43.3685845	56.6314155	diploid	non-miniature
LR15252	TCWC 20317.01	PRJNA1483025	SAMN61141712	SRR39401182	Cyprinoidei	Leuciscidae	*Dionda*	*flavipinnis*	818.2824675	817.7215975	37.845	19.02917894	80.97082106	diploid	non-miniature
110LR15746	MSB 114786.001	PRJNA1483025	SAMN61141713	SRR39401181	Cyprinoidei	Leuciscidae	*Gila*	*pandora*	1135.724004	1133.136695	37.76	38.67681595	61.32318405	diploid	non-miniature
LR15253	TCWC 20318.01	PRJNA1483025	SAMN61141714	SRR39401180	Cyprinoidei	Leuciscidae	*Hybognathus*	*nuchalis*	943.459128	937.2453165	37.955	34.147014	65.852986	diploid	non-miniature
2223_2013	NMBE 1068595	PRJNA1483025	SAMN61141715	SRR39401179	Cyprinoidei	Leuciscidae	*Leuciscus*	*leuciscus*	1162.57238	1159.306152	38.045	42.02645674	57.97354326	diploid	non-miniature
LR15254	TCWC 20317.03	PRJNA1483025	SAMN61141716	SRR39401073	Cyprinoidei	Leuciscidae	*Notropis*	*chalybaeus*	1232.783309	1230.957706	37.88	43.71301054	56.28698946	diploid	non-miniature
2564_2011	NMBE 1061900	PRJNA1483025	SAMN61141717	SRR39401072	Cyprinoidei	Leuciscidae	*Phoxinus*	*csikii*	985.5204915	980.731219	37.955	36.16648661	63.83351339	diploid	non-miniature
109LR15744	MSB 113977-28	PRJNA1483025	SAMN61141718	SRR39401071	Cyprinoidei	Leuciscidae	*Ptychocheilus*	*lucius*	1097.619044	1094.060096	37.72	37.27123859	62.72876146	diploid	non-miniature
LR15251	TCWC 19726.02	PRJNA1483025	SAMN61141719	SRR39401070	Cyprinoidei	Leuciscidae	*Rhinichthys*	*cataractae*	1107.065181	1101.099443	37.915	40.64013989	59.35986011	diploid	non-miniature
LR15572	LR15572	PRJNA1483025	SAMN61141720	SRR39401069	Cyprinoidei	Leuciscidae	*Rhynchocypris*	*lagowskii*	1241.678079	1230.470575	38.64	48.7690066	51.23099344	diploid	non-miniature
2333_2013	NMBE 1068705	PRJNA1483025	SAMN61141721	SRR39401068	Cyprinoidei	Leuciscidae	*Rutilus*	*rutilus*	1065.305076	1063.445747	38.075	37.38804477	62.61195528	diploid	non-miniature
1010_2016	NMBE 1074568	PRJNA1483025	SAMN61141722	SRR39401066	Cyprinoidei	Leuciscidae	*Scardinius*	*hesperidicus*	1107.772575	1103.761005	38	38.51784805	61.48215195	diploid	non-miniature
LR15249	TCWC 15788.06	PRJNA1483025	SAMN61141723	SRR39401065	Cyprinoidei	Leuciscidae	*Semotilus*	*atromaculatus*	1172.868109	1174.221529	38.06	49.82682633	50.17317367	diploid	non-miniature
1104_2014	NMBE 1069134	PRJNA1483025	SAMN61141724	SRR39401064	Cyprinoidei	Leuciscidae	*Squalius*	*cephalus*	1088.206773	1084.922084	37.855	37.27283476	62.72716533	diploid	non-miniature
1450_2013	NMBE 1067637	PRJNA1483025	SAMN61141725	SRR39401063	Cyprinoidei	Leuciscidae	*Telestes*	*muticellus*	1203.785539	1160.188897	38.975	51.92548272	48.07451728	diploid	non-miniature
LR12004	LR12004	PRJEB15524	SAMEA4467423	ERS1366603	Cyprinoidei	Paedocyprididae	*Paedocypris*	*carbunculus*	426.238552	422.0606155	39.25	16.11646586	83.88353414	diploid	progenetic-miniature
LR7898	LR7898	PRJEB15524	SAMEA4467424	ERS1366602	Cyprinoidei	Paedocyprididae	*Paedocypris*	*micromegethes2*	410.3454355	407.596544	39.265	13.93414955	86.06585033	diploid	progenetic-miniature
LR15863A	LR15863A	PRJNA1393767	SAMN54311765	SRR36627396	Cyprinoidei	Paedocyprididae	*Paedocypris*	sp1	409.2763025	401.451712	39.73	18.14113587	81.85886426	diploid	progenetic-miniature
LR15325	LR15325	PRJNA1483025	SAMN61141726	SRR39401251	Cyprinoidei	Paedocyprididae	*Paedocypris*	sp2	514.3421975	517.668667	39.375	30.34004432	69.65995558	diploid	progenetic-miniature
LR09467	LR09467	PRJNA1483025	SAMN61141727	SRR39401090	Cyprinoidei	Psilorhynchidae	*Psilorhynchus*	*brachyrhynchus*	480.9894595	482.2281655	39.58	19.16622091	80.83377909	diploid	non-miniature
LR15089	LR15089	PRJNA1483025	SAMN61141728	SRR39401178	Cyprinoidei	Psilorhynchidae	*Psilorhynchus*	sp2	390.3520735	389.6027315	39.495	17.83568797	82.16431203	diploid	non-miniature
100LR08982	LR08982	PRJNA1483025	SAMN61141729	SRR39401249	Cyprinoidei	Sundadanionidae	*Fangfangia*	*spinicleithralis*	761.3633435	761.3466415	37.8	19.51679464	80.48320536	diploid	progenetic-miniature
LR15870	LR15870	PRJNA1393767	SAMN54311766	SRR36627395	Cyprinoidei	Sundadanionidae	*Sundadanio*	*atomus*	655.9743745	654.471117	37.71	19.43013988	80.5698602	diploid	progenetic-miniature
LR10372	LR10372	PRJNA1483025	SAMN61141730	SRR39401248	Cyprinoidei	Sundadanionidae	*Sundadanio*	*goblinus*	685.971495	679.917281	37.515	20.30890674	79.69109326	diploid	progenetic-miniature
LR15082	LR15082	PRJNA1483025	SAMN61141731	SRR39401247	Cyprinoidei	Sundadanionidae	*Sundadanio*	*margarition*	720.5398105	716.7551635	37.795	24.97589737	75.02410263	diploid	progenetic-miniature
101LR12751	LR12751	PRJNA1483025	SAMN61141732	SRR39401245	Cyprinoidei	Sundadanionidae	*Sundadanio*	*retiarius*	757.7895775	752.2425955	37.645	25.91038937	74.08961063	diploid	progenetic-miniature
fSunRet1	fSunRet1	PRJEB19273	SAMEA104026377	ERS3583670	Cyprinoidei	Sundadanionidae	*Sundadanio*	*retiarius*	726.206226	718.286569	37.85	25.46005131	74.53994862	diploid	progenetic-miniature
LR15110	LR15110	PRJNA1483025	SAMN61141733	SRR39401244	Cyprinoidei	Tanichthyidae	*Tanichthys*	*albonudes*	1080.797926	1071.863989	36.53	33.97173207	66.02826793	diploid	proportioned-dwarf
LR02462	LR02462	PRJNA1483025	SAMN61141734	SRR39401243	Cyprinoidei	Tanichthyidae	*Tanichthys*	*micagemmae*	1102.18459	1093.443826	36.965	34.81896184	65.18103816	diploid	proportioned-dwarf
1545_2010	NMBE 1058507	PRJNA1483025	SAMN61141735	SRR39401242	Cyprinoidei	Tincidae	*Tinca*	*tinca*	1054.17274	1048.69225	38.455	36.88803965	63.11196035	diploid	non-miniature
118LR15719	LR15719	PRJNA1483025	SAMN61141736	SRR39401241	Cyprinoidei	Xenocyprididae	*Candidia*	*pingtungensis*	784.0223965	780.192702	37.66	20.80881231	79.19118769	diploid	non-miniature
103LR15622	LR15622	PRJNA1483025	SAMN61141737	SRR39401103	Cyprinoidei	Xenocyprididae	*Hemiculter*	sp	981.3826645	977.935087	37.11	29.18360971	70.81639034	diploid	non-miniature
102LR15621	LR15621	PRJNA1483025	SAMN61141738	SRR39401101	Cyprinoidei	Xenocyprididae	*Opsariichthys*	*bidens*	902.7294465	905.3265225	37.64	31.18553417	68.81446589	diploid	non-miniature
128LR15724	LR15724	PRJNA1483025	SAMN61141739	SRR39401100	Cyprinoidei	Xenocyprididae	*Opsariichthys*	*pachycephalus*	755.937811	734.149207	38.145	35.85926444	64.14073556	diploid	non-miniature
LR15574	LR15574	PRJNA1483025	SAMN61141740	SRR39401099	Cyprinoidei	Xenocyprididae	*Oxygaster*	*pointoni*	865.7637945	862.965098	36.955	24.58985201	75.41014805	diploid	non-miniature
LR15097	LR15097	PRJNA1483025	SAMN61141741	SRR39401098	Gyrinocheiloidei	Gyrinocheilidae	*Gyrinocheilus*	*pustulosus*	479.815158	478.3126755	39.435	13.44597818	86.55402182	diploid	non-miniature
104EA0138	EA0138	PRJNA1483025	SAMN61141742	SRR39401096	Gyrinocheiloidei	Gyrinocheilidae	*Gyrinocheilus*	sp	475.4019395	475.3712995	39.52	14.85680637	85.14319363	diploid	non-miniature

Table S11. Cytometric genome size estimates for cypriniform species compiled from the Animal Genome Size Database, accessed on 11 November 2024. Records were curated to retain only genera represented in the k-mer profiling dataset of the present study.

Suborder	Family	Genus	C-value	Method	Cell type	Std sp	genomesize_ MB	References
Catostomoidei	Catostomidae	*Erimyzon*	1.86	FCM	RBC	CCR	1819.08	Ferris, S.D. (1984). Tetraploidy and the evolution of the catostomid fishes. In: Evolutionary Genetics of Fishes, edited by B.J. Turner. Plenum Press, New York. 55-93.
Catostomoidei	Catostomidae	*Moxostoma*	2.14	FCM	RBC	CCR	2092.92	Ferris, S.D. (1984). Tetraploidy and the evolution of the catostomid fishes. In: Evolutionary Genetics of Fishes, edited by B.J. Turner. Plenum Press, New York. 55-93.
Catostomoidei	Catostomidae	*Myxocyprinus*	2.02	FD	RBC	CP	1975.56	Suzuki, A. (1992b). Karyotype and DNA contents of the Chinese catostomid fish, Myxocyprinius asiaticus. Chromosome Information Service 53: 25-27.
Catostomoidei	Catostomidae	*Myxocyprinus*	2.75	FCM	RBC	GD	2689.5	Zhu, D., W. Song, K. Yang, X. Cao, Y. Gul, and W. Wang (2012). Flow cytometric determination of genome size for eight commercially important fish species in China. In Vitro CellularÃ‚Â &Ã‚Â Developmental BiologyÃ‚Â -Ã‚Â Animal 48: 507-517.
Cobitoidei	Balitoridae	*Homaloptera*	0.47	FD	RBC	CP	459.66	Suzuki, A. and Y. Taki (1991). Karyological and cytochemical studies of Pseudogastromyzon myersi and Homaloptera hoffmanni in the order Cypriniformes. Chromosome Information Service 52: 32-34.
Cobitoidei	Botiidae	*Botia*	0.78	FCM	RBC	GD	762.84	Kushwaha, B., N. S. Nagpure, S. Srivastava, M. Pandey, R. Kumar, S. Raizada, S. Agarwal, M. Singh, V. S. Basheer, R. G. Kumar, P. Das, S. P. Das, S. Patnaik, A. Bit, S. K. Srivastava, A. L. Vishwakarma, C. G. Joshi, D. Kumar, and J. K. Jena (2023). Genome
Cobitoidei	Botiidae	*Botia*	0.8	FCM	RBC	MM	782.4	Ojima, Y. and K. Yamamoto (1990). Cellular DNA contents of fishes determined by flow cytometry. La Kromosomo II 57: 1871-1888.
Cobitoidei	Botiidae	*Botia*	0.84	BFA	RBC	SP	821.52	Hinegardner, R. and D.E. Rosen (1972). Cellular DNA content and the evolution of teleostean fishes. American Naturalist 106: 621-644.
Cobitoidei	Botiidae	*Botia*	0.93	FD	RBC	HS	909.54	Muramoto, J., S. Ohno, and N.B. Atkin (1968). On the diploid state of the fish order Ostariophysi. Chromosoma 24: 59-66.
Cobitoidei	Botiidae	*Botia*	0.95	FD	RBC	CP	929.1	Suzuki, A. (1992a). Chromosome and DNA studies of eight species in the family Cobitidae (Pisces, Cypriniformes). La Kromosomo II 67/68: 2275-2282.
Cobitoidei	Botiidae	*Botia*	1.03	FCM	RBC	MM	1007.34	Ojima, Y. and K. Yamamoto (1990). Cellular DNA contents of fishes determined by flow cytometry. La Kromosomo II 57: 1871-1888.
Cobitoidei	Botiidae	*Botia*	1.21	FCM	RBC	GD	1183.38	Kushwaha, B., N. S. Nagpure, S. Srivastava, M. Pandey, R. Kumar, S. Raizada, S. Agarwal, M. Singh, V. S. Basheer, R. G. Kumar, P. Das, S. P. Das, S. Patnaik, A. Bit, S. K. Srivastava, A. L. Vishwakarma, C. G. Joshi, D. Kumar, and J. K. Jena (2023). Genome
Cobitoidei	Botiidae	*Leptobotia*	0.56	FD	RBC	CP	547.68	Suzuki, A. (1992a). Chromosome and DNA studies of eight species in the family Cobitidae (Pisces, Cypriniformes). La Kromosomo II 67/68: 2275-2282.
Cobitoidei	Cobitidae	*Cobitis*	1.4	FD	LV	CA	1369.2	Park, E.-H. and C.Y. Chung (1985). Genome and nuclear sizes of Korean cobitid fishes (Teleostomi: Cypriniformes). Korean Journal of Genetics 7: 111-118.
Cobitoidei	Cobitidae	*Cobitis*	1.42	FCM	RBC	MM	1388.76	Vasil'ev, V.P., A.E. Vinogradov, Y.M. Rozanov, and E.D. Vasil'eva (1999). Cellular DNA content in different forms of the bisexual-unisexual complex of spined loaches of the genus Cobitis and in Luther's spined loach C. lutheri (Cobitidae). Journal of Icht
Cobitoidei	Cobitidae	*Cobitis*	1.54	FCM	RBC	HS, RT	1506.12	Vinogradov, A.E. (1998a). Genome size and GC-percent in vertebrates as determined by flow cytometry: the triangular relationship. Cytometry 31: 100-109.
Cobitoidei	Cobitidae	*Cobitis*	1.58	FCM	RBC	GD	1545.24	Juchno, D., B. Lackowska, A. Boron, and W. Kilarski (2010). DNA content of hepatocyte and erythrocyte nuclei of the spined loach (Cobitis taenia L.) and its polyploid forms. Fish Physiology and Biochemistry 36: 523-529.
Cobitoidei	Cobitidae	*Cobitis*	1.69	FD	LV	CA	1652.82	Park, E.-H. and C.Y. Chung (1985). Genome and nuclear sizes of Korean cobitid fishes (Teleostomi: Cypriniformes). Korean Journal of Genetics 7: 111-118.
Cobitoidei	Cobitidae	*Cobitis*	1.76	FCM	RBC	MM	1721.28	Vasil'ev, V.P., A.E. Vinogradov, Y.M. Rozanov, and E.D. Vasil'eva (1999). Cellular DNA content in different forms of the bisexual-unisexual complex of spined loaches of the genus Cobitis and in Luther's spined loach C. lutheri (Cobitidae). Journal of Icht
Cobitoidei	Cobitidae	*Cobitis*	1.76	FCM	RBC	MM	1721.28	Vasil'ev, V.P., A.E. Vinogradov, Y.M. Rozanov, and E.D. Vasil'eva (1999). Cellular DNA content in different forms of the bisexual-unisexual complex of spined loaches of the genus Cobitis and in Luther's spined loach C. lutheri (Cobitidae). Journal of Icht
Cobitoidei	Cobitidae	*Cobitis*	1.95	FCM	RBC	HS, RT	1907.1	Vinogradov, A.E. (1998a). Genome size and GC-percent in vertebrates as determined by flow cytometry: the triangular relationship. Cytometry 31: 100-109.
Cobitoidei	Cobitidae	*Cobitis*	1.97	FCM	RBC	MM	1926.66	Ojima, Y. and K. Yamamoto (1990). Cellular DNA contents of fishes determined by flow cytometry. La Kromosomo II 57: 1871-1888.
Cobitoidei	Cobitidae	*Cobitis*	2.15	FD	RBC	CP	2102.7	Suzuki, A. (1992a). Chromosome and DNA studies of eight species in the family Cobitidae (Pisces, Cypriniformes). La Kromosomo II 67/68: 2275-2282.
Cobitoidei	Cobitidae	*Misgurnus*	1.24	FD	LV	CA	1212.72	Park, E.-H. and C.Y. Chung (1985). Genome and nuclear sizes of Korean cobitid fishes (Teleostomi: Cypriniformes). Korean Journal of Genetics 7: 111-118.
Cobitoidei	Cobitidae	*Misgurnus*	1.37	FD	LV	CA	1339.86	Park, E.-H. and C.Y. Chung (1985). Genome and nuclear sizes of Korean cobitid fishes (Teleostomi: Cypriniformes). Korean Journal of Genetics 7: 111-118.
Cobitoidei	Cobitidae	*Misgurnus*	1.4	BFA	RBC	SP	1369.2	Hinegardner, R. and D.E. Rosen (1972). Cellular DNA content and the evolution of teleostean fishes. American Naturalist 106: 621-644.
Cobitoidei	Cobitidae	*Misgurnus*	1.86	FD	RBC	CP	1819.08	Suzuki, A. (1992a). Chromosome and DNA studies of eight species in the family Cobitidae (Pisces, Cypriniformes). La Kromosomo II 67/68: 2275-2282.
Cobitoidei	Cobitidae	*Misgurnus*	2.6	BCA	S	NS	2542.8	Timofeeva, M.Y. and K.A. Kaviani (1964). Nucleic acids of unfertilized eggs and developing groundling embryos. Biokimiia 29: 96-100.
Cobitoidei	Cobitidae	*Pangio*	1	FD	RBC	CP	978	Suzuki, A. (1992a). Chromosome and DNA studies of eight species in the family Cobitidae (Pisces, Cypriniformes). La Kromosomo II 67/68: 2275-2282.
Cobitoidei	Cobitidae	*Pangio*	1.01	FD	RBC	HS	987.78	Muramoto, J., S. Ohno, and N.B. Atkin (1968). On the diploid state of the fish order Ostariophysi. Chromosoma 24: 59-66.
Cobitoidei	Cobitidae	*Pangio*	1.2	BFA	RBC	SP	1173.6	Hinegardner, R. and D.E. Rosen (1972). Cellular DNA content and the evolution of teleostean fishes. American Naturalist 106: 621-644.
Cobitoidei	Gastromyzontidae	*Pseudogastromyzon*	0.44	FD	RBC	CP	430.32	Suzuki, A. and Y. Taki (1991). Karyological and cytochemical studies of Pseudogastromyzon myersi and Homaloptera hoffmanni in the order Cypriniformes. Chromosome Information Service 52: 32-34.
Cobitoidei	Nemacheilidae	*Barbatula*	0.54	FD	RBC	CP	528.12	Suzuki, A. (1992a). Chromosome and DNA studies of eight species in the family Cobitidae (Pisces, Cypriniformes). La Kromosomo II 67/68: 2275-2282.
Cobitoidei	Nemacheilidae	*Barbatula*	0.74	FD	LV	CA	723.72	Park, E.-H. and C.Y. Chung (1985). Genome and nuclear sizes of Korean cobitid fishes (Teleostomi: Cypriniformes). Korean Journal of Genetics 7: 111-118.
Cobitoidei	Nemacheilidae	*Lefua*	0.48	FD	RBC	CP	469.44	Suzuki, A. (1992a). Chromosome and DNA studies of eight species in the family Cobitidae (Pisces, Cypriniformes). La Kromosomo II 67/68: 2275-2282.
Cobitoidei	Nemacheilidae	*Lefua*	1.2	FD	LV	CA	1173.6	Park, E.-H. and C.Y. Chung (1985). Genome and nuclear sizes of Korean cobitid fishes (Teleostomi: Cypriniformes). Korean Journal of Genetics 7: 111-118.
Cyprinoidei	Acheilognathidae	*Acheilognathus*	0.9	FD	RBC	CP	880.2	Suzuki, A., Y. Taki, M. Mochizuki, and J. Hirata (1995). Chromosomal speciation in Eurasian and Japanese Cyprinidae (Pisces, Cypriniformes). Cytobios 83: 171-186.
Cyprinoidei	Acheilognathidae	*Acheilognathus*	0.94	FD	LV, BR	CA	919.32	Ojima, Y., M. Hayashi, and K. Ueno (1972). Cytogenetic studies in lower vertebrates. X. Karyotype and DNA studies in 15 species of Japanese Cyprinidae. Japanese Journal of Genetics 47: 431-440.
Cyprinoidei	Acheilognathidae	*Acheilognathus*	0.95	FD	RBC	CP	929.1	Suzuki, A., Y. Taki, M. Mochizuki, and J. Hirata (1995). Chromosomal speciation in Eurasian and Japanese Cyprinidae (Pisces, Cypriniformes). Cytobios 83: 171-186.
Cyprinoidei	Acheilognathidae	*Acheilognathus*	0.95	FD	RBC	CP	929.1	Suzuki, A., Y. Taki, M. Mochizuki, and J. Hirata (1995). Chromosomal speciation in Eurasian and Japanese Cyprinidae (Pisces, Cypriniformes). Cytobios 83: 171-186.
Cyprinoidei	Acheilognathidae	*Acheilognathus*	1	FD	RBC	GD	978	Cui, J., X. Ren, and Q. Yu (1991). Nuclear DNA content variation in fishes. Cytologia 56: 425-429.

Cyprinoidei	Acheilognathidae	*Acheilognathus*	1.02	FD	LV, BR	CA	997.56	Ojima, Y., M. Hayashi, and K. Ueno (1972). Cytogenetic studies in lower vertebrates. X. Karyotype and DNA studies in 15 species of Japanese Cyprinidae. Japanese Journal of Genetics 47: 431-440.
Cyprinoidei	Acheilognathidae	*Acheilognathus*	1.06	FCM	RBC	MM	1036.68	Ojima, Y. and K. Yamamoto (1990). Cellular DNA contents of fishes determined by flow cytometry. La Kromosomo II 57: 1871-1888.
Cyprinoidei	Acheilognathidae	*Acheilognathus*	1.16	FCM	RBC	MM	1134.48	Ojima, Y. and K. Yamamoto (1990). Cellular DNA contents of fishes determined by flow cytometry. La Kromosomo II 57: 1871-1888.
Cyprinoidei	Acheilognathidae	*Rhodeus*	0.95	FD	RBC	CP	929.1	Suzuki, A., Y. Taki, M. Mochizuki, and J. Hirata (1995). Chromosomal speciation in Eurasian and Japanese Cyprinidae (Pisces, Cypriniformes). Cytobios 83: 171-186.
Cyprinoidei	Acheilognathidae	*Rhodeus*	1	FD	LV, BR	CA	978	Ojima, Y., M. Hayashi, and K. Ueno (1972). Cytogenetic studies in lower vertebrates. X. Karyotype and DNA studies in 15 species of Japanese Cyprinidae. Japanese Journal of Genetics 47: 431-440.
Cyprinoidei	Acheilognathidae	*Rhodeus*	1.06	FD	RBC	HS	1036.68	Hafez, R., R. Labat, and R. Quillier (1978). Teneurs nuclÃ©aires en A.D.N. et relations Ã©volutives dans la famille des cyprinides (Teleostei). Bulletin de la SocietÃ© d'Histoire Naturelle de Toulouse 114: 71-84.
Cyprinoidei	Acheilognathidae	*Rhodeus*	1.07	FCM	RBC	GD	1046.46	Moreira da Costa, L., M.J. Collares-Pereira, S. Lusk, and P. Rab (1999). New data on genome size variation of Centro-European Cyprinidae (Pisces, Osteichthyes). poster, Barcelona.
Cyprinoidei	Acheilognathidae	*Rhodeus*	1.12	FCM	RBC	MM	1095.36	Ojima, Y. and K. Yamamoto (1990). Cellular DNA contents of fishes determined by flow cytometry. La Kromosomo II 57: 1871-1888.
Cyprinoidei	Cyprinidae	*Acrossocheilus*	1.25	FD	RBC	NS	1222.5	Zan, R., Z. Song, and W. Liu (1986). Studies on karyotypes and nuclear DNA contents of some cyprinoid fishes, with notes on fish polyploids in China. In: Indo-Pacific Fish Biology, edited by T. Uyeno, R. Arai, T. Taniuchi and K. Matsuura. Ichthyological S
Cyprinoidei	Cyprinidae	*Barbodes*	0.87	FCM	RBC	HS, RT	850.86	Vinogradov, A.E. (1998a). Genome size and GC-percent in vertebrates as determined by flow cytometry: the triangular relationship. Cytometry 31: 100-109.
Cyprinoidei	Cyprinidae	*Barbodes*	0.89	FCM	RBC	HS, RT	870.42	Vinogradov, A.E. (1998a). Genome size and GC-percent in vertebrates as determined by flow cytometry: the triangular relationship. Cytometry 31: 100-109.
Cyprinoidei	Cyprinidae	*Barbodes*	0.99	BFA	RBC	SP	968.22	Hinegardner, R. and D.E. Rosen (1972). Cellular DNA content and the evolution of teleostean fishes. American Naturalist 106: 621-644.
Cyprinoidei	Cyprinidae	*Barbodes*	1	BFA	RBC	SP	978	Hinegardner, R. and D.E. Rosen (1972). Cellular DNA content and the evolution of teleostean fishes. American Naturalist 106: 621-644.
Cyprinoidei	Cyprinidae	*Barbonymus*	1.1	BFA	RBC	SP	1075.8	Hinegardner, R. and D.E. Rosen (1972). Cellular DNA content and the evolution of teleostean fishes. American Naturalist 106: 621-644.
Cyprinoidei	Cyprinidae	*Barbonymus*	1.1	FD	RBC	CP	1075.8	Suzuki, A., Y. Taki, M. Mochizuki, and J. Hirata (1995). Chromosomal speciation in Eurasian and Japanese Cyprinidae (Pisces, Cypriniformes). Cytobios 83: 171-186.
Cyprinoidei	Cyprinidae	*Ceratogarra*	1.1	BFA	RBC	SP	1075.8	Hinegardner, R. and D.E. Rosen (1972). Cellular DNA content and the evolution of teleostean fishes. American Naturalist 106: 621-644.
Cyprinoidei	Cyprinidae	*Epalzeorhynchos*	1.22	FIA	RBC	BS, GD, OM, RP	1193.16	Hardie, D.C. and P.D.N. Hebert (2003). The nucleotypic effects of cellular DNA content in cartilaginous and ray-finned fishes. Genome 46: 683-706.; Hardie, D.C. and P.D.N. Hebert (2004). Genome-size evolution in fishes. Canadian Journal of Fisheries and Aquatic Sciences 61: 1636-1646. Click here to download data appendix.
Cyprinoidei	Cyprinidae	*Epalzeorhynchos*	1.23	FIA	RBC	BS, GD, OM, RP	1202.94	Hardie, D.C. and P.D.N. Hebert (2003). The nucleotypic effects of cellular DNA content in cartilaginous and ray-finned fishes. Genome 46: 683-706.; Hardie, D.C. and P.D.N. Hebert (2004). Genome-size evolution in fishes. Canadian Journal of Fisheries and Aquatic Sciences 61: 1636-1646. Click here to download data appendix.
Cyprinoidei	Cyprinidae	*Epalzeorhynchos*	1.3	BFA	RBC	SP	1271.4	Hinegardner, R. and D.E. Rosen (1972). Cellular DNA content and the evolution of teleostean fishes. American Naturalist 106: 621-644.
Cyprinoidei	Cyprinidae	*Garra*	1.08	FCM	RBC	GD	1056.24	Kushwaha, B., N. S. Nagpure, S. Srivastava, M. Pandey, R. Kumar, S. Raizada, S. Agarwal, M. Singh, V. S. Basheer, R. G. Kumar, P. Das, S. P. Das, S. Patnaik, A. Bit, S. K. Srivastava, A. L. Vishwakarma, C. G. Joshi, D. Kumar, and J. K. Jena (2023). Genome
Cyprinoidei	Cyprinidae	*Haludaria*	0.77	FD	RBC	HS	753.06	Ohno, S., J. Muramoto, L. Christian, and N.B. Atkin (1967). Diploid-tetraploid relationship among old-world members of the fish family Cyprinidae. Chromosoma 23: 1-9.
Cyprinoidei	Cyprinidae	*Labeo*	0.64	FCM	RBC	GD	625.92	Kushwaha, B., N. S. Nagpure, S. Srivastava, M. Pandey, R. Kumar, S. Raizada, S. Agarwal, M. Singh, V. S. Basheer, R. G. Kumar, P. Das, S. P. Das, S. Patnaik, A. Bit, S. K. Srivastava, A. L. Vishwakarma, C. G. Joshi, D. Kumar, and J. K. Jena (2023). Genome
Cyprinoidei	Cyprinidae	*Labeo*	0.95	FCM	RBC	GD	929.1	Kushwaha, B., N. S. Nagpure, S. Srivastava, M. Pandey, R. Kumar, S. Raizada, S. Agarwal, M. Singh, V. S. Basheer, R. G. Kumar, P. Das, S. P. Das, S. Patnaik, A. Bit, S. K. Srivastava, A. L. Vishwakarma, C. G. Joshi, D. Kumar, and J. K. Jena (2023). Genome
Cyprinoidei	Cyprinidae	*Labeo*	1.04	FD	RBC	ON	1017.12	Patel, A., P. Das, A. Barat, and N. Sarangi (2009). Estimation of genome size in Indian major carps Labeo rohita (Hamilton), Catla catla (Hamilton), Cirrhinus mrigala (Hamilton) and Labeo calbasu (Hamilton) by Feulgen microdensitometry method. Indian Jour
Cyprinoidei	Cyprinidae	*Labeo*	1.05	FCM	RBC	GD	1026.9	Kushwaha, B., N. S. Nagpure, S. Srivastava, M. Pandey, R. Kumar, S. Raizada, S. Agarwal, M. Singh, V. S. Basheer, R. G. Kumar, P. Das, S. P. Das, S. Patnaik, A. Bit, S. K. Srivastava, A. L. Vishwakarma, C. G. Joshi, D. Kumar, and J. K. Jena (2023). Genome
Cyprinoidei	Cyprinidae	*Labeo*	1.1	FIA	RBC	BS, GD, OM, RP	1075.8	Hardie, D.C. and P.D.N. Hebert (2003). The nucleotypic effects of cellular DNA content in cartilaginous and ray-finned fishes. Genome 46: 683-706.; Hardie, D.C. and P.D.N. Hebert (2004). Genome-size evolution in fishes. Canadian Journal of Fisheries and Aquatic Sciences 61: 1636-1646. Click here to download data appendix.
Cyprinoidei	Cyprinidae	*Labeo*	1.2	BFA	RBC	SP	1173.6	Hinegardner, R. and D.E. Rosen (1972). Cellular DNA content and the evolution of teleostean fishes. American Naturalist 106: 621-644.
Cyprinoidei	Cyprinidae	*Labeo*	1.22	FCM	RBC	GD	1193.16	Kushwaha, B., N. S. Nagpure, S. Srivastava, M. Pandey, R. Kumar, S. Raizada, S. Agarwal, M. Singh, V. S. Basheer, R. G. Kumar, P. Das, S. P. Das, S. Patnaik, A. Bit, S. K. Srivastava, A. L. Vishwakarma, C. G. Joshi, D. Kumar, and J. K. Jena (2023). Genome
Cyprinoidei	Cyprinidae	*Labeo*	1.36	FCM	RBC	GD	1330.08	Kushwaha, B., N. S. Nagpure, S. Srivastava, M. Pandey, R. Kumar, S. Raizada, S. Agarwal, M. Singh, V. S. Basheer, R. G. Kumar, P. Das, S. P. Das, S. Patnaik, A. Bit, S. K. Srivastava, A. L. Vishwakarma, C. G. Joshi, D. Kumar, and J. K. Jena (2023). Genome
Cyprinoidei	Cyprinidae	*Labeo*	1.4	FD	RBC	HS	1369.2	Muramoto, J., S. Ohno, and N.B. Atkin (1968). On the diploid state of the fish order Ostariophysi. Chromosoma 24: 59-66.
Cyprinoidei	Cyprinidae	*Labeo*	1.45	FCM	RBC	GD	1418.1	Kushwaha, B., N. S. Nagpure, S. Srivastava, M. Pandey, R. Kumar, S. Raizada, S. Agarwal, M. Singh, V. S. Basheer, R. G. Kumar, P. Das, S. P. Das, S. Patnaik, A. Bit, S. K. Srivastava, A. L. Vishwakarma, C. G. Joshi, D. Kumar, and J. K. Jena (2023). Genome
Cyprinoidei	Cyprinidae	*Labeo*	1.99	FD	RBC	ON	1946.22	Patel, A., P. Das, A. Barat, and N. Sarangi (2009). Estimation of genome size in Indian major carps Labeo rohita (Hamilton), Catla catla (Hamilton), Cirrhinus mrigala (Hamilton) and Labeo calbasu (Hamilton) by Feulgen microdensitometry method. Indian Jour
Cyprinoidei	Cyprinidae	*Labeo*	2.45	FD	RBC	ON	2396.1	Patel, A., P. Das, A. Barat, and N. Sarangi (2009). Estimation of genome size in Indian major carps Labeo rohita (Hamilton), Catla catla (Hamilton), Cirrhinus mrigala (Hamilton) and Labeo calbasu (Hamilton) by Feulgen microdensitometry method. Indian Jour
Cyprinoidei	Cyprinidae	*Paraspinibarbus*	0.98	BFA	RBC	SP	958.44	Hinegardner, R. and D.E. Rosen (1972). Cellular DNA content and the evolution of teleostean fishes. American Naturalist 106: 621-644.
Cyprinoidei	Cyprinidae	*Paraspinibarbus*	1.08	FCM	RBC	MM	1056.24	Ojima, Y. and K. Yamamoto (1990). Cellular DNA contents of fishes determined by flow cytometry. La Kromosomo II 57: 1871-1888.
Cyprinoidei	Cyprinidae	*Pethia*	0.7	FCM	RBC	GD	684.6	Kushwaha, B., N. S. Nagpure, S. Srivastava, M. Pandey, R. Kumar, S. Raizada, S. Agarwal, M. Singh, V. S. Basheer, R. G. Kumar, P. Das, S. P. Das, S. Patnaik, A. Bit, S. K. Srivastava, A. L. Vishwakarma, C. G. Joshi, D. Kumar, and J. K. Jena (2023). Genome
Cyprinoidei	Cyprinidae	*Pethia*	0.84	FCM	RBC	HS, RT	821.52	Vinogradov, A.E. (1998a). Genome size and GC-percent in vertebrates as determined by flow cytometry: the triangular relationship. Cytometry 31: 100-109.
Cyprinoidei	Cyprinidae	*Pethia*	0.91	FCM	RBC	HS, RT	889.98	Vinogradov, A.E. (1998a). Genome size and GC-percent in vertebrates as determined by flow cytometry: the triangular relationship. Cytometry 31: 100-109.
Cyprinoidei	Cyprinidae	*Pethia*	0.97	BFA	RBC	SP	948.66	Hinegardner, R. and D.E. Rosen (1972). Cellular DNA content and the evolution of teleostean fishes. American Naturalist 106: 621-644.
Cyprinoidei	Cyprinidae	*Pethia*	0.99	FCM	RBC	MM	968.22	Ojima, Y. and K. Yamamoto (1990). Cellular DNA contents of fishes determined by flow cytometry. La Kromosomo II 57: 1871-1888.

Cyprinoidei	Cyprinidae	*Poropuntius*	1.15	FD	RBC	NS	1124.7	Zan, R., Z. Song, and W. Liu (1986). Studies on karyotypes and nuclear DNA contents of some cyprinoid fishes, with notes on fish polyploids in China. In: Indo-Pacific Fish Biology, edited by T. Uyeno, R. Arai, T. Taniuchi and K. Matsuura. Ichthyological S
Cyprinoidei	Cyprinidae	*Puntigrus*	0.7	FD	RBC	HS	684.6	Wolf, U., H. Ritter, N.B. Atkin, and S. Ohno (1969). Polyploidization in the fish family Cyprinidae, Order Cypriniformes. I. DNA-content and chromosome sets in various species of Cyprinidae. Humangenetik 7: 240-244.
Cyprinoidei	Cyprinidae	*Puntigrus*	0.71	FD	RBC	HS	694.38	Ohno, S., J. Muramoto, L. Christian, and N.B. Atkin (1967). Diploid-tetraploid relationship among old-world members of the fish family Cyprinidae. Chromosoma 23: 1-9.
Cyprinoidei	Cyprinidae	*Puntigrus*	0.75	FCM	RBC	HS, RT	733.5	Vinogradov, A.E. (1998a). Genome size and GC-percent in vertebrates as determined by flow cytometry: the triangular relationship. Cytometry 31: 100-109.
Cyprinoidei	Cyprinidae	*Puntigrus*	0.96	BFA	RBC	SP	938.88	Hinegardner, R. and D.E. Rosen (1972). Cellular DNA content and the evolution of teleostean fishes. American Naturalist 106: 621-644.
Cyprinoidei	Cyprinidae	*Puntigrus*	1	FD	RBC	CP	978	Suzuki, A., Y. Taki, M. Mochizuki, and J. Hirata (1995). Chromosomal speciation in Eurasian and Japanese Cyprinidae (Pisces, Cypriniformes). Cytobios 83: 171-186.
Cyprinoidei	Cyprinidae	*Puntius*	0.71	FCM	RBC	GD	694.38	Kushwaha, B., N. S. Nagpure, S. Srivastava, M. Pandey, R. Kumar, S. Raizada, S. Agarwal, M. Singh, V. S. Basheer, R. G. Kumar, P. Das, S. P. Das, S. Patnaik, A. Bit, S. K. Srivastava, A. L. Vishwakarma, C. G. Joshi, D. Kumar, and J. K. Jena (2023). Genome
Cyprinoidei	Cyprinidae	*Rohanella*	1.2	BFA	RBC	SP	1173.6	Hinegardner, R. and D.E. Rosen (1972). Cellular DNA content and the evolution of teleostean fishes. American Naturalist 106: 621-644.
Cyprinoidei	Cyprinidae	*Sahyadria*	1.26	FCM	RBC	GD	1232.28	Kushwaha, B., N. S. Nagpure, S. Srivastava, M. Pandey, R. Kumar, S. Raizada, S. Agarwal, M. Singh, V. S. Basheer, R. G. Kumar, P. Das, S. P. Das, S. Patnaik, A. Bit, S. K. Srivastava, A. L. Vishwakarma, C. G. Joshi, D. Kumar, and J. K. Jena (2023). Genome
Cyprinoidei	Cyprinidae	*Systomus*	0.67	FCM	RBC	GD	655.26	Kushwaha, B., N. S. Nagpure, S. Srivastava, M. Pandey, R. Kumar, S. Raizada, S. Agarwal, M. Singh, V. S. Basheer, R. G. Kumar, P. Das, S. P. Das, S. Patnaik, A. Bit, S. K. Srivastava, A. L. Vishwakarma, C. G. Joshi, D. Kumar, and J. K. Jena (2023). Genome
Cyprinoidei	Cyprinidae	*Systomus*	0.77	FD	RBC	CP	753.06	Suzuki, A. and Y. Taki (1988). Karyotype and DNA content in the cyprinid Catlocarpio siamensis. Japanese Journal of Ichthyology 35: 389-391.
Cyprinoidei	Cyprinidae	*Tor*	1.21	FCM	RBC	GD	1183.38	Kushwaha, B., N. S. Nagpure, S. Srivastava, M. Pandey, R. Kumar, S. Raizada, S. Agarwal, M. Singh, V. S. Basheer, R. G. Kumar, P. Das, S. P. Das, S. Patnaik, A. Bit, S. K. Srivastava, A. L. Vishwakarma, C. G. Joshi, D. Kumar, and J. K. Jena (2023). Genome
Cyprinoidei	Danionidae	*Amblypharyngodon*	1.22	FCM	RBC	GD	1193.16	Kushwaha, B., N. S. Nagpure, S. Srivastava, M. Pandey, R. Kumar, S. Raizada, S. Agarwal, M. Singh, V. S. Basheer, R. G. Kumar, P. Das, S. P. Das, S. Patnaik, A. Bit, S. K. Srivastava, A. L. Vishwakarma, C. G. Joshi, D. Kumar, and J. K. Jena (2023). Genome
Cyprinoidei	Danionidae	*Barilius*	1.22	FCM	RBC	GD	1193.16	Kushwaha, B., N. S. Nagpure, S. Srivastava, M. Pandey, R. Kumar, S. Raizada, S. Agarwal, M. Singh, V. S. Basheer, R. G. Kumar, P. Das, S. P. Das, S. Patnaik, A. Bit, S. K. Srivastava, A. L. Vishwakarma, C. G. Joshi, D. Kumar, and J. K. Jena (2023). Genome
Cyprinoidei	Danionidae	*Danio*	1.42	FD	RBC	CP	1388.76	Suzuki, A., Y. Taki, M. Mochizuki, and J. Hirata (1995). Chromosomal speciation in Eurasian and Japanese Cyprinidae (Pisces, Cypriniformes). Cytobios 83: 171-186.
Cyprinoidei	Danionidae	*Danio*	1.68	FCM	RC	HS	1643.04	Ciudad, J., E. Cid, A. Velasco, J.M. Lara, J. AijÃ³n, and A. Orfao (2002). Flow cytometry measurement of the DNA contents of G0/G1 diploid cells from three different teleost fish species. Cytometry 48: 20-25.
Cyprinoidei	Danionidae	*Danio*	1.75	FCM	RBC	HS, RT	1711.5	Vinogradov, A.E. (1998a). Genome size and GC-percent in vertebrates as determined by flow cytometry: the triangular relationship. Cytometry 31: 100-109.
Cyprinoidei	Danionidae	*Danio*	1.78	FCM	RBC	MM	1740.84	Ojima, Y. and K. Yamamoto (1990). Cellular DNA contents of fishes determined by flow cytometry. La Kromosomo II 57: 1871-1888.
Cyprinoidei	Danionidae	*Danio*	1.8	BFA	RBC	SP	1760.4	Hinegardner, R. (1968). Evolution of cellular DNA content in teleost fishes. American Naturalist 102: 517-523.; Hinegardner, R. and D.E. Rosen (1972). Cellular DNA content and the evolution of teleostean fishes. American Naturalist 106: 621-644.
Cyprinoidei	Danionidae	*Devario*	0.79	FCM	RBC	GD	772.62	Kushwaha, B., N. S. Nagpure, S. Srivastava, M. Pandey, R. Kumar, S. Raizada, S. Agarwal, M. Singh, V. S. Basheer, R. G. Kumar, P. Das, S. P. Das, S. Patnaik, A. Bit, S. K. Srivastava, A. L. Vishwakarma, C. G. Joshi, D. Kumar, and J. K. Jena (2023). Genome
Cyprinoidei	Danionidae	*Devario*	1.4	FIA	RBC	BS, GD, OM, RP	1369.2	Hardie, D.C. and P.D.N. Hebert (2004). Genome-size evolution in fishes. Canadian Journal of Fisheries and Aquatic Sciences 61: 1636-1646. Click here to download data appendix.
Cyprinoidei	Danionidae	*Laubuka*	1.6	BFA	RBC	SP	1564.8	Hinegardner, R. (1968). Evolution of cellular DNA content in teleost fishes. American Naturalist 102: 517-523.; Hinegardner, R. and D.E. Rosen (1972). Cellular DNA content and the evolution of teleostean fishes. American Naturalist 106: 621-644.
Cyprinoidei	Danionidae	*Opsarius*	1.22	FCM	RBC	GD	1193.16	Kushwaha, B., N. S. Nagpure, S. Srivastava, M. Pandey, R. Kumar, S. Raizada, S. Agarwal, M. Singh, V. S. Basheer, R. G. Kumar, P. Das, S. P. Das, S. Patnaik, A. Bit, S. K. Srivastava, A. L. Vishwakarma, C. G. Joshi, D. Kumar, and J. K. Jena (2023). Genome
Cyprinoidei	Danionidae	*Rasbora*	0.85	FCM	RBC	GD	831.3	Kushwaha, B., N. S. Nagpure, S. Srivastava, M. Pandey, R. Kumar, S. Raizada, S. Agarwal, M. Singh, V. S. Basheer, R. G. Kumar, P. Das, S. P. Das, S. Patnaik, A. Bit, S. K. Srivastava, A. L. Vishwakarma, C. G. Joshi, D. Kumar, and J. K. Jena (2023). Genome
Cyprinoidei	Danionidae	*Rasbora*	1.44	FD	RBC	CP	1408.32	Suzuki, A., Y. Taki, M. Mochizuki, and J. Hirata (1995). Chromosomal speciation in Eurasian and Japanese Cyprinidae (Pisces, Cypriniformes). Cytobios 83: 171-186.
Cyprinoidei	Gobionidae	*Hemibarbus*	0.95	FD	RBC	CP	929.1	Suzuki, A., Y. Taki, M. Mochizuki, and J. Hirata (1995). Chromosomal speciation in Eurasian and Japanese Cyprinidae (Pisces, Cypriniformes). Cytobios 83: 171-186.
Cyprinoidei	Gobionidae	*Hemibarbus*	1.05	FD	LV	CA	1026.9	Kang, Y.S. and E.H. Park (1973). Studies on the karyotypes and comparative DNA values in several Korean cyprinid fishes. Korean Journal of Zoology 16: 97-108.
Cyprinoidei	Gobionidae	*Hemibarbus*	1.08	FD	LV	CA	1056.24	Kang, Y.S. and E.H. Park (1973). Studies on the karyotypes and comparative DNA values in several Korean cyprinid fishes. Korean Journal of Zoology 16: 97-108.
Cyprinoidei	Gobionidae	*Hemibarbus*	1.15	FD	RBC	GD	1124.7	Cui, J., X. Ren, and Q. Yu (1991). Nuclear DNA content variation in fishes. Cytologia 56: 425-429.
Cyprinoidei	Gobionidae	*Hemibarbus*	1.17	FD	RBC	GD	1144.26	Cui, J., X. Ren, and Q. Yu (1991). Nuclear DNA content variation in fishes. Cytologia 56: 425-429.
Cyprinoidei	Gobionidae	*Hemibarbus*	1.28	FCM	RBC	MM	1251.84	Ojima, Y. and K. Yamamoto (1990). Cellular DNA contents of fishes determined by flow cytometry. La Kromosomo II 57: 1871-1888.
Cyprinoidei	Gobionidae	*Sarcocheilichthys*	1.2	FD	RBC	CP	1173.6	Suzuki, A., Y. Taki, M. Mochizuki, and J. Hirata (1995). Chromosomal speciation in Eurasian and Japanese Cyprinidae (Pisces, Cypriniformes). Cytobios 83: 171-186.
Cyprinoidei	Gobionidae	*Sarcocheilichthys*	1.21	FD	LV, BR	CA	1183.38	Ojima, Y., M. Hayashi, and K. Ueno (1972). Cytogenetic studies in lower vertebrates. X. Karyotype and DNA studies in 15 species of Japanese Cyprinidae. Japanese Journal of Genetics 47: 431-440.
Cyprinoidei	Gobionidae	*Sarcocheilichthys*	1.21	FD	RBC	CP	1183.38	Zhou, T. (1984). Chromosome studies in Chinese freshwater fishes [in Chinese]. Zoological Research (Tung Wu Hsueh Yen Chiu) 5: 38-51.
Cyprinoidei	Gobionidae	*Sarcocheilichthys*	1.25	FD	RBC	HS	1222.5	Li, Y., K. Li, and T. Zhou (1983). Cellular DNA content of fourteen species of freshwater fishes [in Chinese]. Acta Genetica Sinica 10: 384-389.
Cyprinoidei	Gobionidae	*Sarcocheilichthys*	1.39	FCM	RBC	MM	1359.42	Ojima, Y. and K. Yamamoto (1990). Cellular DNA contents of fishes determined by flow cytometry. La Kromosomo II 57: 1871-1888.
Cyprinoidei	Gobionidae	*Sarcocheilichthys*	1.54	FD	RBC	GD	1506.12	Cui, J., X. Ren, and Q. Yu (1991). Nuclear DNA content variation in fishes. Cytologia 56: 425-429.
Cyprinoidei	Gobionidae	*Squalidus*	1.25	FD	RBC	CP	1222.5	Suzuki, A., Y. Taki, M. Mochizuki, and J. Hirata (1995). Chromosomal speciation in Eurasian and Japanese Cyprinidae (Pisces, Cypriniformes). Cytobios 83: 171-186.
Cyprinoidei	Leuciscidae	*Chrosomus*	1.27	FCM	RBC	GD	1242.06	Goddard, K.A., O. Megwinoff, L.L. Wessner, and F. Giaimo (1998). Confirmation of gynogenesis in Phoxinus eos-neogaeus (Pisces: Cyprinidae). Journal of Heredity 89: 151-157.
Cyprinoidei	Leuciscidae	*Chrosomus*	1.32	FD	RBC	GD	1290.96	Gold, J.R. and C.T. Amemiya (1987). Genome size variation in North American minnows (Cyprinidae). II. Variation among 20 species. Genome 29: 481-489.
Cyprinoidei	Leuciscidae	*Chrosomus*	1.41	FIA	RBC	BS, GD, OM, RP	1378.98	Hardie, D.C. and P.D.N. Hebert (2003). The nucleotypic effects of cellular DNA content in cartilaginous and ray-finned fishes. Genome 46: 683-706.; Hardie, D.C. and P.D.N. Hebert (2004). Genome-size evolution in fishes. Canadian Journal of Fisheries and Aquatic Sciences 61: 1636-1646. Click here to download data appendix.

Cyprinoidei	Leuciscidae	*Chrosomus*	1.42	FCM	RBC	GD	1388.76	Goddard, K.A., O. Megwinoff, L.L. Wessner, and F. Giaimo (1998). Confirmation of gynogenesis in Phoxinus eos-neogaeus (Pisces: Cyprinidae). Journal of Heredity 89: 151-157.
Cyprinoidei	Leuciscidae	*Chrosomus*	1.57	FCM	RBC	GD	1535.46	Goddard, K.A., O. Megwinoff, L.L. Wessner, and F. Giaimo (1998). Confirmation of gynogenesis in Phoxinus eos-neogaeus (Pisces: Cyprinidae). Journal of Heredity 89: 151-157.
Cyprinoidei	Leuciscidae	*Chrosomus*	1.64	FCM	RBC	GD	1603.92	Dawley, R.M. and K.A. Goddard (1988). Diploid-triploid mosaics among unisexual hybrids of the minnows Phoxinus eos and Phoxinus neogaeus. Evolution 42: 649-659.
Cyprinoidei	Leuciscidae	*Chrosomus*	1.75	FCM	RBC	GD	1711.5	Dawley, R.M. and K.A. Goddard (1988). Diploid-triploid mosaics among unisexual hybrids of the minnows Phoxinus eos and Phoxinus neogaeus. Evolution 42: 649-659.
Cyprinoidei	Leuciscidae	*Chrosomus*	1.83	FCM	RBC	GD	1789.74	Dawley, R.M. and K.A. Goddard (1988). Diploid-triploid mosaics among unisexual hybrids of the minnows Phoxinus eos and Phoxinus neogaeus. Evolution 42: 649-659.
Cyprinoidei	Leuciscidae	*Chrosomus*	2.13	FCM	RBC	GD	2083.14	Goddard, K.A., O. Megwinoff, L.L. Wessner, and F. Giaimo (1998). Confirmation of gynogenesis in Phoxinus eos-neogaeus (Pisces: Cyprinidae). Journal of Heredity 89: 151-157.
Cyprinoidei	Leuciscidae	*Chrosomus*	2.14	FIA	RBC	BS, GD, OM, RP	2092.92	Hardie, D.C. and P.D.N. Hebert (2003). The nucleotypic effects of cellular DNA content in cartilaginous and ray-finned fishes. Genome 46: 683-706.; Hardie, D.C. and P.D.N. Hebert (2004). Genome-size evolution in fishes. Canadian Journal of Fisheries and Aquatic Sciences 61: 1636-1646. Click here to download data appendix.
Cyprinoidei	Leuciscidae	*Chrosomus*	2.49	FCM	RBC	GD	2435.22	Dawley, R.M. and K.A. Goddard (1988). Diploid-triploid mosaics among unisexual hybrids of the minnows Phoxinus eos and Phoxinus neogaeus. Evolution 42: 649-659.
Cyprinoidei	Leuciscidae	*Cyprinella*	1.03	FD	RBC	GD	1007.34	Gold, J.R., C.J. Ragland, and L.J. Schliesing (1990). Genome size variation and evolution in North American cyprinid fishes. Genetics, Selection, Evolution 22: 11-29.
Cyprinoidei	Leuciscidae	*Cyprinella*	1.14	FD	RBC	GD	1114.92	Gold, J.R., C.J. Ragland, and L.J. Schliesing (1990). Genome size variation and evolution in North American cyprinid fishes. Genetics, Selection, Evolution 22: 11-29.
Cyprinoidei	Leuciscidae	*Cyprinella*	1.19	FD	RBC	GD	1163.82	Gold, J.R. and H.J. Price (1985). Genome size variation among North American minnows (Cyprinidae). I. Distribution of the variation in five species. Heredity 54: 297-305.
Cyprinoidei	Leuciscidae	*Cyprinella*	1.2	FD	RBC	GD	1173.6	Gold, J.R., C.J. Ragland, and L.J. Schliesing (1990). Genome size variation and evolution in North American cyprinid fishes. Genetics, Selection, Evolution 22: 11-29.
Cyprinoidei	Leuciscidae	*Cyprinella*	1.21	FD	RBC	GD	1183.38	Gold, J.R. and H.J. Price (1985). Genome size variation among North American minnows (Cyprinidae). I. Distribution of the variation in five species. Heredity 54: 297-305.
Cyprinoidei	Leuciscidae	*Cyprinella*	1.25	FD	RBC	GD	1222.5	Gold, J.R. and C.T. Amemiya (1987). Genome size variation in North American minnows (Cyprinidae). II. Variation among 20 species. Genome 29: 481-489.
Cyprinoidei	Leuciscidae	*Cyprinella*	1.27	FD	RBC	GD	1242.06	Gold, J.R., C.J. Ragland, and L.J. Schliesing (1990). Genome size variation and evolution in North American cyprinid fishes. Genetics, Selection, Evolution 22: 11-29.
Cyprinoidei	Leuciscidae	*Cyprinella*	1.45	FD	RBC	GD	1418.1	Gold, J.R., C.J. Ragland, and L.J. Schliesing (1990). Genome size variation and evolution in North American cyprinid fishes. Genetics, Selection, Evolution 22: 11-29.
Cyprinoidei	Leuciscidae	*Dionda*	0.99	FCM	RBC	GD, CP	968.22	Gold, J.R., Y. Li, M.C. Birkner, and J.D. Jenkin (1992). Chromosomal NOR karyotypes and genome sizes in Dionda (Osteichthyes: Cyprinidae) from Texas and New Mexico. Southwestern Naturalist 37: 217-222.
Cyprinoidei	Leuciscidae	*Dionda*	1	FCM	RBC	GD, CP	978	Gold, J.R., Y. Li, M.C. Birkner, and J.D. Jenkin (1992). Chromosomal NOR karyotypes and genome sizes in Dionda (Osteichthyes: Cyprinidae) from Texas and New Mexico. Southwestern Naturalist 37: 217-222.
Cyprinoidei	Leuciscidae	*Dionda*	1.01	FCM	RBC	GD, CP	987.78	Gold, J.R., Y. Li, M.C. Birkner, and J.D. Jenkin (1992). Chromosomal NOR karyotypes and genome sizes in Dionda (Osteichthyes: Cyprinidae) from Texas and New Mexico. Southwestern Naturalist 37: 217-222.
Cyprinoidei	Leuciscidae	*Dionda*	1.04	FCM	RBC	GD, CP	1017.12	Gold, J.R., Y. Li, M.C. Birkner, and J.D. Jenkin (1992). Chromosomal NOR karyotypes and genome sizes in Dionda (Osteichthyes: Cyprinidae) from Texas and New Mexico. Southwestern Naturalist 37: 217-222.
Cyprinoidei	Leuciscidae	*Dionda*	1.04	FCM	RBC	GD, CP	1017.12	Gold, J.R., Y. Li, M.C. Birkner, and J.D. Jenkin (1992). Chromosomal NOR karyotypes and genome sizes in Dionda (Osteichthyes: Cyprinidae) from Texas and New Mexico. Southwestern Naturalist 37: 217-222.
Cyprinoidei	Leuciscidae	*Dionda*	1.07	FD	RBC	GD	1046.46	Gold, J.R., C.J. Ragland, and L.J. Schliesing (1990). Genome size variation and evolution in North American cyprinid fishes. Genetics, Selection, Evolution 22: 11-29.
Cyprinoidei	Leuciscidae	*Hybognathus*	1.41	FD	RBC	GD	1378.98	Gold, J.R., C.J. Ragland, and L.J. Schliesing (1990). Genome size variation and evolution in North American cyprinid fishes. Genetics, Selection, Evolution 22: 11-29.
Cyprinoidei	Leuciscidae	*Leuciscus*	1.19	SCF	RBC	HS	1163.82	De Smet, W.H.O. (1981). The nuclear Feulgen-DNA content of the vertebrates (especially reptiles), as measured by fluorescence cytophotometry, with notes on the cell and chromosome size. Acta Zoologica et Pathologica Antverpiensia 76: 119-167.
Cyprinoidei	Leuciscidae	*Leuciscus*	1.24	FCM	RBC	GD	1212.72	Moreira da Costa, L., M.J. Collares-Pereira, S. Lusk, and P. Rab (1999). New data on genome size variation of Centro-European Cyprinidae (Pisces, Osteichthyes). poster, Barcelona.
Cyprinoidei	Leuciscidae	*Leuciscus*	1.28	FD	RBC	HS	1251.84	Hafez, R., R. Labat, and R. Quillier (1978). Teneurs nuclÃ©aires en A.D.N. et relations Ã©volutives dans la famille des cyprinides (Teleostei). Bulletin de la SocietÃ© d'Histoire Naturelle de Toulouse 114: 71-84.
Cyprinoidei	Leuciscidae	*Leuciscus*	1.3	FCM	RBC	GD	1271.4	Moreira da Costa, L., M.J. Collares-Pereira, S. Lusk, and P. Rab (1999). New data on genome size variation of Centro-European Cyprinidae (Pisces, Osteichthyes). poster, Barcelona.
Cyprinoidei	Leuciscidae	*Leuciscus*	1.5	BFA	RBC	SP	1467	Hinegardner, R. and D.E. Rosen (1972). Cellular DNA content and the evolution of teleostean fishes. American Naturalist 106: 621-644.
Cyprinoidei	Leuciscidae	*Notropis*	1.04	FD	RBC	GD	1017.12	Gold, J.R., C.J. Ragland, and L.J. Schliesing (1990). Genome size variation and evolution in North American cyprinid fishes. Genetics, Selection, Evolution 22: 11-29.
Cyprinoidei	Leuciscidae	*Notropis*	1.1	FD	RBC	GD	1075.8	Gold, J.R. and C.T. Amemiya (1987). Genome size variation in North American minnows (Cyprinidae). II. Variation among 20 species. Genome 29: 481-489.
Cyprinoidei	Leuciscidae	*Notropis*	1.13	FD	RBC	GD	1105.14	Gold, J.R., C.J. Ragland, and L.J. Schliesing (1990). Genome size variation and evolution in North American cyprinid fishes. Genetics, Selection, Evolution 22: 11-29.
Cyprinoidei	Leuciscidae	*Notropis*	1.17	FD	RBC	GD	1144.26	Gold, J.R. and C.T. Amemiya (1987). Genome size variation in North American minnows (Cyprinidae). II. Variation among 20 species. Genome 29: 481-489.
Cyprinoidei	Leuciscidae	*Notropis*	1.18	FD	RBC	GD	1154.04	Gold, J.R., C.J. Ragland, and L.J. Schliesing (1990). Genome size variation and evolution in North American cyprinid fishes. Genetics, Selection, Evolution 22: 11-29.
Cyprinoidei	Leuciscidae	*Notropis*	1.19	FD	RBC	GD	1163.82	Gold, J.R. and C.T. Amemiya (1987). Genome size variation in North American minnows (Cyprinidae). II. Variation among 20 species. Genome 29: 481-489.
Cyprinoidei	Leuciscidae	*Notropis*	1.19	FD	RBC	GD	1163.82	Gold, J.R. and C.T. Amemiya (1987). Genome size variation in North American minnows (Cyprinidae). II. Variation among 20 species. Genome 29: 481-489.
Cyprinoidei	Leuciscidae	*Notropis*	1.19	FD	RBC	GD	1163.82	Gold, J.R., C.J. Ragland, and L.J. Schliesing (1990). Genome size variation and evolution in North American cyprinid fishes. Genetics, Selection, Evolution 22: 11-29.
Cyprinoidei	Leuciscidae	*Notropis*	1.23	FD	RBC	GD	1202.94	Gold, J.R., C.J. Ragland, and L.J. Schliesing (1990). Genome size variation and evolution in North American cyprinid fishes. Genetics, Selection, Evolution 22: 11-29.
Cyprinoidei	Leuciscidae	*Notropis*	1.24	FD	RBC	GD	1212.72	Gold, J.R., C.J. Ragland, and L.J. Schliesing (1990). Genome size variation and evolution in North American cyprinid fishes. Genetics, Selection, Evolution 22: 11-29.
Cyprinoidei	Leuciscidae	*Notropis*	1.25	FD	RBC	GD	1222.5	Gold, J.R., C.J. Ragland, and L.J. Schliesing (1990). Genome size variation and evolution in North American cyprinid fishes. Genetics, Selection, Evolution 22: 11-29.
Cyprinoidei	Leuciscidae	*Notropis*	1.26	FD	RBC	GD	1232.28	Gold, J.R. and C.T. Amemiya (1987). Genome size variation in North American minnows (Cyprinidae). II. Variation among 20 species. Genome 29: 481-489.
Cyprinoidei	Leuciscidae	*Notropis*	1.26	FD	RBC	GD	1232.28	Gold, J.R., C.J. Ragland, and L.J. Schliesing (1990). Genome size variation and evolution in North American cyprinid fishes. Genetics, Selection, Evolution 22: 11-29.
Cyprinoidei	Leuciscidae	*Notropis*	1.27	FD	RBC	GD	1242.06	Gold, J.R., C.J. Ragland, and L.J. Schliesing (1990). Genome size variation and evolution in North American cyprinid fishes. Genetics, Selection, Evolution 22: 11-29.
Cyprinoidei	Leuciscidae	*Notropis*	1.36	FD	RBC	GD	1330.08	Gold, J.R. and C.T. Amemiya (1987). Genome size variation in North American minnows (Cyprinidae). II. Variation among 20 species. Genome 29: 481-489.

Cyprinoidei	Leuciscidae	*Notropis*	1.37	FD	RBC	GD	1339.86	Gold, J.R., C.J. Ragland, and L.J. Schliesing (1990). Genome size variation and evolution in North American cyprinid fishes. Genetics, Selection, Evolution 22: 11-29.
Cyprinoidei	Leuciscidae	*Notropis*	1.4	FD	RBC	GD	1369.2	Gold, J.R., C.J. Ragland, and L.J. Schliesing (1990). Genome size variation and evolution in North American cyprinid fishes. Genetics, Selection, Evolution 22: 11-29.
Cyprinoidei	Leuciscidae	*Notropis*	1.45	FD	RBC	GD	1418.1	Gold, J.R., C.J. Ragland, and L.J. Schliesing (1990). Genome size variation and evolution in North American cyprinid fishes. Genetics, Selection, Evolution 22: 11-29.
Cyprinoidei	Leuciscidae	*Phoxinus*	1.15	FD	RBC	HS	1124.7	Hafez, R., R. Labat, and R. Quillier (1978). Teneurs nuclÃ©aires en A.D.N. et relations Ã©volutives dans la famille des cyprinides (Teleostei). Bulletin de la SocietÃ© d'Histoire Naturelle de Toulouse 114: 71-84.
Cyprinoidei	Leuciscidae	*Ptychocheilus*	1.26	FCM	RBC	GD, CP	1232.28	Gold, J.R. (1994). Cytosystematic evidence that the genus Richardsonius belongs in the western clade of phoxinin cyprinids. Copeia 1994: 815-818.
Cyprinoidei	Leuciscidae	*Ptychocheilus*	1.29	FCM	RBC	GD, CP	1261.62	Gold, J.R. (1994). Cytosystematic evidence that the genus Richardsonius belongs in the western clade of phoxinin cyprinids. Copeia 1994: 815-818.
Cyprinoidei	Leuciscidae	*Ptychocheilus*	1.38	FCM	RBC	GD, CP	1349.64	Gold, J.R. (1994). Cytosystematic evidence that the genus Richardsonius belongs in the western clade of phoxinin cyprinids. Copeia 1994: 815-818.
Cyprinoidei	Leuciscidae	*Rhinichthys*	1.29	FD	RBC	GD	1261.62	Gold, J.R., C.J. Ragland, and L.J. Schliesing (1990). Genome size variation and evolution in North American cyprinid fishes. Genetics, Selection, Evolution 22: 11-29.
Cyprinoidei	Leuciscidae	*Rhynchocypris*	1.02	FD	LV	CA	997.56	Kang, Y.S. and E.H. Park (1973). Studies on the karyotypes and comparative DNA values in several Korean cyprinid fishes. Korean Journal of Zoology 16: 97-108.
Cyprinoidei	Leuciscidae	*Rhynchocypris*	1.11	FD	LV	CA	1085.58	Kang, Y.S. and E.H. Park (1973). Studies on the karyotypes and comparative DNA values in several Korean cyprinid fishes. Korean Journal of Zoology 16: 97-108.
Cyprinoidei	Leuciscidae	*Rhynchocypris*	1.52	FCM	RBC	MM	1486.56	Ojima, Y. and K. Yamamoto (1990). Cellular DNA contents of fishes determined by flow cytometry. La Kromosomo II 57: 1871-1888.
Cyprinoidei	Leuciscidae	*Rutilus*	0.97	FD	RBC	HS	948.66	Hafez, R., R. Labat, and R. Quillier (1978). Teneurs nuclÃ©aires en A.D.N. et relations Ã©volutives dans la famille des cyprinides (Teleostei). Bulletin de la SocietÃ© d'Histoire Naturelle de Toulouse 114: 71-84.
Cyprinoidei	Leuciscidae	*Rutilus*	0.98	FD	RBC	HS	958.44	Wolf, U., H. Ritter, N.B. Atkin, and S. Ohno (1969). Polyploidization in the fish family Cyprinidae, Order Cypriniformes. I. DNA-content and chromosome sets in various species of Cyprinidae. Humangenetik 7: 240-244.
Cyprinoidei	Leuciscidae	*Rutilus*	1.08	BCA	RBC	GD	1056.24	Vendrely, R. and C. Vendrely (1953). Argenine an deoxyribonucleic acid content of erythrocyte nuclei and sperms of some species of fishes. Nature 172: 30-31.; Vendrely, R. and C. Vendrely (1952a). Sur la teneur comparÃ©e en arginine et en acide dÃ©soxyribonuclÃ©ique des noyaux d'Ã©rythrocytes de quelques espÃ¨ces de poissons. Comptes Rendus de l'AcadÃ©mie des Sciences 235: 395-397.
Cyprinoidei	Leuciscidae	*Rutilus*	1.11	BCA	RBC	HS	1085.58	Vendrely, R. and C. Vendrely (1950). Sur la teneur absolue en acide dÃ©soxyribonuclÃ©ique du noyau cellulaire chez quelques espÃ¨ces d'oiseaux et de poissons. Comptes Rendus de l'AcadÃ©mie des Sciences 230: 788-790.
Cyprinoidei	Leuciscidae	*Rutilus*	1.21	FCM	RBC	GD	1183.38	Moreira da Costa, L., M.J. Collares-Pereira, S. Lusk, and P. Rab (1999). New data on genome size variation of Centro-European Cyprinidae (Pisces, Osteichthyes). poster, Barcelona.
Cyprinoidei	Leuciscidae	*Rutilus*	1.32	FCM	RBC	HS, RT	1290.96	Vinogradov, A.E. (1998a). Genome size and GC-percent in vertebrates as determined by flow cytometry: the triangular relationship. Cytometry 31: 100-109.
Cyprinoidei	Leuciscidae	*Rutilus*	1.51	SCF	RBC	HS	1476.78	De Smet, W.H.O. (1981). The nuclear Feulgen-DNA content of the vertebrates (especially reptiles), as measured by fluorescence cytophotometry, with notes on the cell and chromosome size. Acta Zoologica et Pathologica Antverpiensia 76: 119-167.
Cyprinoidei	Leuciscidae	*Scardinius*	0.94	BCA	RBC	NS	919.32	Mauro, M.L. and G. Micheli (1979). DNA reassociation kinetics in diploid and phylogenetically tetraploid cyprinidae. Journal of Experimental Zoology 208: 407-416.
Cyprinoidei	Leuciscidae	*Scardinius*	0.98	FD	RBC	HS	958.44	Hafez, R., R. Labat, and R. Quillier (1978). Teneurs nuclÃ©aires en A.D.N. et relations Ã©volutives dans la famille des cyprinides (Teleostei). Bulletin de la SocietÃ© d'Histoire Naturelle de Toulouse 114: 71-84.
Cyprinoidei	Leuciscidae	*Scardinius*	1.41	FCM	RBC	GD	1378.98	Moreira da Costa, L., M.J. Collares-Pereira, S. Lusk, and P. Rab (1999). New data on genome size variation of Centro-European Cyprinidae (Pisces, Osteichthyes). poster, Barcelona.
Cyprinoidei	Leuciscidae	*Scardinius*	1.71	FD	RBC	CP	1672.38	Suzuki, A., Y. Taki, M. Mochizuki, and J. Hirata (1995). Chromosomal speciation in Eurasian and Japanese Cyprinidae (Pisces, Cypriniformes). Cytobios 83: 171-186.
Cyprinoidei	Leuciscidae	*Semotilus*	1.23	FIA	RBC	BS, GD, OM, RP	1202.94	Hardie, D.C. and P.D.N. Hebert (2003). The nucleotypic effects of cellular DNA content in cartilaginous and ray-finned fishes. Genome 46: 683-706.; Hardie, D.C. and P.D.N. Hebert (2004). Genome-size evolution in fishes. Canadian Journal of Fisheries and Aquatic Sciences 61: 1636-1646. Click here to download data appendix.
Cyprinoidei	Leuciscidae	*Semotilus*	1.26	FD	RBC	GD	1232.28	Gold, J.R. and C.T. Amemiya (1987). Genome size variation in North American minnows (Cyprinidae). II. Variation among 20 species. Genome 29: 481-489.
Cyprinoidei	Leuciscidae	*Squalius*	1.18	FCM	RBC	GD	1154.04	Collares-Pereira, M.J. and L. Moreira da Costa (1999). Intraspecific and interspecific genome size variation in Iberian Cyprinidae and the problem of diploidy and polyploidy, with review of genome sizes within the family. Folia Zoologica 48: 61-76.
Cyprinoidei	Leuciscidae	*Squalius*	1.21	FCM	RBC	GD	1183.38	Collares-Pereira, M.J. and L. Moreira da Costa (1999). Intraspecific and interspecific genome size variation in Iberian Cyprinidae and the problem of diploidy and polyploidy, with review of genome sizes within the family. Folia Zoologica 48: 61-76.
Cyprinoidei	Leuciscidae	*Squalius*	1.21	FCM	RBC	GD	1183.38	Collares-Pereira, M.J. and L. Moreira da Costa (1999). Intraspecific and interspecific genome size variation in Iberian Cyprinidae and the problem of diploidy and polyploidy, with review of genome sizes within the family. Folia Zoologica 48: 61-76.
Cyprinoidei	Leuciscidae	*Squalius*	1.22	FCM	RBC	GD	1193.16	Collares-Pereira, M.J. and L. Moreira da Costa (1999). Intraspecific and interspecific genome size variation in Iberian Cyprinidae and the problem of diploidy and polyploidy, with review of genome sizes within the family. Folia Zoologica 48: 61-76.
Cyprinoidei	Leuciscidae	*Squalius*	1.23	FCM	RBC	GD	1202.94	Prospero, M.I. and M.J. Collares-Pereira (2000). Nuclear DNA content variation in the diploid-polyploid Leuciscus alburnoides complex (Teleostei, Cyprinidae) assessed by flow cytometry. Folia Zoologica 49: 53-58.
Cyprinoidei	Leuciscidae	*Squalius*	1.27	BCA	RBC	NS	1242.06	Mauro, M.L. and G. Micheli (1979). DNA reassociation kinetics in diploid and phylogenetically tetraploid cyprinidae. Journal of Experimental Zoology 208: 407-416.
Cyprinoidei	Leuciscidae	*Squalius*	1.33	FD	RBC	HS	1300.74	Hafez, R., R. Labat, and R. Quillier (1978). Teneurs nuclÃ©aires en A.D.N. et relations Ã©volutives dans la famille des cyprinides (Teleostei). Bulletin de la SocietÃ© d'Histoire Naturelle de Toulouse 114: 71-84.
Cyprinoidei	Leuciscidae	*Squalius*	1.33	FD	RBC	HS	1300.74	Wolf, U., H. Ritter, N.B. Atkin, and S. Ohno (1969). Polyploidization in the fish family Cyprinidae, Order Cypriniformes. I. DNA-content and chromosome sets in various species of Cyprinidae. Humangenetik 7: 240-244.
Cyprinoidei	Tincidae	*Tinca*	0.81	BCA	RBC	NS	792.18	Mauro, M.L. and G. Micheli (1979). DNA reassociation kinetics in diploid and phylogenetically tetraploid cyprinidae. Journal of Experimental Zoology 208: 407-416.
Cyprinoidei	Tincidae	*Tinca*	0.85	FD	RBC	TT	831.3	Fontana, F. (1976). Nuclear DNA content and cytometry of erythrocytes of Huso huso L., Acipenser sturio L. and Acipenser naccarii Bonaparte. Caryologia 29: 127-137.
Cyprinoidei	Tincidae	*Tinca*	0.93	NS	RBC	CP	909.54	Gerzeli, G., C. Casati, and A. Meneghetti Gennaro (1956). I volumi nucleari e cellulari in relazione al tenore di acido desossiribonucleico in eritrociti di alcune specie di Vertebrati. Rivista di Istochimica Normale e Patologica 2: 149-155.
Cyprinoidei	Tincidae	*Tinca*	0.95	NS	NS	GD	929.1	Vialli, M. (1957a). Volume et contenu en ADN par noyau. Experimental Cell Research Suppl. 4: 284-293.
Cyprinoidei	Tincidae	*Tinca*	0.97	BCA	RBC	GD	948.66	Vendrely, R. and C. Vendrely (1953). Argenine an deoxyribonucleic acid content of erythrocyte nuclei and sperms of some species of fishes. Nature 172: 30-31.; Vendrely, R. and C. Vendrely (1952a). Sur la teneur comparÃ©e en arginine et en acide dÃ©soxyribonuclÃ©ique des noyaux d'Ã©rythrocytes de quelques espÃ¨ces de poissons. Comptes Rendus de l'AcadÃ©mie des Sciences 235: 395-397.
Cyprinoidei	Tincidae	*Tinca*	0.98	BCA	S	BT	958.44	Vendrely, R. and C. Vendrely (1952b). Sur la teneur individuelle en arginine des spermatozoÃ¯des comparÃ©e Ã la teneur individuelle en arginine des noyaux d'Ã©rythrocytes chez quelques espÃ¨ces de poissons. Comptes Rendus de l'AcadÃ©mie des Sciences 235:
Cyprinoidei	Tincidae	*Tinca*	1.01	FIA	RBC	GD	987.78	Bytyutskyy, D. and M. Flajshans (2014). Use of diploid and triploid tench (Tinca tinca) blood as standards for genome size measurements. Journal of Applied Ichthyology 30 (Suppl. 1): 12-14.
Cyprinoidei	Tincidae	*Tinca*	1.02	FIA	RBC	GD	997.56	Bytyutskyy, D., J. Srp, and M. Flajshans (2012). Use of Feulgen image analysis densitometry to study the effect of genome size on nuclear size in polyploid sturgeons. Journal of Applied Ichthyology 28: 704-708.
Cyprinoidei	Tincidae	*Tinca*	1.04	FD	RBC	HS	1017.12	Hafez, R., R. Labat, and R. Quillier (1978). Teneurs nuclÃ©aires en A.D.N. et relations Ã©volutives dans la famille des cyprinides (Teleostei). Bulletin de la SocietÃ© d'Histoire Naturelle de Toulouse 114: 71-84.

Cyprinoidei	Tincidae	*Tinca*	1.05	BCA	RBC	HS	1026.9	Vendrely, R. and C. Vendrely (1950). Sur la teneur absolue en acide dÃ©soxyribonuclÃ©ique du noyau cellulaire chez quelques espÃ¨ces d'oiseaux et de poissons. Comptes Rendus de l'AcadÃ©mie des Sciences 230: 788-790.
Cyprinoidei	Tincidae	*Tinca*	1.05	FD	RBC	HS	1026.9	Wolf, U., H. Ritter, N.B. Atkin, and S. Ohno (1969). Polyploidization in the fish family Cyprinidae, Order Cypriniformes. I. DNA-content and chromosome sets in various species of Cyprinidae. Humangenetik 7: 240-244.
Cyprinoidei	Tincidae	*Tinca*	1.13	FCM	RBC	GD	1105.14	Collares-Pereira, M.J. and L. Moreira da Costa (1999). Intraspecific and interspecific genome size variation in Iberian Cyprinidae and the problem of diploidy and polyploidy, with review of genome sizes within the family. Folia Zoologica 48: 61-76.
Cyprinoidei	Xenocyprididae	*Hemiculter*	1.22	FD	RBC	GD	1193.16	Cui, J., X. Ren, and Q. Yu (1991). Nuclear DNA content variation in fishes. Cytologia 56: 425-429.
Cyprinoidei	Xenocyprididae	*Opsariichthys*	1.15	FCM	RBC	MM	1124.7	Ojima, Y. and K. Yamamoto (1990). Cellular DNA contents of fishes determined by flow cytometry. La Kromosomo II 57: 1871-1888.
Cyprinoidei	Xenocyprididae	*Opsariichthys*	1.67	FD	LV, BR	CA	1633.26	Ojima, Y., M. Hayashi, and K. Ueno (1972). Cytogenetic studies in lower vertebrates. X. Karyotype and DNA studies in 15 species of Japanese Cyprinidae. Japanese Journal of Genetics 47: 431-440.
Gyrinocheiloidei	Gyrinocheilidae	*Gyrinocheilus*	0.51	FD	RBC	CP	498.78	Arai, R., A. Suzuki, and Y. Akai (1988). The karyotype and DNA value of a cypriniform algae eater, Gyrinocheilus aymonieri. Japanese Journal of Ichthyology 34: 515-517.
Gyrinocheiloidei	Gyrinocheilidae	*Gyrinocheilus*	0.61	FCM	RBC	MM	596.58	Ojima, Y. and K. Yamamoto (1990). Cellular DNA contents of fishes determined by flow cytometry. La Kromosomo II 57: 1871-1888.
Gyrinocheiloidei	Gyrinocheilidae	*Gyrinocheilus*	0.62	FIA	RBC	BS, GD, OM, RP	606.36	Hardie, D.C. and P.D.N. Hebert (2003). The nucleotypic effects of cellular DNA content in cartilaginous and ray-finned fishes. Genome 46: 683-706.; Hardie, D.C. and P.D.N. Hebert (2004). Genome-size evolution in fishes. Canadian Journal of Fisheries and Aquatic Sciences 61: 1636-1646. Click here to download data appendix.
Gyrinocheiloidei	Gyrinocheilidae	*Gyrinocheilus*	0.65	BFA	RBC	SP	635.7	Hinegardner, R. (1968). Evolution of cellular DNA content in teleost fishes. American Naturalist 102: 517-523.; Hinegardner, R. and D.E. Rosen (1972). Cellular DNA content and the evolution of teleostean fishes. American Naturalist 106: 621-644.

Table S12. Comparative dataset of 309 cypriniform species used in the phylogenetic comparative analyses of miniaturization.

Voucher_reads	BioProject	BioSample	Suborder	Family	Genus	Species	Source	Genome_assembly_Mb_NCBI	Ploidy	Miniature	SL.max.cm	mean_k31_genomesize_MB	genomesize_MB
LR15247	PRJNA1483025	SAMN61141513	Catostomoidei	Catostomidae	*Cycleptus*	sp	This study	NA	polyploid	non-miniature	66.5	2509.817213	2509.817213
LR15245	PRJNA1483025	SAMN61141514	Catostomoidei	Catostomidae	*Erimyzon*	*sucetta*	This study	NA	polyploid	non-miniature	31.4	1788.172173	1788.172173
LR15248	PRJNA1483025	SAMN61141516	Catostomoidei	Catostomidae	*Moxostoma*	*congestum*	This study	NA	polyploid	non-miniature	41.1	2017.530905	2017.530905
GCF_019703515.2	PRJNA739167	SAMN19774990	Catostomoidei	Catostomidae	*Myxocyprinus*	*asiaticus*	NCBI	NA	polyploid	non-miniature	50	2309.856512	2309.856512
LR15354	PRJNA1483025	SAMN61141518	Cobitoidei	Balitoridae	*Balitora*	sp	This study	NA	diploid	non-miniature	7.7	492.1509565	492.1509565
LR15094	PRJNA1483025	SAMN61141519	Cobitoidei	Balitoridae	*Balitoropsis*	*zollingeri*	This study	NA	diploid	non-miniature	6.77	416.506414	416.506414
KUFOS-AN-2019.1.1	PRJNA1393636	SAMN54310100	Cobitoidei	Balitoridae	*Bhavania*	*australis*	This study	NA	diploid	non-miniature	9	432.345153	432.345153
LR15176	PRJNA1483025	SAMN61141520	Cobitoidei	Balitoridae	*Hemimyzon*	*formosanum*	This study	NA	diploid	non-miniature	10	509.5217015	509.5217015
LR15139	PRJNA1483025	SAMN61141521	Cobitoidei	Balitoridae	*Homaloptera*	*orthogoniata*	This study	NA	diploid	non-miniature	13	450.377603	450.377603
LR15140	PRJNA1483025	SAMN61141522	Cobitoidei	Balitoridae	*Homalopteroides*	*stephensoni*	This study	NA	diploid	non-miniature	6.5	488.628322	488.628322
LR15093	PRJNA1483025	SAMN61141523	Cobitoidei	Balitoridae	*Homalopteroides*	*tweediei*	This study	NA	diploid	proportioned-dwarf	4	413.243012	413.243012
LR15091	PRJNA1483025	SAMN61141524	Cobitoidei	Balitoridae	*Homalopterula*	*gymnogaster*	This study	NA	diploid	non-miniature	6.6	347.959084	347.959084
LR15172	PRJNA1483025	SAMN61141525	Cobitoidei	Balitoridae	*Lepturichthys*	*fimbriatus*	This study	NA	diploid	non-miniature	16.1	530.93438	530.93438
LR15487	PRJNA1483025	SAMN61141526	Cobitoidei	Balitoridae	*Neohomaloptera*	*johorensis*	This study	NA	diploid	proportioned-dwarf	2.05	357.9961275	357.9961275
LR15088	PRJNA1483025	SAMN61141527	Cobitoidei	Balitoridae	*Sinogastromyzon*	*puliensis*	This study	NA	diploid	non-miniature	7.45	537.5911635	537.5911635
LR15181	PRJNA1483025	SAMN61141528	Cobitoidei	Balitoridae	*Travancoria*	*elongata*	This study	NA	diploid	non-miniature	11.4	419.2010845	419.2010845
LR15360	PRJNA1483025	SAMN61141529	Cobitoidei	Barbuccidae	*Barbucca*	*elongatus*	This study	NA	diploid	proportioned-dwarf	2.7	379.578087	379.578087
LR15330	PRJNA1483025	SAMN61141530	Cobitoidei	Botiidae	*Ambastaia*	*sidthimunki*	This study	NA	polyploid	non-miniature	5.5	728.1004125	728.1004125
GCA_036877575.1	PRJNA1067307	SAMN39625707	Cobitoidei	Botiidae	*Botia*	*almorhae*	NCBI	894.1	polyploid	non-miniature	15.5	NA	894.1
LR15149	PRJNA1483025	SAMN61141531	Cobitoidei	Botiidae	*Botia*	*kubotai*	This study	NA	polyploid	non-miniature	8.49	820.033105	820.033105
LR15148	PRJNA1483025	SAMN61141532	Cobitoidei	Botiidae	*Chromobotia*	*macracanthus*	This study	NA	polyploid	non-miniature	25	821.307172	821.307172
LR15074	PRJNA1483025	SAMN61141533	Cobitoidei	Botiidae	*Leptobotia*	*elongata*	This study	NA	diploid	non-miniature	50	626.037003	626.037003
112LR15712	PRJNA1483025	SAMN61141534	Cobitoidei	Botiidae	*Parabotia*	*bimaculatus*	This study	NA	diploid	non-miniature	15.8	615.909513	615.909513
LR15151	PRJNA1483025	SAMN61141535	Cobitoidei	Botiidae	*Sinibotia*	*robusta*	This study	NA	polyploid	non-miniature	6.63	755.627618	755.627618
LR15446	PRJNA1483025	SAMN61141536	Cobitoidei	Botiidae	*Syncrossus*	*berdmorei*	This study	NA	polyploid	non-miniature	11.1	650.9152395	650.9152395
GCA_036877545.1	PRJNA1067307	SAMN39625709	Cobitoidei	Botiidae	*Yasuhikotakia*	*modesta*	NCBI	636.8	polyploid	non-miniature	25	NA	636.8
115LR15715	PRJNA1483025	SAMN61141537	Cobitoidei	Cobitidae	*Acanthopsoides*	*robertsi*	This study	NA	diploid	non-miniature	5.5	595.835145	595.835145
LR15193	PRJNA1483025	SAMN61141539	Cobitoidei	Cobitidae	*Bibarba*	*bibarba*	This study	NA	diploid	non-miniature	5.5	527.2919795	527.2919795
LR15575	PRJNA1483025	SAMN61141540	Cobitoidei	Cobitidae	*Canthophrys*	*gongota*	This study	NA	diploid	non-miniature	6.51	540.609599	540.609599
LR15465	PRJNA1483025	SAMN61141541	Cobitoidei	Cobitidae	*Cobitis*	*sinensis*	This study	NA	diploid	non-miniature	10.9	1612.45102	1612.45102
LR15170	PRJNA1483025	SAMN61141542	Cobitoidei	Cobitidae	*Kottelatlimia*	*katik*	This study	NA	diploid	proportioned-dwarf	1.35	1043.902574	1043.902574
114LR15714	PRJNA1483025	SAMN61141543	Cobitoidei	Cobitidae	*Kottelatlimia*	*pristes*	This study	NA	diploid	non-miniature	4	892.8929045	892.8929045
LR15467	PRJNA1483025	SAMN61141544	Cobitoidei	Cobitidae	*Lepidocephalichthys*	sp	This study	NA	diploid	non-miniature	6.28	656.0133925	656.0133925
GCF_027580225.1	PRJNA812369	SAMN26377316	Cobitoidei	Cobitidae	*Misgurnus*	*anguillicaudatus*	NCBI	1104	diploid	non-miniature	28	NA	1104
GCA_025389145.1	PRJNA751427	SAMN20518172	Cobitoidei	Cobitidae	*Misgurnus*	*mizolepis*	NCBI	1112	diploid	non-miniature	16.7	NA	1112
BNHS-FWF-981	PRJNA1393636	SAMN54310101	Cobitoidei	Cobitidae	*Pangio*	*bhujia*	This study	NA	diploid	proportioned-dwarf	2.6	676.7185705	676.7185705
LR15141	PRJNA1483025	SAMN61141546	Cobitoidei	Cobitidae	*Pangio*	*semicincta*	This study	NA	diploid	non-miniature	6.62	813.269303	813.269303
LR15609	PRJNA1483025	SAMN61141547	Cobitoidei	Cobitidae	*Sabanejewia*	*balcanica*	This study	NA	diploid	non-miniature	9	1039.00824	1039.00824
LR15146	PRJNA1483025	SAMN61141548	Cobitoidei	Cobitidae	*Theriodes*	*sandakanensis*	This study	NA	diploid	non-miniature	3.5	487.8762655	487.8762655
141LR15748	PRJNA1483025	SAMN61141549	Cobitoidei	Ellopostomatidae	*Ellopostoma*	*mystax*	This study	NA	diploid	non-miniature	5.53	452.711028	452.711028
LR15180	PRJNA1483025	SAMN61141550	Cobitoidei	Gastromyzontidae	*Annamia*	*normani*	This study	NA	diploid	non-miniature	7.91	417.169605	417.169605
GCA_019155185.1	PRJNA650219	SAMN15699278	Cobitoidei	Gastromyzontidae	*Beaufortia*	*kweichowensis*	NCBI	NA	diploid	non-miniature	5.4	462.685559	462.685559
LR15491	PRJNA1483025	SAMN61141552	Cobitoidei	Gastromyzontidae	*Erromyzon*	*compactus*	This study	NA	diploid	non-miniature	5.2	446.541696	446.541696

LR15090	PRJNA1483025	SAMN61141553	Cobitoidei	Gastromyzontidae	*Glaniopsis*	*muliradiatus*	This study	NA	diploid	non-miniature	6	472.8609795	472.8609795
LR15133	PRJNA1483025	SAMN61141554	Cobitoidei	Gastromyzontidae	*Katibasia*	*insidiosa*	This study	NA	diploid	non-miniature	3.4	427.0149425	427.0149425
LR15175	PRJNA1483025	SAMN61141555	Cobitoidei	Gastromyzontidae	*Liniparhomaloptera*	*disparis*	This study	NA	diploid	non-miniature	6.3	431.0346715	431.0346715
LR15476	PRJNA1483025	SAMN61141556	Cobitoidei	Gastromyzontidae	*Plesiomyzon*	*baotingensis*	This study	NA	diploid	non-miniature	3.94	428.9316375	428.9316375
LR15425	PRJNA1483025	SAMN61141557	Cobitoidei	Gastromyzontidae	*Protomyzon*	*borneensis*	This study	NA	diploid	non-miniature	5.4	416.6464905	416.6464905
LR15067	PRJNA1483025	SAMN61141558	Cobitoidei	Gastromyzontidae	*Pseudogastromyzon*	sp	This study	NA	diploid	non-miniature	8.2	438.344733	438.344733
LR15561	PRJNA1483025	SAMN61141559	Cobitoidei	Gastromyzontidae	*Sewellia*	*elongata*	This study	NA	diploid	non-miniature	7.3	411.1789435	411.1789435
113LR15713	PRJNA1483025	SAMN61141561	Cobitoidei	Gastromyzontidae	*Yaoshania*	*pachychilus*	This study	NA	diploid	non-miniature	5.8	435.496029	435.496029
LR15343	PRJNA1483025	SAMN61141562	Cobitoidei	Nemacheilidae	*Aborichthys*	*elongatus*	This study	NA	diploid	non-miniature	7.4	518.9789115	518.9789115
GCA_947034865.1	PRJEB56420	SAMEA11296540	Cobitoidei	Nemacheilidae	*Barbatula*	*barbatula*	NCBI	617.6	diploid	non-miniature	12	NA	617.6
LR15190	PRJNA1483025	SAMN61141563	Cobitoidei	Nemacheilidae	*Barbatula*	sp	This study	NA	diploid	non-miniature	7	647.9074045	647.9074045
LR15073	PRJNA1483025	SAMN61141564	Cobitoidei	Nemacheilidae	*Homatula*	*variegata*	This study	NA	diploid	non-miniature	12.4	697.3544255	697.3544255
KUFOS-AN-2019.2.1	PRJNA1393636	SAMN54310102	Cobitoidei	Nemacheilidae	*Indoreonectes*	*keralensis*	This study	NA	diploid	non-miniature	4.65	393.362597	393.362597
LR15462	PRJNA1483025	SAMN61141565	Cobitoidei	Nemacheilidae	*Lefua*	sp	This study	NA	diploid	non-miniature	5.3	551.812393	551.812393
KUFOS-AN-2021.1.1	PRJNA1393636	SAMN54310103	Cobitoidei	Nemacheilidae	*Mesonoemacheilus*	*triangularis*	This study	NA	diploid	non-miniature	5.8	493.90045	493.90045
LR15337	PRJNA1483025	SAMN61141566	Cobitoidei	Nemacheilidae	*Micronemacheilus*	sp	This study	NA	diploid	non-miniature	5.8	551.353682	551.353682
LR15459	PRJNA1483025	SAMN61141567	Cobitoidei	Nemacheilidae	*Nemacheilus*	*selangoricus*	This study	NA	diploid	non-miniature	4.8	475.761951	475.761951
LR15611	PRJNA1483025	SAMN61141568	Cobitoidei	Nemacheilidae	*Neonoemacheilus*	sp	This study	NA	diploid	non-miniature	7.32	611.2010315	611.2010315
LR15490	PRJNA1483025	SAMN61141569	Cobitoidei	Nemacheilidae	*Oreonectes*	*platycephalus*	This study	NA	diploid	non-miniature	4.38	606.3790165	606.3790165
LR15504	PRJNA1483025	SAMN61141570	Cobitoidei	Nemacheilidae	*Paracanthocobitis*	*botia*	This study	NA	diploid	non-miniature	5.77	550.526874	550.526874
LR15189	PRJNA1483025	SAMN61141571	Cobitoidei	Nemacheilidae	*Petruichthys*	*brevis*	This study	NA	diploid	non-miniature	3.21	573.6448505	573.6448505
LR15152	PRJNA1483025	SAMN61141572	Cobitoidei	Nemacheilidae	*Schistura*	*fasciolata*	This study	NA	diploid	non-miniature	6	697.99144	697.99144
LR15454	PRJNA1483025	SAMN61141573	Cobitoidei	Nemacheilidae	*Schistura*	sp1	This study	NA	diploid	non-miniature	5.4	521.701272	521.701272
LR15496	PRJNA1483025	SAMN61141574	Cobitoidei	Nemacheilidae	*Schistura*	sp2	This study	NA	diploid	non-miniature	4.2	689.9526065	689.9526065
LR15455	PRJNA1483025	SAMN61141575	Cobitoidei	Nemacheilidae	*Sundoreonectes*	*sabanus*	This study	NA	diploid	non-miniature	5.41	655.1661915	655.1661915
LR15072	PRJNA1483025	SAMN61141576	Cobitoidei	Nemacheilidae	*Traccatichthys*	*pulcher*	This study	NA	diploid	non-miniature	5.39	580.90548	580.90548
GCF_015846415.1	PRJNA624716	SAMN14582390	Cobitoidei	Nemacheilidae	*Triplophysa*	*dalaica*	NCBI	607.9	diploid	non-miniature	8.2	NA	607.9
GCF_024868665.1	PRJNA673076	SAMN16587819	Cobitoidei	Nemacheilidae	*Triplophysa*	*rosa*	NCBI	684.8	diploid	non-miniature	5.6	NA	684.8
GCA_006030095.1	PRJNA543715	SAMN11775600	Cobitoidei	Nemacheilidae	*Triplophysa*	*siluroides*	NCBI	583.4	diploid	non-miniature	50	NA	583.4
GCA_008369825.1	PRJNA526292	SAMN11094431	Cobitoidei	Nemacheilidae	*Triplophysa*	*tibetana*	NCBI	652.9	diploid	non-miniature	13.9	NA	652.9
GCA_033220385.1	PRJNA995909	SAMN36510128	Cobitoidei	Nemacheilidae	*Triplophysa*	*yarkandensis*	NCBI	520.8	diploid	non-miniature	17.7	NA	520.8
LR15357	PRJNA1483025	SAMN61141577	Cobitoidei	Serpenticobitidae	*Serpenticobitis*	*zonata*	This study	NA	diploid	non-miniature	4.3	440.308335	440.308335
LR15334	PRJNA1483025	SAMN61141578	Cobitoidei	Vaillantellidae	*Vaillantella*	*maassii*	This study	NA	diploid	non-miniature	12.5	427.556523	427.556523
LR15562	PRJNA1483025	SAMN61141579	Cyprinoidei	Acheilognathidae	*Acheilognathus*	*deignani*	This study	NA	diploid	non-miniature	5.2	752.796849	752.796849
LR15063	PRJNA1483025	SAMN61141580	Cyprinoidei	Acheilognathidae	*Acheilognathus*	sp	This study	NA	diploid	non-miniature	5.2	830.2436615	830.2436615
LR15636	PRJNA1483025	SAMN61141581	Cyprinoidei	Acheilognathidae	*Paratanakia*	*himantegus*	This study	NA	diploid	non-miniature	6.3	703.096636	703.096636
LR15661	PRJNA1483025	SAMN61141582	Cyprinoidei	Acheilognathidae	*Rhodeus*	*amarus*	This study	NA	diploid	non-miniature	9.5	840.9927755	840.9927755
GCA_037043235.1	PRJNA1033701	SAMN38042216	Cyprinoidei	Acheilognathidae	*Rhodeus*	*uyekii*	NCBI	894.5	diploid	non-miniature	5	NA	894.5
LR15266	PRJNA1483025	SAMN61141583	Cyprinoidei	Cyprinidae	*Acapoeta*	*tanganicae*	This study	NA	polyploid	non-miniature	29	1296.278517	1296.278517
GCA_039880705.1	PRJNA1049304	SAMN39587862	Cyprinoidei	Cyprinidae	*Acrossocheilus*	*fasciatus*	NCBI	NA	diploid	non-miniature	19.2	937.795839	937.795839
LR15615	PRJNA1483025	SAMN61141585	Cyprinoidei	Cyprinidae	*Ageneiogarra*	sp	This study	NA	diploid	non-miniature	34.5	1156.287975	1156.287975
GCA_023376895.1	PRJNA832538	SAMN27865501	Cyprinoidei	Cyprinidae	*Aspiorhynchus*	*laticeps*	NCBI	1583	polyploid	non-miniature	100	NA	1583
LR15639	PRJNA1483025	SAMN61141586	Cyprinoidei	Cyprinidae	*Balantiocheilos*	*melanopterus*	This study	NA	diploid	non-miniature	35	738.167276	738.167276
LR15079	PRJNA1483025	SAMN61141587	Cyprinoidei	Cyprinidae	*Barbichthys*	*laevis*	This study	NA	diploid	non-miniature	30	887.40424	887.40424
LR15102	PRJNA1483025	SAMN61141588	Cyprinoidei	Cyprinidae	*Barbodes*	*lateristriga*	This study	NA	diploid	non-miniature	7	740.2849255	740.2849255

120LR15721	PRJNA1483025	SAMN61141589	Cyprinoidei	Cyprinidae	*Barbodes*	*sellifer*	This study	NA	diploid	non-miniature	9.8	778.466779	778.466779
LR15256	PRJNA1483025	SAMN61141590	Cyprinoidei	Cyprinidae	*Barboides*	*gracilis*	This study	NA	diploid	proportioned-dwarf	1.8	636.29766	636.29766
EA0124	PRJNA1483025	SAMN61141591	Cyprinoidei	Cyprinidae	*Barbonymus*	*altus*	This study	NA	diploid	non-miniature	15	809.7455375	809.7455375
GCA_936440315.1	PRJEB52077	SAMEA13335783	Cyprinoidei	Cyprinidae	*Barbus*	*barbus*	NCBI	1584	polyploid	non-miniature	90	NA	1584
GCF_003368295.1	PRJNA481500	SAMN09670328	Cyprinoidei	Cyprinidae	*Carassius*	*auratus*	NCBI	1820	polyploid	non-miniature	26	NA	1820
GCF_963082965.1	PRJEB64837	SAMEA11296534	Cyprinoidei	Cyprinidae	*Carassius*	*carassius*	NCBI	1684	polyploid	non-miniature	55	NA	1684
GCA_023724075.1	PRJNA779620	SAMN23046545	Cyprinoidei	Cyprinidae	*Carassius*	*gibelio*	NCBI	1949	polyploid	non-miniature	35	NA	1949
LR15111	PRJNA1483025	SAMN61141592	Cyprinoidei	Cyprinidae	*Ceratogarra*	*cambodgiensis*	This study	NA	diploid	non-miniature	10	847.3830295	847.3830295
EA0401	PRJNA1483025	SAMN61141594	Cyprinoidei	Cyprinidae	*Chagunius*	*nicholsi*	This study	NA	diploid	non-miniature	25	720.7493135	720.7493135
LR15270	PRJNA1483025	SAMN61141595	Cyprinoidei	Cyprinidae	*Clypeobarbus*	sp	This study	NA	diploid	non-miniature	3.3	829.8340545	829.8340545
LR10955	PRJNA1483025	SAMN61141596	Cyprinoidei	Cyprinidae	*Crossocheilus*	*reticulatus*	This study	NA	diploid	non-miniature	17	1296.106392	1296.106392
LR10341	PRJNA1483025	SAMN61141597	Cyprinoidei	Cyprinidae	*Cyclocheilichthys*	*apogon*	This study	NA	diploid	non-miniature	16	833.4889075	833.4889075
GCF_018340385.1	PRJNA682709	SAMN17005855	Cyprinoidei	Cyprinidae	*Cyprinus*	*carpio*	NCBI	1680	polyploid	non-miniature	40	NA	1680
KUFOS-AN-2021.4.1	PRJNA1393636	SAMN54310104	Cyprinoidei	Cyprinidae	*Dawkinsia*	*filamentosa*	This study	NA	diploid	non-miniature	12	638.710795	638.710795
140LR15737	PRJNA1483025	SAMN61141598	Cyprinoidei	Cyprinidae	*Desmopuntius*	*pentazona*	This study	NA	diploid	non-miniature	3	765.758192	765.758192
122LR15725	PRJNA1483025	SAMN61141599	Cyprinoidei	Cyprinidae	*Discherodontus*	*ashmeadi*	This study	NA	diploid	non-miniature	13.6	723.054833	723.054833
119LR15720	PRJNA1483025	SAMN61141600	Cyprinoidei	Cyprinidae	*Eirmotus*	*isthmus*	This study	NA	diploid	non-miniature	3.6	648.622376	648.622376
LR09582	PRJNA1483025	SAMN61141601	Cyprinoidei	Cyprinidae	*Eirmotus*	sp2	This study	NA	diploid	non-miniature	3.6	658.8349075	658.8349075
GCA_034779775.1	PRJNA915624	SAMN32526004	Cyprinoidei	Cyprinidae	*Enteromius*	*afrohamiltoni*	NCBI	663.5	diploid	non-miniature	17.5	NA	663.5
GCA_032359395.1	PRJNA917146	SAMN32526002	Cyprinoidei	Cyprinidae	*Enteromius*	*anoplus*	NCBI	737.7	diploid	non-miniature	10	NA	737.7
GCA_032357425.1	PRJNA917131	SAMN32525989	Cyprinoidei	Cyprinidae	*Enteromius*	*argenteus*	NCBI	681.6	diploid	non-miniature	17.5	NA	681.6
GCA_032359235.1	PRJNA915750	SAMN32525988	Cyprinoidei	Cyprinidae	*Enteromius*	*mattozi*	NCBI	727.4	diploid	non-miniature	40	NA	727.4
GCA_032357445.1	PRJNA917141	SAMN32525998	Cyprinoidei	Cyprinidae	*Enteromius*	*radiatus*	NCBI	616.7	diploid	non-miniature	12	NA	616.7
LR15606	PRJNA1483025	SAMN61141603	Cyprinoidei	Cyprinidae	*Enteromius*	sp1	This study	NA	diploid	non-miniature	15	859.7960585	859.7960585
GCA_032359435.1	PRJNA917137	SAMN32525994	Cyprinoidei	Cyprinidae	*Enteromius*	*unitaeniatus*	NCBI	NA	diploid	non-miniature	17.5	878.386854	878.386854
LR01655	PRJNA1483025	SAMN61141604	Cyprinoidei	Cyprinidae	*Epalzeorhynchos*	*kalopterum*	This study	NA	diploid	non-miniature	12	1362.220601	1362.220601
LR15563	PRJNA1483025	SAMN61141605	Cyprinoidei	Cyprinidae	*Folifer*	*brevifilis*	This study	NA	diploid	non-miniature	19.2	900.587643	900.587643
KUFOS-RR-2022.P.4	PRJNA1393636	SAMN54310106	Cyprinoidei	Cyprinidae	*Garra*	*periyarensis*	This study	NA	diploid	non-miniature	16	967.001756	967.001756
GCA_027564155.1	PRJNA771798	SAMN22344571	Cyprinoidei	Cyprinidae	*Gymnocypris*	*eckloni-scoliostomus*	NCBI	948.1	polyploid	non-miniature	39.5	NA	948.1
LR15635	PRJNA1483025	SAMN61141606	Cyprinoidei	Cyprinidae	*Haludaria*	*fasciata*	This study	NA	diploid	non-miniature	6	590.2574145	590.2574145
LR15078	PRJNA1483025	SAMN61141607	Cyprinoidei	Cyprinidae	*Hampala*	*ampalong*	This study	NA	diploid	non-miniature	15	667.0398945	667.0398945
KUFOS-RR-2022.P.3	PRJNA1393636	SAMN54310107	Cyprinoidei	Cyprinidae	*Hypselobarbus*	*periyarensis*	This study	NA	polyploid	non-miniature	40	752.678211	752.678211
GCA_012976165.1	PRJNA557138	SAMN12390082	Cyprinoidei	Cyprinidae	*Labeo*	*catla*	NCBI	1019	diploid	non-miniature	120	NA	1019
GCF_022985175.1	PRJNA650519	SAMN15716776	Cyprinoidei	Cyprinidae	*Labeo*	*rohita*	NCBI	1126	diploid	non-miniature	150	NA	1126
LR15278	PRJNA1483025	SAMN61141608	Cyprinoidei	Cyprinidae	*Labeo*	*cylindricus*	This study	NA	diploid	non-miniature	25	1028.751666	1028.751666
GCA_034780575.1	PRJNA996131	SAMN32526010	Cyprinoidei	Cyprinidae	*Labeobarbus*	*marequensis*	NCBI	NA	polyploid	non-miniature	35	1263.354553	1263.354553
LR12884	PRJNA1483025	SAMN61141610	Cyprinoidei	Cyprinidae	*Labiobarbus*	*ocellatus*	This study	NA	diploid	non-miniature	15.5	1143.454791	1143.454791
KUFOS-RR-2022.P.2	PRJNA1393636	SAMN54310108	Cyprinoidei	Cyprinidae	*Lepidopygopsis*	*typus*	This study	NA	polyploid	non-miniature	20	1406.545258	1406.545258
LR15104	PRJNA1483025	SAMN61141611	Cyprinoidei	Cyprinidae	*Lobocheilos*	*aureolineatus*	This study	NA	diploid	non-miniature	5.99	1011.286788	1011.286788
LR15565	PRJNA1483025	SAMN61141612	Cyprinoidei	Cyprinidae	*Mystacoleucus*	*argenteus*	This study	NA	diploid	non-miniature	15	683.1196445	683.1196445
LR15612	PRJNA1483025	SAMN61141613	Cyprinoidei	Cyprinidae	*Neolissochilus*	sp	This study	NA	polyploid	non-miniature	60	857.157747	857.157747
143LR15682	PRJNA1483025	SAMN61141614	Cyprinoidei	Cyprinidae	*Oliotius*	*oligolepis*	This study	NA	diploid	non-miniature	5	605.885024	605.885024
GCF_012432095.1	PRJNA608076	SAMN14163390	Cyprinoidei	Cyprinidae	*Onychostoma*	*macrolepis*	NCBI	886.6	diploid	non-miniature	26.5	NA	886.6
EA1291	PRJNA1483025	SAMN61141615	Cyprinoidei	Cyprinidae	*Oreichthys*	sp	This study	NA	diploid	non-miniature	3	679.6391495	679.6391495
LR09367	PRJNA1483025	SAMN61141616	Cyprinoidei	Cyprinidae	*Osteobrama*	*cunma*	This study	NA	diploid	non-miniature	12	718.0802345	718.0802345

LR15096	PRJNA1483025	SAMN61141617	Cyprinoidei	Cyprinidae	*Osteochilus*	*vittatoides*	This study	NA	diploid	non-miniature	11.9	1105.531316	1105.531316
GCA_003573665.1	PRJNA485858	SAMN09762613	Cyprinoidei	Cyprinidae	*Oxygymnocypris*	*stewartii*	NCBI	1849	polyploid	non-miniature	54.6	NA	1849
LR01656	PRJNA1483025	SAMN61141618	Cyprinoidei	Cyprinidae	*Paracrossochilus*	*vittatus*	This study	NA	diploid	non-miniature	9.5	1281.936214	1281.936214
LR15618	PRJNA1483025	SAMN61141619	Cyprinoidei	Cyprinidae	*Paraspinibarbus*	*sp*	This study	NA	diploid	non-miniature	40	823.683848	823.683848
KUFOS-AN-2021.5.1	PRJNA1393636	SAMN54310110	Cyprinoidei	Cyprinidae	*Pethia*	*conchonius*	This study	NA	diploid	non-miniature	5.6	601.786245	601.786245
LR06584	PRJNA1483025	SAMN61141620	Cyprinoidei	Cyprinidae	*Pethia*	*sp1*	This study	NA	diploid	non-miniature	6	686.5376455	686.5376455
LR15646	PRJNA1483025	SAMN61141621	Cyprinoidei	Cyprinidae	*Pethia*	*gelius*	This study	NA	diploid	non-miniature	4.12	683.0203955	683.0203955
GCA_004124795.1	PRJNA511031	SAMN10662616	Cyprinoidei	Cyprinidae	*Poropuntius*	*huangchuchieni*	NCBI	760.2	diploid	non-miniature	25.6	NA	760.2
LR15083	PRJNA1483025	SAMN61141622	Cyprinoidei	Cyprinidae	*Poropuntius*	*normani*	This study	NA	diploid	non-miniature	20	844.2840135	844.2840135
LR15065	PRJNA1483025	SAMN61141623	Cyprinoidei	Cyprinidae	*Probarbus*	*jullieni*	This study	NA	polyploid	non-miniature	150	1350.403991	1350.403991
125LR15728	PRJNA1483025	SAMN61141624	Cyprinoidei	Cyprinidae	*Puntigrus*	*partipentazona*	This study	NA	diploid	non-miniature	2.95	597.0353715	597.0353715
GCF_018831695.1	PRJNA683758	SAMN17036344	Cyprinoidei	Cyprinidae	*Puntigrus*	*tetrazona*	NCBI	NA	diploid	non-miniature	4.78	579.1733795	579.1733795
EA1300	PRJNA1483025	SAMN61141627	Cyprinoidei	Cyprinidae	*Puntius*	sp	This study	NA	diploid	non-miniature	14.7	797.7085135	797.7085135
LR15257	PRJNA1483025	SAMN61141628	Cyprinoidei	Cyprinidae	*Rohanella*	*titteya*	This study	NA	diploid	non-miniature	3.66	752.468901	752.468901
124LR15727	PRJNA1483025	SAMN61141629	Cyprinoidei	Cyprinidae	*Rohtee*	*ogilbii*	This study	NA	diploid	non-miniature	40	707.9410645	707.9410645
145LR15691	PRJNA1483025	SAMN61141630	Cyprinoidei	Cyprinidae	*Sahyadria*	*denisonii*	This study	NA	diploid	non-miniature	19	697.7408185	697.7408185
LR15061	PRJNA1483025	SAMN61141631	Cyprinoidei	Cyprinidae	*Sawbwa*	*resplendens*	This study	NA	diploid	proportioned-dwarf	2.17	792.2622485	792.2622485
LR15564	PRJNA1483025	SAMN61141632	Cyprinoidei	Cyprinidae	*Scaphiodonichthys*	*acanthopterus*	This study	NA	diploid	non-miniature	31	780.9871525	780.9871525
LR15101	PRJNA1483025	SAMN61141633	Cyprinoidei	Cyprinidae	*Schismatorhynchos*	*heterorhynchos*	This study	NA	diploid	non-miniature	28.3	892.758514	892.758514
LR09392	PRJNA1483025	SAMN61141634	Cyprinoidei	Cyprinidae	*Semiplotus*	*cirrhosus*	This study	NA	diploid	non-miniature	18.5	790.364518	790.364518
GCF_001515605.1	PRJNA274017	SAMN03320099	Cyprinoidei	Cyprinidae	*Sinocyclocheilus*	*anshuiensis*	NCBI	1632	polyploid	non-miniature	10	NA	1632
GCF_001515645.1	PRJNA274017	SAMN03320097	Cyprinoidei	Cyprinidae	*Sinocyclocheilus*	*grahami*	NCBI	1750	polyploid	non-miniature	15	NA	1750
GCA_018148995.1	PRJNA669120	SAMN16435219	Cyprinoidei	Cyprinidae	*Sinocyclocheilus*	*maitianheensis*	NCBI	1680	polyploid	non-miniature	9.4	NA	1680
GCF_001515625.1	PRJNA274017	SAMN03320098	Cyprinoidei	Cyprinidae	*Sinocyclocheilus*	*rhinocerous*	NCBI	1655	polyploid	non-miniature	9.7	NA	1655
111LR15711	PRJNA1483025	SAMN61141635	Cyprinoidei	Cyprinidae	*Spinibarbichthys*	*denticulatus*	This study	NA	polyploid	non-miniature	41.5	1725.003298	1725.003298
LR15617	PRJNA1483025	SAMN61141636	Cyprinoidei	Cyprinidae	*Spinibarbichthys*	sp	This study	NA	polyploid	non-miniature	41.5	1769.692711	1769.692711
126LR15731	PRJNA1483025	SAMN61141637	Cyprinoidei	Cyprinidae	*Striuntius*	*lineatus*	This study	NA	diploid	non-miniature	5.95	652.6217305	652.6217305
LR06590	PRJNA1483025	SAMN61141638	Cyprinoidei	Cyprinidae	*Systomus*	*binduchitra*	This study	NA	diploid	non-miniature	6	649.663832	649.663832
KUFOS-RR-2022.P.1	PRJNA1393636	SAMN54310111	Cyprinoidei	Cyprinidae	*Tariqilabeo*	*periyarensis*	This study	NA	diploid	non-miniature	13.1	1038.771302	1038.771302
LR12880	PRJNA1483025	SAMN61141639	Cyprinoidei	Cyprinidae	*Thynnichthys*	*vaillanti*	This study	NA	diploid	non-miniature	20	946.176263	946.176263
LR09401	PRJNA1483025	SAMN61141640	Cyprinoidei	Cyprinidae	*Tor*	*putitora*	This study	NA	polyploid	non-miniature	150	1807.676521	1807.676521
LR01638	PRJNA1483025	SAMN61141642	Cyprinoidei	Danionidae	*Amblypharyngodon*	*chulabhornae*	This study	NA	diploid	non-miniature	4	561.2414775	561.2414775
fAmbSpp1	PRJEB19273	SAMEA104130232	Cyprinoidei	Danionidae	*Amblypharyngodon*	sp	NCBI-SRA	NA	diploid	non-miniature	5.5	582.919701	582.919701
LR15576	PRJNA1483025	SAMN61141643	Cyprinoidei	Danionidae	*Barilius*	*mesopotamicus*	This study	NA	diploid	non-miniature	5	632.689895	632.689895
LR15871	PRJNA1393767	SAMN54311767	Cyprinoidei	Danionidae	*Boraras*	*brigittae*	This study	NA	diploid	proportioned-dwarf	1.8	989.810021	989.810021
fBorMac1	PRJEB19273	SAMEA104026409	Cyprinoidei	Danionidae	*Boraras*	*maculatus*	NCBI-SRA	NA	diploid	proportioned-dwarf	2	923.9557445	923.9557445
LR12206	PRJNA1483025	SAMN61141644	Cyprinoidei	Danionidae	*Boraras*	*micros*	This study	NA	diploid	proportioned-dwarf	1.3	953.8686275	953.8686275
LR12190	PRJNA1483025	SAMN61141645	Cyprinoidei	Danionidae	*Boraras*	*urophthalmoides*	This study	NA	diploid	proportioned-dwarf	1.8	1043.537416	1043.537416
LR09154	PRJNA1483025	SAMN61141646	Cyprinoidei	Danionidae	*Brevibora*	*cheeya1*	This study	NA	diploid	proportioned-dwarf	2.8	1057.686109	1057.686109
LR10130	PRJNA1483025	SAMN61141647	Cyprinoidei	Danionidae	*Brevibora*	*cheeya2*	This study	NA	diploid	proportioned-dwarf	2.8	1029.485158	1029.485158
LR11616	PRJNA1483025	SAMN61141648	Cyprinoidei	Danionidae	*Brevibora*	*dorsiocellata*	This study	NA	diploid	proportioned-dwarf	2.3	866.844275	866.844275
LR15567	PRJNA1483025	SAMN61141649	Cyprinoidei	Danionidae	*Cabdio*	*morar*	This study	NA	diploid	non-miniature	10	549.1380975	549.1380975
fCheCac1	PRJEB19273	SAMEA104130244	Cyprinoidei	Danionidae	*Chela*	*cachius*	NCBI-SRA	NA	diploid	non-miniature	5	1008.955852	1008.955852
131LR08589	PRJNA1483025	SAMN61141650	Cyprinoidei	Danionidae	*Chelaethiops*	*elongatus*	This study	NA	diploid	non-miniature	4.8	579.350842	579.350842
LR15596	PRJNA1483025	SAMN61141651	Cyprinoidei	Danionidae	*Chelaethiops*	*bibie*	This study	NA	diploid	non-miniature	5	608.825888	608.825888

LR15597	PRJNA1483025	SAMN61141652	Cyprinoidei	Danionidae	*Chelaethiops*	sp2	This study	NA	diploid	non-miniature	5	593.233103	593.233103
GCF_903798145.1	PRJEB38583	SAMEA104240212	Cyprinoidei	Danionidae	*Danio*	*aesculapii*	NCBI	1381	diploid	non-miniature	3	NA	1381
GCA_903798035.1	PRJEB38565	SAMEA104026392	Cyprinoidei	Danionidae	*Danio*	*albolineatus*	NCBI	1463	diploid	non-miniature	3.4	NA	1463
GCA_903798125.1	PRJEB38567	SAMEA104026429	Cyprinoidei	Danionidae	*Danio*	*choprae*	NCBI	1102	diploid	non-miniature	3.1	NA	1102
LR01642	PRJNA1483025	SAMN61141653	Cyprinoidei	Danionidae	*Danio*	*erythromicron*	This study	NA	diploid	proportioned-dwarf	2.37	1006.792028	1006.792028
GCA_903798115.1	PRJEB38569	SAMEA104026422	Cyprinoidei	Danionidae	*Danio*	*jaintianensis*	NCBI	1122	diploid	non-miniature	8	NA	1122
GCA_903798195.1	PRJEB38580	SAMEA104130240	Cyprinoidei	Danionidae	*Danio*	*kyathit*	NCBI	1708	diploid	non-miniature	3.5	NA	1708
LR02056	PRJNA1483025	SAMN61141654	Cyprinoidei	Danionidae	*Danio*	*margaritatus*	This study	NA	diploid	proportioned-dwarf	2.1	928.546121	928.546121
GCA_944039275.1	PRJEB53407	SAMEA14088276	Cyprinoidei	Danionidae	*Danio*	*rerio*	NCBI	1413	diploid	non-miniature	3.8	NA	1413
GCA_903798205.1	PRJEB38571	SAMEA104026413	Cyprinoidei	Danionidae	*Danio*	*tinwini*	NCBI	1497	diploid	proportioned-dwarf	2.6	NA	1497
GCA_903798025.1	PRJEB38563	SAMEA104129915	Cyprinoidei	Danionidae	*Danionella*	*cerebrum*	NCBI	657.5	diploid	progenetic-miniature	1.4	NA	657.5
GCA_900490495.1	PRJEB27320	SAMEA104026433	Cyprinoidei	Danionidae	*Danionella*	*dracula*	NCBI	665.2	diploid	progenetic-miniature	1.7	NA	665.2
fDevDev2	PRJEB19273	SAMEA104132453	Cyprinoidei	Danionidae	*Devario*	*devario*	NCBI-SRA	NA	diploid	non-miniature	7	1617.316438	1617.316438
144LR15698	PRJNA1483025	SAMN61141655	Cyprinoidei	Danionidae	*Devario*	*sondhii*	This study	NA	diploid	non-miniature	6.5	1064.408718	1064.408718
fDanAeq2	PRJEB19273	SAMEA104132461	Cyprinoidei	Danionidae	*Devario*	sp	NCBI-SRA	NA	diploid	non-miniature	10	1613.603733	1613.603733
LR15598	PRJNA1483025	SAMN61141656	Cyprinoidei	Danionidae	*Engraulicypris*	sp1	This study	NA	diploid	non-miniature	7.5	574.0788735	574.0788735
LR15656	PRJNA1483025	SAMN61141657	Cyprinoidei	Danionidae	*Engraulicypris*	*spinifer*	This study	NA	diploid	non-miniature	7.5	554.3224095	554.3224095
116LR15716	PRJNA1483025	SAMN61141658	Cyprinoidei	Danionidae	*Esomus*	*danricus*	This study	NA	diploid	non-miniature	6	521.562969	521.562969
LR01653	PRJNA1483025	SAMN61141659	Cyprinoidei	Danionidae	*Esomus*	*metallicus*	This study	NA	diploid	non-miniature	7.5	513.0350255	513.0350255
LR12503	PRJNA1483025	SAMN61141660	Cyprinoidei	Danionidae	*Esomus*	sp	This study	NA	diploid	non-miniature	7.5	519.440578	519.440578
fHorAtu1	PRJEB19273	SAMEA104130248	Cyprinoidei	Danionidae	*Horadandia*	*atukorali*	NCBI-SRA	NA	diploid	proportioned-dwarf	1.93	585.840953	585.840953
LR01646	PRJNA1483025	SAMN61141661	Cyprinoidei	Danionidae	*Inlecypris*	*auropurpurea*	This study	NA	diploid	non-miniature	5	1316.965625	1316.965625
LR01674	PRJNA1483025	SAMN61141662	Cyprinoidei	Danionidae	*Kottelatia*	*brittani*	This study	NA	diploid	non-miniature	4.13	689.6199065	689.6199065
fLauMye1	PRJEB19273	SAMEA104130242	Cyprinoidei	Danionidae	*Laubuka*	*siamensis*	NCBI-SRA	NA	diploid	non-miniature	6.1	1336.049704	1336.049704
132LR09413	PRJNA1483025	SAMN61141663	Cyprinoidei	Danionidae	*Laubuka*	sp1	This study	NA	diploid	non-miniature	8	1300.971656	1300.971656
KUFOS-AN-2021.3.1	PRJNA1393636	SAMN54310112	Cyprinoidei	Danionidae	*Laubuka*	sp2	This study	NA	diploid	non-miniature	8	1271.61059	1271.61059
LR15601	PRJNA1483025	SAMN61141665	Cyprinoidei	Danionidae	*Leptocypris*	*niloticus*	This study	NA	diploid	non-miniature	11.8	620.067572	620.067572
fLucSpp1	PRJEB19273	SAMEA104130230	Cyprinoidei	Danionidae	*Luciosoma*	sp1	NCBI-SRA	NA	diploid	non-miniature	26	599.554698	599.554698
LR01659	PRJNA1483025	SAMN61141666	Cyprinoidei	Danionidae	*Luciosoma*	*pellegrinii*	This study	NA	diploid	non-miniature	26	618.508899	618.508899
LR15098	PRJNA1483025	SAMN61141667	Cyprinoidei	Danionidae	*Malayochela*	*maassi*	This study	NA	diploid	non-miniature	4	828.8339765	828.8339765
LR15568	PRJNA1483025	SAMN61141670	Cyprinoidei	Danionidae	*Microdevario*	*kubotai*	This study	NA	diploid	proportioned-dwarf	1.9	1010.972865	1010.972865
LR15650	PRJNA1483025	SAMN61141671	Cyprinoidei	Danionidae	*Microrasbora*	*rubescens*	This study	NA	diploid	proportioned-dwarf	2.63	1019.876315	1019.876315
121LR15723	PRJNA1483025	SAMN61141673	Cyprinoidei	Danionidae	*Nematabramis*	*everetti*	This study	NA	diploid	non-miniature	10.5	861.4281705	861.4281705
LR15099	PRJNA1483025	SAMN61141674	Cyprinoidei	Danionidae	*Nematabramis*	*steindachneri*	This study	NA	diploid	non-miniature	11.2	831.6380315	831.6380315
LR14062	PRJNA1483025	SAMN61141675	Cyprinoidei	Danionidae	*Neochela*	*dadiburjori*	This study	NA	diploid	non-miniature	3	761.266707	761.266707
LR15289	PRJNA1483025	SAMN61141676	Cyprinoidei	Danionidae	*Opsaridium*	sp1	This study	NA	diploid	non-miniature	12	672.00933	672.00933
LR15290	PRJNA1483025	SAMN61141677	Cyprinoidei	Danionidae	*Opsaridium*	*zambezense*	This study	NA	diploid	non-miniature	12	644.8919075	644.8919075
LR15602	PRJNA1483025	SAMN61141678	Cyprinoidei	Danionidae	*Opsaridium*	*ubangiense*	This study	NA	diploid	non-miniature	12	686.125769	686.125769
142LR15666	PRJNA1483025	SAMN61141679	Cyprinoidei	Danionidae	*Opsarius*	sp1	This study	NA	diploid	non-miniature	9.1	596.7035535	596.7035535
LR02136	PRJNA1483025	SAMN61141681	Cyprinoidei	Danionidae	*Opsarius*	sp2	This study	NA	diploid	non-miniature	9.1	565.8439115	565.8439115
LR12240	PRJNA1483025	SAMN61141680	Cyprinoidei	Danionidae	*Opsarius*	*pulchellus*	This study	NA	diploid	non-miniature	11	588.440035	588.440035
LR15081	PRJNA1483025	SAMN61141682	Cyprinoidei	Danionidae	*Pectenocypris*	sp	This study	NA	diploid	non-miniature	3.5	783.181521	783.181521
fRaiGut1	PRJEB19273	SAMEA104130243	Cyprinoidei	Danionidae	*Raiamas*	*guttatus*	NCBI-SRA	NA	diploid	non-miniature	30	623.62552	623.62552
LR15292	PRJNA1483025	SAMN61141684	Cyprinoidei	Danionidae	*Raiamas*	*moorii*	This study	NA	diploid	non-miniature	20	619.3998015	619.3998015
105LR15605	PRJNA1483025	SAMN61141685	Cyprinoidei	Danionidae	*Raiamas*	sp	This study	NA	diploid	non-miniature	20	686.7324995	686.7324995

LR10786	PRJNA1483025	SAMN61141686	Cyprinoidei	Danionidae	*Rasbora*	*cephalotaenia*	This study	NA	diploid	non-miniature	7.73	766.9291225	766.9291225
KUFOS-AN-2021.2.1	PRJNA1393636	SAMN54310113	Cyprinoidei	Danionidae	*Rasbora*	*dandia*	This study	NA	diploid	non-miniature	9.9	540.6536305	540.6536305
fRasDan1	PRJEB19273	SAMEA104130236	Cyprinoidei	Danionidae	*Rasbora*	*daniconius*	NCBI-SRA	NA	diploid	non-miniature	11	675.4717815	675.4717815
LR15347	PRJNA1483025	SAMN61141688	Cyprinoidei	Danionidae	*Rasbora*	*kalbarensis*	This study	NA	diploid	proportioned-dwarf	2.5	814.203926	814.203926
LR15877	PRJNA1393767	SAMN54311768	Cyprinoidei	Danionidae	*Rasbora*	*kalochroma*	This study	NA	diploid	non-miniature	6	1290.849861	1290.849861
LR10804	PRJNA1483025	SAMN61141689	Cyprinoidei	Danionidae	*Rasbora*	*kottelati*	This study	NA	diploid	non-miniature	7	1246.322742	1246.322742
LR09739	PRJNA1483025	SAMN61141690	Cyprinoidei	Danionidae	*Rasbora*	*myersi*	This study	NA	diploid	non-miniature	7.3	766.9803475	766.9803475
fRasRas1	PRJEB19273	SAMEA104130231	Cyprinoidei	Danionidae	*Rasbora*	*rasbora*	NCBI-SRA	NA	diploid	non-miniature	10	914.083791	914.083791
LR09847	PRJNA1483025	SAMN61141691	Cyprinoidei	Danionidae	*Rasbora*	*trilineata*	This study	NA	diploid	non-miniature	6	885.3081555	885.3081555
LR05463	PRJNA1483025	SAMN61141692	Cyprinoidei	Danionidae	*Rasboroides*	*pallidus*	This study	NA	diploid	non-miniature	3.6	628.5166625	628.5166625
LR12212	PRJNA1483025	SAMN61141693	Cyprinoidei	Danionidae	*Rasbosoma*	*spilocerca*	This study	NA	diploid	proportioned-dwarf	2.6	901.6240925	901.6240925
KUFOS-AN-2021.6.1	PRJNA1393636	SAMN54310114	Cyprinoidei	Danionidae	*Salmostoma*	*balookee*	This study	NA	diploid	non-miniature	14.5	583.0889565	583.0889565
EA0634	PRJNA1483025	SAMN61141695	Cyprinoidei	Danionidae	*Thryssocypris*	*tonlesapensis*	This study	NA	diploid	non-miniature	6.4	447.905259	447.905259
LR08172	PRJNA1483025	SAMN61141697	Cyprinoidei	Danionidae	*Trigonopoma*	*gracile*	This study	NA	diploid	non-miniature	5.5	872.3102705	872.3102705
LR10181	PRJNA1483025	SAMN61141698	Cyprinoidei	Danionidae	*Trigonopoma*	*pauciperforatum*	This study	NA	diploid	non-miniature	5	984.3918645	984.3918645
LR11656	PRJNA1483025	SAMN61141699	Cyprinoidei	Danionidae	*Trigonopoma*	*sp*	This study	NA	diploid	non-miniature	5	1076.334203	1076.334203
LR08812	PRJNA1483025	SAMN61141700	Cyprinoidei	Danionidae	*Trigonostigma*	*espei*	This study	NA	diploid	proportioned-dwarf	2.5	1111.510812	1111.510812
LR11086	PRJNA1483025	SAMN61141701	Cyprinoidei	Danionidae	*Trigonostigma*	*heteromorpha*	This study	NA	diploid	proportioned-dwarf	2.84	1115.305677	1115.305677
GCA_949357685.1	PRJEB60324	SAMEA11296538	Cyprinoidei	Gobionidae	*Gobio*	*gobio*	NCBI	1460	diploid	non-miniature	13	NA	1460
GCA_023029165.1	PRJNA669452	SAMN16453471	Cyprinoidei	Gobionidae	*Gobiocypris*	*rarus*	NCBI	1134	diploid	non-miniature	5.9	NA	1134
LR15569	PRJNA1483025	SAMN61141702	Cyprinoidei	Gobionidae	*Hemibarbus*	*maculatus*	This study	NA	diploid	non-miniature	34	1025.660767	1025.660767
LR15570	PRJNA1483025	SAMN61141703	Cyprinoidei	Gobionidae	*Ladislavia*	*taczanowskii*	This study	NA	diploid	non-miniature	12	1113.559064	1113.559064
LR15062	PRJNA1483025	SAMN61141704	Cyprinoidei	Gobionidae	*Microphysogobio*	*tafangensis*	This study	NA	diploid	non-miniature	7.8	947.9214225	947.9214225
GCA_018749465.1	PRJNA686690	SAMN17120673	Cyprinoidei	Gobionidae	*Paracanthobrama*	*guichenoti*	NCBI	1087	diploid	non-miniature	41.5	NA	1087
GCA_024679245.1	PRJNA843929	SAMN28772710	Cyprinoidei	Gobionidae	*Pseudorasbora*	*parva*	NCBI	1228	diploid	non-miniature	9.5	NA	1228
GCA_023566225.1	PRJNA799923	SAMN25220526	Cyprinoidei	Gobionidae	*Romanogobio*	*albipinnatus*	NCBI	1428	diploid	non-miniature	11.5	NA	1428
LR15571	PRJNA1483025	SAMN61141705	Cyprinoidei	Gobionidae	*Sarcocheilichthys*	*hainanensis*	This study	NA	diploid	non-miniature	7.5	1208.260729	1208.260729
LR15579	PRJNA1483025	SAMN61141706	Cyprinoidei	Gobionidae	*Squalidus*	*atromaculatus*	This study	NA	diploid	non-miniature	18.8	994.7189	994.7189
LR15106	PRJNA1483025	SAMN61141707	Cyprinoidei	Leptobarbidae	*Leptobarbus*	*hoeveni*	This study	NA	diploid	non-miniature	100	1001.959424	1001.959424
GCA_963993115.1	PRJEB73975	SAMEA11296536	Cyprinoidei	Leuciscidae	*Abramis*	*brama*	NCBI	NA	diploid	non-miniature	70	1081.577457	1081.577457
2180_2012	PRJNA1483025	SAMN61141709	Cyprinoidei	Leuciscidae	*Alburnoides*	*bipunctatus*	This study	NA	diploid	non-miniature	13	1096.164941	1096.164941
108LR15742	PRJNA1483025	SAMN61141710	Cyprinoidei	Leuciscidae	*Chrosomus*	*erythrogaster*	This study	NA	diploid	non-miniature	6.1	1134.19284	1134.19284
GCA_027497735.1	PRJNA806520	SAMN25889353	Cyprinoidei	Leuciscidae	*Cyprinella*	*lepida*	NCBI	768.2	diploid	non-miniature	7.1	NA	768.2
GCA_027497675.1	PRJNA806518	SAMN25889351	Cyprinoidei	Leuciscidae	*Cyprinella*	*lutrensis*	NCBI	NA	diploid	non-miniature	7.1	1010.965464	1010.965464
GCA_027361055.1	PRJNA806519	SAMN25889352	Cyprinoidei	Leuciscidae	*Cyprinella*	*venusta*	NCBI	805.4	diploid	non-miniature	15.5	NA	805.4
LR15252	PRJNA1483025	SAMN61141712	Cyprinoidei	Leuciscidae	*Dionda*	*flavipinnis*	This study	NA	diploid	non-miniature	7.6	817.7215975	817.7215975
110LR15746	PRJNA1483025	SAMN61141713	Cyprinoidei	Leuciscidae	*Gila*	*pandora*	This study	NA	diploid	non-miniature	15	1133.136695	1133.136695
GCA_030378365.1	PRJNA887477	SAMN32668620	Cyprinoidei	Leuciscidae	*Hybognathus*	*amarus*	NCBI	NA	diploid	non-miniature	9.3	937.2453165	937.2453165
GCA_023718395.1	PRJNA818486	SAMN26865216	Cyprinoidei	Leuciscidae	*Leucaspius*	*delineatus*	NCBI	1153	diploid	non-miniature	9	NA	1153
GCA_021554675.1	PRJNA768343	SAMN22028852	Cyprinoidei	Leuciscidae	*Leuciscus*	*idus*	NCBI	1104	diploid	non-miniature	85	NA	1104
2223_2013	PRJNA1483025	SAMN61141715	Cyprinoidei	Leuciscidae	*Leuciscus*	*leuciscus*	This study	NA	diploid	non-miniature	25	1159.306152	1159.306152
GCA_030578275.1	PRJNA947235	SAMN33845343	Cyprinoidei	Leuciscidae	*Meda*	*fulgida*	NCBI	882.1	diploid	non-miniature	6.5	NA	882.1
LR15254	PRJNA1483025	SAMN61141716	Cyprinoidei	Leuciscidae	*Notropis*	*chalybaeus*	This study	NA	diploid	non-miniature	5	1230.957706	1230.957706
GCA_949152265.1	PRJEB59820	SAMEA11296539	Cyprinoidei	Leuciscidae	*Phoxinus*	*phoxinus*	NCBI	NA	diploid	non-miniature	10	980.731219	980.731219
GCF_016745375.1	PRJNA565199	SAMN12875914	Cyprinoidei	Leuciscidae	*Pimephales*	*promelas*	NCBI	1066	diploid	non-miniature	8.2	NA	1066

109LR15744	PRJNA1483025	SAMN61141718	Cyprinoidei	Leuciscidae	*Ptychocheilus*	*lucius*	This study	NA	diploid	non-miniature	18	1094.060096	1094.060096
LR15251	PRJNA1483025	SAMN61141719	Cyprinoidei	Leuciscidae	*Rhinichthys*	*cataractae*	This study	NA	diploid	non-miniature	10.4	1101.099443	1101.099443
LR15572	PRJNA1483025	SAMN61141720	Cyprinoidei	Leuciscidae	*Rhynchocypris*	*lagowskii*	This study	NA	diploid	non-miniature	18.5	1230.470575	1230.470575
GCA_951802725.1	PRJEB62672	SAMEA11296543	Cyprinoidei	Leuciscidae	*Rutilus*	*rutilus*	NCBI	NA	diploid	non-miniature	50	1063.445747	1063.445747
GCA_024453875.1	PRJNA841808	SAMN28618522	Cyprinoidei	Leuciscidae	*Scardinius*	*erythrophthalmus*	NCBI	NA	diploid	non-miniature	35	1103.761005	1103.761005
GCA_031834385.1	PRJNA994924	SAMN36452606	Cyprinoidei	Leuciscidae	*Semotilus*	*atromaculatus*	NCBI	NA	diploid	non-miniature	24.5	1174.221529	1174.221529
GCA_949319135.1	PRJEB60239	SAMEA11296547	Cyprinoidei	Leuciscidae	*Squalius*	*cephalus*	NCBI	NA	diploid	non-miniature	60	1084.922084	1084.922084
1450_2013	PRJNA1483025	SAMN61141725	Cyprinoidei	Leuciscidae	*Telestes*	*muticellus*	This study	NA	diploid	non-miniature	17	1160.188897	1160.188897
GCA_030578255.1	PRJNA947235	SAMN33845349	Cyprinoidei	Leuciscidae	*Tiaroga*	*cobitis*	NCBI	1320	diploid	non-miniature	6	NA	1320
GCA_022828995.1	PRJNA798300	SAMN25064631	Cyprinoidei	Leuciscidae	*Vimba*	*vimba*	NCBI	1054	diploid	non-miniature	35	NA	1054
LR12004	PRJEB15524	SAMEA4467423	Cyprinoidei	Paedocyprididae	*Paedocypris*	*carbunculus*	NCBI-SRA	NA	diploid	progenetic-miniature	1.15	422.0606155	422.0606155
GCA_028031935.1	PRJNA474779	SAMN09373892	Cyprinoidei	Paedocyprididae	*Paedocypris*	*micromegethes*1	NCBI	421.3	diploid	progenetic-miniature	1.3	NA	421.3
LR7898	PRJEB15524	SAMEA4467424	Cyprinoidei	Paedocyprididae	*Paedocypris*	*micromegethes*2	NCBI-SRA	NA	diploid	progenetic-miniature	1.3	407.596544	407.596544
LR15325	PRJNA1483025	SAMN61141726	Cyprinoidei	Paedocyprididae	*Paedocypris*	sp2	This study	NA	diploid	progenetic-miniature	1.3	517.668667	517.668667
LR15863A	PRJNA1393767	SAMN54311765	Cyprinoidei	Paedocyprididae	*Paedocypris*	sp1	This study	NA	diploid	progenetic-miniature	1.3	401.451712	401.451712
LR09467	PRJNA1483025	SAMN61141727	Cyprinoidei	Psilorhynchidae	*Psilorhynchus*	*brachyrhynchus*	This study	NA	diploid	non-miniature	5.1	482.2281655	482.2281655
LR15089	PRJNA1483025	SAMN61141728	Cyprinoidei	Psilorhynchidae	*Psilorhynchus*	sp2	This study	NA	diploid	non-miniature	4.9	389.6027315	389.6027315
100LR08982	PRJNA1483025	SAMN61141729	Cyprinoidei	Sundadanionidae	*Fangfangia*	*spinicleithralis*	This study	NA	diploid	progenetic-miniature	2.3	761.3466415	761.3466415
LR15870	PRJNA1393767	SAMN54311766	Cyprinoidei	Sundadanionidae	*Sundadanio*	*atomus*	This study	NA	diploid	progenetic-miniature	1.6	654.471117	654.471117
LR10372	PRJNA1483025	SAMN61141730	Cyprinoidei	Sundadanionidae	*Sundadanio*	*goblinus*	This study	NA	diploid	progenetic-miniature	1.9	679.917281	679.917281
LR15082	PRJNA1483025	SAMN61141731	Cyprinoidei	Sundadanionidae	*Sundadanio*	*margarition*	This study	NA	diploid	progenetic-miniature	2	716.7551635	716.7551635
101LR12751	PRJNA1483025	SAMN61141732	Cyprinoidei	Sundadanionidae	*Sundadanio*	*retiarius*	This study	NA	diploid	progenetic-miniature	1.9	752.2425955	752.2425955
LR15110	PRJNA1483025	SAMN61141733	Cyprinoidei	Tanichthyidae	*Tanichthys*	*albonudes*	This study	NA	diploid	proportioned-dwarf	2.77	1071.863989	1071.863989
LR02462	PRJNA1483025	SAMN61141734	Cyprinoidei	Tanichthyidae	*Tanichthys*	*micagemmae*	This study	NA	diploid	proportioned-dwarf	2.3	1093.443826	1093.443826
1545_2010	PRJNA1483025	SAMN61141735	Cyprinoidei	Tincidae	*Tinca*	*tinca*	This study	NA	diploid	non-miniature	70	1048.69225	1048.69225
GCA_003731715.1	PRJNA477399	SAMN09469597	Cyprinoidei	Xenocyprididae	*Anabarilius*	*grahami*	NCBI	991.9	diploid	non-miniature	15.6	NA	991.9
118LR15719	PRJNA1483025	SAMN61141736	Cyprinoidei	Xenocyprididae	*Candidia*	*pingtungensis*	This study	NA	diploid	non-miniature	11.4	780.192702	780.192702
GCA_024489055.1	PRJNA827856	SAMN27624467	Cyprinoidei	Xenocyprididae	*Chanodichthys*	*erythropterus*	NCBI	1085	diploid	non-miniature	80	NA	1085
GCF_019924925.1	PRJNA745929	SAMN20170273	Cyprinoidei	Xenocyprididae	*Ctenopharyngodon*	*idella*	NCBI	893.2	diploid	non-miniature	80	NA	893.2
GCA_028476615.1	PRJNA700093	SAMN31113473	Cyprinoidei	Xenocyprididae	*Culter*	*alburnus*	NCBI	1055	diploid	non-miniature	60	NA	1055
GCA_037101425.1	PRJNA1035000	SAMN38080342	Cyprinoidei	Xenocyprididae	*Elopichthys*	*bambusa*	NCBI	827.7	diploid	non-miniature	180	NA	827.7
103LR15622	PRJNA1483025	SAMN61141737	Cyprinoidei	Xenocyprididae	*Hemiculter*	sp	This study	NA	diploid	non-miniature	20.5	977.935087	977.935087
GCA_004764525.1	PRJNA305140	SAMN04317213	Cyprinoidei	Xenocyprididae	*Hypophthalmichthys*	*molitrix*	NCBI	1104	diploid	non-miniature	100	NA	1104
GCA_019925145.1	PRJNA657698	SAMN15835045	Cyprinoidei	Xenocyprididae	*Hypophthalmichthys*	*nobilis*	NCBI	857	diploid	non-miniature	146	NA	857
GCF_018812025.1	PRJNA343584	SAMN18522313	Cyprinoidei	Xenocyprididae	*Megalobrama*	*amblycephala*	NCBI	1109	diploid	non-miniature	180	NA	1109
102LR15621	PRJNA1483025	SAMN61141738	Cyprinoidei	Xenocyprididae	*Opsariichthys*	*bidens*	This study	NA	diploid	non-miniature	16	905.3265225	905.3265225
128LR15724	PRJNA1483025	SAMN61141739	Cyprinoidei	Xenocyprididae	*Opsariichthys*	*pachycephalus*	This study	NA	diploid	non-miniature	12.2	734.149207	734.149207
LR15574	PRJNA1483025	SAMN61141740	Cyprinoidei	Xenocyprididae	*Oxygaster*	*pointoni*	This study	NA	diploid	non-miniature	15.5	862.965098	862.965098
GCA_034642465.1	PRJNA1028840	SAMN37853698	Cyprinoidei	Xenocyprididae	*Zacco*	*platypus*	NCBI	815	diploid	non-miniature	13	NA	815
LR15097	PRJNA1483025	SAMN61141741	Gyrinocheiloidei	Gyrinocheilidae	*Gyrinocheilus*	*pustulosus*	This study	NA	diploid	non-miniature	28	478.3126755	478.3126755
104EA0138	PRJNA1483025	SAMN61141742	Gyrinocheiloidei	Gyrinocheilidae	*Gyrinocheilus*	sp	This study	NA	diploid	non-miniature	28	475.3712995	475.3712995

Table S13. Miniature fishes within Cypriniformes, comprising progenetic miniatures and proportioned dwarfs, with indication of which species were included in the present study.

Suborder	Family	Genus	Miniature species	Miniaturization type	Included_309Cyp_dataset	Distribution	Reference	Remarks
Cobitoidei	Balitoridae	*Homalopteroides*	*Homalopteroides tweediei*	Proportional dwarf	yes	Asian freshwaters	Kottelat & Vidthayanon (1993)	Genus comprises both miniature (n=1) and non-miniature species (n=12)
Cobitoidei	Balitoridae	*Neohomaloptera*	*Neohomaloptera johorensis*	Proportional dwarf	yes	Asian freshwaters	Kottelat & Vidthayanon (1993)	Monotypic genus comprising a miniature species (n=1)
Cobitoidei	Barbuccidae	*Barbucca*	*Barbucca elongatus*	Proportional dwarf	yes	Asian freshwaters	Vasil'eva & Vasil'ev (2013)	Genus comprises only miniature species (n=3)
Cobitoidei	Barbuccidae	*Barbucca*	*Barbucca diabolica*	Proportional dwarf	no	Asian freshwaters	Kottelat & Vidthayanon (1993)	
Cobitoidei	Barbuccidae	*Barbucca*	*Barbucca heokhuii*	Proportional dwarf	no	Asian freshwaters	Kottelat (2025)	
Cobitoidei	Cobitidae	*Kottelatlimia*	*Kottelatlimia katik*	Proportional dwarf	yes	Asian freshwaters	Kottelat & Vidthayanon (1993)	Genus comprises both miniature (n=1) and non-miniature species (n=2)
Cobitoidei	Cobitidae	*Pangio*	*Pangio bhujia*	Proportional dwarf	yes	Asian freshwaters	Anoop et al. (2019)	Genus comprises both miniature (n=4) and non-miniature species (n=31)
Cobitoidei	Cobitidae	*Pangio*	*Pangio ammophila*	Proportional dwarf	no	Asian freshwaters	Britz et al. (2012)	
Cobitoidei	Cobitidae	*Pangio*	*Pangio longimanus*	Proportional dwarf	no	Asian freshwaters	Britz & Kottelat (2010)	
Cobitoidei	Cobitidae	*Pangio*	*Pangio pathala*	Proportional dwarf	no	Asian freshwaters	Sundar et al. (2022)	
Cobitoidei	Cobitidae	*Gitchak*	*Gitchak nakana*	Proportional dwarf	no	Asian freshwaters	Britz et al. (2026)	Monotypic genus comprising a miniature species (n=1)
Cobitoidei	Nemacheilidae	*Tuberoschistura*	*Tuberoschistura cambodgiensis*	Proportional dwarf	no	Asian freshwaters	Kottelat & Vidthayanon (1993)	Genus comprises both miniature (n=1) and non-miniature species (n=1)
Cobitoidei	Nemacheilidae	*Physoschistura*	*Physoschistura mango*	Proportional dwarf	no	Asian freshwaters	Conway & Kottelat (2023)	Genus comprises both miniature (n=1) and non-miniature species (n=8)
Cobitoidei	Nemacheilidae	*Schistura*	*Schistura diminuta*	Proportional dwarf	no	Asian freshwaters	Ou et al. (2011)	Genus comprises majority of non-miniature species (n=244)
Cobitoidei	Nemacheilidae	*Schistura*	*Schistura rikiki*	Proportional dwarf	no	Asian freshwaters	Kottelat (2000)	
Cyprinoidei	Cyprinidae	*Sawbwa*	*Sawbwa resplendens*	Proportional dwarf	yes	Asian freshwaters	Kottelat & Vidthayanon (1993)	Monotypic genus comprising a single species (n=1); considered here a miniature based on Kottelat & Vidthayanon (1993) but may grow bigger
Cyprinoidei	Cyprinidae	*Garra*	*Garra lancrenonensis*	Proportional dwarf	no	African freshwaters	Conway & Moritz (2006) and ref herein	Genus comprises majority of non-miniature species (n=192)
Cyprinoidei	Cyprinidae	*Enteromius*	*Enteromius candens*	Proportional dwarf	no	African freshwaters	Conway & Moritz (2006) and ref herein	Genus comprises majority of non-miniature species (n=223); the number of miniature species reported maybe underrepresented ?
Cyprinoidei	Cyprinidae	*Enteromius*	*Enteromius erythrozonus*	Proportional dwarf	no	African freshwaters	Conway & Moritz (2006) and ref herein	
Cyprinoidei	Cyprinidae	*Enteromius*	*Enteromius kissiensis*	Proportional dwarf	no	African freshwaters	Conway & Moritz (2006) and ref herein	
Cyprinoidei	Cyprinidae	*Enteromius*	*Enteromius nigrifilis*	Proportional dwarf	no	African freshwaters	Conway & Moritz (2006) and ref herein	
Cyprinoidei	Cyprinidae	*Enteromius*	*Enteromius nigroluteus*	Proportional dwarf	no	African freshwaters	Conway & Moritz (2006) and ref herein	
Cyprinoidei	Cyprinidae	*Enteromius*	*Enteromius papilio*	Proportional dwarf	no	African freshwaters	Conway & Moritz (2006) and ref herein	
Cyprinoidei	Cyprinidae	*Enteromius*	*Enteromius pumilus*	Proportional dwarf	no	African freshwaters	Conway & Moritz (2006) and ref herein	
Cyprinoidei	Cyprinidae	*Enteromius*	*Enteromius sylvaticus*	Proportional dwarf	no	African freshwaters	Conway & Moritz (2006) and ref herein	
Cyprinoidei	Cyprinidae	*Enteromius*	*Enteromius tongaensis*	Proportional dwarf	no	African freshwaters	Conway & Moritz (2006) and ref herein	
Cyprinoidei	Cyprinidae	*Barboides*	*Barboides gracilis*	Proportional dwarf	yes	African freshwaters	Conway & Moritz (2006)	Genus comprises only miniature species (n=2); here considered as a proportioned dwarf but the two species of Barboides display some progenetic features (Conway et al. 2017)
Cyprinoidei	Cyprinidae	*Barboides*	*Barboides britzi*	Proportional dwarf	no	African freshwaters	Conway & Moritz (2006)	
Cyprinoidei	Danionidae	*Danio*	*Danio erythromicron*	Proportional dwarf	yes	Asian freshwaters	Kottelat & Vidthayanon (1993)	Genus comprises both miniature (n=3) and non-miniature species (n=24)
Cyprinoidei	Danionidae	*Danio*	*Danio margaritatus*	Proportional dwarf	yes	Asian freshwaters	Roberts (2007)	
Cyprinoidei	Danionidae	*Danio*	*Danio tinwini*	Proportional dwarf	yes	Asian freshwaters	Kullander & Fang (2009)	
Cyprinoidei	Danionidae	*Danionella*	*Danionella cerebrum*	Progenetic miniature	yes	Asian freshwaters	Britz et al. (2021)	Genus comprises only miniature species (n=5)
Cyprinoidei	Danionidae	*Danionella*	*Danionella dracula*	Progenetic miniature	yes	Asian freshwaters	Britz et al. (2021)	
Cyprinoidei	Danionidae	*Danionella*	*Danionella mirifica*	Progenetic miniature	no	Asian freshwaters	Britz et al. (2021)	
Cyprinoidei	Danionidae	*Danionella*	*Danionella priapus*	Progenetic miniature	no	Asian freshwaters	Britz et al. (2021)	
Cyprinoidei	Danionidae	*Danionella*	*Danionella translucida*	Progenetic miniature	no	Asian freshwaters	Britz et al. (2021)	
Cyprinoidei	Danionidae	*Microdevario*	*Microdevario kubotai*	Proportional dwarf	yes	Asian freshwaters	Kottelat & Witte (1999)	Genus comprises only miniature species (n=4)
Cyprinoidei	Danionidae	*Microdevario*	*Microdevario gatesi*	Proportional dwarf	no	Asian freshwaters	Kottelat & Vidthayanon (1993)	
Cyprinoidei	Danionidae	*Microdevario*	*Microdevario microphthalma*	Proportional dwarf	no	Asian freshwaters	Jiang et al. (2008)	
Cyprinoidei	Danionidae	*Microdevario*	*Microdevario nanus*	Proportional dwarf	no	Asian freshwaters	Kottelat & Witte (1999)	
Cyprinoidei	Danionidae	*Microrasbora*	*Microrasbora rubescens*	Proportional dwarf	yes	Asian freshwaters	Kottelat & Vidthayanon (1993)	Monotypic genus comprising a miniature species (n=1); considered here a miniature based on Kottelat & Vidthayanon (1993) but may grow bigger
Cyprinoidei	Danionidae	*Neobola*	*Neobola stellae*	Proportional dwarf	no	African freshwaters	Daget et al. (1984)	Genus comprises both miniature (n=1) and non-miniature species (n=4)

Cyprinoidei	Danionidae	*Boraras*	*Boraras brigittae*	Proportional dwarf	yes	Asian freshwaters	Kottelat & Vidthayanon (1993)	Genus comprises only miniature species (n=6)
Cyprinoidei	Danionidae	*Boraras*	*Boraras maculatus*	Proportional dwarf	yes	Asian freshwaters	Kottelat & Vidthayanon (1993)	
Cyprinoidei	Danionidae	*Boraras*	*Boraras merah*	Proportional dwarf	no	Asian freshwaters	Kottelat & Vidthayanon (1993)	
Cyprinoidei	Danionidae	*Boraras*	*Boraras micros*	Proportional dwarf	yes	Asian freshwaters	Kottelat & Vidthayanon (1993)	
Cyprinoidei	Danionidae	*Boraras*	*Boraras naevus*	Proportional dwarf	no	Asian freshwaters	Conway & Kottelat (2011)	
Cyprinoidei	Danionidae	*Boraras*	*Boraras urophthalmoides*	Proportional dwarf	yes	Asian freshwaters	Kottelat & Vidthayanon (1993)	
Cyprinoidei	Danionidae	*Brevibora*	*Brevibora cheeya*	Proportional dwarf	yes	Asian freshwaters	Liao & Tan (2011)	Genus comprises only miniature species (n=3)
Cyprinoidei	Danionidae	*Brevibora*	*Brevibora dorsiocellata*	Proportional dwarf	yes	Asian freshwaters	Liao & Tan (2011)	
Cyprinoidei	Danionidae	*Brevibora*	*Brevibora exilis*	Proportional dwarf	no	Asian freshwaters	Liao & Tan (2014)	
Cyprinoidei	Danionidae	*Horadandia*	*Horadandia atukorali*	Proportional dwarf	yes	Asian freshwaters	Kottelat & Vidthayanon (1993)	Genus comprises only miniature species (n=2)
Cyprinoidei	Danionidae	*Horadandia*	*Horadandia brittani*	Proportional dwarf	no	Asian freshwaters	Batuwita et al. (2013)	
Cyprinoidei	Danionidae	*Rasbora*	*Rasbora kalbarensis*	Proportional dwarf	yes	Asian freshwaters	Kottelat & Vidthayanon (1993)	Genus comprises majority of non-miniature species (n=89)
Cyprinoidei	Danionidae	*Rasbosoma*	*Rasbosoma spilocerca*	Proportional dwarf	yes	Asian freshwaters	Kottelat & Vidthayanon (1993)	Monotypic genus comprising a miniature species (n=1)
Cyprinoidei	Danionidae	*Trigonostigma*	*Trigonostigma espei*	Proportional dwarf	yes	Asian freshwaters	Kottelat & Witte (1999)	Genus comprises only miniature species (n=5)
Cyprinoidei	Danionidae	*Trigonostigma*	*Trigonostigma hengeli*	Proportional dwarf	no	Asian freshwaters	Kottelat & Witte (1999)	
Cyprinoidei	Danionidae	*Trigonostigma*	*Trigonostigma heteromorpha*	Proportional dwarf	yes	Asian freshwaters	Kottelat & Witte (1999)	
Cyprinoidei	Danionidae	*Trigonostigma*	*Trigonostigma somphongsi*	Proportional dwarf	no	Asian freshwaters	Kottelat & Witte (1999)	
Cyprinoidei	Danionidae	*Trigonostigma*	*Trigonostigma truncata*	Proportional dwarf	no	Asian freshwaters	Tan (2020)	
Cyprinoidei	Paedocyprididae	*Paedocypris*	*Paedocypris carbunculus*	Progenetic miniature	yes	Asian freshwaters	Britz & Kottelat (2008)	Genus comprises only miniature species (n=3)
Cyprinoidei	Paedocyprididae	*Paedocypris*	*Paedocypris micromegethes*	Progenetic miniature	yes	Asian freshwaters	Kottelat et al. (2006)	
Cyprinoidei	Paedocyprididae	*Paedocypris*	*Paedocypris progenetica*	Progenetic miniature	no	Asian freshwaters	Kottelat et al. (2006)	
Cyprinoidei	Sundadanionidae	*Fangfangia*	*Fangfangia spinicleithralis*	Progenetic miniature	yes	Asian freshwaters	Britz et al. (2011)	Monotypic genus comprising a miniature species (n=1)
Cyprinoidei	Sundadanionidae	*Sundadanio*	*Sundadanio atomus*	Progenetic miniature	yes	Asian freshwaters	Conway et al. (2011)	Genus comprises only miniature species (n=8)
Cyprinoidei	Sundadanionidae	*Sundadanio*	*Sundadanio axelrodi*	Progenetic miniature	no	Asian freshwaters	Conway et al. (2011)	
Cyprinoidei	Sundadanionidae	*Sundadanio*	*Sundadanio echinus*	Progenetic miniature	no	Asian freshwaters	Conway et al. (2011)	
Cyprinoidei	Sundadanionidae	*Sundadanio*	*Sundadanio gargula*	Progenetic miniature	no	Asian freshwaters	Conway et al. (2011)	
Cyprinoidei	Sundadanionidae	*Sundadanio*	*Sundadanio goblinus*	Progenetic miniature	yes	Asian freshwaters	Conway et al. (2011)	
Cyprinoidei	Sundadanionidae	*Sundadanio*	*Sundadanio margarition*	Progenetic miniature	yes	Asian freshwaters	Conway et al. (2011)	
Cyprinoidei	Sundadanionidae	*Sundadanio*	*Sundadanio retiarius*	Progenetic miniature	yes	Asian freshwaters	Conway et al. (2011)	
Cyprinoidei	Sundadanionidae	*Sundadanio*	*Sundadanio rubellus*	Progenetic miniature	no	Asian freshwaters	Conway et al. (2011)	
Cyprinoidei	Tanichthyidae	*Tanichthys*	*Tanichthys albonubes*	Proportional dwarf	yes	Asian freshwaters	Freyhof & Herder (2001)	Genus comprises only miniature species (n=9)
Cyprinoidei	Tanichthyidae	*Tanichthys*	*Tanichthys micagemmae*	Proportional dwarf	yes	Asian freshwaters	Freyhof & Herder (2001)	
Cyprinoidei	Tanichthyidae	*Tanichthys*	*Tanichthys albiventris*	Proportional dwarf	no	Asian freshwaters	Li et al. (2022)	
Cyprinoidei	Tanichthyidae	*Tanichthys*	*Tanichthys flavianalis*	Proportional dwarf	no	Asian freshwaters	Li et al. (2022)	
Cyprinoidei	Tanichthyidae	*Tanichthys*	*Tanichthys guipingensis*	Proportional dwarf	no	Asian freshwaters	Jin et al. (2022)	
Cyprinoidei	Tanichthyidae	*Tanichthys*	*Tanichthys huidongensis*	Proportional dwarf	no	Asian freshwaters	Jin et al. (2022)	
Cyprinoidei	Tanichthyidae	*Tanichthys*	*Tanichthys kuehnei*	Proportional dwarf	no	Asian freshwaters	Bohlen et al. (2019)	
Cyprinoidei	Tanichthyidae	*Tanichthys*	*Tanichthys luheensis*	Proportional dwarf	no	Asian freshwaters	Jin et al. (2022)	
Cyprinoidei	Tanichthyidae	*Tanichthys*	*Tanichthys shenzhenensis*	Proportional dwarf	no	Asian freshwaters	Jin et al. (2022)	

References

Anoop, V. K., Britz, R., Arjun, C. P., Dahanukar, N., & Raghavan, R. (2019). Pangio bhujia, a new, peculiar species of miniature subterranean eel loach lacking dorsal and pelvic fins from India (Teleostei: Cobitidae). Zootaxa, 4683(1). https://doi.org/10.11646/zootaxa.4683.1.8
Batuwita, S., de Silva, M., & Edirisinghe, U. (2013). A review of the danionine genera Rasboroides and Horadandia (Pisces: Cyprinidae), with description of a new species from Sri Lanka. Ichthyological Exploration of Freshwaters, 24(2), 121–140.
Bohlen, J., Dvorak, T., Thang, H. N., & Šlechtová, V. (2019). Tanichthys kuehnei, new species, from Central Vietnam (Cypriniformes: Cyprinidae). Ichthyological Exploration of Freshwaters, 29(1), 9–18.
Britz, R., Ali, A., & Raghavan, R. (2012). Pangio ammophila, a new species of eel-loach from Karnataka, southern India (Teleostei: Cypriniformes: Cobitidae). Ichthyological Exploration of Freshwaters, 23(1), 45–50.
Britz, R., Conway, K. W., & Rüber, L. (2021). The emerging vertebrate model species for neurophysiological studies is Danionella cerebrum, new species (Teleostei: Cyprinidae). Scientific Reports, 11(1), 18942. https://doi.org/10.1038/s41598-021-97600-0
Britz, R., & Kottelat, M. (2008). Paedocypris carbunculus, a new species of miniature fish from Borneo (Teleostei: Cypriniformes: Cyprinidae). Raffles Bulletin of Zoology, 56(2), 415–422.
Britz, R., & Kottelat, M. (2010). Pangio longimanus, a miniature species of eel-loach from central Laos (Teleostei: Cypriniformes: Cobitidae). Ichthyological Exploration of Freshwaters, 20(4).
Britz, R., Kottelat, M., & Tan, H. H. (2011). Fangfangia spinicleithralis, a new genus and species of miniature cyprinid fish from the peat swamp forests of Borneo (Teleostei: Cyprinidae). Ichthyological Exploration of Freshwaters, 22(4), 327–335.
Britz, R., Marak, W. K., Velentina, K., Lokeshwor, Y., Raghavan, R., Pinion, A. K., & Rüber, L. (2026). A miniature, subterranean, blind cobitid loach, Gitchak nakana, new genus and species, is the first groundwater-dwelling fish from Northeast India. Scientific Reports, 16(1), 7746. https://doi.org/10.1038/s41598-026-40425-6

Conway, K. W., & Kottelat, M. (2011). Boraras naevus, a new species of miniature and sexually dichromatic freshwater fish from peninsular Thailand (Ostariophysi: Cyprinidae). Zootaxa, 3002, 45–51. https://doi.org/10.11646/zootaxa.3002.1.4
Conway, K. W., & Kottelat, M. (2023). Physoschistura mango, a new miniature species of loach from Myanmar (Teleostei: Nemacheilidae). Raffles Bulletin of Zoology, 71, 681–701. https://doi.org/10.26107/RBZ-2023-0051
Conway, K. W., Kottelat, M., & Hui, T. H. (2011). Review of the Southeast Asian miniature cyprinid genus Sundadanio (Ostariophysi: Cyprinidae) with descriptions of seven new species from Indonesia and Malaysia. Ichthyological Exploration of Freshwaters, 22(3), 251–288.
Conway, K. W., Kubicek, K. M., & Britz, R. (2017). Morphological novelty and modest developmental truncation in Barboides, Africa's smallest vertebrates (Teleostei: Cyprinidae). Journal of Morphology, 278(6), 750–767. https://doi.org/10.1002/jmor.20670
Conway, K. W., & Moritz, T. (2006). Barboides britzi, a new species of miniature cyprinid from Benin (Ostariophysi: Cyprinidae), with a neotype designation for B. gracilis. Ichthyological Exploration of Freshwaters, 17(1), 73–84.
Daget, J., Gosse, J.-P., & Thys van den Audenaerde, D. F. E. (Eds.). (1984). Check-list of the freshhwater fishes of Africa (Vol. 1). ORSTOM - MRAC.
Freyhof, J., & Herder, F. (2001). Tanichthys micagemmae, a new miniature cyprinid fish from Central Vietnam (Cypriniformes: Cyprinidae). Ichthyological Exploration of Freshwaters, 12(3), 215–220.
Jiang, Y.-E., Chen, X.-Y., & Yang, J.-X. (2008). Microrasbora Annandale, a new genus record in China, with description of a new species (Teleostei: Cyprinidae). Environmental Biology of Fishes, 83(3), 299–304. https://doi.org/10.1007/s10641-008-9336-8
Jin, J., Li, C., & Zhao, J. (2022). Descriptions of six new species in White Cloud Mountain minnow Tanichthys albonubes complex (Cypriniformes: Tanichthyidae) in southern China. Journal of Fish Biology, 100(4), 1062–1087. https://doi.org/10.1111/jfb.15012
Kottelat, M. (2000). Diagnoses of a new genus and 64 new species of fishes from Laos (Teleostei: Cyprinidae, Balitoridae, Bagridae, Syngnathidae, Chaudhuriidae and Tetraodontidae). Journal of South Asian Natural History, 5(1), 37–82.
Kottelat, M. (2025). Barbucca heokhuii, a new species of loach from central Borneo (Teleostei: Barbuccidae). Raffles Bulletin of Zoology, 73, 54–66. https://doi.org/10.26107/RBZ-2025-0005
Kottelat, M., Britz, R., Tan, H. H., & Witte, K.-E. (2006). Paedocypris, a new genus of Southeast Asian cyprinid fish with a remarkable sexual dimorphism, comprises the world's smallest vertebrate. Proceedings of the Royal Society B: Biological Sciences, 273(1589), 895–899. https://doi.org/10.1098/rspb.2005.3419
Kottelat, M., & Vidthayanon, C. (1993). Boraras micros, a new genus and species of minute freshwater fish from Thailand (Teleostei: Cyprinidae). Ichthyological Exploration of Freshwaters, 4(2), 161–176.
Kottelat, M., & Witte, K.-E. (1999). Two new species of Microrasbora from Thailand and Myanmar, with two new generic names for small southeast Asian cyprinid fishes (Teleostei: Cyprinidae). Journal of South Asian Natural History, 4(1), 49–56.
Kullander, S. O., & Fang, F. (2009). Danio tinwini, a new species of spotted danio from northern Myanmar (Teleostei: Cyprinidae). Ichthyological Exploration of Freshwaters, 20(3), 223–228.
Li, F., Liao, T.-Y., Bohlen, J., Shen, Z.-X., Zhao, L.-J., & Li, S. (2022). Two new species of Tanichthys (Teleostei: Cypriniformes) from China. Journal of Vertebrate Biology, 71(21067). https://doi.org/10.25225/jvb.21067
Liao, T.-Y., & Tan, H. H. (2011). Brevibora cheeya, a new species of cyprinid fish from Malay Peninsula and Sumatra. Raffles Bulletin of Zoology, 59(1), 77–82.
Liao, T.-Y., & Tan, H. H. (2014). Brevibora exilis, a new rasborin fish from Borneo (Teleostei: Cyprinidae). Ichthyological Exploration of Freshwaters, 24(3), 209–215.
Ou, C., Montaña, C. G., Winemiller, K. O., & Conway, K. W. (2011). Schistura diminuta, a new miniature loach from the Mekong River drainage of Cambodia (Teleostei: Nemacheilidae). Ichthyological Exploration of Freshwaters, 22(3), 193–200.
Roberts, T. R. (2007). The "Celestial Pearl Danio", a new genus and species of colourful minute cyprinid fish from Myanmar (Pisces: Cypriniformes). Raffles Bulletin of Zoology, 55(1), 131–140.
Sundar, R. L., Arjun, C. P., Sidharthan, A., Dahanukar, N., & Raghavan, R. (2022). A new diminutive subterranean eel loach species of the genus Pangio (Teleostei: Cobitidae) from Southern India. Zootaxa, 5138(1), 89–97. https://doi.org/10.11646/zootaxa.5138.1.9
Tan, H. H. (2020). Trigonostigma truncata, a new species of harlequin rasbora from Malay Peninsula (Teleostei: Danionidae). Raffles Bulletin of Zoology, 68, 421–433. https://doi.org/10.26107/RBZ-2020-0058
Vasil'eva, E. D., & Vasil'ev, V. P. (2013). Two new species of Cyprinoid fishes from the fauna of Phu Quoc Island, Gulf of Thailand, Vietnam. Journal of Ichthyology, 53(5), 317–325. https://doi.org/10.1134/S0032945213030132

Table S14. Fitted transition-rate (Q) matrices for equal-rates (ER), symmetric (SYM), and all-rates-different (ARD) models of miniaturization-state evolution, estimated under two root-prior specifications (equal and fitzjohn).

Model	Root prior	From state	to non-miniature	to progenetic	to proportioned
ER	equal	non-miniature	-0.003632	0.001816	0.001816
		progenetic-miniature	0.001816	-0.003632	0.001816
		proportioned-dwarf	0.001816	0.001816	-0.003632
ER	fitzjohn	non-miniature	-0.003625	0.001812	0.001812
		progenetic-miniature	0.001812	-0.003625	0.001812
		proportioned-dwarf	0.001812	0.001812	-0.003625
SYM	equal	non-miniature	-0.00383	0	0.00387
		progenetic-miniature	0	-0.007779	0.007779
		proportioned-dwarf	0.00387	0.007779	-0.011649
SYM	fitzjohn	non-miniature	-0.00383	0.000516	0.003315
		progenetic-miniature	0.000516	-0.000516	0
		proportioned-dwarf	0.003315	0	-0.003315
ARD	equal	non-miniature	-0.002928	0	0.002928
		progenetic-miniature	0	0	0
		proportioned-dwarf	0.038792	0.003552	-0.042343
ARD	fitzjohn	non-miniature	-0.002756	0	0.002756
		progenetic-miniature	0	0	0
		proportioned-dwarf	0.038417	0.003393	-0.04181

Table S15. Comparison of evolutionary model fit for log-transformed genome size and body size across Cypriniformes. Models were fitted using the fitContinuous function in the R package geiger.

Trait	Model	sigsq	alpha	a	log-likelihood	AIC	AICc	deltaAICc	weight
log10GenomeSize	**BM**	**0.000324**			**344.596126**	**-685.192253**	**-685.153**	**0.619**	**0.3672**
	OU	0.000324	0		344.596126	-683.192253	-683.114	2.659	0.1324
	**EB**	**0.00091**		**-0.010328**	**345.925513**	**-685.851025**	**-685.772**	**0**	**0.5004**
	WN	0.02905			108.282147	-212.564293	-212.525	473.247	0
log10BodySize	BM	0.007054			-131.503969	267.007938	267.047	35.717	0
	**OU**	**0.010609**	**0.026546**		**-112.625856**	**231.251713**	**231.33**	**0**	**1**
	EB	0.007055		-0.000001	-131.50473	269.009461	269.088	37.758	0
	WN	0.206863			-195.006364	394.012727	394.052	162.722	0

Table S16. Comparison of Brownian motion (BM) and Ornstein–Uhlenbeck (OU) model fit within the phylogenetic generalized least squares (PGLS) regression framework for log-transformed genome size and body size across Cypriniformes.

		Brownian motion model			Ornstein Uhlenbeck model			
**Trait**	**Predictor**	**BM_log likelihood**	**BM_AIC**	**BM_AICw**	**OU_log likelihood**	**OU_AIC**	**OU_AICw**	**delta AIC**
log10GenomeSize	miniature vs non-miniature	**344.7**	**-683.4**	**0.95843673**	342.5	-677.1	0.04156327	6.276173
log10GenomeSize	non-miniature vs proportioned-dwarf vs progenetic-miniature	**345.8**	**-683.6**	**0.95611298**	343.7	-677.4	0.04388702	6.162515
log10GenomeSize	diploid vs polyploid	**379.9**	**-753.8**	**0.93879306**	378.2	-748.4	0.06120694	5.460669
log10GenomeSize	miniature vs non-miniature + Ploidy	**380**	**-751.9**	**0.93850085**	378.2	-746.5	0.06149915	5.450521
log10GenomeSize	non-miniature vs proportioned-dwarf vs progenetic-miniature + Ploidy	**380.8**	**-751.7**	**0.93396206**	379.2	-746.4	0.06603794	5.298413
log10GenomeSize	log10BodySize	**346**	**-686**	**0.95472774**	343.9	-679.9	0.04527226	6.097464
log10GenomeSize	log10BodySize + miniature vs non-miniature	**346**	**-684**	**0.95469239**	343.9	-677.9	0.04530761	6.095828
log10GenomeSize	log10BodySize + non-miniature vs proportioned-dwarf vs progenetic-miniature	**347**	**-684**	**0.95226423**	345	-678	0.04773577	5.986323
log10GenomeSize	log10BodySize + miniature vs non-miniature + Ploidy	**380**	**-750.1**	**0.93686869**	378.3	-744.7	0.06313131	5.394653
log10GenomeSize	log10BodySize + non-miniature vs proportioned-dwarf vs progenetic-miniature + Ploidy	**380.9**	**-749.8**	**0.93246352**	379.3	-744.5	0.06753648	5.250324
log10GenomeSize	log10BodySize * miniature vs non-miniature	**346**	**-682.1**	**0.95450069**	344	-676	0.04549931	6.086983
log10GenomeSize	log10BodySize * non-miniature vs proportioned-dwarf vs progenetic-miniature	**347.4**	**-680.7**	**0.95109488**	345.4	-674.8	0.04890512	5.935463
log10BodySize	miniature vs non-miniature	-113.3	232.6	3.10E-13	**-83.47**	**174.95**	**1.00E+00**	57.60758
log10BodySize	non-miniature vs proportioned-dwarf vs progenetic-miniature	-112.9	233.7	1.37E-13	**-82.25**	**174.5**	**1.00E+00**	59.24452

Table S17. Reference genome assemblies used in the analyses of transposable element content and gene architecture across miniature and non-miniature cypriniform species.

Suborder	Family	Genus	Species	Miniature	Source	Assembly.Accession	Assembly.Name	Total.Number.of. Chromosomes	Assembly.Level	WGS.project. accession	BioProject	BioSample	Publication
Catostomoidei	Catostomidae	*Myxocyprinus*	*asiaticus*	non-miniature	NCBI	GCF_019703515.2	UBuf_Myxa_2	50	Chromosome	JAIGOD02	PRJNA739167	SAMN19774990	https://doi.org/10.1093/gbe/evab190
Catostomoidei	Catostomidae	*Xyrauchen*	*texanus*	non-miniature	NCBI	GCF_025860055.1	RBS_HiC_50CHRs	50	Chromosome	JAOTQA01	PRJNA885108	SAMN31066171	
Cobitoidei	Cobitidae	*Misgurnus*	*anguillicaudatus*	non-miniature	NCBI	GCF_027580225.2	ASM2758022v2	25	Chromosome	JALDXJ02	PRJNA812369	SAMN26377316	https://doi.org/10.1186/s12915-023-01517-1
Cobitoidei	Cobitidae	*Paramisgurnus*	*dabryanus*	non-miniature	NCBI	GCF_030506205.2	PD_genome_1.1	24	Chromosome	JAUJEC02	PRJNA980909	SAMN35654119	https://doi.org/10.1038/s41597-025-04498-8
Cobitoidei	Nemacheilidae	*Triplophysa*	*dalaica*	non-miniature	NCBI	GCF_015846415.1	ASM1584641v1	25	Chromosome	JABZFK01	PRJNA624716	SAMN14582390	https://doi.org/10.1093/gbe/evab153
Cobitoidei	Nemacheilidae	*Triplophysa*	*rosa*	non-miniature	NCBI	GCF_024868665.1	Trosa_1v2	25	Chromosome	JAFHDT01	PRJNA673076	SAMN16587819	https://doi.org/10.1111/mec.16700
Cobitoidei	Nemacheilidae	*Triplophysa*	*tibetana*	non-miniature	NCBI	GCA_008369825.1	ASM836982v1	25	Chromosome	SOYY01	PRJNA526292	SAMN11094431	https://doi.org/10.1111/1755-0998.13021
Cyprinoidei	Cyprinidae	*Carassius*	*auratus*	non-miniature	NCBI	GCF_003368295.1	ASM336829v1	59	Chromosome	QPKE01	PRJNA481500	SAMN09670328	https://doi.org/10.1126/sciadv.aav0547
Cyprinoidei	Cyprinidae	*Carassius*	*carassius*	non-miniature	NCBI	GCF_963082965.1	fCarCar2.1	50	Chromosome	CAUJCD01	PRJEB64837	SAMEA11296534	
Cyprinoidei	Cyprinidae	*Carassius*	*gibelio*	non-miniature	NCBI	GCF_023724105.1	carGib1.2-hapl.c	50	Chromosome	JAJQLC01	PRJNA780760	SAMN23046545	https://doi.org/10.1038/s41467-022-31515-w
Cyprinoidei	Cyprinidae	*Cirrhinus*	*molitorella*	non-miniature	NCBI	GCA_033026305.1	C_molitorella v1.0	25	Chromosome	JAUYZG01	PRJNA1001164	SAMN36797183	https://doi.org/10.1038/s41597-024-04075-5
Cyprinoidei	Cyprinidae	*Cyprinus*	*carpio*	non-miniature	NCBI	GCF_018340385.1	ASM1834038v1	50	Chromosome	JAEOAB01	PRJNA682709	SAMN17005855	https://doi.org/10.1038/s41588-021-00933-9
Cyprinoidei	Cyprinidae	*Garra*	*rufa*	non-miniature	NCBI	GCF_049309525.1	GarRuf1.0	25	Chromosome	JBLIWC01	PRJNA1204062	SAMN46009517	https://doi.org/10.1038/s41597-025-05101-w
Cyprinoidei	Cyprinidae	*Labeo*	*rohita*	non-miniature	NCBI	GCF_022985175.1	IGBB_LRoh.1.0	25	Chromosome	JACTAM01	PRJNA650519	SAMN15716776	https://doi.org/10.1093/g3journal/jkad009
Cyprinoidei	Cyprinidae	*Onychostoma*	*macrolepis*	non-miniature	NCBI	GCF_012432095.1	ASM1243209v1	25	Chromosome	JAAMOB01	PRJNA608076	SAMN14163390	https://doi.org/10.1111/1755-0998.13190
Cyprinoidei	Cyprinidae	*Puntigrus*	*tetrazona*	non-miniature	NCBI	GCF_018831695.1	ASM1883169v1	25	Chromosome	JAEQBD01	PRJNA683758	SAMN17036344	https://doi.org/10.1038/s41588-021-00933-9
Cyprinoidei	Danionidae	*Boraras*	*brigittae*	proportioned-dwarf	Sudasinghe et al. (2026)	JBXUCT000000000	LR15871	25	Chromosome		PRJNA1393767	SAMN54311767	https://doi.org/10.64898/2026.03.21.713365
Cyprinoidei	Danionidae	*Danio*	*aesculapii*	non-miniature	NCBI	GCF_903798145.1	fDanAes4.1	25	Chromosome	CAIGQR01	PRJEB38583	SAMEA104240212	
Cyprinoidei	Danionidae	*Danio*	*rerio*	non-miniature	NCBI	GCF_049306965.1	GRCz12tu	25	Complete Genome	JBMGRA01	PRJNA1193077	SAMN43570788	https://doi.org/10.1101/2025.11.17.688901
Cyprinoidei	Danionidae	*Danionella*	*cerebrum*	progenetic-miniature	NCBI	GCA_007224835.1	ASM722483v1		Scaffold	SRMA01	PRJNA445947	SAMN11288624	https://doi.org/10.1038/s41597-019-0161-z
Cyprinoidei	Danionidae	*Rasbora*	*kalochroma*	non-miniature	Sudasinghe et al. (2026)	JBXUDC000000000	LR15877	25	Chromosome		PRJNA1393767	SAMN54311768	https://doi.org/10.64898/2026.03.21.713365
Cyprinoidei	Gobionidae	*Pseudorasbora*	*parva*	non-miniature	NCBI	GCF_024679245.1	ASM2467924v1	25	Chromosome	JANLNJ01	PRJNA843929	SAMN28772710	
Cyprinoidei	Leuciscidae	*Leuciscus*	*chuanchicus*	non-miniature	NCBI	GCA_965140175.1	ASM96514017v1		Scaffold	CBCSLH01	PRJEB85705	SAMEA117683954	https://doi.org/10.1038/s41597-025-04787-2
Cyprinoidei	Leuciscidae	*Leuciscus*	*waleckii*	non-miniature	NCBI	GCA_041200155.1	ASM4120015v1	25	Chromosome		PRJNA929316	SAMN32957118	
Cyprinoidei	Leuciscidae	*Phoxinus*	*phoxinus*	non-miniature	NCBI	GCA_037504875.1	fPhoPho.hap1	25	Chromosome	JAYKXG01	PRJNA1030284	SAMN37908444	https://doi.org/10.1093/gigascience/giae116
Cyprinoidei	Leuciscidae	*Pimephales*	*promelas*	non-miniature	NCBI	GCF_016745375.1	EPA_FHM_2.0		Scaffold	WIOS01	PRJNA565199	SAMN12875914	https://doi.org/10.1002/etc.5266
Cyprinoidei	Paedocyprididae	*Paedocypris*	sp	progenetic-miniature	Sudasinghe et al. (2026)	JCBLRK000000000	LR15863A	15	Chromosome		PRJNA1393767	SAMN54311765	https://doi.org/10.64898/2026.03.21.713365
Cyprinoidei	Sundadanionidae	*Sundadanio*	*atomus*	progenetic-miniature	Sudasinghe et al. (2026)	JBXUCS000000000	LR15870	24	Chromosome		PRJNA1393767	SAMN54311766	https://doi.org/10.64898/2026.03.21.713365
Cyprinoidei	Xenocyprididae	*Chanodichthys*	*erythropterus*	non-miniature	NCBI	GCF_024489055.1	ASM2448905v1	24	Chromosome	JALPSW01	PRJNA827856	SAMN27624467	https://doi.org/10.1038/s41597-022-01648-0
Cyprinoidei	Xenocyprididae	*Ctenopharyngodon*	*idella*	non-miniature	NCBI	GCF_019924925.1	HZGC01	24	Chromosome	JAIKLG01	PRJNA745929	SAMN20170273	https://doi.org/10.1186/s12864-022-08503-x
Cyprinoidei	Xenocyprididae	*Culter*	*alburnus*	non-miniature	NCBI	GCA_040182925.1	ASM4018292v1	24	Chromosome	JAWDJR01	PRJNA1019486	SAMN37486085	
Cyprinoidei	Xenocyprididae	*Megalobrama*	*amblycephala*	non-miniature	NCBI	GCF_018812025.1	ASM1881202v1	24	Chromosome	JAGTWI01	PRJNA343584	SAMN18522313	https://doi.org/10.1093/molbev/msab152
Gyrinocheiloidei	Gyrinocheilidae	*Gyrinocheilus*	*aymonieri*	non-miniature	NCBI	GCA_051167395.1	ASM5116739v1	24	Chromosome	JBPGVV01	PRJNA1269254	SAMN48783300	https://doi.org/10.1038/s41597-025-06070-w

Dataset S1 (separate file). GenomeScope genome-profiling estimates and GC content for the k-mer profiling dataset (n = 261).
